# Diels–Alder Reactions During the Biosynthesis of Sorbicillinoids

**DOI:** 10.1002/anie.201915486

**Published:** 2020-02-04

**Authors:** Lukas Kahlert, Eman F. Bassiony, Russell J. Cox, Elizabeth J. Skellam

**Affiliations:** ^1^ Institute for Organic Chemistry and BMWZ Leibniz University of Hannover Schneiderberg 38 30167 Hannover Germany; ^2^ Biochemistry Department Faculty of Science Zagazig University Zagazig Ash Sharqia Governorate 44519 Egypt

**Keywords:** biosynthesis, Diels–Alder reactions, flavin-dependent monooxygenases, polyketides, sorbicillinoids

## Abstract

The sorbicillinoids are a class of biologically active and structurally diverse fungal polyketides arising from sorbicillin. Through co‐expression of sorA, sorB, sorC, and sorD from Trichoderma reesei QM6a, the biosynthetic pathway to epoxysorbicillinol and dimeric sorbicillinoids, which resemble Diels–Alder‐like and Michael‐addition‐like products, was reconstituted in Aspergillus oryzae NSAR1. Expression and feeding experiments demonstrated the crucial requirement of the flavin‐dependent monooxygenase SorD for the formation of dimeric sorbicillinoids, hybrid sorbicillinoids, and epoxysorbicillinol in vivo. In contrast to prior reports, SorD catalyses neither the oxidation of 2′,3′‐dihydrosorbicillin to sorbicillin nor the oxidation of sorbicillinol to oxosorbicillinol. This is the first report that both the intermolecular Diels–Alder and Michael dimerization reactions, as well as the epoxidation of sorbicillinol are catalysed in vivo by SorD.

## Graphical abstract

**One for all**: The biosynthetic pathway to dimeric sorbicillinoids and epoxysorbicillinol was reconstituted in *Aspergillus oryzae* NSAR1. Expression and feeding experiments demonstrated that the flavin‐dependent monooxygenase SorD catalyzes epoxidation, Diels–Alder addition, and Michael addition reactions in vivo.

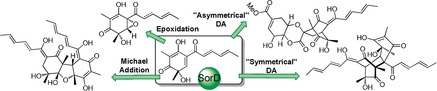

## Introduction

**Scheme 1:**
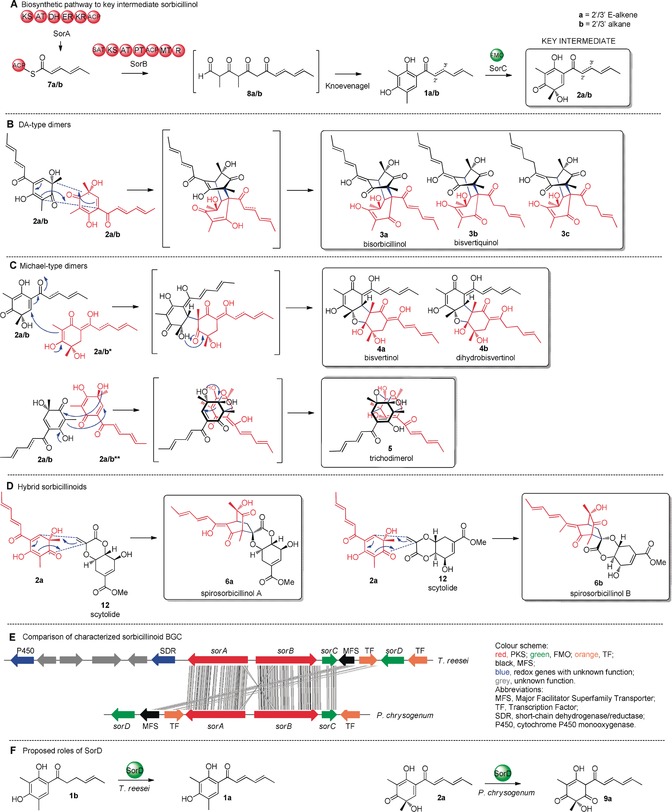
A) Sorbicillinol biosynthetic pathway. B–D) Representative sorbicillinoid metabolites arising from the key intermediate sorbicillinol **2 a**. E) Comparison of the *T. reesei* QMa6 and *P. chrysogenum* sorbicillinoid BGC. F) Proposed roles of SorD. New C−C bonds formed by Diels–Alder (DA)‐ or Michael‐addition‐like reactions are shown in blue. The **a**/**b** nomenclature denotes 2′/3′ E‐alkene or 2′/3′alkane functionality, respectively. **a**/b* and **a**/b** indicate tautomeric forms.

Sorbicillinoids are an important family of hexaketides produced by terrestrial^1^, ^2^ and marine^3^, ^4^, ^5^ fungi.^6^ Sorbicillin (**1 a**) was first isolated from *Penicillium notatum* by Cram and Tishler in 1948.^7^, ^8^ **1 a** is oxidatively dearomatised to form sorbicillinol (**2 a**; Scheme 1 A),^9^ which reacts with itself and other compounds to form various sorbicillinoids (Scheme 1), more than 90 of which are known.^10^, ^11^, ^12^ Trifonov first proposed **2 a** as the intermediate for self‐dimerization due to its dual diene and dienophile character.^2^, ^13^, ^14^ This was confirmed by Abe et al. through elegant feeding experiments.^15^ Dimeric sorbicillinoids include bisorbicillinol **3 a**, which is formed by an intermolecular Diels–Alder (DA) reaction (Scheme 1 B) and displays radical‐scavenging activity almost matching that of α‐tocopherol.^16^ Other dimers include the Michael‐addition‐like products bisvertinol **4a** (Scheme 1 C)^17^ and trichodimerol **5** (Scheme 1 D), a potent inhibitor of prostaglandin biosynthesis.^18^ Hybrid sorbicillinoids such as spirosorbicillinols A and B **6 a** and **6 b** (Scheme 1 E),^19^ are formed by DA reaction of **2 a** with different dienophiles.

A breakthrough was achieved when the sorbicillinoid biosynthetic gene cluster (BGC) was discovered in *P. chrysogenum*. The BGC encodes a highly reducing iterative polyketide synthase (hr‐PKS, SorA), a non‐reducing iterative PKS (nr‐PKS, SorB), an FAD‐dependent monooxygenase (FMO, SorC), and a second FMO (SorD).^9^ SorA was proposed to synthesize the triketide intermediate **7**, which remains tethered to the ACP domain due to lack of any release domain (Scheme 1 A). Two intermediates, **7 a** and **7 b**, were proposed to be formed depending on whether the SorA enoyl reductase (ER) domain acts during the second cycle of polyketide chain elongation. The starter acyl transferase (SAT) domain of SorB loads **7** and elongates this starter unit three more times, introducing two methyl groups with its C‐methyltransferase (C‐MeT) domain. Reductive release from SorB yields aldehyde **8**, which undergoes Knoevenagel condensation to give the first isolatable intermediates sorbicillin (**1 a**) and 2′,3′‐dihydrosorbicillin (**1 b**). SorC was shown to stereoselectively catalyse the oxidative dearomatisation of **1 a** to **2 a** and **1 b** to **2 b**.^9^ In subsequent studies, knockout of *sorA* by the Dreissen group abolished production of **1** and all related sorbicillinoids.^20^

The only other confirmed homologous sorbicillinoid BGC exists in *T. reesei* QM6a^21^ and shares the three core genes *sorA*, *sorB* and *sorC* required for formation of **2** (Scheme 1 E).^9^, ^20^, ^21^, ^22^ The *T. reesei* BGC also encodes a second FMO named SorD. Derntl et al.^21^ proposed that *T. reesei* SorD catalyses the oxidation of **1 b** to **1 a** (Scheme 1 F). In contrast, Guzmán‐Chávez et al.^22^ suggested that *P. chrysogenum* SorD catalyses the oxidation of **2 a** into oxosorbicillinol **9 a** (Scheme 1 F), since increased amounts of **2 a** were produced by their Δ*sorD* strain. ARTEMIS analysis shows that the two *sorD* genes are not homologous (Scheme 1 E), thus indicating that they may catalyse different reactions in *T. reesei* and *P. chrysogenum*, despite both fungi synthesizing a similar variety of sorbicillinoids.^20^, ^21^ However, the precise role of SorD has not yet been elucidated.

Dimeric sorbicillinoids are also formed during organic extraction and workup. Corey et al.^23^ observed that **2 a** is highly reactive and the dimer **5** was isolated after silica‐gel chromatography with 3:1 hexane/EtOAc. Shortly afterwards, Abe et al.^10^ reported spontaneous dimerization of **2 a** to **3 a** during liquid–liquid extraction with EtOAc and formation of **5** during lyophilization of **2 a**. Recently, Gulder et al.^24^, ^25^ showed conclusively that in vitro production of **2 a** by SorC, forms different dimeric sorbicillinoids depending on the organic co‐solvent added to the reaction. For instance, addition of 20 % *v/v* acetone yielded **3 a** (27 %) after extraction with CH_2_Cl_2_, whereas addition of 20 % *v/v* dimethylformamide (DMF) yielded **5** (27 %) after extraction with CH_2_Cl_2_. However, the confirmed formation of dimeric sorbicillinoids from **2 a** in vitro in the presence of organic solvents does not rule out the possibility of the existence of in vivo catalysts that can also form these compounds.

Since questions remain regarding the precise role of SorD and other enzymes, for example, a P450 and an SDR, encoded by genes adjacent to the *T. reesei* sorbicillinoid BGC (Scheme 1 E), we set out to investigate the BGC in more detail.

## Results and Discussion

**Figure 1:**
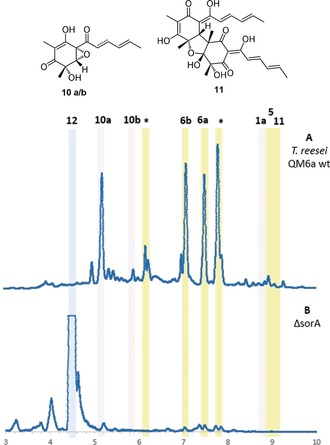
LCMS (DAD 210–600 nm) analysis of wt (A) and *Δ*sorA (B) *T. reesei* QM6a. * denotes uncharacterized sorbicillinoid‐related compounds (Figure S4 and S5). Dimeric sorbicillinoids are highlighted in yellow; monomeric sorbicillinoids are highlighted in grey; non‐sorbicillinoids are highlighted in blue.

Sorbicillinoid production in *T. reesei* QM6a was confirmed by fermentation and analysis by liquid‐chromatography mass spectrometry (LCMS, Figure 1 A). Numerous sorbicillinoid‐related compounds were purified and identified based on UV, HRMS, and 1D and 2D NMR analysis (see the Supporting Information for details). These include the monomer **1 a**, as well as epoxysorbicillinols **10 a** and **10 b**. The reactive intermediate **2 a** could not be purified. The hybrid sorbicillinoid spirosorbicillinol B (**6 b**) was also identified and fully elucidated. A number of other dimeric sorbicillinoids were identified based on their UV and MS profiles, including bisvertinolone (**11**),^14^ **5,** 26 and **6 a** (Figure S1–S3), as well as several dimers that could not be fully characterized (* in Figure 1 A, Figures S4, S5). Compounds **6 a** and **6 b** are proposed to arise through the reaction of **2 a** with scytolide (**12**),^19^ which has not previously been reported in *T. reesei*. Knockout of *sorA* completely abolished production of all sorbicillinoids, but led to the production of **12** (Figure 1 B).

**Figure 2:**
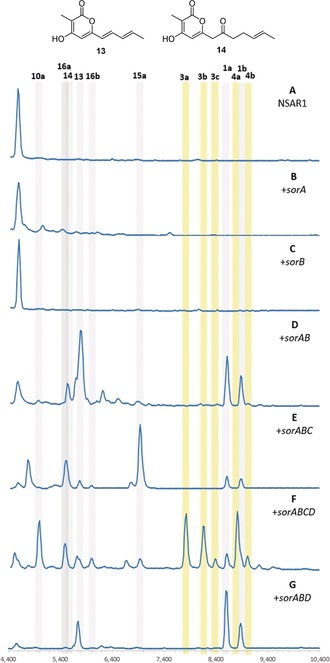
LCMS analysis [DAD 210–600 nm] of *A. oryzae* expressing different combinations of genes from the sorbicillinoid biosynthetic gene cluster. Dimeric sorbicillinoids are highlighted in yellow; monomeric sorbicillinoids are highlighted in grey.

The sorbicillinoid biosynthetic pathway was reconstituted in the heterologous host *Aspergillus oryzae* NSAR1 from *T. reesei* QM6a cDNA templates. Compared to the NSAR1 control (Figure 2 A), transformants expressing *sorA* (Figure 2 B) and *sorB* (Figure 2 C) individually did not produce any new compounds (Scheme 1 A). However, LCMS analysis of *A. oryzae*+*sorAB* transformants revealed four new peaks, of which two corresponded to **1 a** and **1 b** (Figure 2 D). The known trichopyrone (**13**)^27^ and the related pyrone **14** were also produced. Pyrones **13** and **14** are likely premature off‐loading shunt products of SorB, since similar products are known to be formed when methylation steps are not properly completed by nr‐PKS.^28^

Next, *sorC* was added to *sorAB* in the expectation of forming **2 a**. However, *A. oryzae*+*sorABC* produced **15 a** as the major product (Figure 2 E). The UV absorption of **15 a** is distinct from that of **2 a**,^9^ and the *m*/*z* [*M*−H]^−^ of 249 indicated a reduction. Thorough NMR analysis of **15 a** and its mono‐methylated derivative (Me_3_SiCHN_2_)^29^ confirmed the structure of the reduced sorbicillinol **15 a**. The *A. oryzae*+*sorABC* transformants also produced the vertinolides **16 a** 13 and **16 b**. The absolute stereochemistries of **16 a** and **16 b** were assigned based on comparison with data reported by Takaia and Yamashita.^30^

**Scheme 2:**
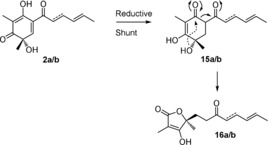
Proposed pathway for the reduction of **2** by *A. oryzae* leading to the formation of **15** and **16**.

SorC therefore catalyses the oxidative dearomatisation of **1** to **2** as expected, however **2** appears to be reduced to **15**. This is likely the result of an unknown enzyme in *A. oryzae* since this host is known to reduce other heterologously produced intermediates.^31^, ^32^, ^33^ Vertinolides **16** are also derived from **15**. The furanone backbone of **16** presumably originates from a retro‐aldol like ring opening or an intramolecular rearrangement of **15** (Scheme 2). A very similar skeletal rearrangement has been observed during the synthesis of paclitaxel derivatives.^34^ No formation of any dimeric sorbicillinoids was observed in any of the +*sorABC* transformants.

Heterologous expression of +*sorABCD* resulted in many new peaks, which were identified as dimeric sorbicillinoids originating from either DA‐like (**3 a**–**c**) or Michael‐addition‐like (**4 a**,**b**) dimerization of **2 a** and/or **2 b** (Figure 2 F). All compounds were fully elucidated using NMR, except for **4 b**, which was characterized according to UV absorption and MS data (Figure S6). Compound **3 c** must arise from a DA dimerisation of **2 b**. In addition to the dimeric sorbicillinoids, oxidation to the epoxysorbicillinols **10 a** and **10 b** was observed for the first time (Figure 2 F). The production of **15 a** is minimal, thus suggesting that in the absence of SorD, **2 a** is quickly reduced by *A. oryzae*, however when SorD is present, **2 a**/**b** can be efficiently converted into dimers **3 a**–**c** and **4 a**,**b** (Scheme 1 B,C) or oxidized to **10 a**/**b**.

Although **2 a** is known to be inherently unstable, spontaneous formation of dimeric compounds during the extraction process is unlikely because the +*sorABC* and +*sorABCD* transformants had all been cultured and extracted under identical conditions, yet no dimeric compounds were observed in +*sorABC* transformants (Figure 2 E). To exclude the possibility that these dimers formed during the extraction procedure, the crude culture supernatant of +*sorABCD* transformants was directly subjected to LCMS analysis prior to extraction, and it showed the same qualitative composition (Figure S10). Therefore, under the in vivo conditions used, dimerization of **2 a**/**b** is independent of any added solvents and can be attributed to the presence of SorD.

LCMS chromatograms obtained from +*sorABD* transformants (Figure 2 G) did not significantly differ from those for +*sorAB* transformants, thus indicating that **2 a** and **2 b** are the required substrates for SorD. The observation of both **1 a** and **1 b** in these transformants indicates that SorD does not oxidize **1 b** to **1 a** as proposed by Derntl et al.^21^ Similarly, since the oxosorbicillinols **9 a** and **9 b** were not observed in any of the chromatograms, SorD does not seem to have a role in oxidizing **2** to **9** as proposed by Guzmán‐Chávez et al.^22^ This shows that SorD has two roles: dimerization of **2** to **3** and **4**, which does not require oxidation of **2**, and an independent role in oxidizing **2 a**/**b** to epoxides **10 a**/**b**.

To further investigate the role of SorD in dimerization, we attempted in vitro assays. Extensive attempts were made to obtain soluble SorD, but all efforts at expression in either *E. coli* or *S. cerevisiae* resulted in insoluble and inactive protein. Experiments with cell‐free extract (CFE) or whole cells of these organisms expressing SorD also met with failure.

In contrast, we were able to obtain soluble his_6_‐SorC (52.8 kDa) in very high yields (180 mg L^−1^), and in vitro assays were performed as described previously.^9^ Substrates **1 a**/**b** were purified from +*sorAB* transformants. As expected, substrates **1 a** and **1 b** were quickly converted into **2 a** and **2 b** (Figure S11). In contrast, when the assays were performed under the specific conditions as described by Gulder et al.^24^ in the presence of 20 % *v*/*v* acetone followed by extraction of the assay mixture with CH_2_Cl_2_ or CHCl_3_, dimers **3 a**–**c** were formed (Figure S11). These observations are in accordance with previous reports^10^, ^23^, ^24^, ^25^ that under specific conditions, dimerization of **2 a** can be induced by organic solvents.

When an excess of NAD(P)H or prolonged incubation times were employed in the SorC assays, small amounts of **15 a** or **10 a**/**b** could be detected (Figure S12). Therefore, the reduction of **2** to **15** in the heterologous expression experiments may not even require an enzyme. Although the reduction of **2** prevents subsequent dimerization reactions, the reduction of **2** in vivo must be faster than any spontaneous dimerization reactions.

**Figure 3:**
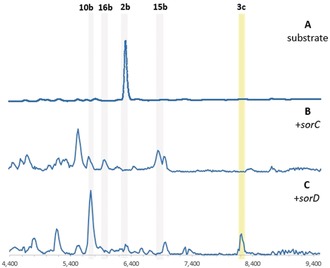
LCMS analysis [DAD 210–600 nm] of *A. oryzae* expressing either *sorC* (B) or *sorD* (C) fed with compound **2 b**. Dimeric sorbicillinoids are highlighted in yellow; monomeric sorbicillinoids are highlighted in grey.

We next investigated the individual functions of SorC and SorD in vivo. Since **2** cannot be purified from our heterologous expression experiments due to its conversion to **15**, we used our in vitro assay with SorC to obtain **2**. Substrates **1 a**/**b** were incubated with SorC leading to the formation of compounds **2 a**/**b**. Dihydrosorbicillin (**2 b**) was purified (Figure 3 A) and supplemented to *A. oryzae* expressing either *sorC* or *sorD*. When **2 b** was fed to *A. oryzae* expressing *sorC* and cultured and extracted using standard conditions, no dimeric compounds were detected. Instead, compounds **15 b** and **16 b** were detected as well as a small amount of **10 b** (Figure 3 B). **2 b** is therefore directly converted into **15 b** and **16 b** (Scheme 2), thus proving that dimerization is not spontaneous and nor is it catalysed by SorC under the conditions investigated.

When **2 b** was fed to *A. oryzae* expressing *sorD* under identical conditions, the dimeric sorbicillinoid **3 c** was detected (Figure 3 C). Since all other dimeric compounds form from at least one molecule of **2 a**, no other dimeric molecules were detected. **10 b** was also detected but **15 b** was not (Figure 3 C), thus demonstrating conclusively that in the presence of SorD, **2 b** is efficiently dimerized to **3 c** or epoxidized to **10 b**. Clearly SorD, catalyzes distinct epoxidation or dimerization reactions, both of which are faster than the reduction of **2 b** to **15 b** either by *A. oryzae* or intracellular NAD(P)H.

**Figure 4:**
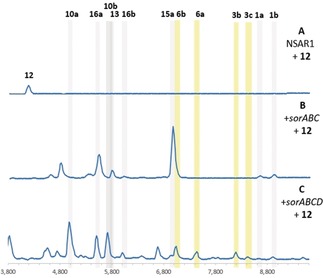
LCMS analysis (DAD 210–600 nm) of *A. oryzae* NSAR1 (A; control) and transformants expressing *sorABC* (B) or *sorABCD* (C) fed with scytolide (**12**). Dimeric sorbicillinoids are highlighted in yellow; monomeric sorbicillinoids are highlighted in grey.

The hybrid sorbicillinoids **6 a**/**b** are considered to derive from dimerization of **2 a** with **12** (Scheme 1 D).^19^ To further investigate the role of SorD in the dimerization of **2**, scytolide (**12**) was purified from *T. reesei* Δ*sorA* (Figure 1 B) and fed to *A. oryzae* strains and then extracted using standard conditions. **12** remained unaltered in the NSAR1 control (Figure 4 A). In strains expressing +*sorABC*, no conversion to **6** was observed. The major product remained the reduced shunt product **15 a**, thus indicating that **2** does not spontaneously undergo a Diels–Alder reaction with **12**, nor does SorC catalyse the reaction (Figure 3 B). However, when **12** was fed to +*sorABCD* transformants, **6 a** and **6 b** were observed (Figure 3 C). These results demonstrate that **6 a** and **6 b** are exclusively formed in the presence of sorD.

Surprisingly, compounds **5** and **11**, which were observed in *T. reesei* QM6a, were not detected in any of our expression strains. Compound **5** is proposed to arise from two consecutive Michael‐like additions (Scheme 1 C). That **5** is not observed further supports these dimerization mechanisms not being spontaneous and requiring an enzyme catalyst. Compound **11** is a dimer of **2 a** and **9 a**, however the formation of **9 a** appears to be independent of *SorABCD*. To be certain that the additional genes encoding an SDR and P450 are not required for the production of **5** and/or **11**, both genes were successfully disrupted in *T. reesei* using the bipartite knock‐out strategy (Figure S18).^35^ However, no changes in the metabolite profile were observed. Similarly, heterologous expression of the SDR and P450 genes with *+sorABCD* in *A. oryzae* did not yield any new pathway intermediates (Figure S19). This indicates that genes encoding the enzymes necessary for the formation of **5** and **11** are located elsewhere in the genome of *T. reesei*.

## Conclusion

**Scheme 3:**
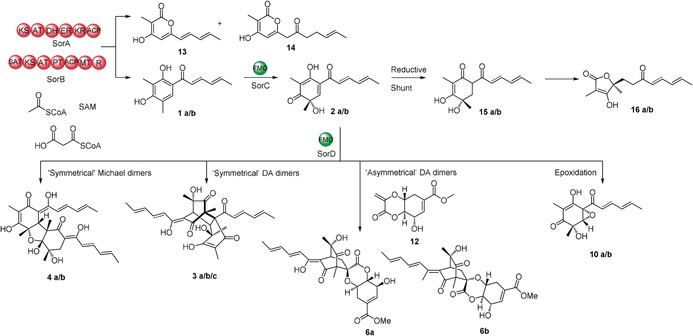
Summary of the sorbicillinoid biosynthetic pathway in *T. reesei* QM6a. The dual‐function FMO SorD catalyses both Diels–Alder‐ and Michael‐addition‐like dimerizations, as well as epoxidation of **2**.

**Scheme 4:**
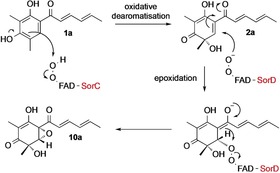
Putative mechanism for the oxidative dearomatisation of **1 a** to **2 a** by SorC and its subsequent epoxidation to **10 a** catalysed by SorD.

Our results demonstrate that the SorAB PKS system of *T. reesei* QM6a behaves as expected to produce **1** in the heterologous host *A. oryzae* (Scheme 3). The FMO SorC catalyses the oxidative dearomatisation of **1** to **2** in vitro as expected. Sorbicillinol (**2**) can also be reduced to **15** in vitro by NAD(P)H alone. In vivo, SorABC produces **2**, which is also reduced to **15**. Although SorC can catalyse consecutive oxidation of **2** to epoxysorbicillinol (**10**) in vitro, the second oxidation is very inefficient. This epoxidation is primarily catalysed in vivo by the FMO SorD. The requirement for two different enzymes may reflect the differences in mechanism of the two oxidation reactions. During the oxidative dearomatisation of **1** to **2**, the enzyme bound‐hydro‐peroxyflavin species acts as an electrophile, whereas during the epoxidation of **2** to **10**, the peroxyflavin species would be expected to act as a nucleophile (Scheme 4). Gulder et al. reported a similar facile reaction of **2 a** with ^*t*^BuO_2_H.^25^

Dimeric sorbicillinoids derived from **2** were only detected in vivo in the presence of SorD. SorD is also able to epoxidize **2** to **10**, which is independent of the dimerization mechanism. These dimeric compounds were previously described as resulting from organic‐solvent‐induced spontaneous reactions. However, in our hands, we developed extraction and purification conditions, which do not catalyse these dimerization reactions. Under these conditions but in the presence of SorD, dimeric products were observed, including “symmetrical” Michael‐addition dimers such as **4**, “symmetrical” Diels–Alder reaction dimers such as **3**, and “asymmetrical” Diels–Alder reaction dimers such as **6 a** and **6 b** (Scheme 3). Therefore, we reveal a role of SorD in dimerizing the highly reactive intermediate **2** and demonstrate the scope of the reactions catalysed by this enzyme. Furthermore, we clearly demonstrate that the isomers spirosorbicillinol A (**6 a**) and B (**6 b**) arise from **2 a** and the shikimic acid derivative **12**, thereby confirming their biosynthetic origin.

Formation of epoxides is a common reaction catalysed by FMOs,^36^, ^37^, ^38^, ^39^, ^40^, ^41^ but catalysis of DA reactions is much rarer. Fujii and co‐workers reported a dual‐acting flavin‐dependent enzyme, Sol5, which catalyses both an alcohol oxidation and an *intramolecular* DA cyclisation during the biosynthesis of solanapyrone.^42^ In contrast, SorD catalyses *intermolecular* DA and Michael reactions, which do not require initial oxidation. SorD is the first flavin‐dependent enzyme reported to behave in this way.

## Conflict of interest

The authors declare no conflict of interest.

## Supporting information

As a service to our authors and readers, this journal provides supporting information supplied by the authors. Such materials are peer reviewed and may be re‐organized for online delivery, but are not copy‐edited or typeset. Technical support issues arising from supporting information (other than missing files) should be addressed to the authors.

Supplementary
