# Supplementary material for: Diels–Alder Reactions During the Biosynthesis of Sorbicillinoids
Source: Angew Chem Int Ed Engl. 2020 Feb 4;59(14):5816–22. doi: 10.1002/anie.201915486 (PMC7154774; doi:10.1002/anie.201915486)
Supplement: Supplementary file 1 — Supplementary [file ANIE-59-5816-s001.pdf]

Angewandte Chemie
Eine Zeitschrift der Gesellschaft Deutscher Chemiker

Supporting Information

Diels–Alder Reactions During the Biosynthesis of Sorbicillinoids

*Lukas Kahlert, Eman F. Bassiony, Russell J. Cox, and Elizabeth J. Skellam**

anie_201915486_sm_miscellaneous_information.pdf

Table of Contents

General Information 2

Experimental procedures 2

Tables 7

Figures 11

Sorbicillinoid Compounds Identified by Mass and UV Absorption Profiles 11

Figure S1: Spirosorbicillinol A **6a** 11
Figure S2: Trichodimerol **5** 12
Figure S3: Bisvertinolone **11** 13
Figure S4: Unknown sorbicillinoid related compound in Fig. 1A marked with * eluting at approx. 6.2 min. 14
Figure S5: Unknown sorbicillinoid related compound in Fig. 1A marked with * eluting at approx. 7.8 min. 14
Figure S6: Dihydrobisvertinol **4b** 15
Figure S7: Sorbicillinol **2a** 16
Figure S8: Dihydrosorbicillinol **2b** 17
Figure S9: Reduced dihydrosorbicillinol **15b** 18

LCMS Chromatograms 20

***In Vitro* Experimental Design and Results** 21

***In Vivo* Experimental Design and Results** 24

Compound Physical Data 26

References 123

General information

Reagents

Analytical grade chemicals and reagents were purchased from Sigma, Roth and Fischer. The solvents used for HPLC were analytical grade. General molecular biology procedures were performed as standard and molecular biology kits were used according to the manufacturer's protocols. Analytical PCR was performed using OneTaq polymerase and preparative PCR for cloning procedures was performed using Q5 polymerase manufactured by NEB. Restriction endonucleases were also purchased from NEB.

Media

All media were prepared in deionised water and autoclaved at 126 °C for 20 min. See Table 1 for details.

Analytical LCMS

LCMS data were obtained with either (LCMS method 1): a Waters 2795HT HPLC using a Phenomenex Kinetex column (2.6µ,C18, 100 Å, 4.6 × 100mm) equipped with a Phenomenex Security Guard precolumn (Luna C5 300 Å) eluting at 1 mL min^{-1}, with a Waters 996 Diode Array detector between 210 and 600 nm and a Waters ZQ mass detector operating simultaneously in ES^+ and ES^- modes between 100 and 1000 m/z; or (LCMS method 2): a Waters 2767 sample manager connected to Waters 2545 pumps and SFO, a Phenomenex Kinetex column (2.6µ,C18, 100 Å, 4.6 × 100 mm) equipped with a Phenomenex Security Guard precolumn (Luna C5 300 Å) eluted at 1.0 mL min^{-1}, with a waters 2998 Diode Array detector (210–600 nm) and Waters 2424 ELSD and Waters SQD-2 mass detector operating simultaneously in ES^+ and ES^- modes between 100 and 1000 m/z. Solvents were: **A**, HPLC-grade H_2O containing 0.05% formic acid and **B**, HPLC-grade acetonitrile containing 0.045% formic acid. The gradient was run over 15 min starting at 10 % **B** and ramping to 90 % **B** within the first ten minutes, followed by two minutes at 90 % **B** and three minutes at 10 % **A**.

Preparative LCMS

Purification of all compounds was generally achieved using a Waters mass-directed autopurification system comprising of a Waters 2767 autosampler, Waters 2545 pump system, a Phenomenex Kinetex Axia column (5µ, C18, 100 Å, 21.2 × 250 mm) equipped with a Phenomenex Security Guard pre- column (Luna C5 300 Å) eluted at 20 mL min^{-1} at ambient temperature. Solvents were used as above. The post-column flow was split (100:1) and the minority flow was made up with HPLC-grade MeOH + 0.045% formic acid to 1 mL min^{-1} for simultaneous analysis by diode array (Waters 2998), evaporative light-scattering (Waters 2424) and electrospray ionisation mass spectrometry in positive and negative modes (Waters SQD-2). Detected peaks were collected into glass test tubes. Combined fractions were evaporated (vacuum centrifuge), weighed and residues dissolved directly in deuterated solvent for NMR.

HRMS and NMR

HRMS was obtained using a Waters Acquity Ultraperformance UPLC system connected to a Q-TOF Premier mass spectrometer. NMR data were recorded using either a Bruker Ascend 400, a Bruker Ultrashield 500 or a Bruker Ascend 600 instrument each equipped with a cryo-cooled probe at 400/ 500/ 600 MHz (^1H) and 125 MHz (^{13}C) and are referenced relative to Me_4Si. 2D spectra (COSY, HSQC and HMBC) were recorded using standard parameters. Samples were dissolved in deuterated as solvents indicated in the respective NMR tables and figures.

Experimental Procedures

Fermentation and Extraction Protocols

A. oryzae: A spore suspension of transformants grown on selective agar plates (CZD/S, CZD/S1 or CZD/S1 w/o methionine) was inoculated into 100 mL DPY-medium in a 500 mL baffled flask and incubated for four days at 28 °C with 110 rpm shaking. Cultures were homogenized using a hand blender and cells were separated by filtration. The liquid supernatant was acidified with 2 M HCl to pH 3-4 and extracted twice with ethyl acetate (2 x 100 mL). Combined organic layers were dried over $MgSO_4$ and solvent was removed under reduced pressure. The organic residue was dissolved in methanol to a concentration of 10 mg/mL, filtered over glass wool and analysed by LCMS. For preparative LCMS cultures were grown in a scale between 1-2 L and the concentration of the organic extract

applied for purification was 50 mg/mL.
T. reesei: A spore solution of the wild type strain or transformants grown on PDB-agar plates or PDB-agar plates supplemented with hygromycin B (100 µg/mL), respectively, were inoculated into 100 mL ME-medium in a 500 mL flask and incubated for seven days at 28 °C with 180 rpm shaking. Cultures were homogenized using a hand blender and cells were separated by filtration. The liquid supernatant was acidified with 2 M HCl to pH 3-4 and extracted twice with ethyl acetate (2 x 100 mL). Combined organic layers were dried over $MgSO_4$ and solvent was removed under reduced pressure. The organic residue was dissolved in methanol to a concentration of 10 mg/mL, filtered over glass wool and analysed by LCMS. For preparative LCMS cultures were grown in a scale between 1-2 L and the concentration of the organic extract applied for purification was 50 mg/mL

Construction of *A. oryzae* Expression Vectors

T. reesei total RNA was extracted using TRIzol reagent (Thermo Fischer Scientific) and subsequently converted to cDNA using the High-Capacity RNA-to-DNA™ kit (Thermo Fischer Scientific) according to the manufacturer’s instructions. The intron free cDNA was used for amplification of sorbicillinoids biosynthetic genes by PCR (See Table S2 for primers used in this study).
The tailoring genes *sorC, sorD, sdr* and *p450* were cloned into any of the respective multigene expression vectors pTYGSarg/ade/met[1] *via* yeast homologous recombination under control of any of the constitutive promoters p*eno*, p*adh* or p*gdpA* (Table S3). The procedure was as followed: *S. cerevisiae* was streaked out on YPAD agar and incubated at 30 °C for 3-5 days. A single colony was transferred into 10 mL YPAD medium and incubated over night at 30 °C with 200 rpm shaking. This starter culture was added to 40 mL YPAD medium in a 250 mL flask and incubated for another 4.5 h at 30 °C with 200 rpm shaking. The culture was harvested by centrifugation at 3.000 g for 5 min. The pellet was washed with 25 mL ddH_2O and centrifugation was repeated. The pellet was resuspended in 1 ddH_2O, transferred into a 1.5 mL reaction tube and centrifuged at 18.000 g for 15 s. The pellet was resuspended in 400 µL ddH_2O solution and aliquots of 50 µL were transferred into a separate 1.5 mL reaction tube. For each sample one aliquot was centrifuged at 18.000 g for 15 s and the pellet was dissolved in the transformation mixture consisting of 240 µL PEG solution (50 % (*w/v*) polyethylene glycol 3350),36 µL LiOAc (1 M), 50 µL denaturated salmon testis DNA (2 mg/mL in TE buffer), 34 µL DNA master mix containing the linearized vector and desired inserts obtained by PCR in equimolar concentration. The PCR fragments contain each 30 bp overlap at both 5’ and 3’ with the cut sites of the vector fragments to facilitate homologous recombination. Cells were first incubated for 30 min at 30 °C, then for 40 min at 42 °C. Cells were pelleted by centrifugation at 18.000 g for 15 s and supernatant was removed. The pellet was resuspended in 500 µL ddH_2O and 250 µL was spread on selective SM-Ura plates, which were incubated for four days at 30 °C. Constructed plasmid DNA was extracted from yeast cells using a Zymoprep™ Yeast Plasmid Miniprep II kit (Zymo research, Orange, California, USA) and transformed into *E. coli ccdB* Survival cells by standard heat shock method for amplification.
In same manner both PKS *sorA* and *sorB* were assembled in the entry vector pEYA which was subsequently used to transfer the target genes into any of the target multigene expression vectors pTYGSarg/ade/met by Gateway™ cloning.

Construction of *T. reesei* Knockout Vectors

All vectors were built using yeast homologous recombination as described above. For the construction of pEY-TR-*sor*A the gDNA of *T. reesei* QM6a was used as a template for amplifying the right and left fragments for *sor*A , including about 1.5 kb for each fragment. The hygR cassette was amplified from the plasmid pTH-GS-e*GFP*. The primers (Table S2) were designed to include 30 bps overhangs to be recombined in yeast with compatible overhangs in a cut *p*EYA plasmid. For the construction of pEY-TR-s*dr*, about 1 kb were amplified from the left and right ends of the *sdr* gene from the gDNA of *T. reesei* QM6a, together with the hygR cassette fragment to be recombined by yeast homologous recombination. Similarly, about 1 kb length were amplified from the *P450* gene together with a *hygR* cassette to build the KO vector pEYA-TR-*p450*.

Transformation of *A. oryzae* NSAR1

Spore suspension collected from a fresh *A. oryzae* NSAR1 DPY plate (approximately 5 days) was used to inoculate 50 mL (250 mL flask) of GN liquid culture and incubated for 16 h (28 °C, 110 rpm). Cells were collected by filtration over sterile miracloth, washed with 0.8M NaCl and suspended in 10 mL of filter-sterilised *A. oryzae* NSAR1 protoplasting solution (10 mg/mL lysing enzyme from *Trichoderma harzianum*, Sigma-Aldrich, 0.8M NaCl, 10 mM $CaCl_2$). The suspension was incubated for 4 h at ambient temperature with gentle shaking. Protoplasts were released by pipetting, collected by centrifugation (3000 × g, 5 min) and directly suspended in the required amount of fungal transformation solution I (10mM $CaCl_2$, 0.8M NaCl and 50mM Tris-HCl at pH 7.5). Vector DNA (≥1 µg in 10 µL of ddH_2O) was mixed with 100 µL protoplasts and incubated on ice for 5 min. One millilitre of fungal transformation solution II (10mM $CaCl_2$, 0.8M NaCl and 50 mM Tris-HCl at pH 7.5, 60% (*w/v*) PEG3350) was added and the mixture was incubated at ambient temperature for 20 min. Five millilitres of molten selective soft agar (CZD/S, CZD/S1 or CZD/S1 w/o methionine) was added and the mixture was poured over selective agar plates (CZD/S, CZD/S1 or CZD/S1 w/o methionine). Plates were incubated at 28 °C until colonies appeared, which were transferred to secondary plates of the respective selective agar. Vigorously growing colonies were transferred onto a third plate selective plate. For strains constructed in this study see Table S4.

Transformation of *T. reesei* QM6a

Spore suspension collected from a fresh *T. reesei* PD plate was used to inoculate 50 mL (250 mL flask) of GN liquid culture and incubated for 16 h (28 °C, 110 rpm). Cells were collected by filtration over sterile miracloth, washed with washing solution (1.2 M sorbitol, 10 mM Tris-HCl pH 7.5) and suspended in 10 mL of filter-sterilised *T. reesei* protoplasting solution (10 mg/mL lysing enzyme from *Trichoderma harzianum*, 5 mg/mL driselase, 1.2 M sorbitol, 100 mM potassium phosphate pH 5.6). The suspension was incubated for

2 h at 28 °C with gentle shaking. Protoplasts were released by pipetting, collected by centrifugation (3000 × g, 5 min) and directly suspended in the required amount of resuspension solution (1 M sorbitol, 10 mM Tris-HCl pH 7.5).). Vector DNA (≥1 µg, in 10 µL of ddH_2O) was mixed with 200 µL protoplasts and 2 mL of transformation solution III (50mM $CaCl_2$, 10 mM Tris-HCl at pH 7.5, 25% (*w/v*) PEG6000). Samples were incubated on ice for 20 min followed by incubation at ambient temperature for 5 min. 4 mL of resuspension solution were added and aliquots of 200 µL were mixed with 20 ml molten, 50 °C warm PD agar containing 50 µg/mL hygromycin B, spread over cultivation plates and incubated at 28 °C for 3-5 days until colonies were visible. Colonies were picked from the transformation plates and selected for three rounds on PD agar plates supplemented with 100 µg/ml hygromycin B to obtain pure colonies for further analysis. Positive transformants were grown on ME medium flasks at 28 °C for 7 days at 110 rpm for sorbicillinoid production and analysis. For strains constructed in this study see Table S4.

Cloning, Expression and Purification of SorC

For expression of *sorC* in *E. coli* BL21 (DE3) the expression plasmid pET-28/a-*sorC* (encoding for an N-terminal hexa-histidine tag) was built by restriction digest with *Nde*I and *Not*I, followed by ligation using T4 ligase. *T. reesei* cDNA was used as the DNA template (Figure S13). Transformation of competent cells was performed based on a standard heat shock protocol. A pre-culture was grown overnight in LB-medium containing50 µg/ mL kanamycin at 37 °C with 200 rpm shaking. Each 1 mL of this seed culture was used to inoculate 100 mL 2TY-medium containing 50 µg/ mL kanamycin. Cells were grown at 37 °C and 200 rpm until an OD_{600} between 0.4-0.6 was reached. To induce protein expression Isopropyl-β-D-thiogalactopyranoside (IPTG) was added to a final concentration of 0.1 mM and cells were incubated for another 16 h at 16 °C and 200 rpm. Cells were harvested by centrifugation (3500 × g, 20 min) at 4 °C and resuspended in loading buffer (50 mM Tris-HCL pH 8.0, 150 mM NaCl, 10 mM imidazole, 10% glycerol (*v/v*)) and lysed by sonication. Cell debris was removed from the total lysate by centrifugation (10.000 × g, 40 min, 4 °C).
SorC containing a his_6-tag (52.8 kDa) was purified by Ni^{2+} affinity chromatography using a gravity column. 1 mL of Ni-NTA Agarose (Biozym) was washed with loading buffer and added to each 15 mL supernatant obtained in the previous step. The mixture was incubated for 1 h at 8 °C on an agitator with gentle shaking to facilitate protein binding. A 15 mL CHROMABOND® column (Macherey-Nagel) was activated with 20 % ethanol and equilibrated with loading buffer. Ni-NTA with bound protein was collected by gravity and washed twice with each 15 mL loading buffer, followed by another wash step with loading buffer (supplemented with 50 mM imidazole). Protein was eluted in 2 mL fractions with elution buffer (loading buffer + 500 mM imidazole). The buffer was exchanged to 50 mM phosphate buffer pH 8 by ultrafiltration with a molecular weight cut-off of 30 kDa and protein concentration was determined based on extinction coefficient and molecular weight using a spectrophotometer. Purified protein was assessed by SDS-PAGE (Figure S14).

Cloning, Expression and Purification of SorD

First, the synthetic vector pET100D/TOPO-*sorD* was purchased from Thermo Fischer encoding for a codon optimized (Figure S15), intron-free *sorD* (65.0 kDa with N-terminal his_6-tag) for expression in *E. coli* (Figure S16A). Protein expression was performed as described above, varying the expression temperature between 12-37°C and the IPTG concentration between 0.1-1 mM. No overexpressed protein was observed (Figure S16B).
The expression vector plasmid pET-28/a-*sorD* (encoding for an N-terminal hexa-histidine tag) was constructed as described above, using NheI (5') and NotI (3'). Expression temperatures between 12-37°C and IPTG concentrations between 0.1-1 mM were tested, but all resulted in insoluble protein. Autoinduction using TB-medium was also not successful for both plasmids. For a new construct the first 27 amino acids (possible signal sequence) of *sorD* were removed, still resulting in insoluble protein. Usage of *E. coli* Codon Plus cells also met with failure.

For expression of *sorD* in *Saccharomyces cerevisiae* W303B the expression plasmid pESC-*ura*-*sorD* was built by restriction digest/ligation procedure using SalI (5') and KpnI (3'). Successful plasmid construction was confirmed by sequencing. Transformation of yeast was performed as described previously, with the 34 µL transformation mixture consisting only of the already assembled plasmid pESC-ura-*sorD*. Positive colonies were selected for two rounds on SM-ura agar and subsequently grown in glucose-ura medium (50 mL) for 2 days at 30 °C and 160 rpm. Cells were harvested by centrifugation, washed with ddH_2O and subsequently dissolved in 1 mL ddH_20. 200 µL were used to inoculate 50 mL galactose-ura medium to induce protein expression. Cells were incubated for 16 h at 30 °C and 160 rpm. After washing with ddH_2O pelleted cells were dissolved in 1 mL PBS buffer (100 mM phosphate buffer pH 7.5, 8g/L NaCl) and lysed mechanically. The supernatant obtained after centrifugation was directly used for enzyme assays (assays were performed by adding 50 µL of the supernatant to the "standard" SorC assay) and was also analysed by SDS-PAGE. No putative target protein could be seen and the assays did not differ from any control.

Enzyme Assays with SorC

<u>Enzymatic formation of **2a/2b**:</u> All analytical assays were performed on a 200 µL scale consisting of SorC (1 mg/mL) dissolved in 50 mM potassium phosphate buffer (pH 8), 10 mM NAD(P)H and 8 mM substrate **1a/1b** (dissolved in acetone). Final concentration of acetone in the assay mixture was 5% (*v/v*), but the same results were obtained with 20 % (*v/v*). Assays were incubated at ambient temperature for one hour with gentle shaking. 200 mL MeCN was added and precipitated enzyme was separated by centrifugation. Supernatant was directly subjected to LCMS analysis.

<u>Enzymatic formation **10a/10b** and **15a**:</u> Assays were performed as described above, but modified slightly by adding an excess of NAD(P)H (20 mM) and raising the incubation time to 16-24 hours.

Enzymatic formation of bisorbicillinols **3a-c**: All analytical assays were performed on a 200 µL scale consisting of SorC (1 mg/mL) dissolved in 50 mM potassium phosphate buffer (pH 8), 10 mM NAD(P)H and 8 mM substrate **1a/1b** (dissolved in acetone). Final concentration of acetone in the assay mixture was 20 % (*v/v*). Assays were extracted with 400 µL CH_2Cl_2 or $CHCl_3$ and the organic phase was removed by vacuum-centrifugation at 45 °C. Organic residue was dissolved in 100 µL MeOH and subjected to LCMS analysis. Formation of bisorbicillinols was also observed when the volume of acetone in the assays mixture was kept at 5% (*v/v*), indicating that the organic solvent inducing the dimerization is CH_2Cl_2/ $CHCl_3$.

Enzymatic formation of spirosorbicillinols **6a/6b**: Assays were performed as described for the formation of bisorbicillinols **3a-c**, but adding an excess of scytolide **12** (20 mM) to the assay mixture.

Feeding Experiments

Cultures of the respective fungal transformants or the wt strain were inoculated into liquid cultures (50 mL DPY medium in 250 mL flask) and grown for two days at 28 °C with 110 rpm shaking. 2 mg of the respective compound (**1a** or **12**) dissolved in DMSO were added and cultures were grown for another 16 h. Chemical extraction was performed as mentioned above.

Synthesis of 15a*

6 mg of **15a** were dissolved in 2 mL MeOH and a slight excess of TMS-diazomethane (2 M in diethyl ether) were added dropwise while stirring. The sample was incubated for 2 h, the solvent was evaporated and the sample was directly analysed by NMR.

Biosynthetic Gene Cluster Analysis

Cluster identification: Draft genome of *T. reesei* QM6a was obtained from NCBI (WGS: AAIL02) and putative gene clusters were predicted using the secondary metabolites analysis tool fungiSMASH.[2] Among the 32 predicted gene clusters, the first shared 71 % homology with the sorbicillinoid biosynthetic gene cluster of *P. chrysogenum*. The respective cluster sequence was used as the query sequence for gene identification/ protein prediction with FGENESH.[3] Subsequently conserved domain analysis was applied using BLASTp. In same manner the sorbicillinoid biosynthetic gene cluster was identified in the draft genome of *P. chrysogenum* (WGS: JMSF01).

Selection of candidate genes: Bioinformatic analysis of the sorbicillinoid biosynthetic gene cluster in *T. reesei* QM6a revealed three candidate genes that, based on their predicted function, could be involved in sorbicillinoid biosynthesis: A second FMO (*sorD*), a short-chain dehydrogenase/ reductase (*sdr*) and a cytochrome P450 dependent monooxygenase (*p450*). Analysis of *T. reesei* cDNA showed that *sorD* and *p450* were expressed under sorbicillinoid producing conditions, although the expression of *p450* was weak. Although the SorD proteins from *T. reesei* (XP_006961562) and *P. chrysogenum* (XP_002567557) possess the same conserved domains, both proteins share only 18.3 % identity and 30.2 % similarity based on protein alignment using the software geneious® (standard parameters).

ARTEMIS analysis: The alignment tool tBLASTx was used to create a comparison file between the sorbicillinoid BGC of *T. reesei* and *P. chrysogenum*. This file was used for identification of homologous proteins between the two cluster using ARTEMIS.[4] Results show that the following proteins are homologous: SorA, SorB, SorC, one MFS and one TF, but not SorD.

Additional Results

Confirming Expression of *sorD* in +*sorABD* Transformants

In order to confirm that *sorD* was truly expressed in +*sorABD* transformants their total RNA was extracted and converted to cDNA as described previously. Expression of *sorD* was confirmed by PCR (Figure S17). The expression plasmid pTYGSade-*sorA* was used as a positive control and the freshly isolated RNA was used as a negative control to exclude contamination with genomic DNA.

Comparison of Sorbicillinoids Produced by +*sorABCD* Transformants Prior to Chemical Extraction

Transformants were grown for four days in liquid DPY-medium at 28°C with 110 rpm shaking. Cultures were homogenized using a hand blender and cells were separated by filtration. Crude supernatant was directly subjected to LCMS-analysis. LCMS-analysis revealed presence of dimeric sorbicillinoids already prior to chemical extraction (Figure S10).

I*n Vitro* Formation of 15a and 10ab

Sorbicillinol **2a** was produced enzymatically by SorC using **1a** as the substrate and extracted with ethyl acetate. Formation of **2a** was confirmed by LCMS prior to incubation . **2a** was incubated for 20 h in 50 mM phosphate (pH 8) buffer at ambient temperature either in presence or absence of NADPH. Upon longer incubation (16-24 h) epoxysorbicillinols **10** and the reduced sorbicillinol **15a** are formed from sorbicillinol **2** during *in vitro* assay with SorC when an excess of NADPH is present (Figure S12).

Tables

Table S1: Media used during this study.

Media	Components
2TY medium	16 g/L tryptone, 10 g/L yeast extract, 5 g/L NaCl
CZD/S agar	35 g/L Czapek Dox broth, 182.2 g/L D-sorbitol,<br>1 g/L ammonium sulphate, 0.5 g/L adenine, 1.5 g/L-methionine, 15 g/L agar
CZD/S soft agar	35 g/L Czapek Dox broth, 182.2 g/L D-sorbitol,<br>1 g/L ammonium sulphate, 0.5 g/L adenine, 1.5 g/L-methionine, 8 g/L agar
CZD/S1 agar	35 g/L Czapek Dox broth, 182.2 g/L D-sorbitol,<br>1 g/L ammonium sulphate, 1.5 g/L-methionine, 15 g/L agar
CZD/S1 soft agar	35 g/L Czapek Dox broth, 182.2 g/L D-sorbitol,<br>1 g/L ammonium sulphate, 1.5 g/L-methionine, 8 g/L agar
CZD/S1 agar w/o methionine	35 g/L Czapek Dox broth, 182.2 g/L D-sorbitol,<br>1 g/L ammonium sulphate, 15 g/L agar
CZD/S1 soft agar w/o methionine	35 g/L Czapek Dox broth, 182.2 g/L D-sorbitol,<br>1 g/L ammonium sulphate, 8 g/L agar
DPY medium	20 g/L dextrine from potato starch, 10 g/L polypeptone, 5 g/L KH_2PO_4, 0.5 g/L $MgSO_4 \cdot H_2O$
Galactose-ura medium	6.7g/L yeast nitrogen base, 20g/L galactose monohydrate, 1.3g/L complete supplement mixture minus uracil
Glucose-ura medium	6.7g/L yeast nitrogen base, 20g/L glucose monohydrate, 1.3g/L complete supplement mixture minus uracil
GN medium	20 g/L D (+)-glucose monohydrate, 10 g/L nutrient broth No. 2 from oxoid (Thermo Scientific)
LB agar	5 g/L yeast extract, 10 g/L tryptone, 5 g/L NaCl, 15 g/L agar
LB medium	5 g/L yeast extract, 10 g/L tryptone, 5 g/L NaCl
ME medium (Roth x923)	12.75 g/L malt extract, 0.78 g/L peptone from soy bean, 2.35 g/L glycerine, 2.75 g/L dextrin from potato starch
PD agar	24 g/L potato dextrose broth, 15 g/L agar
PDB medium	24 g/L potato dextrose broth
SM-ura agar	1,7 g/L yeast nitrogen base, 20 g/L D (+)-glucose monohydrate, 5 g/L ammonium sulphate,<br>0.77 g/L complete supplement mixture minus uracil (Q biogene), 25 g/L agar
SOB medium	2 g/L tryptone, 0.5 g/L yeast extract, 58.4 mg/L NaCl, 18.6 mg/L KCl
SOC medium	937.5 mL/L SOB medium, 12.5 mL/L $MgCl_2$, 50 mL/L glucose (20 %)<br>components were autoclaved separately and sterile filtrated after mixing
TB-medium	24 g/L yeast extract, 12 g/L tryptone, 4 mL/L glycerine,
YPAD agar	10 g/L yeast extract, 10 g/L tryptone, 0.3 g/L adenine, 20 g/L D (+)-glucose monohydrate,<br>15 g/L agar
YPAD medium	10 g/L yeast extract, 10 g/L tryptone, 0.3 g/L adenine, 20 g/L D (+)-glucose monohydrate

Table S2: Primers used during this study.

Primer name	Sequence (5’ to 3’)
SorA P1	GCCAACTTTGTACAAAAAAGCAGGCTCCGCATGAAGCTGACGGCCCTCAA
SorA P3	TTGCAAATGTCTACGTCGAGG
SorA P6	CGAGATTATACAGGATAGGCTC
SorA P7	TCGAAGACTCATTGCGGCTGTT
SorA P9	CCTGTACTCATGGCCTTAATGC
SorA P10	ATCTGCATCTTTATCGGGGAAAT
SorB P1	GCCAACTTTGTACAAAAAAGCAGGCTCCGCATGGCGGCCTCAAGTACACG
SorB P2	ATTAACACTAAACGCCAGGTCTTGGATATCGAGATTGCTGTGAGAGTAAA
SorB P3	GCAATCTCGATATCCAAGACC
SorB P4	GTCACCAAGCGTAGACTGTAG
SorB P5	CCTTAAGTTCCTACAGTCTACGCTTGGTGACGAGGACGTTCACGACTCTT
SorB P6	TGCCAACTTTGTACAAGAAAGCTGGGTCGGTCATCGCAAAAAGCCGCTAT
SorC F	TTCTTTCAACACAAGATCCCAAAGTCAAAGATGGAGCCGAACAATCATCAC
SorC R	TTTCATTCTATGCGTTATGAACATGTTCCCTCTAATGCTTCTCTAACACCT
SorC for ligation F	GGGAATTCCATATGGAGCCGAACAATCATCA
SorC for ligation R	TTCCTTTTTTGCGGCCGCCTAATGCTTCTCTAACACCT
SorD F	CGACTGACCAATTCCGCAGCTCGTCAAAGGATGTACGCCCCCCCTTTTGT
SorD R	AGGTTGGCTGGTAGACGTCATATAATCATATTACGAGACTCGGCAAAGGC
SorD-his F	TTCTTTCAACACAAGATCCCAAAGTCAAAGATGGGCAGCAGCCATCATCA
SorD-his R	TTCATTCTATGCGTTATGAACATGTTCCCTTTACGAGACTCGGCAAAGGC
SorD yeast F	ACGCGTCGACATGGCTTCTCTGAAATCGTCCGG
SorD yeast R	CGGGGTACCTTACGAGACTCGGCAAAGGC
SorD ligation F	CTAGCTAGCGCAAGCCTGAAAAGCAGCGG
SorD ligation R	TTCCTTTTTTGCGGCCGCTTAGCTAACACGACACAGAC
SorD ligation F2	CTAGCTAGCTACGCCCCCCCTTTTGTCCG
SorD ligation F3	CTAGCTAGCGCTTCTCTGAAATCGTCCGG
SorD ligation R2	TTCCTTTTTTGCGGCCGCTTACGAGACTCGGCAAAGGC
SorP450 F	TTCTTTCAACACAAGATCCCAAAGTCAAAGATGTCAAACCTGGCCTTGGC
SorP450 R	TTTCATTCTATGCGTTATGAACATGTTCCCTCTATTTTCTCAATTTGTCAA
SorP450 F2	TCGTCGTGAACCAGATGAGCAA
SorP450 R4	TCGGTAGGCAGTAATCAGCTTG
Sor-SDR F	ACAGCTACCCCGCTTGAGCAGACATCACCGATGTCCTCTCCAGCCATAGG
Sor-SDR R	ACGACAATGTCCATATCATCAATCATGACCTTACGGGTCAACCTGATTAT
Sor-SDR F2	GCAAAAGTGGTCATGAGAGGAGCATGCAAGTCAATTGCTACACTCACCTT
Sor-SDR R2	GGGAGATGAGAAGGTGAGTGTAGCAATTGACTTGCATGCTCCTCTCATGA
TR-SorA-KO-P1F	ATGCCAACTTTGTACAAAAAAGCAGGCTCCCAACTTGTCGAGTATCTCCG
TR-SorA-KO-P2R	CGAAAGATCCACTAGAGGATCCCCATCATGTTGCAAGCCAGATCAAGGGC
TR-SorA-KO-P3F	CCCAGCACTCGTCCGAGGGCAAAGGAATAGGCCAAGACAAGTACCGCTTC
TR-SorA-KO-P4R	AATGCCAACTTTGTACAAGAAAGCTGGGTCGCGAGTGAACATTTCCATCC
HygR-KO-P1F	CATGATGGGGATCCTCTAGTG
HygR-KO-P2R	CTATTCCTTTGCCCTCGGAC
TR-SDR-FL-P1F	GCCAACTTTGTACAAAAAAGCAGGCTCCGCCCATATTATATATAGGTATG
TR-SDR-FL-P2R	ACGTATTTCAGTGTCGAAAGATCCACTAGACTTCTATCTCAAGCTGCCCA
TR-SDR-FR-P3F	GTCCGAGGGCAAAGGAATAGGGACAAGCGTTATTAACTATAACTTCGGAT
TR-SDR-FR-P4R	TGCCAACTTTGTACAAGAAAGCTGGGTCGGTAACTTCACAGCAGACAGAC
TR-P450-KO-P1F	ATGCCAACTTTGTACAAAAAAGCAGGCTCCGATGAAGGCTTCTTTTTTTT
TR-P450-KO-P2R	CGAAAGATCCACTAGAGGATCCCCATCATGCAAAGGCAGTCACATGTACT
TR-P450-KO-P3F	CCCAGCACTCGTCCGAGGGCAAAGGAATAGGGAACAAATTATCAGGGTCC
TR-P450-KO-P4R	AATGCCAACTTTGTACAAGAAAGCTGGGTCCTTCTCGATGCACAACAGAG
SorA-KOPo1-P1F	CTCTTGCCCTGTGATGGTT
SorA-KOPo1-P2R	CTGGCCCTTATCGTACTCCA
SorA-KOPo2-P1F	GGCAATTTCGATGATGCAGC
SorA-KOPo2-P2R	CAGATCTCACTCGTTCGCCT

SorA-KOPo3-P1F	GAAATTGCGATCTGACAAGT
SorA-KOPo3-P2RF	CACTCTCGACTCGTTCATCA
Sdr-KOPo1-P1F	GGTGTCATGATCAAATTTGA
Sdr-KOPo1-P2R	CTGGCCCTTATCGTACTCCA
Sdr-KOPo2-P1F	GGCAATTTCGATGATGCAGC
Sdr-KOPo2-P2R	TGTGTGAAATAACTTCACAG
Sdr-KOPo3-P1F	GGTTCTTGTCTATGCCAGTT
Sdr-KOPo3-P2R	CTGAGCAAACAGCCTATATC
P450-KOPo1-P1F	CACGGAGGGATGAAAGGACT
P450-KOPo1-P2R	CTGGCCCTTATCGTACTCCA
P450-KOPo2-P1F	GGCAATTTCGATGATGCAGC
P450-KOPo2-P2R	CAACCTCAGCTTCAGTCCGC
P450-KOPo3-P1F	CTCTGAGAAGTTGGTCATGA
P450-KOPo3-P2R	CCATAGTTCTCCTTCATGAT

Table S3: Plasmids used in this study.

Plasmid	Features
pESC-ura (AgilentTechnologies)	*URA3 PGAL1 PGAL10 amp^R 2µori ColE1*
pESC-ura-*sorD*	*sorD* inserted into MCS2
pET100/D-TOPO (Invitrogen)	*kmp^R pBR322 rop LacI pT7 His-Tag*
pET100/D-TOPO-*sorD*	encodes for *sorD* with codon-optimized sequence for expression in *E.coli*
pET28a (Novagen)	*kan^R pBR322 ori LacI f1 ori pT7 His-Tag*
pET28a-*sorC*	encodes for *sorC* with N-terminal his$_6$-tag; cloned from *T. reesei* cDNA
pET28a-*sorD*	encodes for *sorD* with N-terminal his$_6$-tag; cloned from *T. reesei* cDNA
pE-YA[1]	*kan^R pUC ori 2µ ori URA3 ccdB*
pE-YA-*sorA*	*sorA* inserted by LR-recombination
pE-YA-*sorB*	*sorB* inserted by LR-recombination
pE-Y-TR-*sorA*	*hyg^R* flanked by *sorA* fragments
pE-Y-TR-*sor"p450"*	*hyg^R* flanked by *sor"p450"* fragments
pE-Y-TR-*sor"sdr"*	*hyg^R* flanked by *sor"sdr"* fragments
pTYGSarg/ade/met[1]	*PamyB Padh Peno PgdpA amp^R ColE1 2µ ori URA3 ccdB argB/adeA/sC*
pTYGSade-*sorA*	*sorA* under control of *PamyB*
pTYGSade-*sorD*	*sorD* under control of *Padh*
pTYGSade-*sorAD*	*sorA* under control of *PamyB, sorD* under control of *Padh*
pTYGSarg-*sorB*	*sorB* under control of *PamyB*
pTYGSarg-*sorC*	*sorC* under control of *Padh*
pTYGSarg-*sorBC*	*sorB* under control of *PamyB, sorC* under control of *Padh*
pTYGSmet-*sor"p450"*	*Sor"p450"* under control of *Padh*
pTYGSmet-*sor"sdr"*	*Sor"sdr"* under control of *PgdpA*

Table S4: Fungal strains used during this study.

Fungal strain	genotype
A. oryzae NSAR1	*argB*$^{-}$, *adeA*$^{-}$, *sC*$^{-}$, *niaD*$^{-}$
T. reesei QM6a	wild type
A. oryzae-sorA	*+sorA, sC*$^{-}$, *niaD*$^{-}$
A. oryzae-sorB	*+sorB, sC*$^{-}$, *niaD*$^{-}$
A. oryzae-sorAB	*+sorA,+sorB, sC*$^{-}$, *niaD*$^{-}$
A. oryzae-sorABC	*+sorA,+sorB, +sorC, sC*$^{-}$, *niaD*$^{-}$
A. oryzae-sorABCD	*+sorA, +sorB, +sorC, +sorD, sC*$^{-}$, *niaD*$^{-}$
A. oryzae-sorABD	*+sorA, +sorB, +sorD, sC*$^{-}$, *niaD*$^{-}$
A. oryzae-sorC	*+sorC, sC*$^{-}$, *niaD*$^{-}$
A. oryzae-sorD	*+sorD, sC*$^{-}$, *niaD*$^{-}$
A. oryzae-sorCD	*+sorC, +sorD, sC*$^{-}$, *niaD*$^{-}$
A. oryzae-sorABCDp450	*+sorA, +sorB, +sorC, +sorD, +sor"p450" , niaD*$^{-}$
A. oryzae-sorABCDsdr	*+sorA, +sorB, +sorC, +sorD, +sor"sdr", niaD*$^{-}$
T. reesei QM6a ∆tmus53[5]	deletion strain of human LIG4 homolog
T. reesei QM6a ∆tmus53∆sorA	∆tmus53, ∆*sorA, hygR*
T. reesei QM6a ∆tmus53∆sor"p450"	∆tmus53, ∆*sor"p450", hyg*^{R}
T. reesei QM6a ∆tmus53∆sor"sdr"	∆tmus53, ∆*sor"sdr", hyg*^{R}

Figures

Sorbicillinoid Compounds Identified by Mass and UV Absorption Profiles

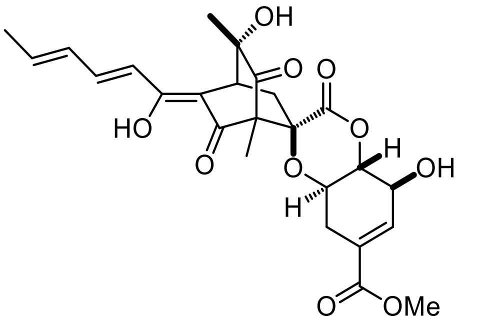

spirosorbicillinol A **6a**

Chemical Formula: $C_{25}H_{28}O_{10}$

Exact Mass: 488,1682

UV$_{\lambda max}$ (MeOH): 216 nm, 250 nm, 361 nm

UV-absorption, mass (identical) and fragmentation pattern (very similar) are matching spirosorbicillinol B **6b** whose structure is fully characterized by NMR (see section NMR).

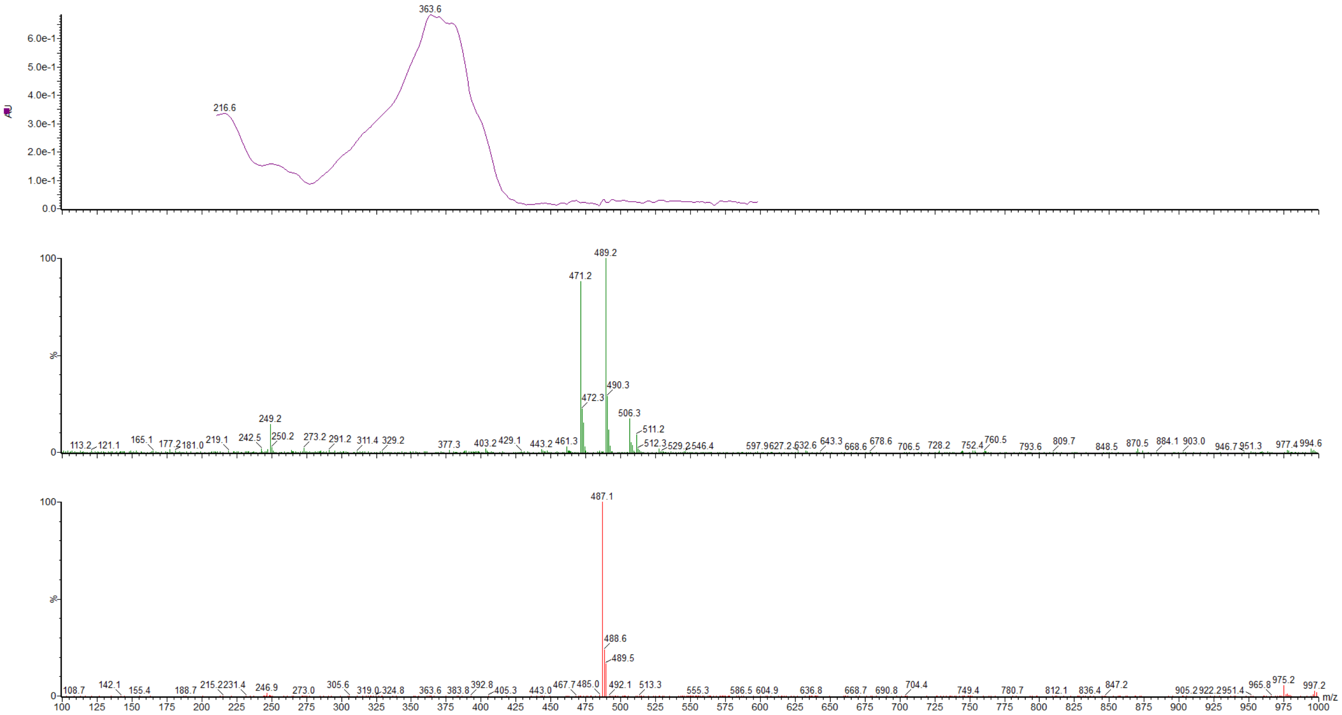

Figure S1: UV-absorption (top) and fragmentation pattern of **6a** in ES$^+$ TIC (middle) and ES$^-$ TIC (bottom).

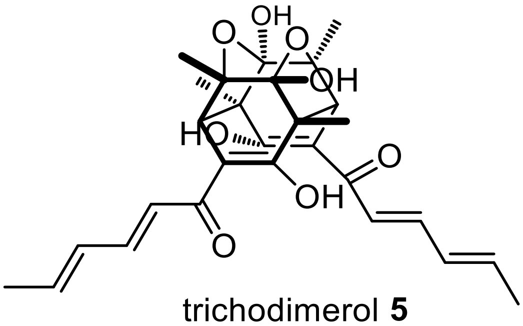

trichodimerol **5**

Chemical Formula: $C_{28}H_{32}O_8$
Exact Mass: 496,2097

UV$_{\lambda max}$ (MeOH): 230nm, 295 nm, 310 nm, 363 nm
Compound was identified based on mass, retention time and UV-absorption[6]

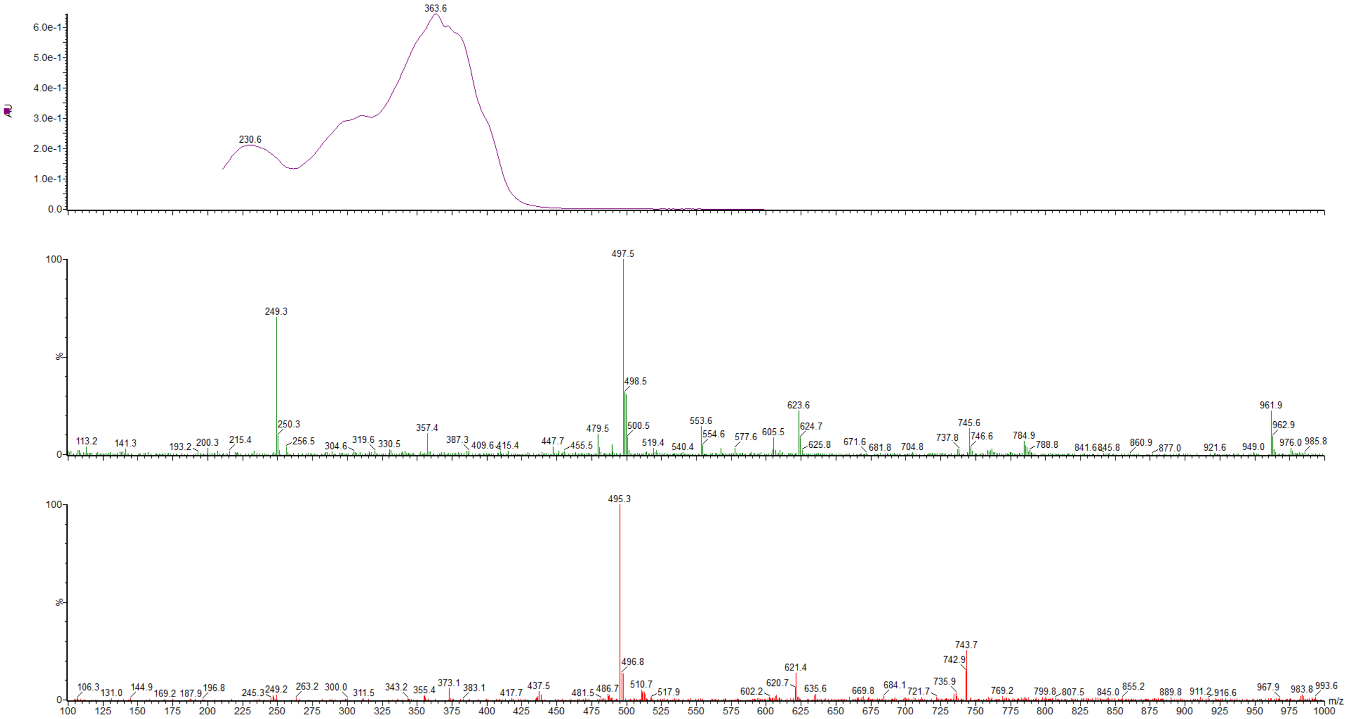

Figure S2: UV-absorption (top) and fragmentation pattern of **5** in ES$^+$ TIC (middle) and ES$^-$ TIC (bottom).

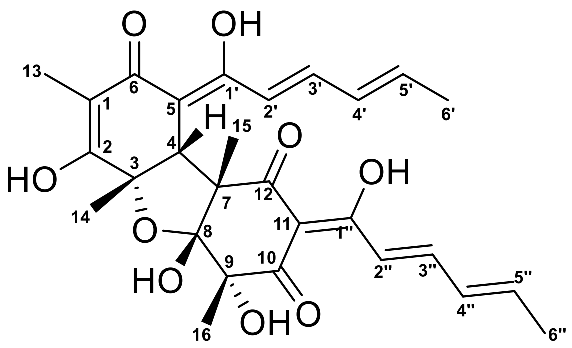

bisvertinolone **11**

Chemical Formula: $C_{28}H_{32}O_9$
Exact Mass: 512,2046

UV$_{\lambda max}$ (MeOH): 226 nm, 271 nm, 305 nm, 370 nm
Compound was identified based on mass, retention time and UV-absorption[7]

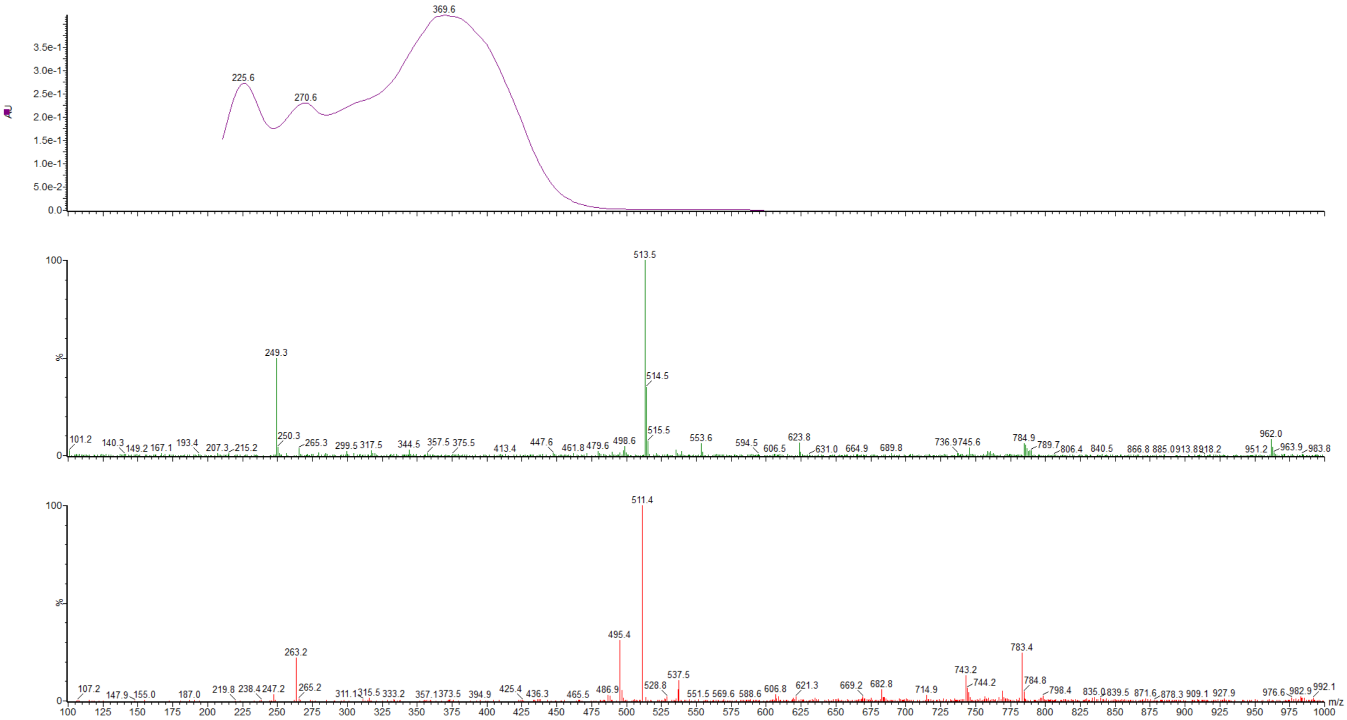

Figure S3: UV-absorption (top) and fragmentation pattern of **11** in ES$^+$ TIC (middle) and ES$^-$ TIC (bottom).

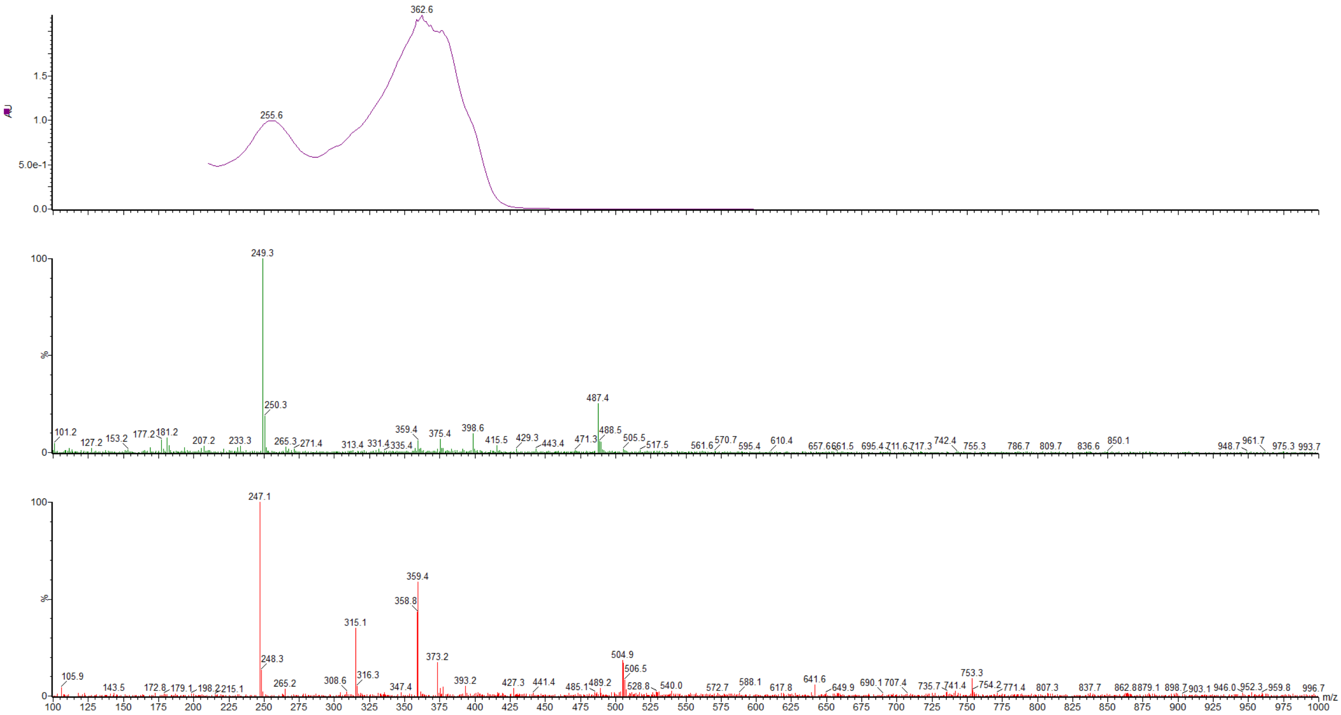

Figure S4: UV-absorption (top) and fragmentation pattern in ES+ TIC (middle) and ES- TIC (bottom) for a putative sorbicillinoid related compound in Fig. 1A marked with * eluting at approx. 6.2 min.

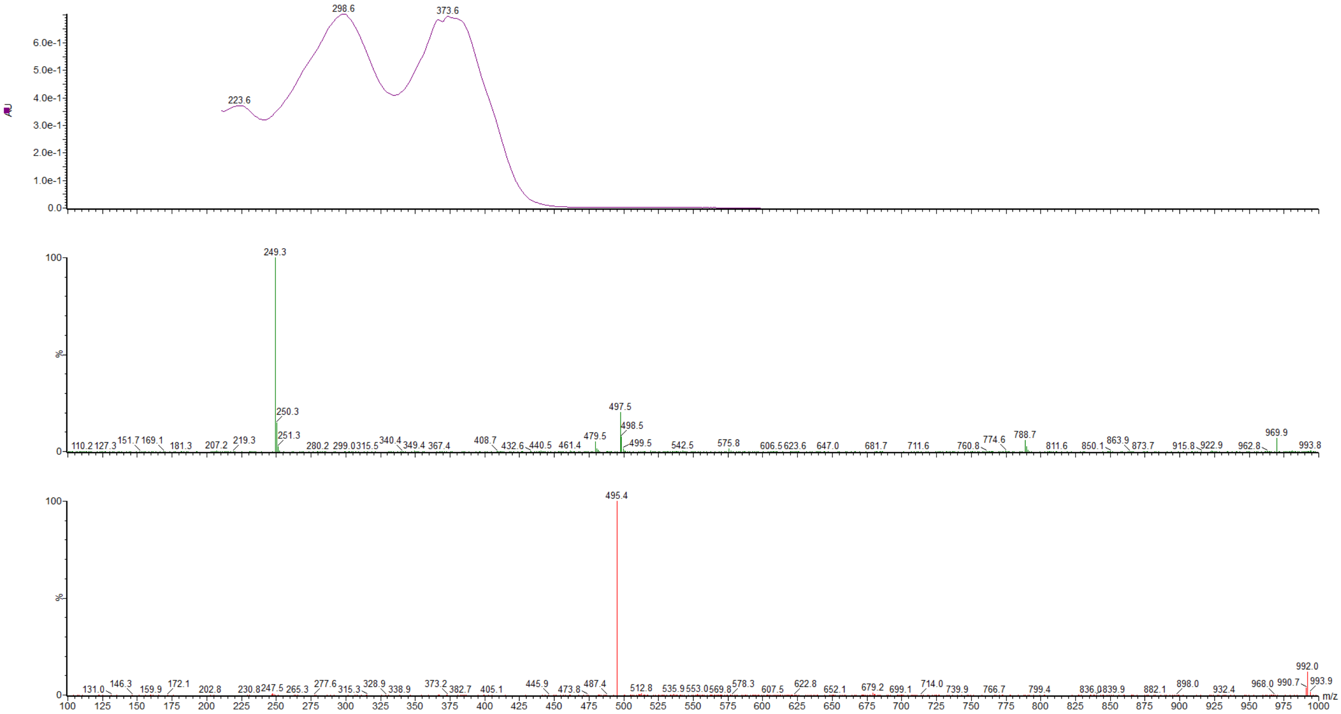

Figure S5: UV-absorption (top) and fragmentation pattern in ES+ TIC (middle) and ES- TIC (bottom) for **a** putative sorbicillinoid related compound in Fig. 1A marked with * eluting at approx. 7.8 min.

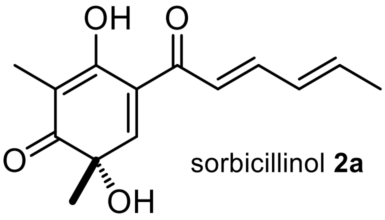

sorbicillinol **2a**

Chemical Formula: $C_{14}H_{16}O_4$
Exact Mass: 248,1049

UV$_{\lambda max}$ (MeOH): 292 nm
Mass data and UV-absorption are in agreement with Cox *et al*.[8]

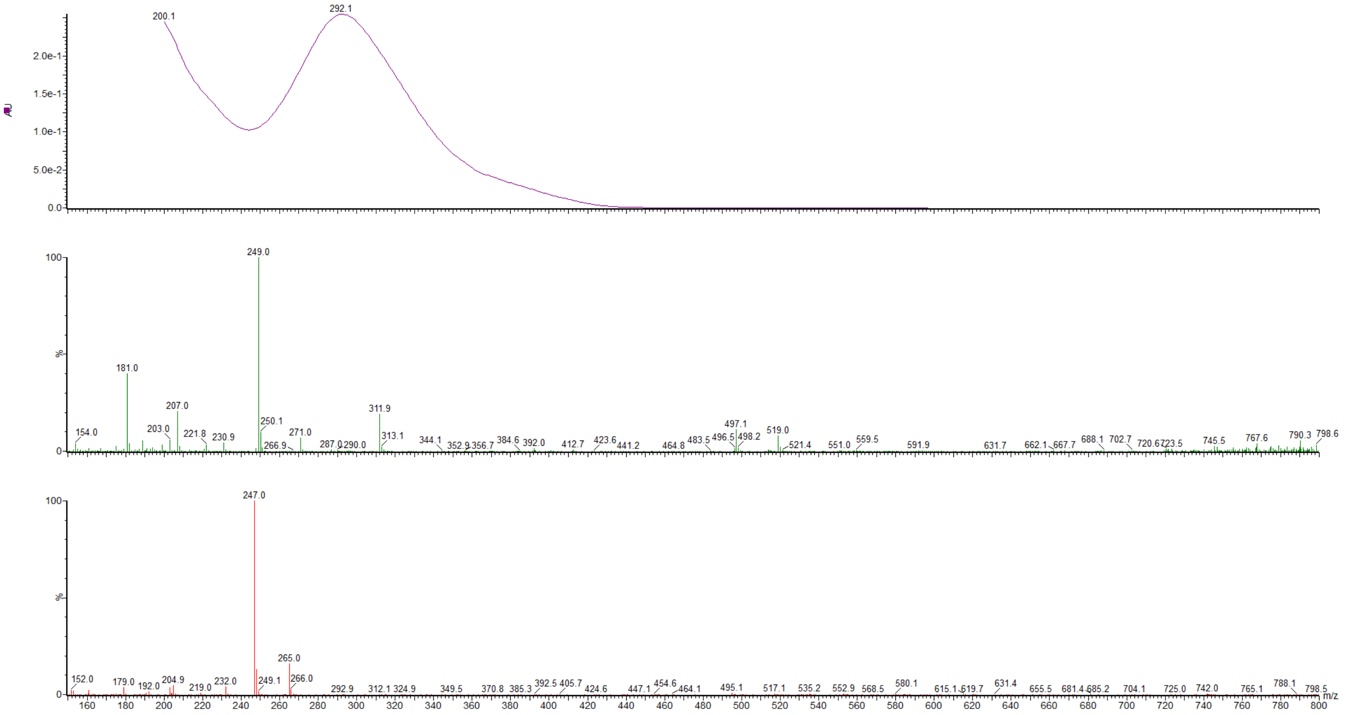

Figure S6: UV-absorption (top) and fragmentation pattern of **2a** in ES+ TIC (middle) and ES- TIC (bottom).

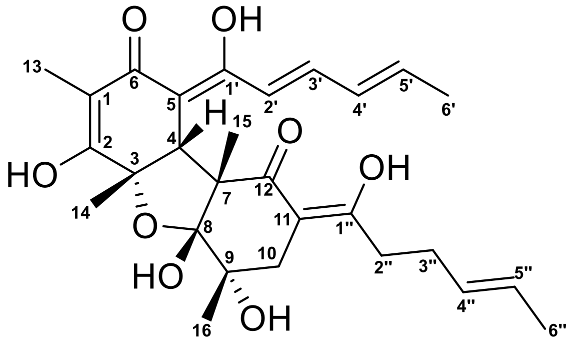

2'',3''-dihydrobisvertinol **4b**

Chemical Formula: $C_{28}H_{36}O_8$
Exact Mass: 500,2410

UV$_{\lambda max}$ (MeOH): 224nm, 269 nm, 306 nm, 379 nm
Compound was identified based on mass, retention time and UV-absorption[7]

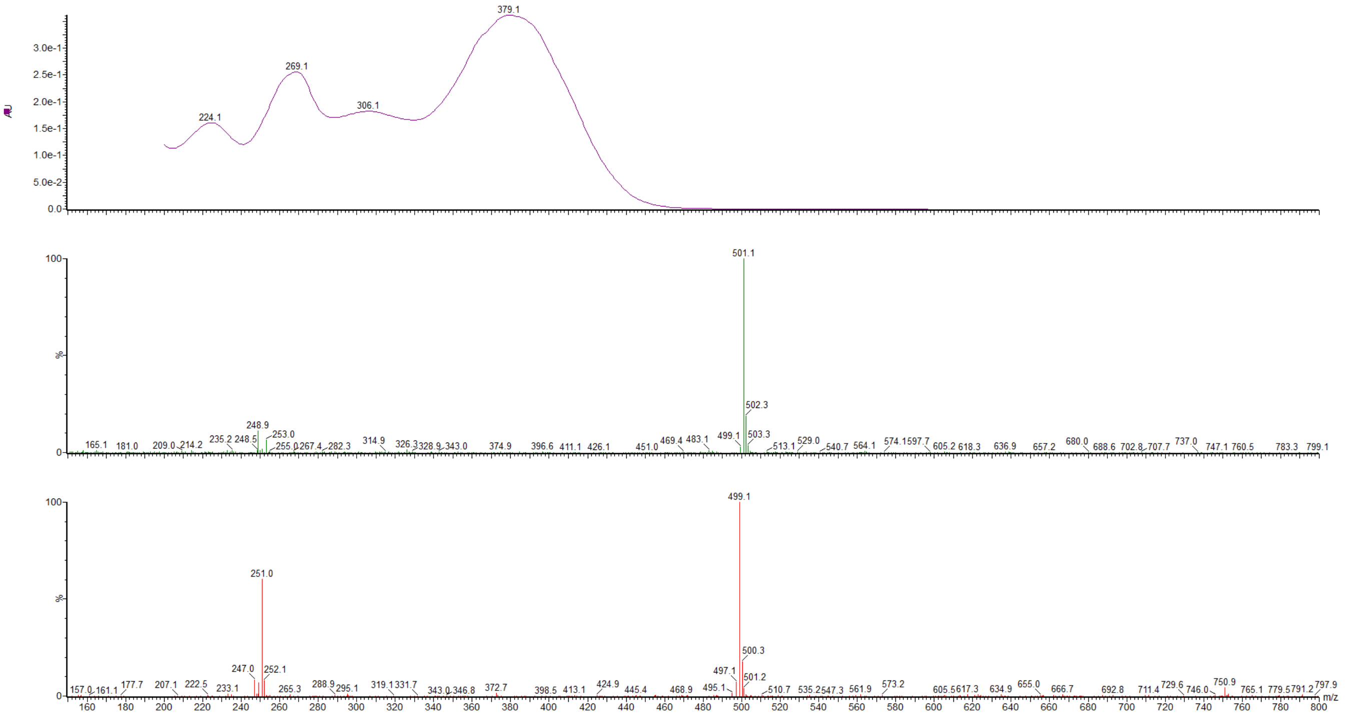

Figure S7: UV-absorption (top) fragmentation pattern of **4b** in ES+ TIC (middle) and ES- TIC (bottom).

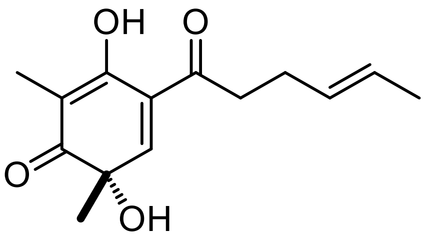

2', 3'-dihydrosorbicillinol **2b**

Chemical Formula: $C_{14}H_{18}O_4$
Exact Mass: 250,1205

UV$_{\lambda max}$ (MeOH): 220 nm, 272 nm, 315 nm
Mass data and UV-absorption are in agreement with Cox *et al*.[8]

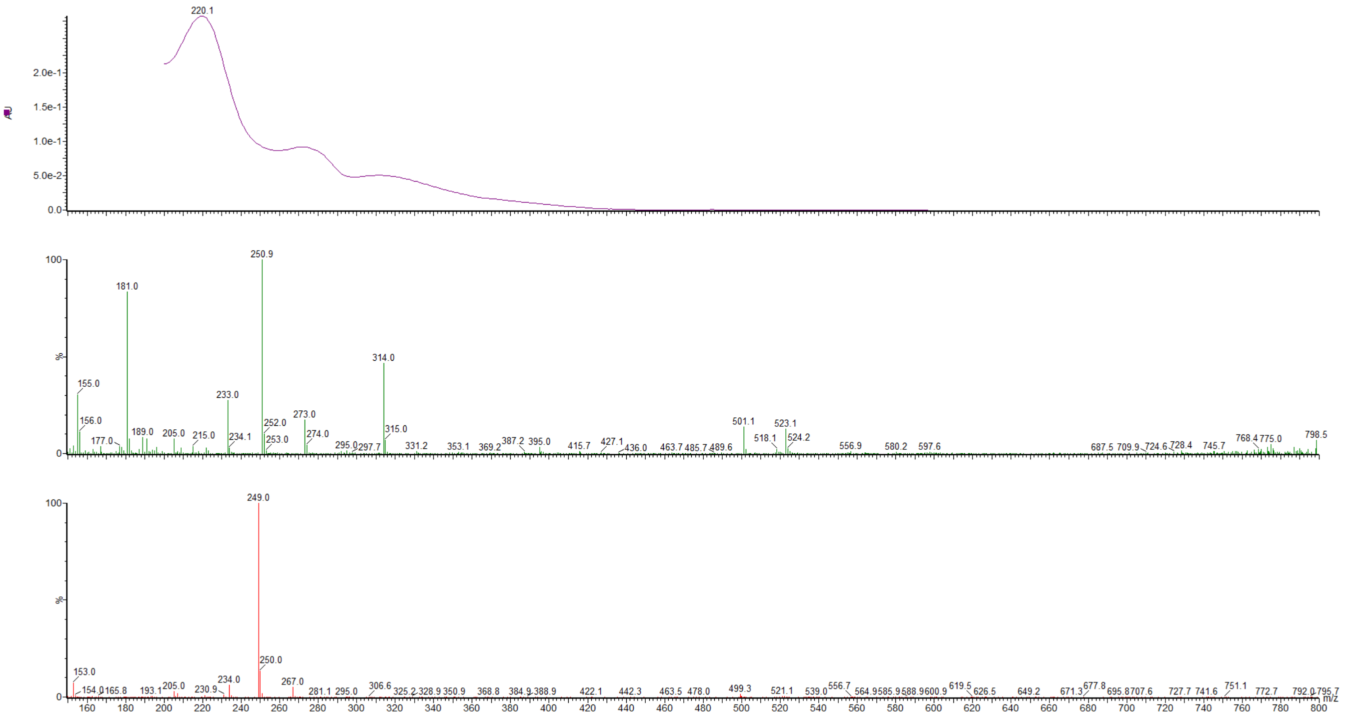

Figure S8: UV-absorption (top) and fragmentation pattern of **2b** in ES+ TIC (middle) and ES- TIC (bottom).

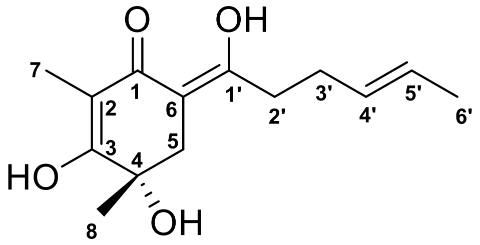

reduced 2', 3'-dihydrosorbicillinol **15b**

Chemical Formula: $C_{14}H_{20}O_4$
Exact Mass: 252,1362

UV$_{\lambda max}$ (MeOH): 218nm, 265 nm, 330 nm

Compound was identified based on mass, retention time and biosynthetic considerations.

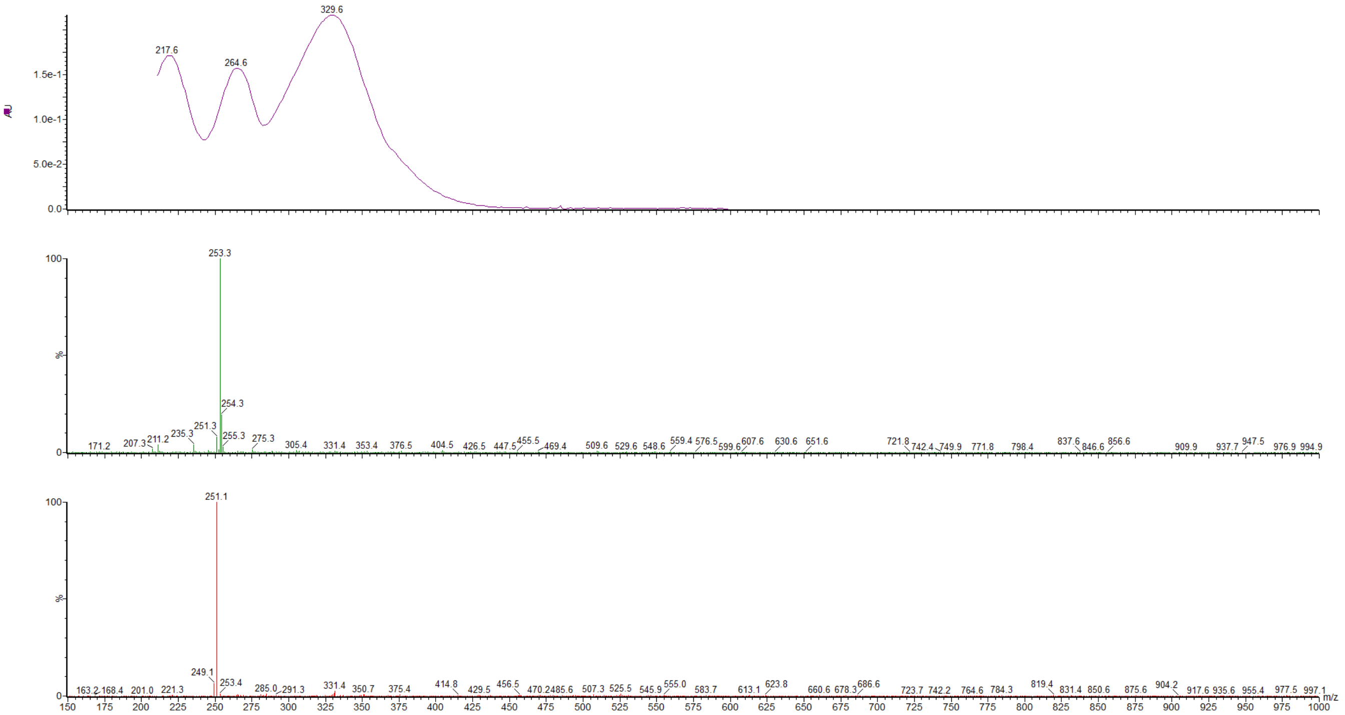

Figure S9: UV-absorption (top) and fragmentation pattern of **15b** in ES$^+$ TIC (middle) and ES$^-$ TIC (bottom).

LCMS Chromatograms

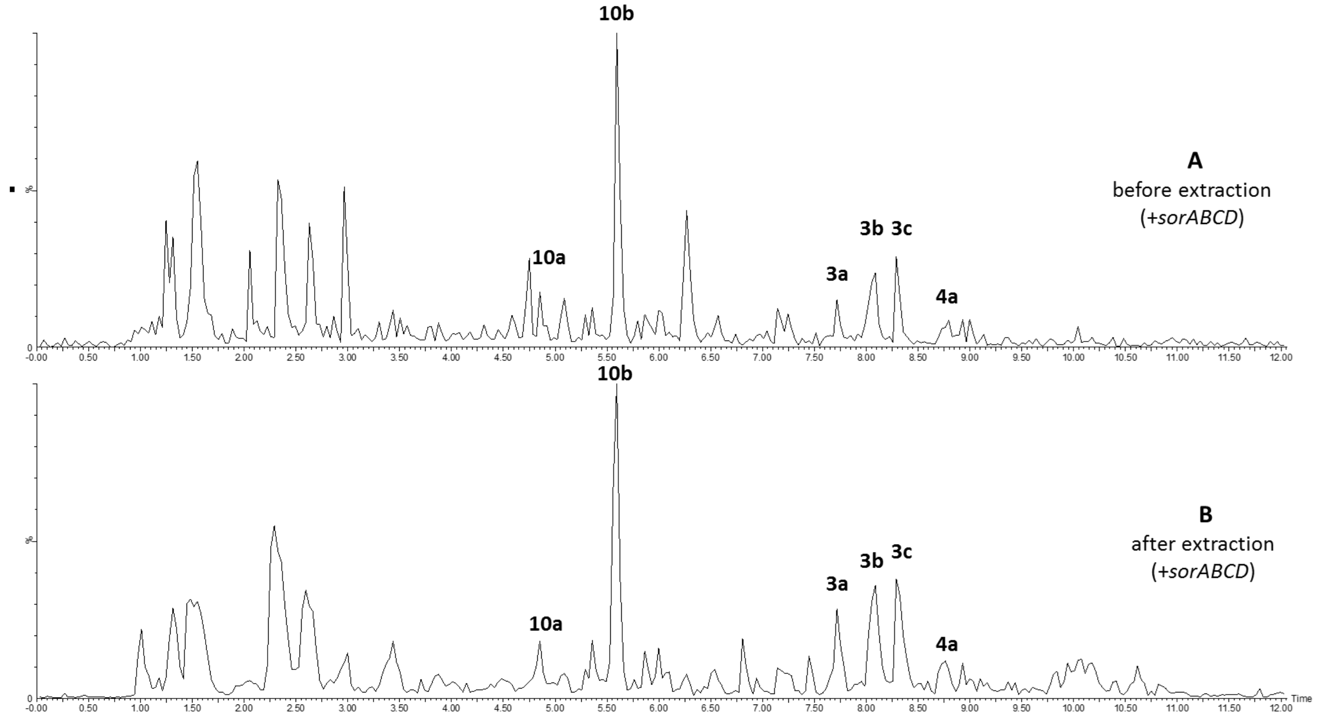

Figure S10: LCMS-chromatograms (ES⁻ TIC) of transformants expressing *sorABCD* before (**A**) or after (**B**) extraction. Dimeric sorbicillinoids are already formed prior to extraction with solvents.

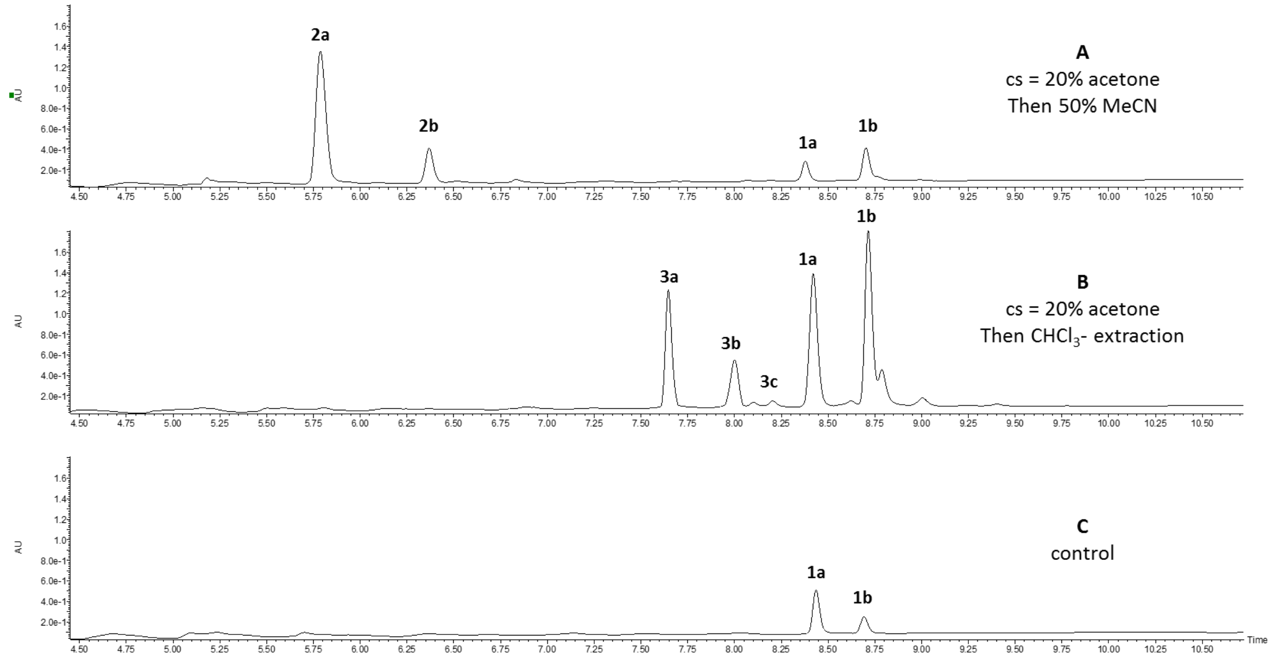

Figure S11. LCMS analysis [DAD 210-600nm] of *in vitro* assays with SorC under different conditions. The control assay contained boiled enzyme; cs = co-solvent (*v/v*).

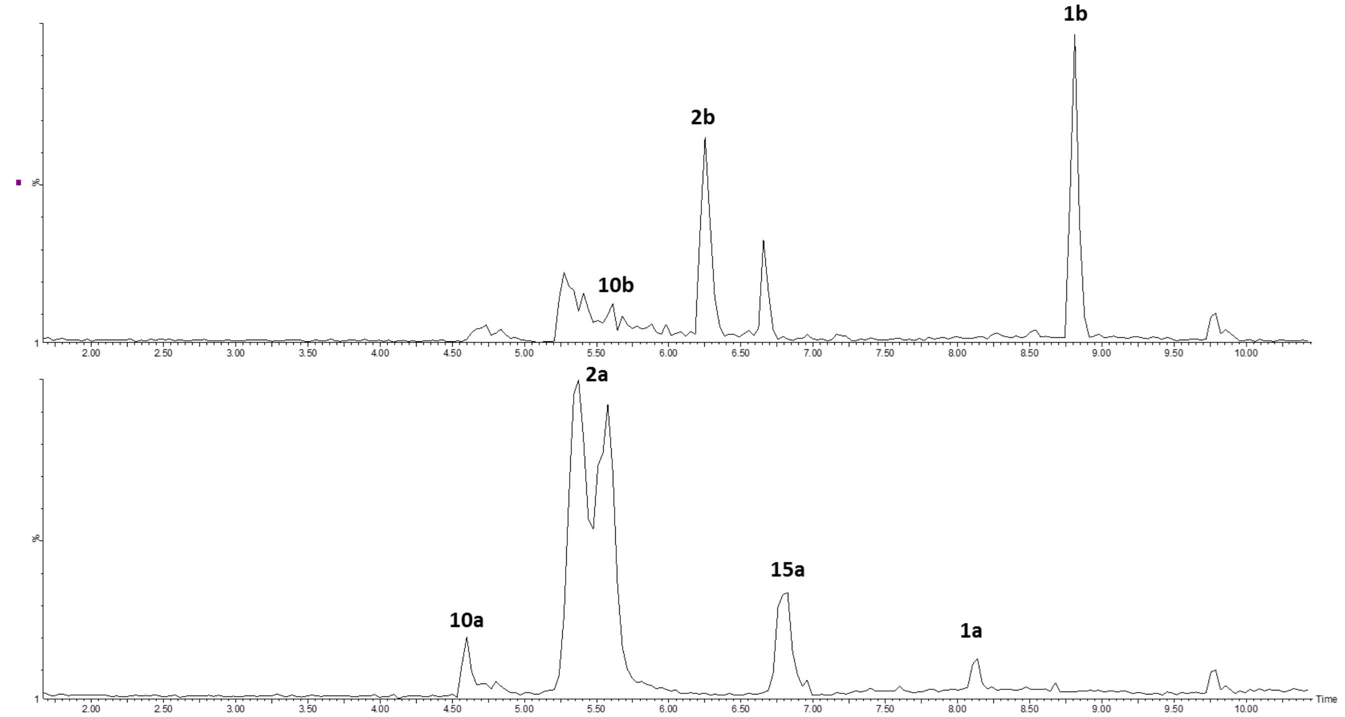

Figure S12: LCMS-chromatograms (ES$^-$ TIC) of **1b** incubated with SorC (top chromatogram) and of **1a** incubated with SorC for a prolonged incubation time and an excess of NADPH (bottom chromatogram).

In Vitro Experimental Design and Results

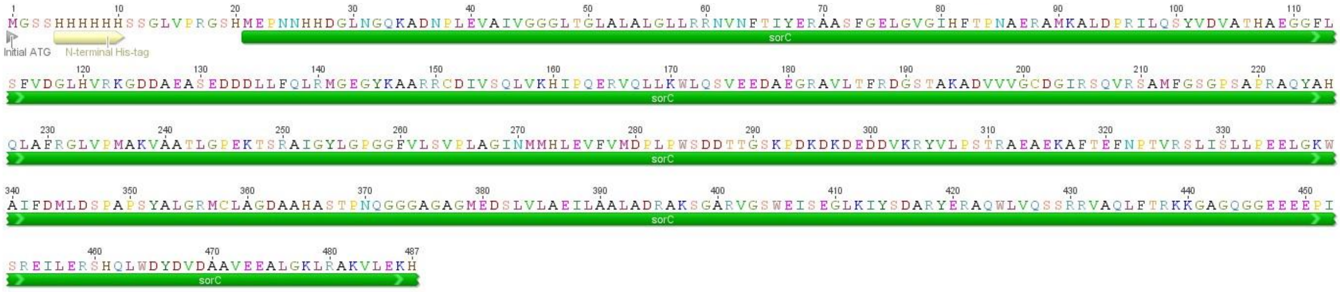

Figure S13: Protein sequence of SorC based on *T. reesei* QM6a cDNA.

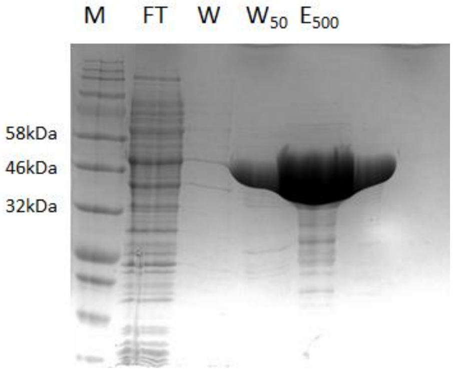

Figure S14: SDS-PAGE of SorC during purification. M: marker, FT: flow through, W: wash, W_{50}:wash (50 mM imidazole), E_{500}: elution. 12% SDS gel run at 40mA for 40 min.

```
ATGTACGCCCCCCCTTTTGTCCGCGCCTTCGGAATCGCAGTCCTTGCCGTCCTCCCATCTTTCTCTTCGCCCGCAACTGCCGCTTCTCTGAAATCGTCCGGATCTTCGTCATCGTGCAG
ATGCTTCCCGGGAGACGCCTGCTGGCCATCCCCGGCCGACTGGAAGGCCTTCAACCAGTCCGTCGGCGGACGTCTCATTGCCACGGTCCCGCTTGGATCCGTCTGCCATGGCACCACGT
ATGATGCCGCACGCTGCGCAGACGTCAAAGCCGCATGGCCGTATGCGGACACACACACCGACTCGTCTAGTTCCGTCCTTGCGCCTTTCTTTGCGAACCAGAGCTGCGATCCGTTTCTC
CCTAGAGAGACTCCCTGCGTTATCGGCACATATGTCCAGTACGCCGTCAACGTCTCCAGCGTTGCAGACATCCAAAAGACTCTTGCGTTTTCTCAGAAGAAGAATCTTCGCCTGGTAGT
GAGAAACACGGGCCATGATTACTTTGGAAAGTCCACTGGAGCCGGTGGTCTGGGACTATGGATGCATAATCTCAAGACTTACGATATCCACGACTACAAGTCGGCTGCTTACACTGGAA
AAGCGGTCACCATGGGCGCGGGCATCCAGGCGGGAGAGTCTGCTGCTACTGCTTTCAAGCAAGGCCTTACCATTGTGTCTGGGATATGTCCGACTGTTGGCCTGGCCGGAGGATACACC
CAGGGCGGCGGCTTAGGGCCCTTGACGACCAGATACGGTCTCGGAGCTGACCAGGTACTGGAGTGGCATGCGGTGCTTGCAAACGGCTCCGAAATCACTGCGACTCCAACCAAGAACAG
CGATCTGTACTGGGCCTTGACTGGTGGCGGTGGTGGCACATACGCCGTCGTGTACTCCATGACCGTCAAAGCACATGCAAACGAGAAGACCACAGGGGCGAATCTCACGTTTCCGAATG
CCGGGTCAGAAGATGTCTTCTTTCAGGGAGTTCAAGCATTTCACGATATTATACCTGCGATATCTGATGCCGGGGGCACGGCGGTCTGGACCGTGCTTTCCAAGGCCTTGTCCGTTGGG
CCAGTTACCGGGCCGAACATGACCAAGGCGACCATGGATAGCATCTTTCAGCCCGTGCTCCAGAAACTGGATGCTCTCAATATTACATACTCCTACTCCTCTGGGGAGTTTTCAAGCTT
TTATGAGAGCAACGCCGCTTATGACCCACCGGTGGTGTCAAATGGTCTCCAAATTGGCGGCAGACTCGTAAAGCGGTCTGACTTTACCGGGAACCCAGACGGCTTCATCCAGGCCATTC
GTGGTATCGCCGATCAAGGTGGACTGGTCACCGGGGCCTCATACCAGCTATCGAGCAGCCTCCAACACCCGCCCAACTCGGTGAACCCAGAGCTGAGGAAGAGCTTGATTTCGTTTCAG
ATCGGAGTACCCTGGATCAACACAGACTGGGCTACAGATCTTCACAACCAGGATCTCATCACCAACTCATTCGTACCCGCGCTGGCTGCCCTTCTTCCCAGTGGGGGCTCTGCTTACCT
TAACCAGGCGGACTTCCGGGAACCAGGATGGCAGCAGGTATTTTACGGAGAAAACTATGAGAAACTGCTTGAGTTGAAGGATGTGTATGACCCTAATGGGGTCTTCTGGGGAAGGACTA
CGGTTGGGAGCGAACGGTGGGCTGAGACGGAAGATAAGCGCCTTTGCCGAGTCTCGTAA
```

Figure S15: Coding sequence of sorD from *T. reesei* QM6a based on cDNA sequencing.

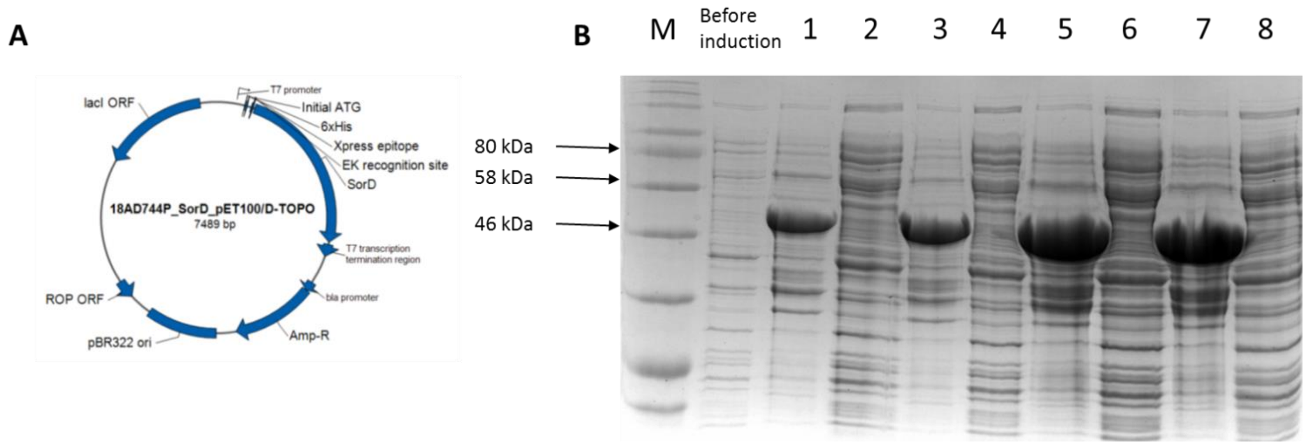

Figure S16: **A**, map of the expression plasmid pET100/D-TOPO-*sorD;* **B**, SDS-PAGE of *sorD*-expression using the plasmid pET-100/D-TOPO-*sorD* in *E. coli BL21*. M: protein ladder; 1: pellet fraction (12 °C, 0.1 mM IPTG); 2: soluble fraction (12 °C, 0.1 mM IPTG); 3: pellet fraction (12 °C, 0.5 mM IPTG); 4: soluble fraction (12 °C, 0.5 mM IPTG); 5: pellet fraction (16 °C, 0.1 mM IPTG); 6: soluble fraction (16 °C, 0.1 mM IPTG); 7: pellet fraction (16 °C, 0.5 mM IPTG); 8: soluble fraction (16 °C, 0.5 mM IPTG). 12% SDS gel run at 40 mA for 40 min.

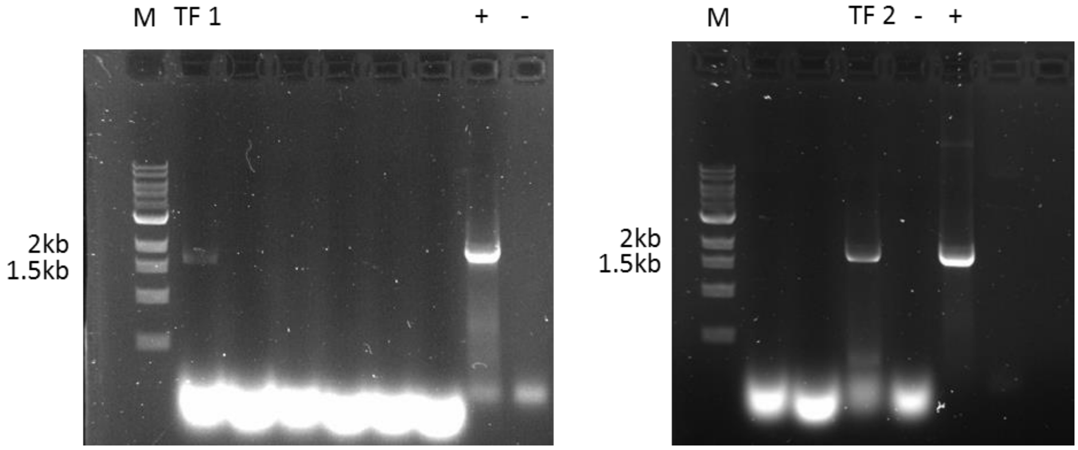

Figure S17: Confirmation of expression of *sorD* in +*sorABD* transformants by PCR using cDNA as template. M: DNA ladder marker; TF1: transformant 1; TF2: transformant 2; +: positive control using pTYGSade-*sorD* as template; -: negative control using freshly prepared RNA (from the respective TF tested) as template. 1% agarose gel run at 110 V for 20 min.

In Vivo Experimental Design and Results

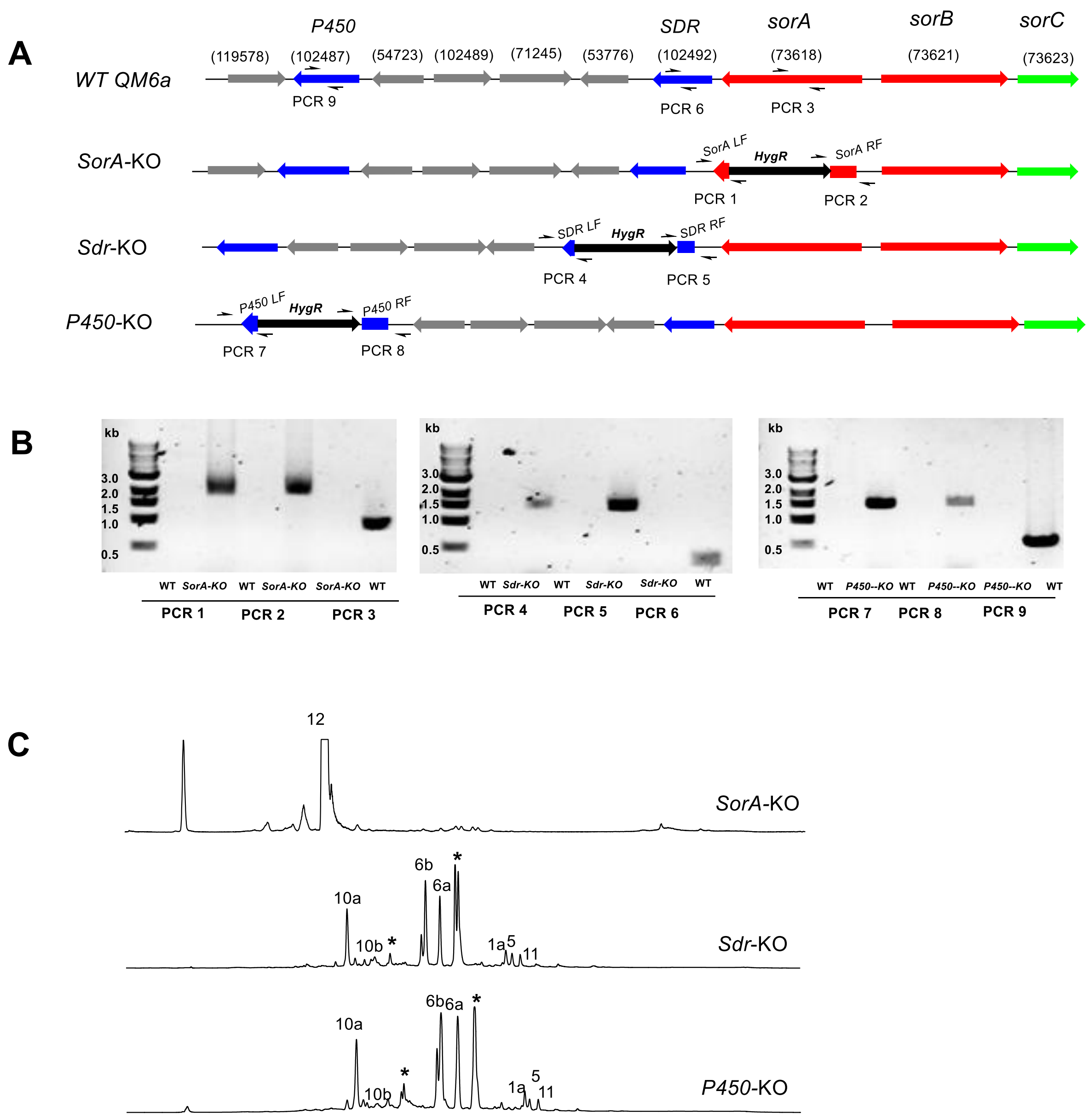

Figure S18. Deletion of *Sor*A, *Sdr* and *P450* genes in *T. reesei QM6a*. (A) The strain *QM6a∆Tmus35*, which contains the wild type gene cluster, like the parent strain *QM6a,* was transformed with bipartite fragments bearing the deletion cassette to replace the genes *sor*A, *sdr* and *P450* with hygromycin B resistance gene. **(B)** PCRs were performed to test the successful integration of the deletion cassette using chromosomal DNA from ∆*Tmus35* (WT), ∆*sor*A, ∆*sdr* and ∆*P450* as templates and the indicated PCRs Primers are shown by arrows and the primers sequences are found in Table (S2). **(C)** LCMS analysis of the cultured strains ∆*sor*A, ∆*sdr* and ∆*P450* on ME medium showing the produced metabolites for each strain.

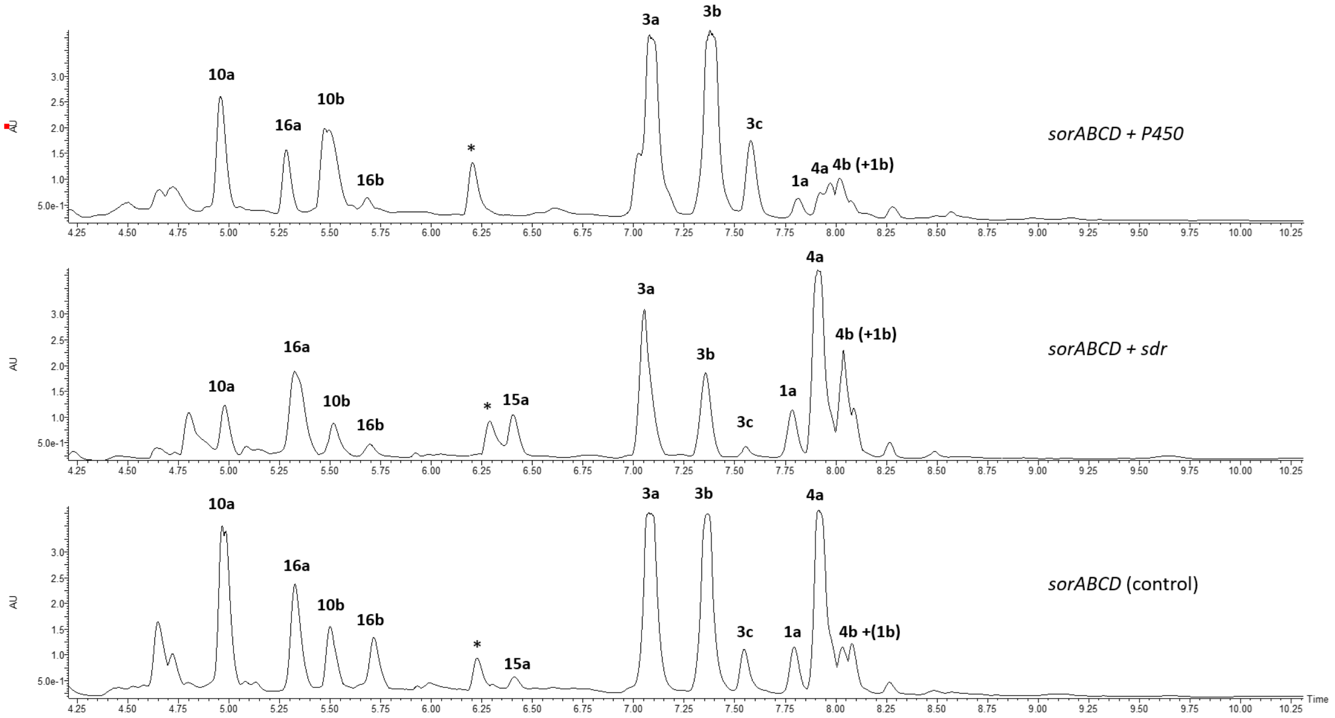

Figure S19. Heterologous co-expression of the cryptic P450 gene (top) chromatogram (DAD 210-600nm) or cryptic sdr gene (middle) with sorABCD. No differences in metabolite profile were observed compared to the sorABCD control strain (bottom).

Compound Physical Data

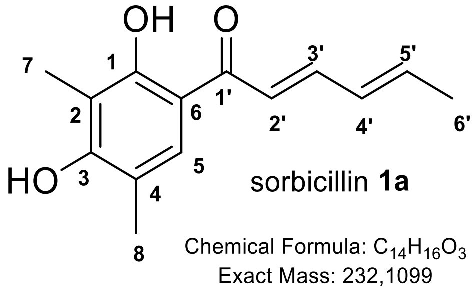

UV$_{\lambda max}$ (MeOH): 206 nm, 323 nm
HRMS (ESI) *m/z* (*M*-H)$^-$ calcd for $C_{14}H_{15}O_3$: 231.1021, found: 231.1022

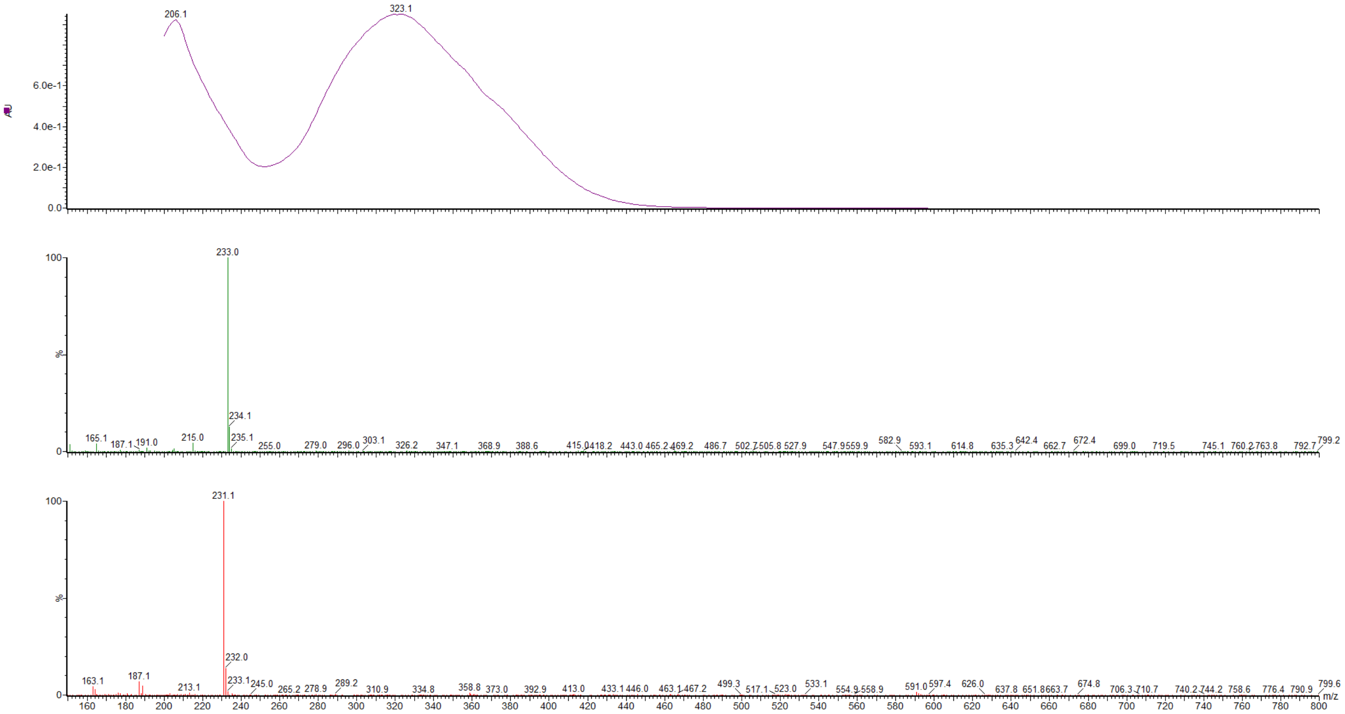

Figure S20: UV-absorption (top) and fragmentation pattern of **1a** in ES$^+$ TIC (middle) and ES$^-$ TIC (bottom).

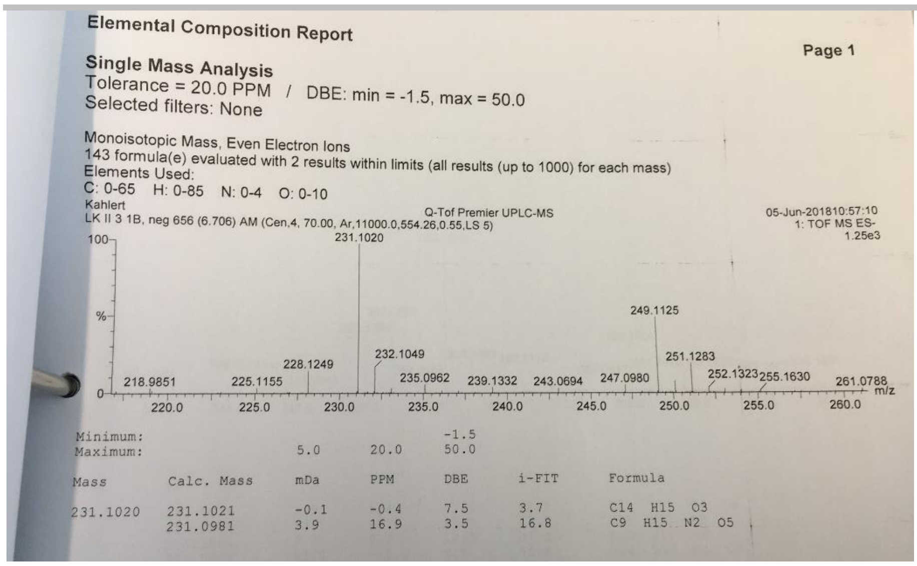

Figure S21: HRMS data for **1a**.

Table S5: Summarized NMR data for **1a** recorded at 400 MHz in $CDCl_3$.

Position	δ_C	δ_H	M	$J_{H\text{-}H}$/Hz	HSQC	HMBC H to C	H-H COSY
1	162.7	13.59	s	-	-	1, 2, 7	-
2	110.5	-	-	-	-	-	-
3	158.9	-	-		-	-	-
4	114.5	-	-	-	-	-	-
5	128.9	7.44	s	-	CH	1, 3, 8, 1‘	8
6	113.7	-	-	-	-	-	-
7	7.7	2.15	s	-	CH_3	1, 2, 3	-
8	15.8	2.22	s	-	CH_3	3, 4, 5	5
1‘	192.7	-	-	-	-	-	-
2‘	122.0	6.94	d	14.8	CH	1‘, 4‘	3‘, 6‘
3‘	144.7	7.48	dd	10.2, 14.8	CH	1‘, 5‘	2‘, 6‘
4‘	130.7	6.33	m	-	CH	2‘, 3‘, 6‘	6‘
5‘	141.3	6.29	m	-	CH	3‘, 6‘	6‘
6‘	19.1	1.91	d	6.2	CH_3	3’, 4’, 5’	2’, 3’, 4’, 5’

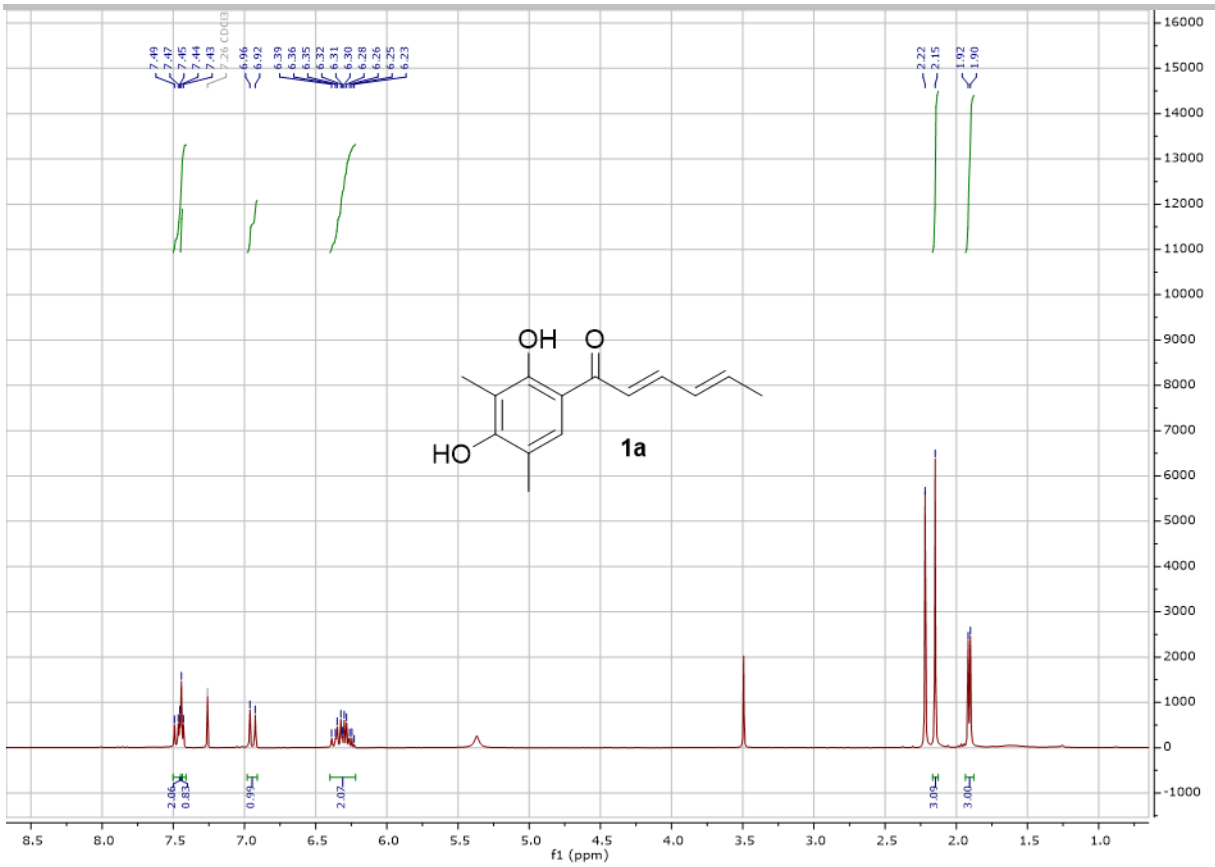

Figure S22: ^{1}H-NMR spectrum for **1a** recorded at 400 MHz in $CDCl_3$.

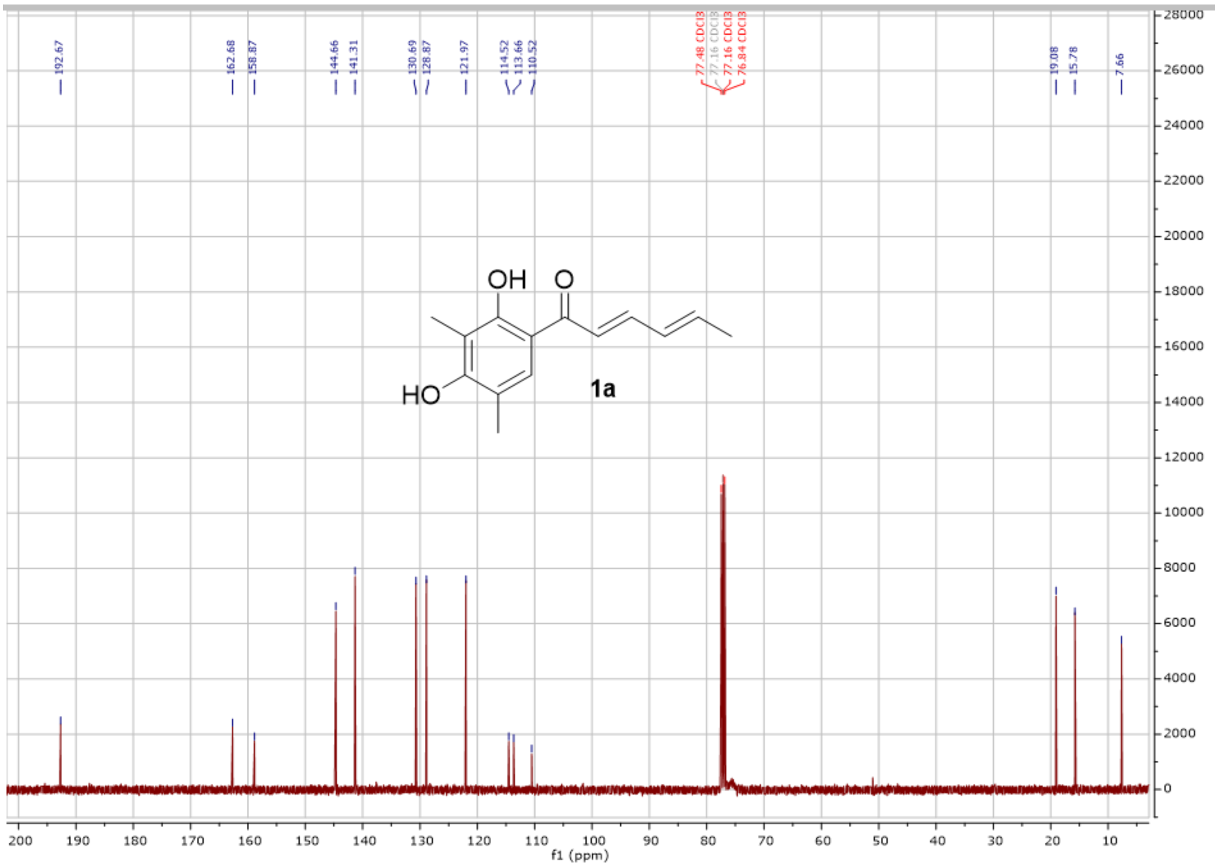

Figure S23: ^{13}C-NMR spectrum for **1a** recorded at 125 MHz in $CDCl_3$.

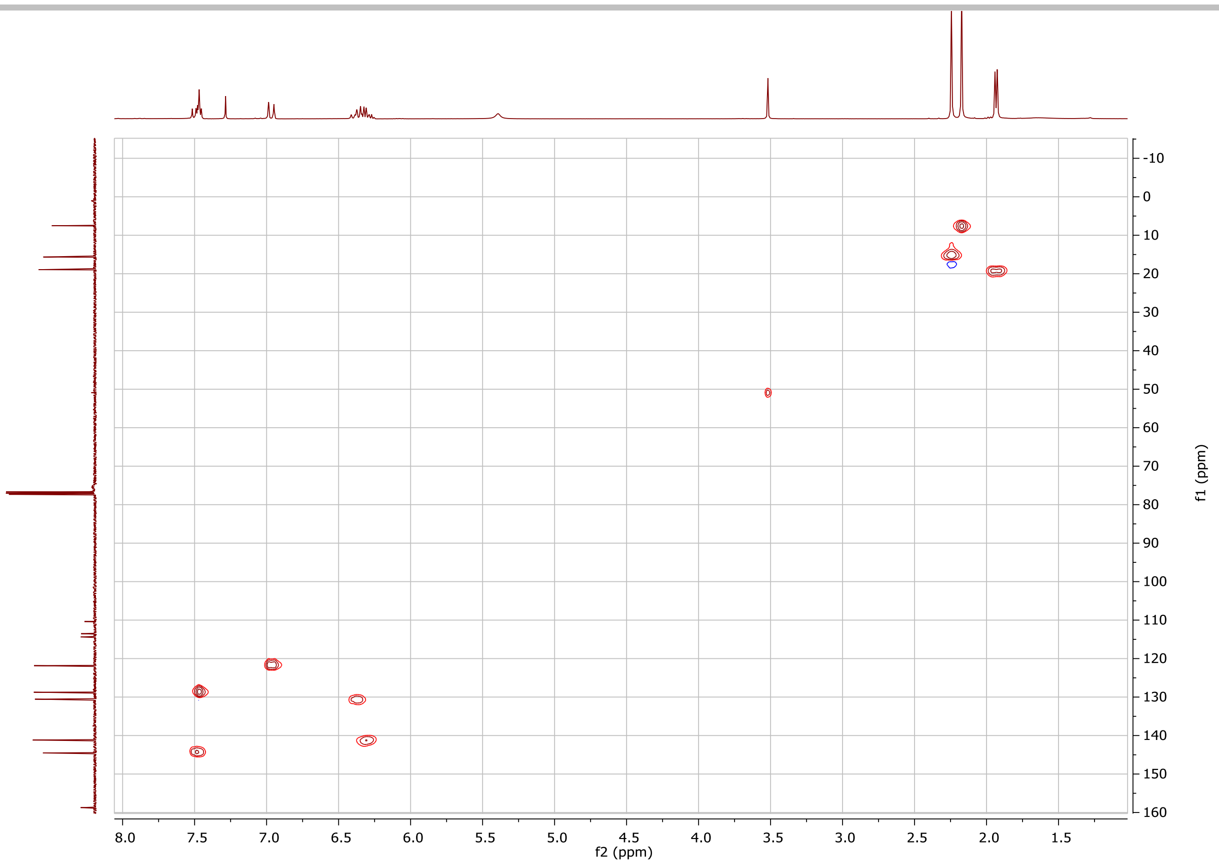

Figure S24: HSQC spectrum for **1a** recorded at 400 MHz in $CDCl_3$.

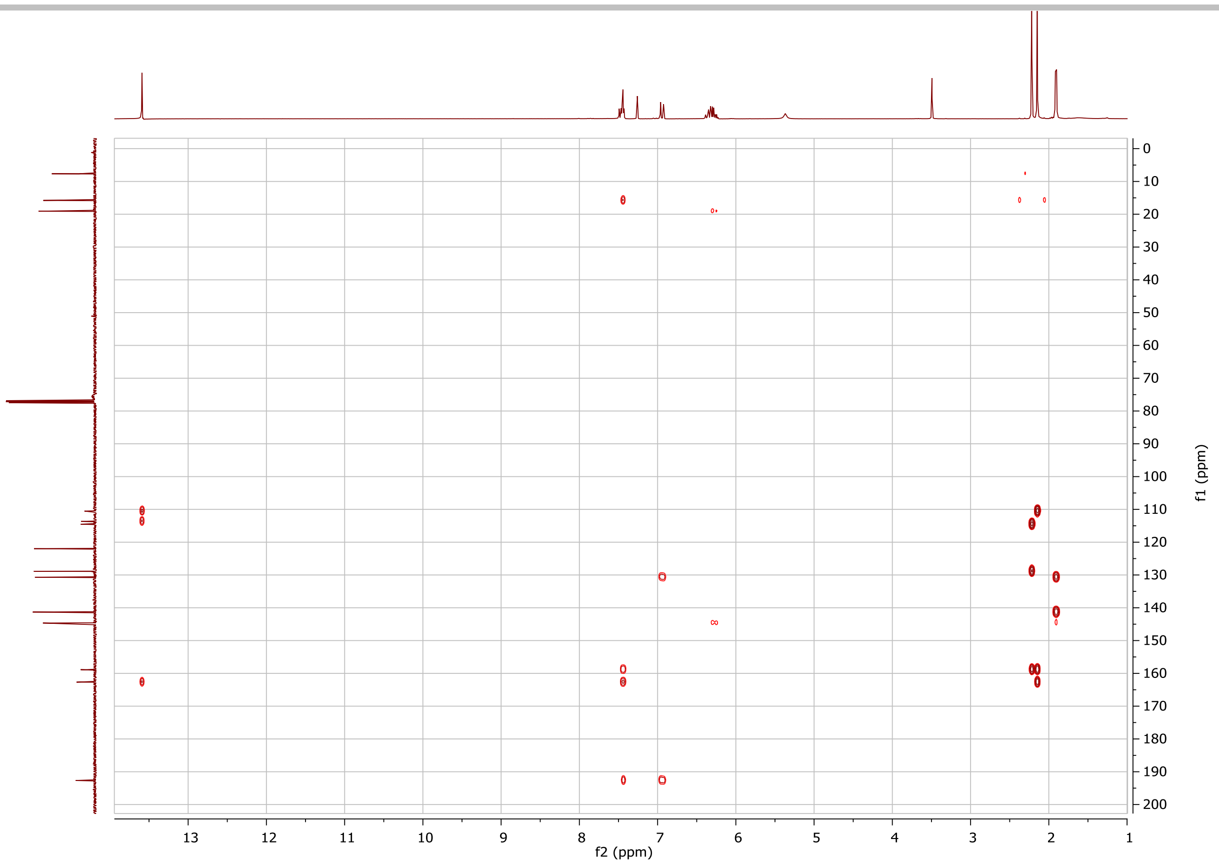

Figure S25: HMBC spectrum for **1a** recorded at 400 MHz in $CDCl_3$.

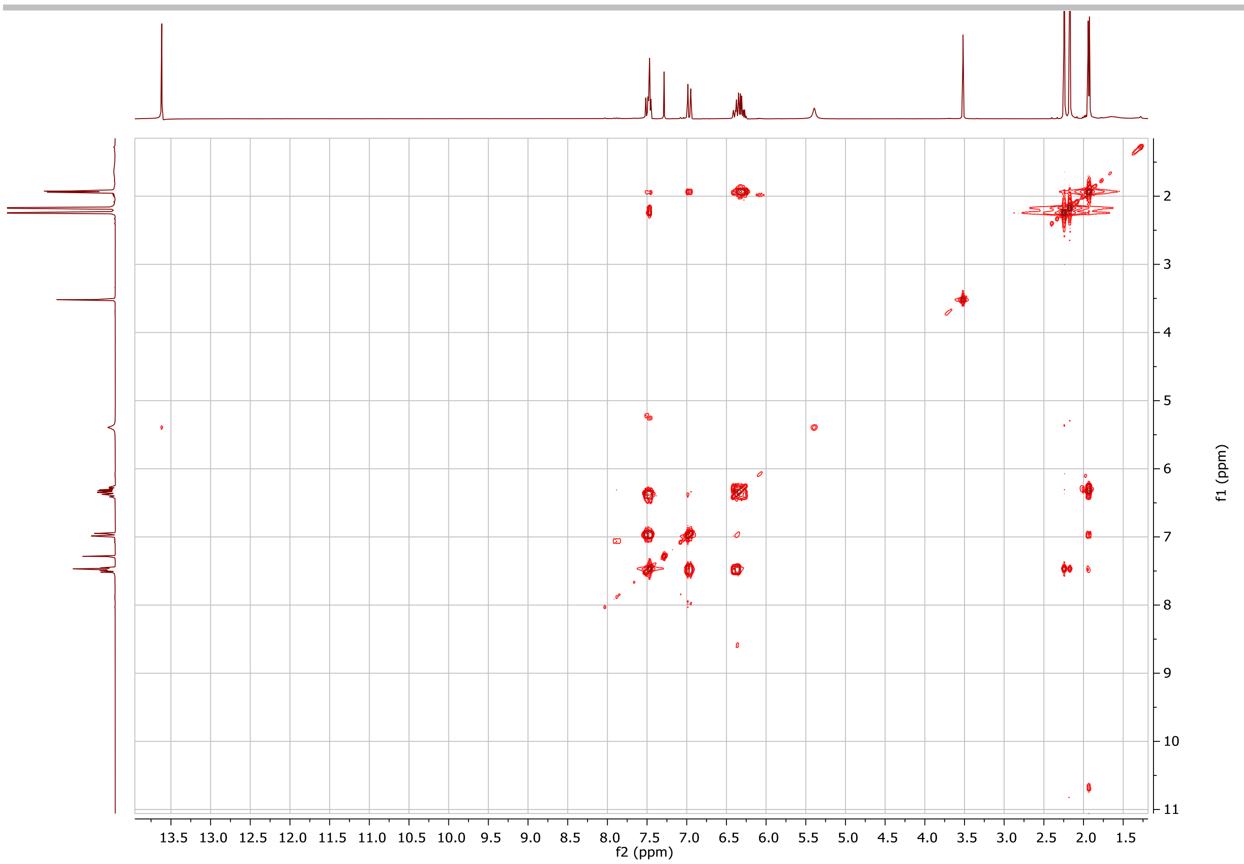

Figure S26: ^{1}H-^{1}H COSY spectrum for **1a** recorded at 400 MHz in $CDCl_3$.

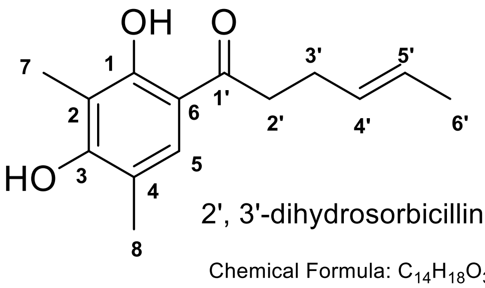

2', 3'-dihydrosorbicillin **1b**

Chemical Formula: $C_{14}H_{18}O_3$
Exact Mass: 234,1256

UV$_{\lambda max}$ (MeOH): 215 nm, 283 nm, 332 nm
HRMS (ESI) *m/z* (*M*-H)$^-$ calcd for $C_{14}H_{17}O_3$: 233.1178, found: 233.1177

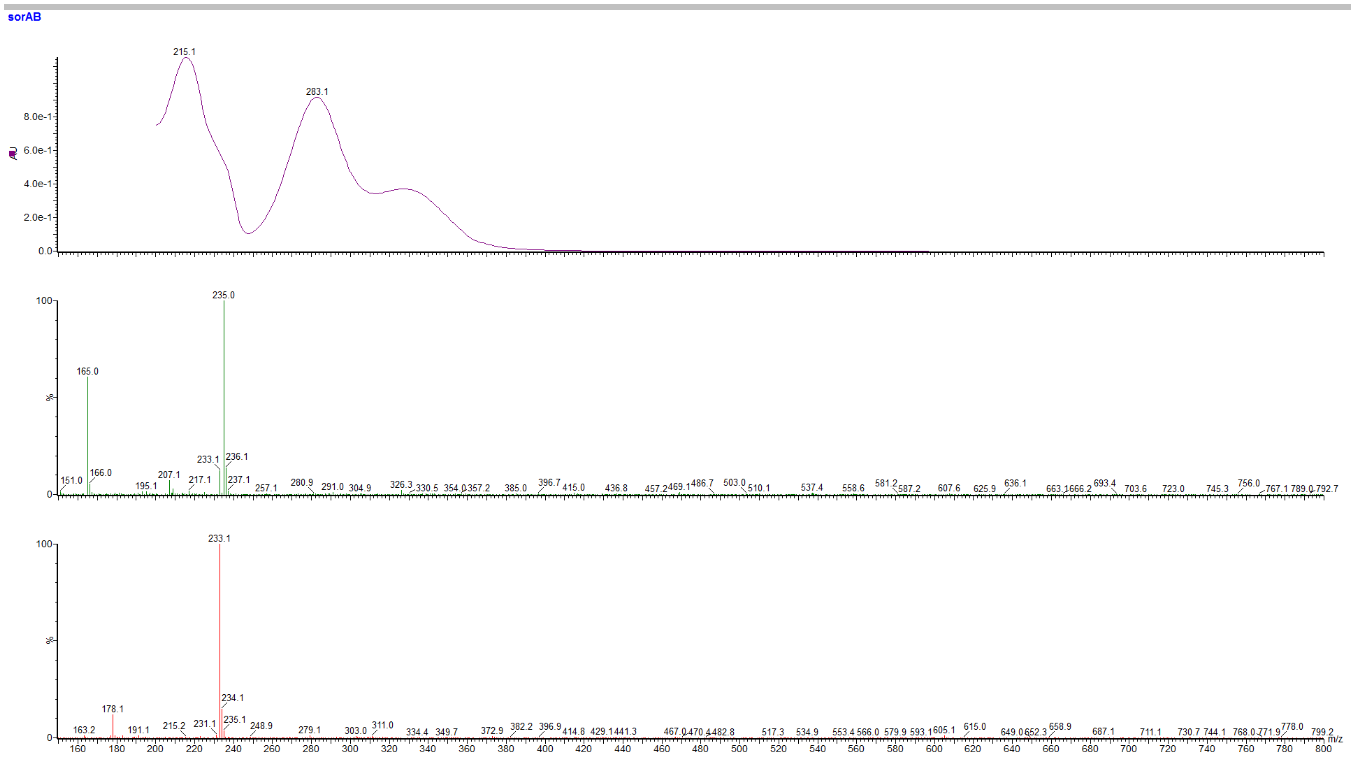

Figure S27: UV-absorption (top) and fragmentation pattern of **1b** in ES+ TIC (middle) and ES- TIC (bottom).

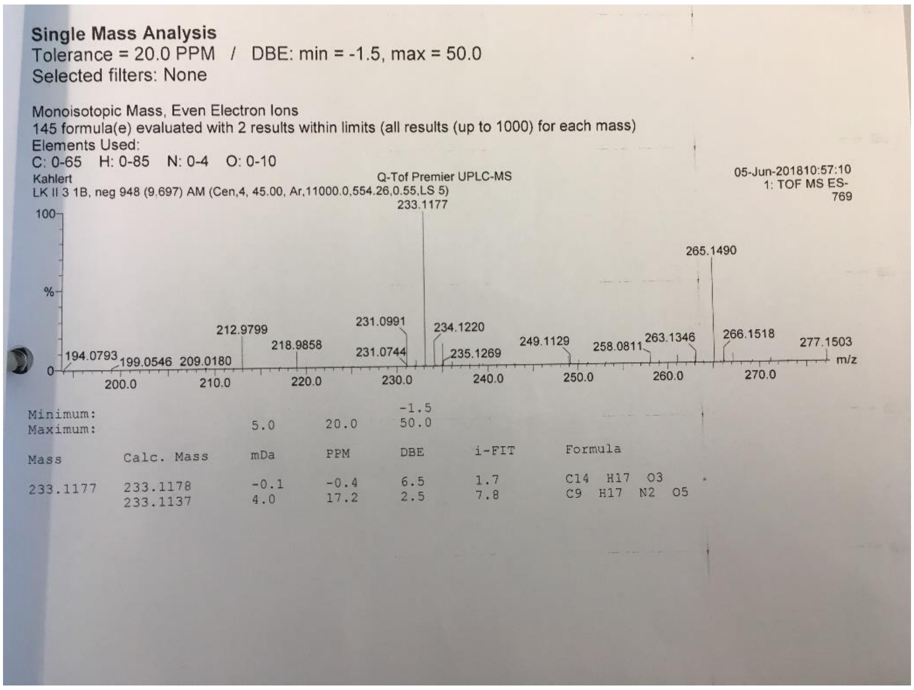

Figure S28: HRMS data for **1b**.

Table S6: Summarized NMR data for **1b** recorded in $CDCl_3$.

Position	δ_C	δ_H	M	$J_{H\text{-}H}$/Hz	HSQC	HMBC H to C	H-H COSY
1	161.5	12.97	s	-	-	1, 2, 3, 6	-
2	110.4	-	-	-	-	-	-
3	158.7	-	-	-	-	-	-
4	114.5	-	-	-	-	-	-
5	129.3	7.39	s	-	CH	1, 2, 3, 6, 8	8
6	113.1	-	-	-	-	-	-
7	7.6	2.16	s	-	CH_3	1, 2, 3	-
8	15.7	2.23	s	-	CH_3	3, 4, 5	-
1‘	204.5	-	-	-	-	-	-
2‘	38.0	2.99	t	12.0	CH_2	1‘, 3‘, 5‘, 6‘	3‘, 4‘
3‘	27.7	2.43	m	-	CH_2	1‘, 2‘, 4‘	2‘, 4‘
4‘	126.3	5.53	m	-	CH	3‘, 6‘	3‘
5‘	129.7	5.53	m	-	CH	3‘, 6‘	6‘
6‘	18.0	1.68	d	4.74	CH_3	5’	4’, 5’

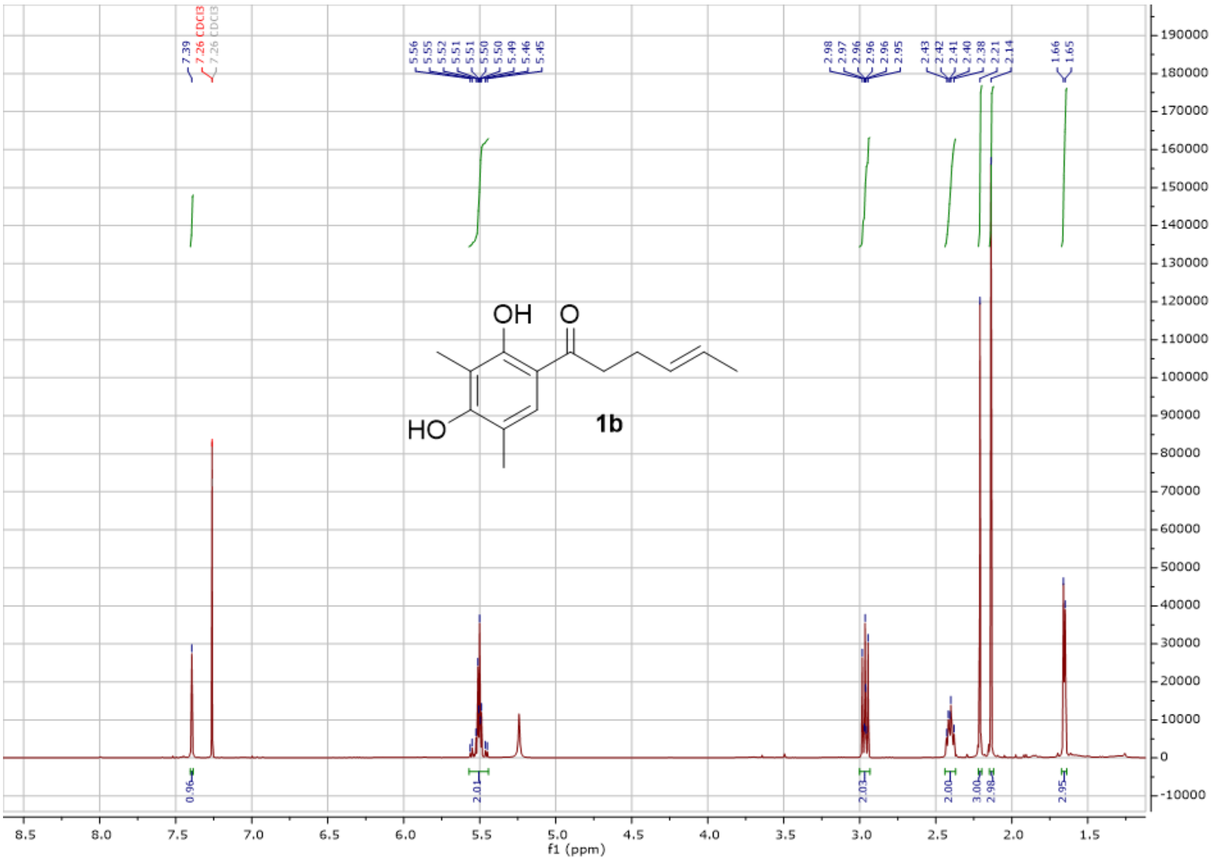

Figure S29: ^{1}H-NMR spectrum for **1b** recorded at 400 MHz in $CDCl_3$.

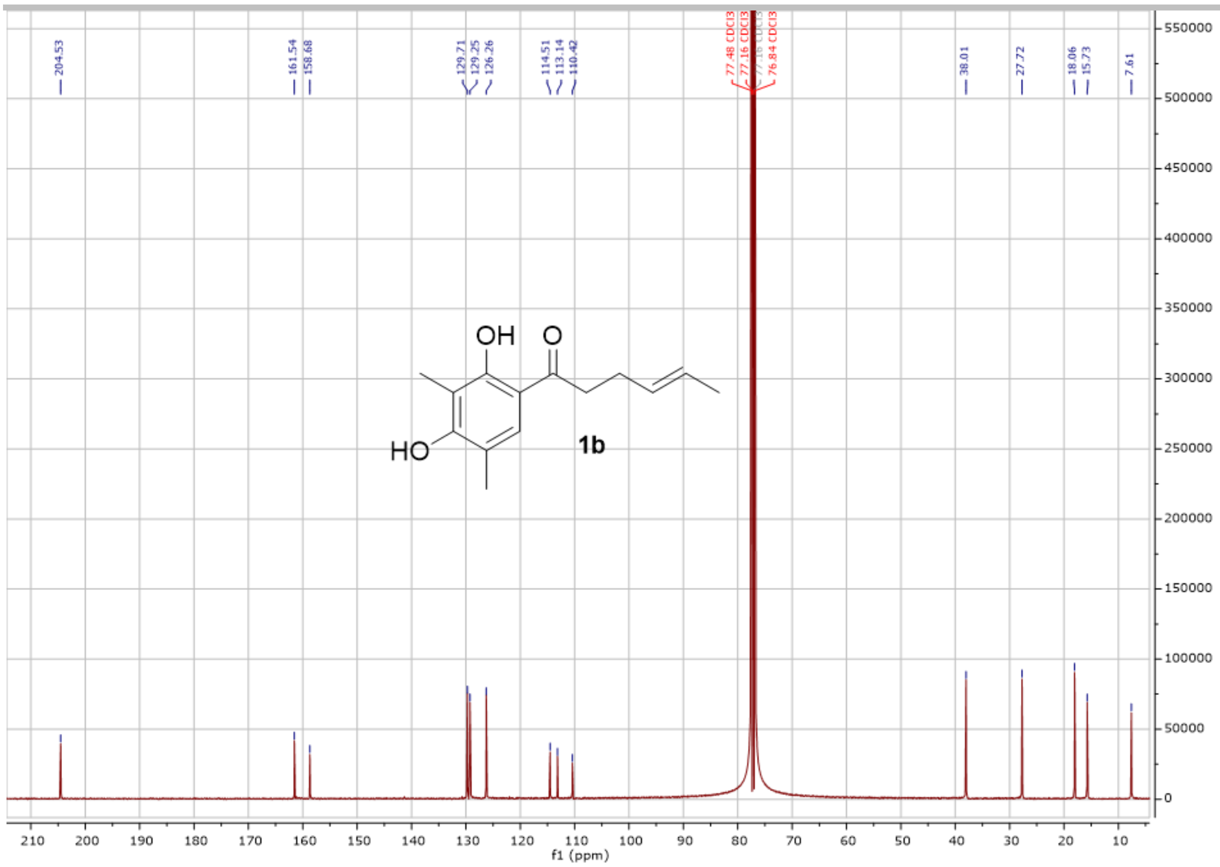

Figure S30: ^{13}C-NMR spectrum for **1b** recorded at 125 MHz in $CDCl_3$.

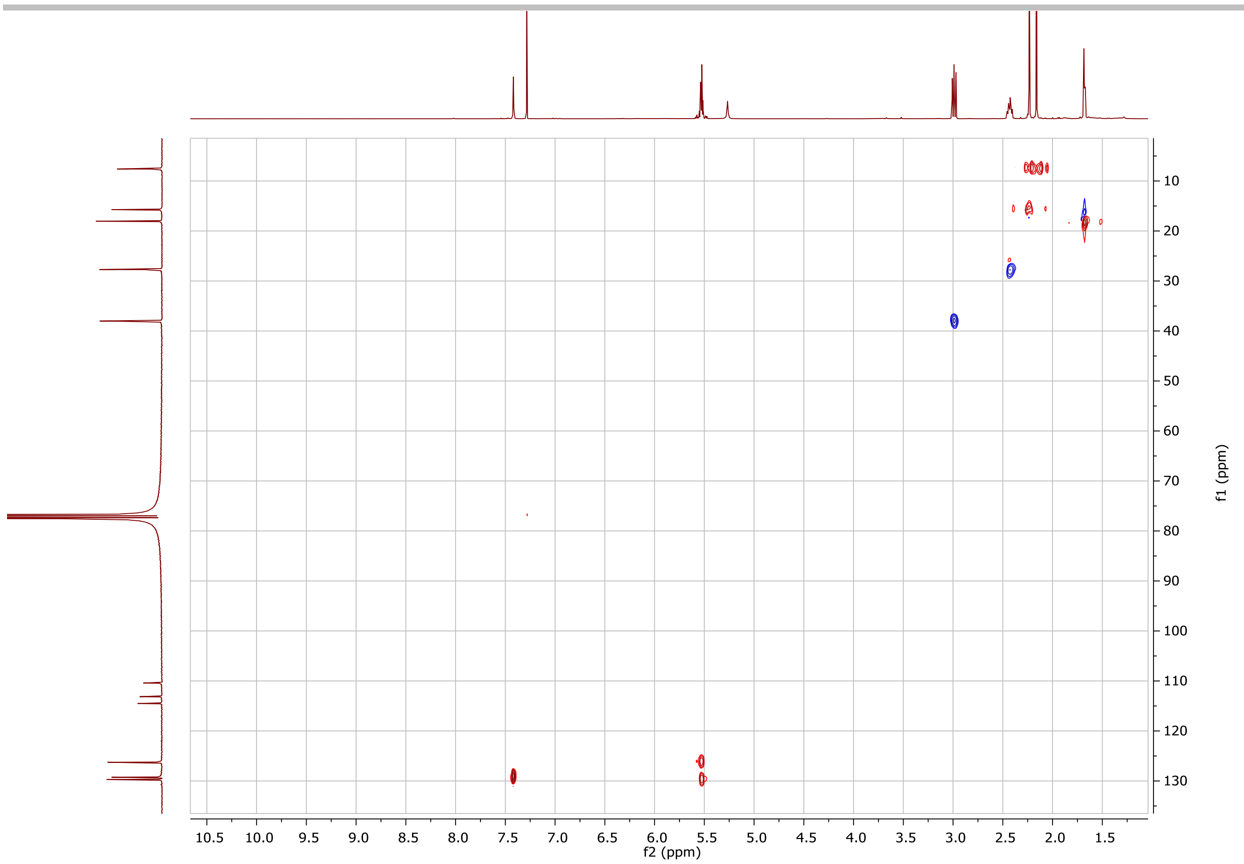

Figure S31: HSQC spectrum for **1b** recorded at 400 MHz in $CDCl_3$.

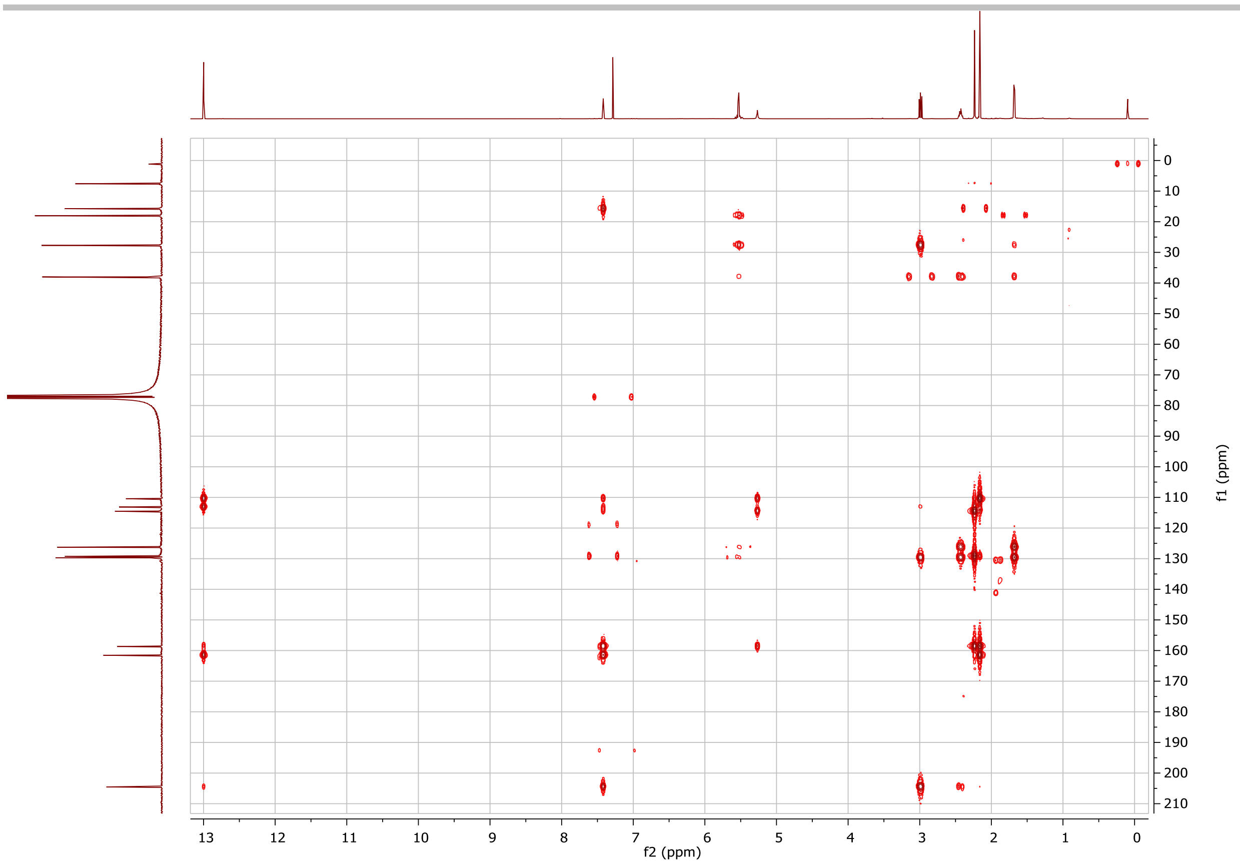

Figure S32: HMBC spectrum for **1b** recorded at 400 MHz in $CDCl_3$.

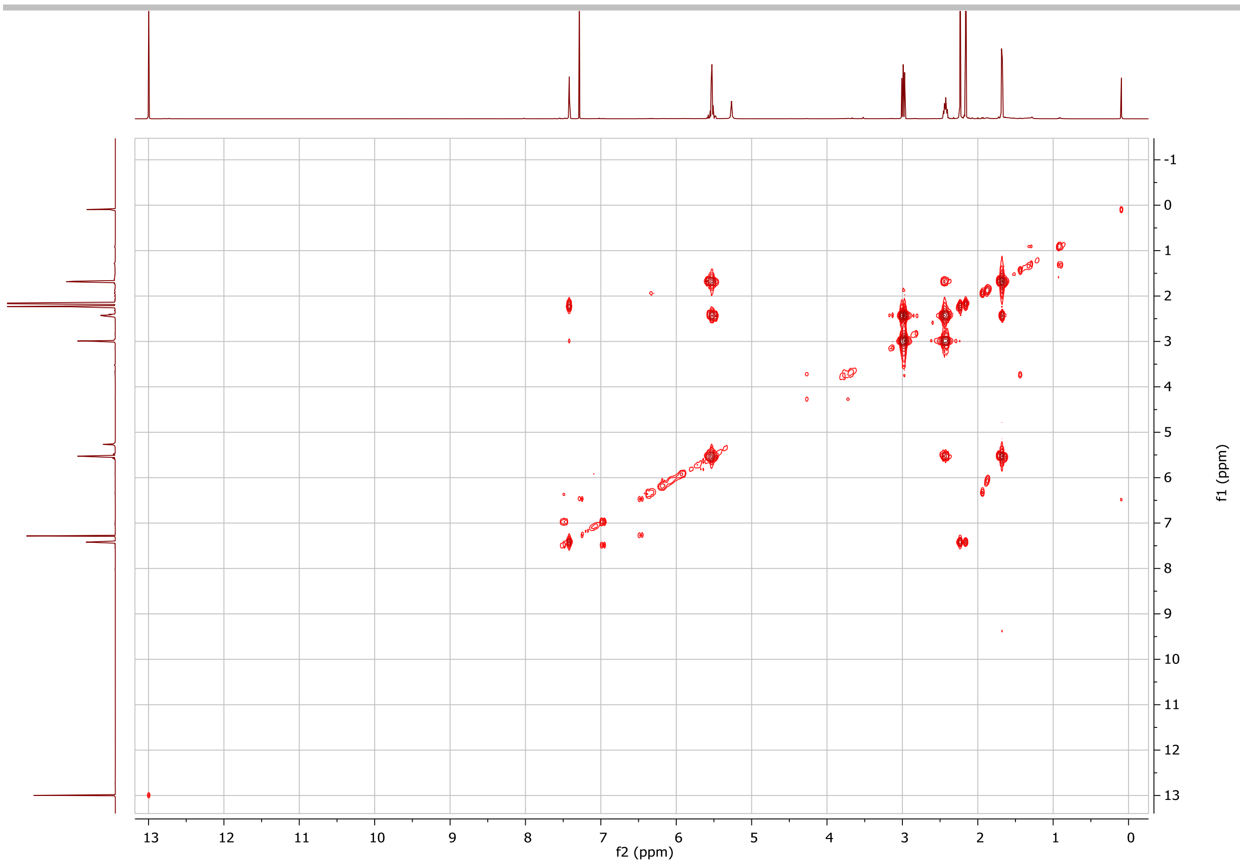

Figure S33: ^{1}H-^{1}H COSY spectrum for **1b** recorded at 400 MHz in $CDCl_3$.

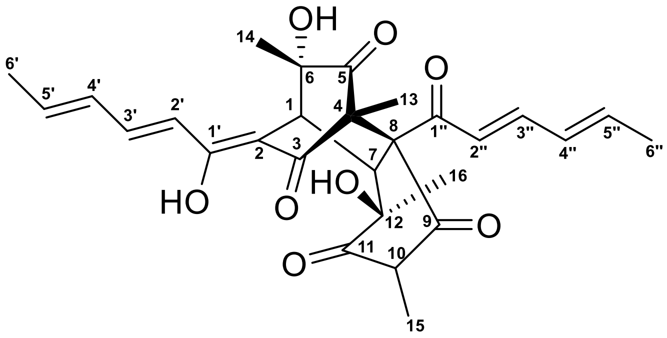

bisorbicillinol **3a**

Chemical Formula: $C_{28}H_{32}O_8$
Exact Mass: 496,2097

UV$_{\lambda max}$ (MeOH): 298 nm, 383 nm
HRMS (ESI) *m/z* (*M*-H)$^-$ calcd for $C_{28}H_{31}O_8$: 495.2019, found: 495.2018

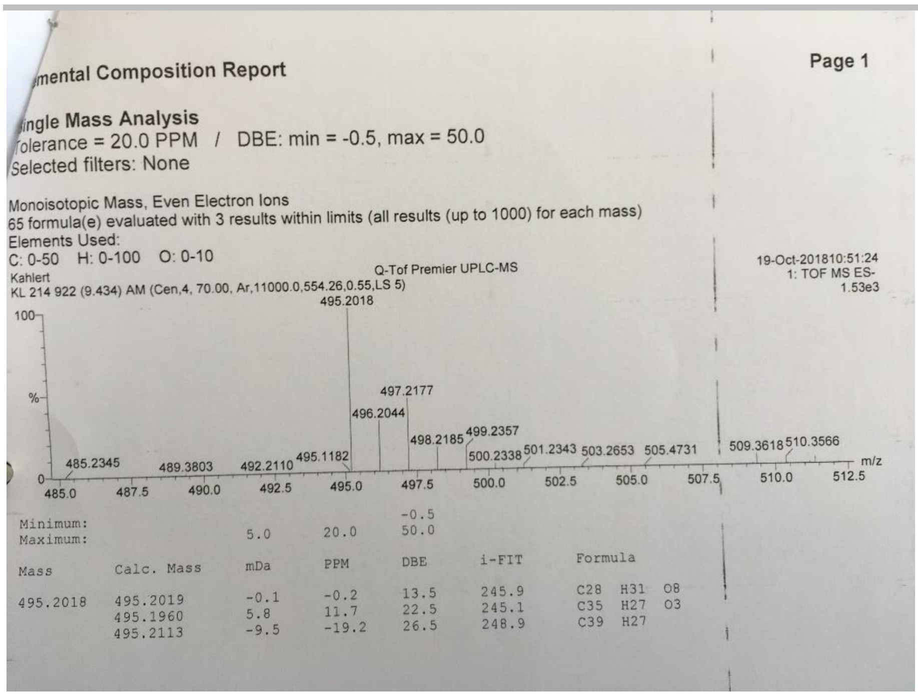
...mental Composition Report

Page 1

...ingle Mass Analysis
...olerance = 20.0 PPM / DBE: min = -0.5, max = 50.0
Selected filters: None

Monoisotopic Mass, Even Electron Ions
65 formula(e) evaluated with 3 results within limits (all results (up to 1000) for each mass)
Elements Used:
C: 0-50 H: 0-100 O: 0-10
Kahlert
KL 214 922 (9.434) AM (Cen,4, 70.00, Ar,11000.0,554.26,0.55,LS 5)

Q-Tof Premier UPLC-MS

19-Oct-201810:51:24
1: TOF MS ES-
1.53e3

Minimum:				-0.5		
Maximum:		5.0	20.0	50.0		
Mass	Calc. Mass	mDa	PPM	DBE	i-FIT	Formula
495.2018	495.2019	-0.1	-0.2	13.5	245.9	C28 H31 O8
	495.1960	5.8	11.7	22.5	245.1	C35 H27 O3
	495.2113	-9.5	-19.2	26.5	248.9	C39 H27

Figure S34: HRMS data for **3a**.

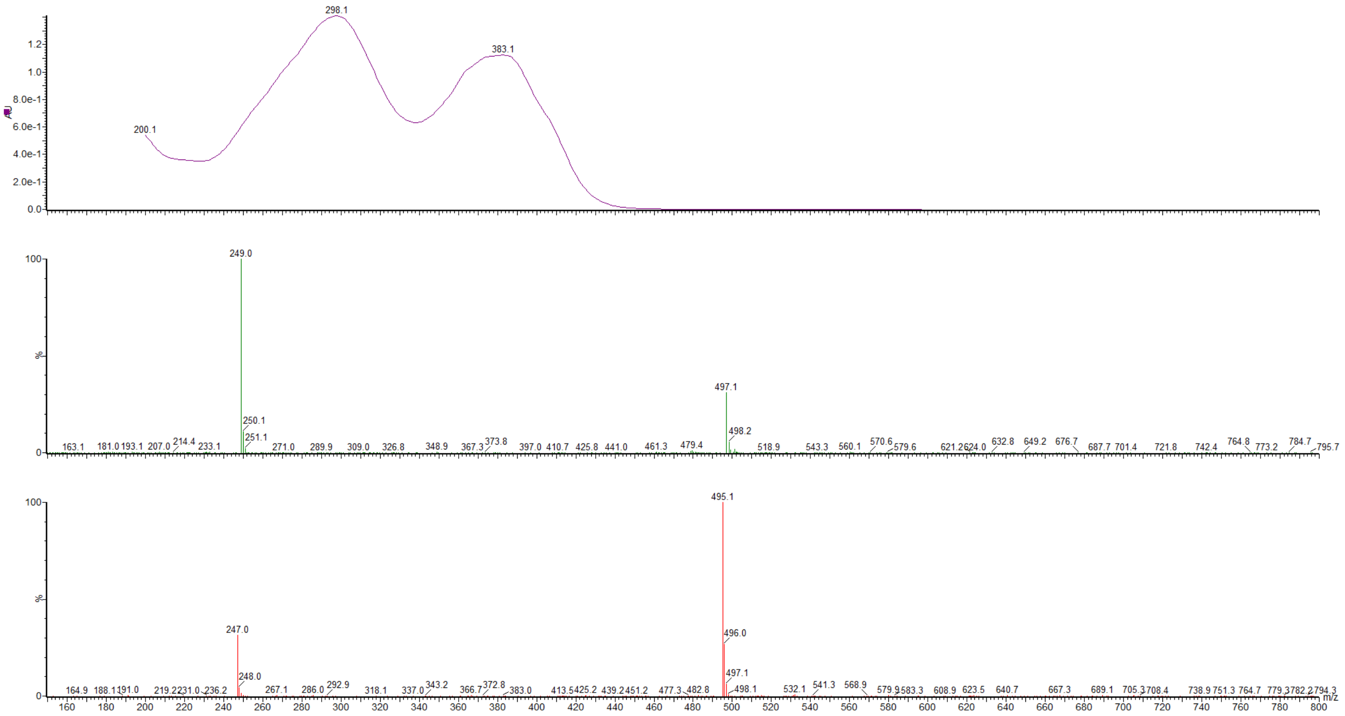

Figure S35: UV-absorption (top) and fragmentation pattern of **3a** in ES+ TIC (middle) and ES- TIC (bottom).

Table S7: Summarized NMR data for **3a** recorded in $CDCl_3$.

Position	δ_C	δ_H	M	$J_{H\text{-}H}$/Hz	HSQC	HMBC H to C	H-H COSY
1	41.5	3.70	d	3.0	CH	2, 5, 6, 8 14, 1‘	7
2	108.8	-	-	-	-	-	-
3	194.8	-	-	-	-	-	-
4	65.1	-	-	-	-	-	-
5	203.9	-	-	-	-	-	-
6	73.6	-	-	-	-	-	-
7	48.4	3.43	d	3.0	CH	1, 2, 8 11, 12, 16, 1“	1
8	70.9	-	-	-	-	-	-
9	202.8	-	-	-	-	-	-
10	53.9	2.91	q	7.1	CH	9, 11, 12, 15,	15
11	209.4	-	-	-	-	-	-
12	77.4	-	-	-	-	-	-
13	9.4	1.18	s	-	CH_3	3, 4, 5, 8,	-
14	24.8	1.27	s	-	CH_3	1, 5, 6	-
15	13.6	1.38	d	7.1	CH_3	9, 10, 11	10
16	26.6	1.36	s	-	CH_3	7, 11, 12	-
1‘	173.1	-	-	-	-	-	-
2‘	117.9	6.16	d	7.1	CH	1‘, 4‘, 5‘	3‘
3‘	145.6	7.42	m	-	CH	1‘, 4‘	2‘
4‘ = 4“	142.4	7.27	dd	10.9, 14.9	CH	2‘	5‘, 2“
5‘ = 5“	130.6	6.25	m	-	CH	2‘, 6‘, 3‘	4‘, 6‘
6‘ = 6“	19.3	1.91	d	6.7	CH_3	4‘, 5‘	5‘
1“	196.9	-	-	-	-	-	-
2“	123.1	6.33	m	-	CH	1“, 3“ 5“,	3“, 4“
3“	147.4	7.37	m	-	CH	1“, 4“, 5“	2“

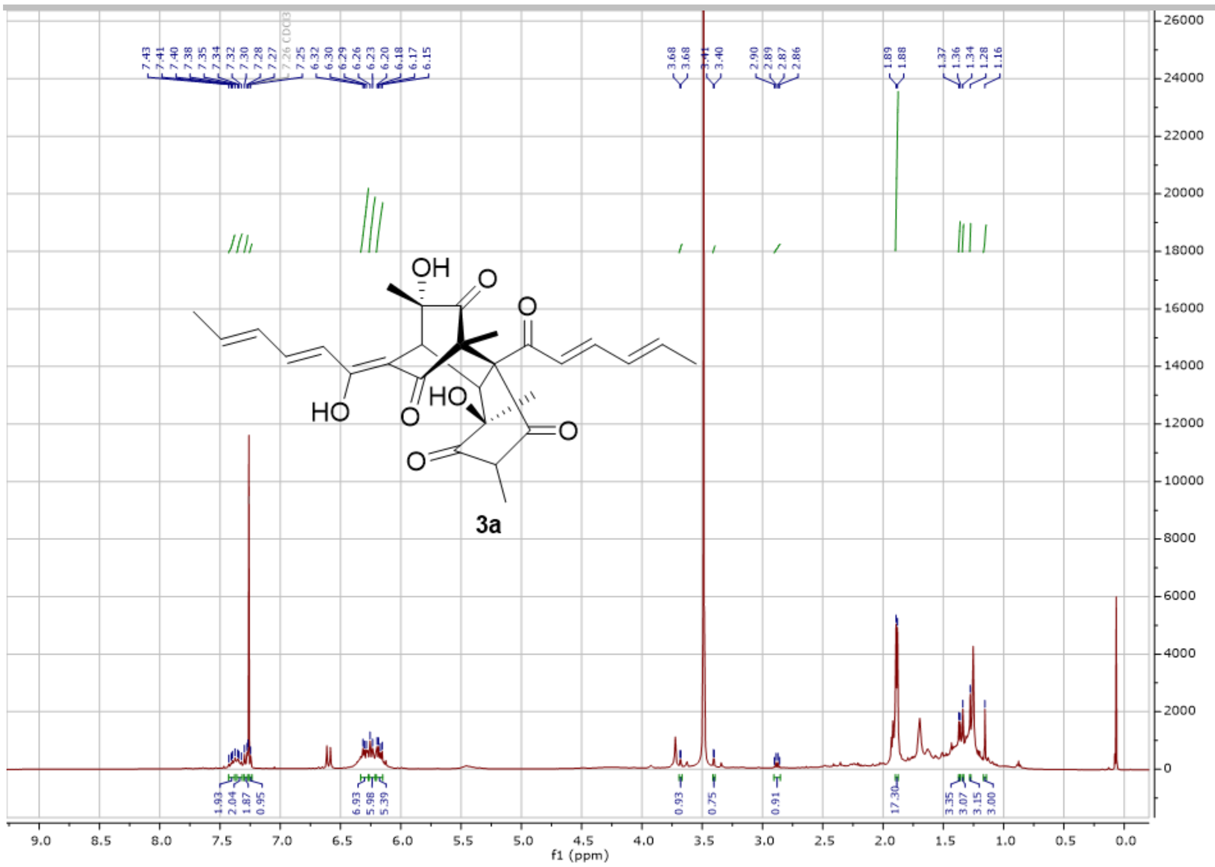

Figure S36: ^{1}H-NMR spectrum for **3a** recorded at 500 MHz in $CDCl_3$.

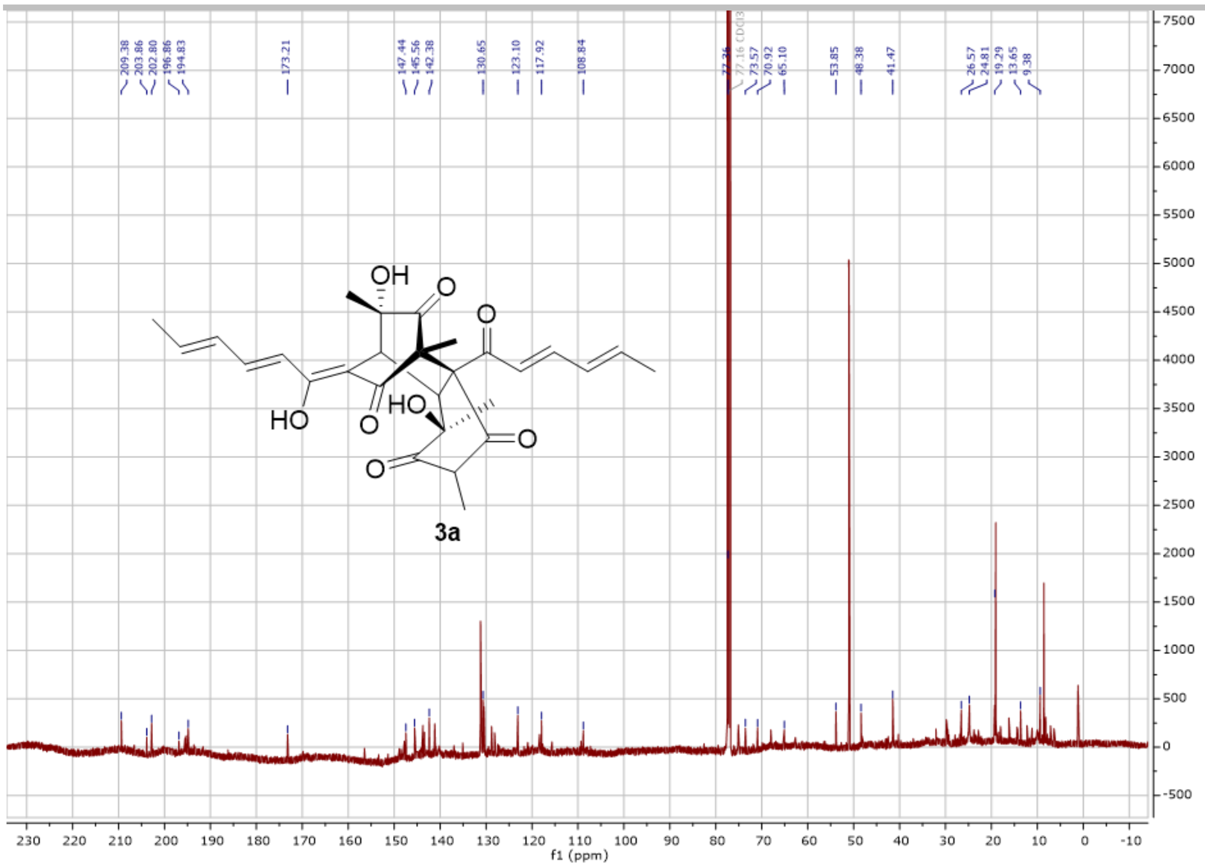

Figure S37: ^{13}C-NMR spectrum for **3a** recorded at 125 MHz in $CDCl_3$.

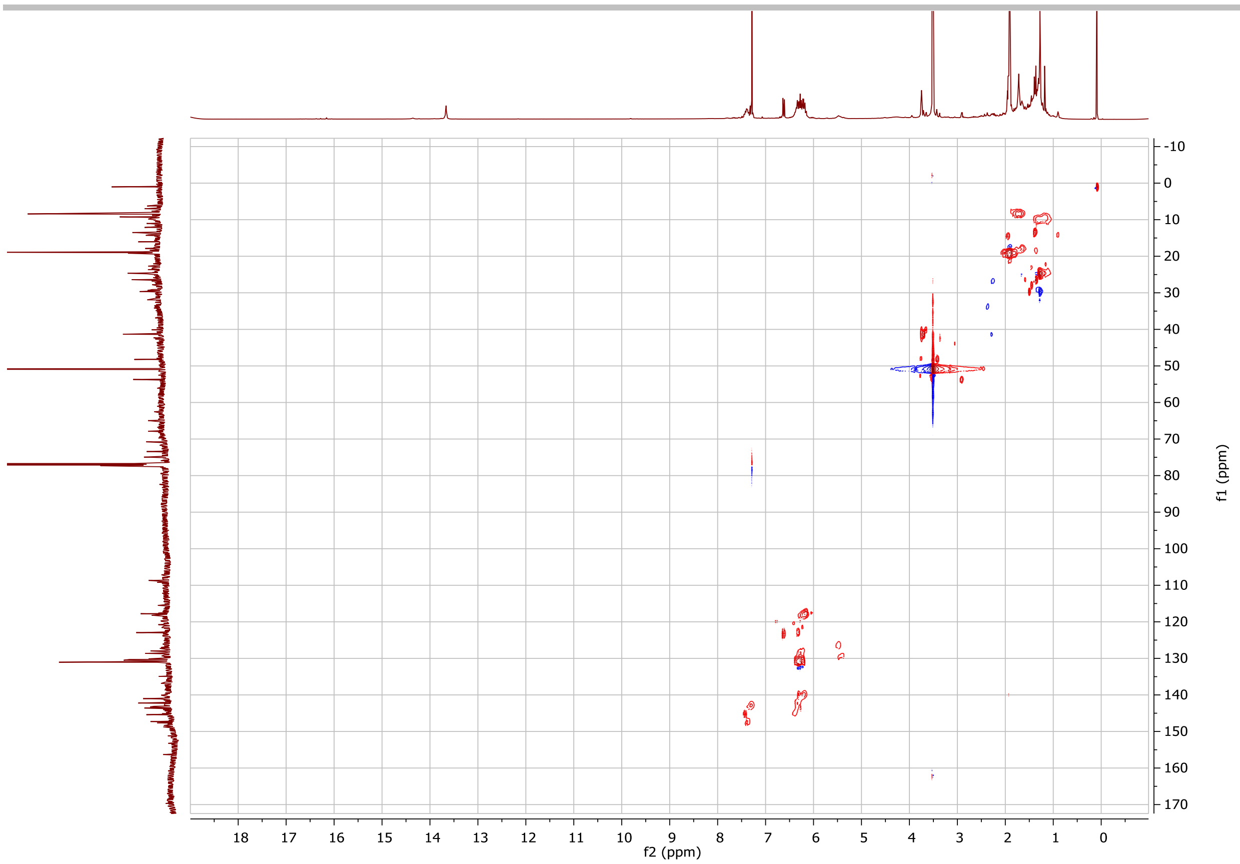

Figure S38: HSQC spectrum for **3a** recorded at 500 MHz in $CDCl_3$.

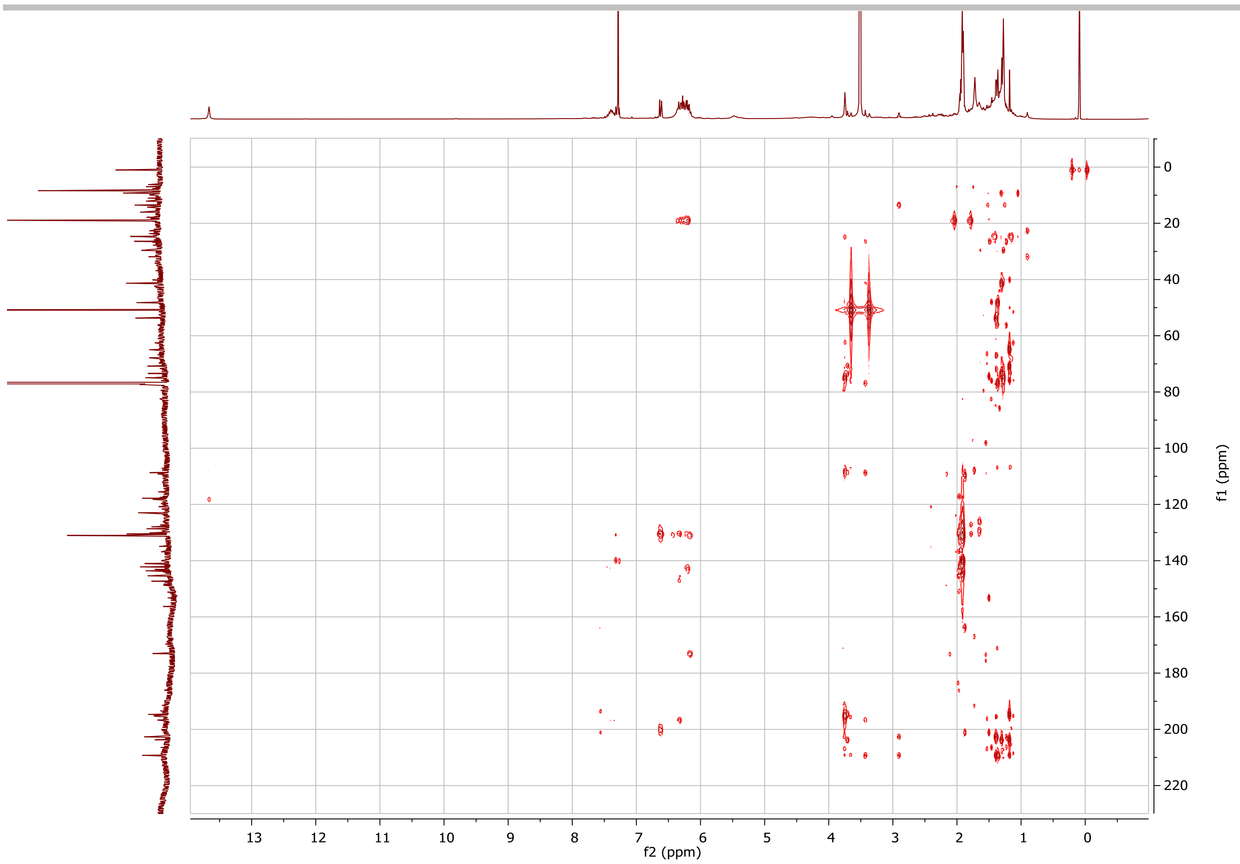

Figure S39: HMBC spectrum for **3a** recorded at 500 MHz in $CDCl_3$.

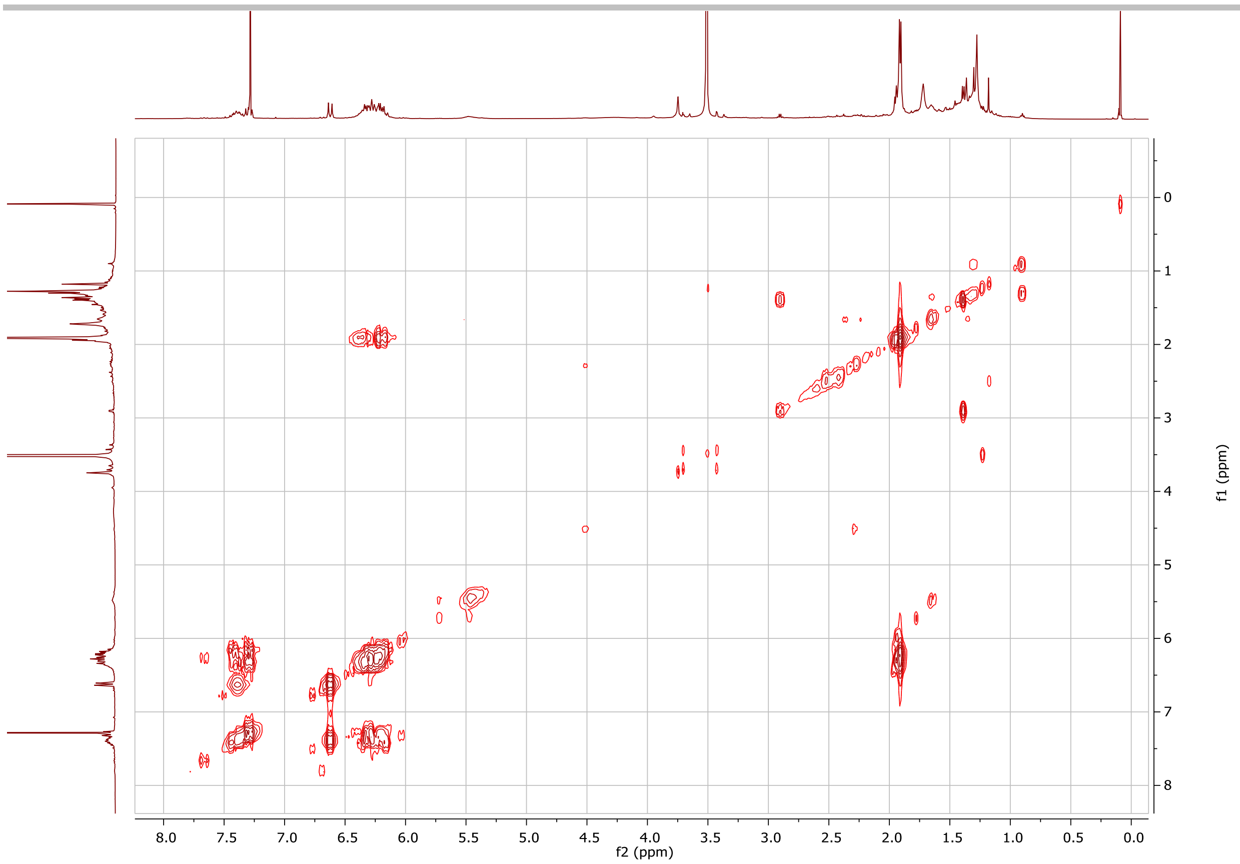

Figure S40: ^{1}H-^{1}H COSY spectrum for **3a** recorded at 500 MHz in $CDCl_3$.

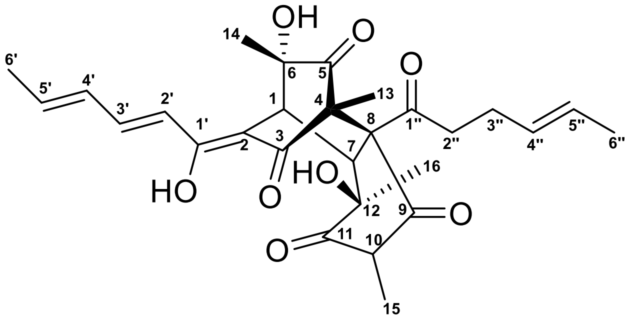

bisvertinoquinol **3b**

Chemical Formula: $C_{28}H_{34}O_8$
Exact Mass: 498,2254

UV$_{\lambda max}$ (MeOH): 266 nm, 295 nm, 382 nm
HRMS (ESI) *m/z* (*M*-H)$^-$ calcd for $C_{28}H_{33}O_8$: 497.2175, found: 497.2155

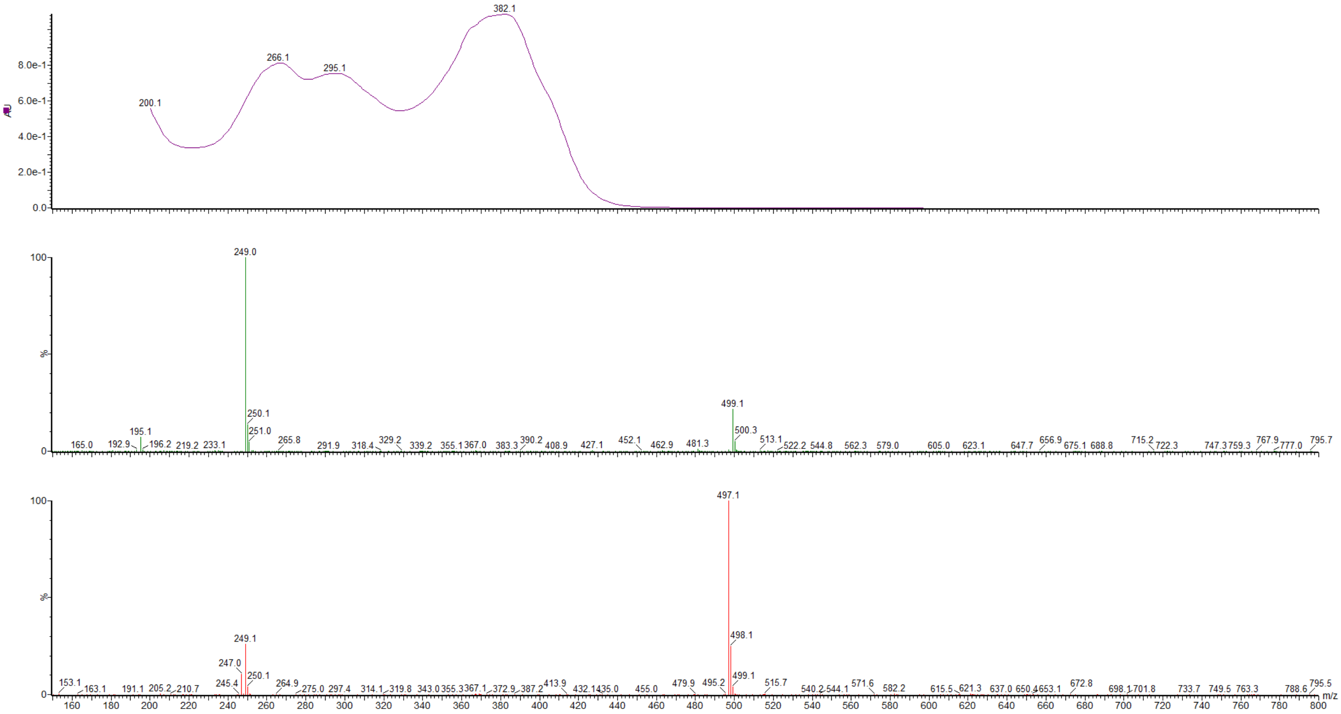

Figure S41: UV-absorption (top) and fragmentation pattern of **3b** in ES+ TIC (middle) and ES- TIC (bottom).

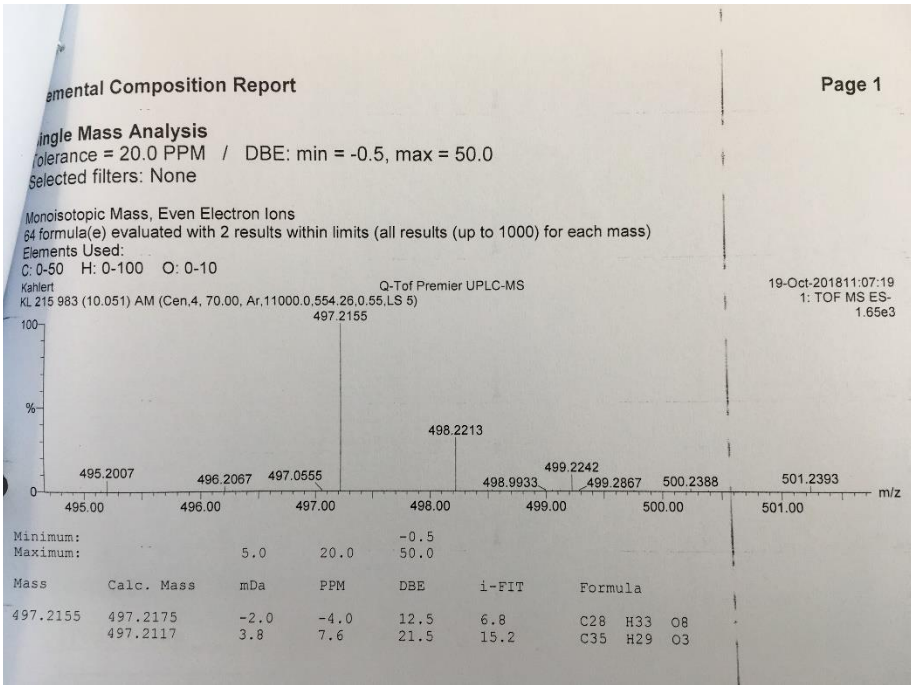

Figure S42: HRMS data for **3b**.

Table S8: Summarized NMR data for **3b** recorded in $CDCl_3$.

Position	δ_C	δ_H	M	J_{H-H}/Hz	HSQC	HMBC H to C	H-H COSY
1	41.2	3.72	d	3.4	CH	2, 5, 6, 14, 1‘	7
2	108.9	-	-	-	-	-	-
3	194.3	-	-	-	-	-	-
4	64.4	-	-	-	-	-	-
5	203.2	-	-	-	-	-	-
6	73.2	-	-	-	-	-	-
7	47.0	3.49	d	3.4	CH	1, 2, 8 11, 12, 16, 1“	1
8	71.7	-	-	-	-	-	-
9	202.8	-	-	-	-	-	-
10	53.7	2.95	q	3.4	CH	9, 11, 12, 15,	15
11	209.2	-	-	-	-	-	-
12	77.4	-	-	-	-	-	-
13	9.4	1.21	s	-	CH_3	3, 4, 5, 8,	-
14	24.6	1.30	s	-	CH_3	1, 5, 6	-
15	13.4	1.36	d	7.1	CH_3	9, 10, 11	10
16	26.9	1.41	s	-	CH_3	7, 11, 12	-
1‘	173.6	-	-	-	-	-	-
2‘	117.9	6.21	d	6.8	CH	1‘, 4‘, 5‘	3‘
3‘	145.7	7.44	dd	10.0, 15.0	CH	1‘, 4‘, 5‘, 6‘	2‘
4‘	142.6	7.30	dd	10.9, 14.9	CH	3‘, 5‘	5‘
5‘	131.1	6.31	m	-	CH	2‘, 4‘, 6‘	3‘, 4‘, 6‘
6‘	19.1	1.91	d	6.6	CH_3	4‘, 5‘	5‘
1“	205.7	-	-	-	-	-	-
2“	40.3	2.65	m	-	CH_2	1“, 3“, 4“	3“
3“	27.5	2.32	m	-	CH_2	1“, 4“, 5“	2“, 4“
4“	129.4	5.37	m	-	CH	2“, 3“, 5“, 6“	3“
5“	126.6	5.47	m	-	CH	3“, 5“, 6“	6“
6“	18.0	1.64	d	6.8	CH_3	4“, 5“	5“

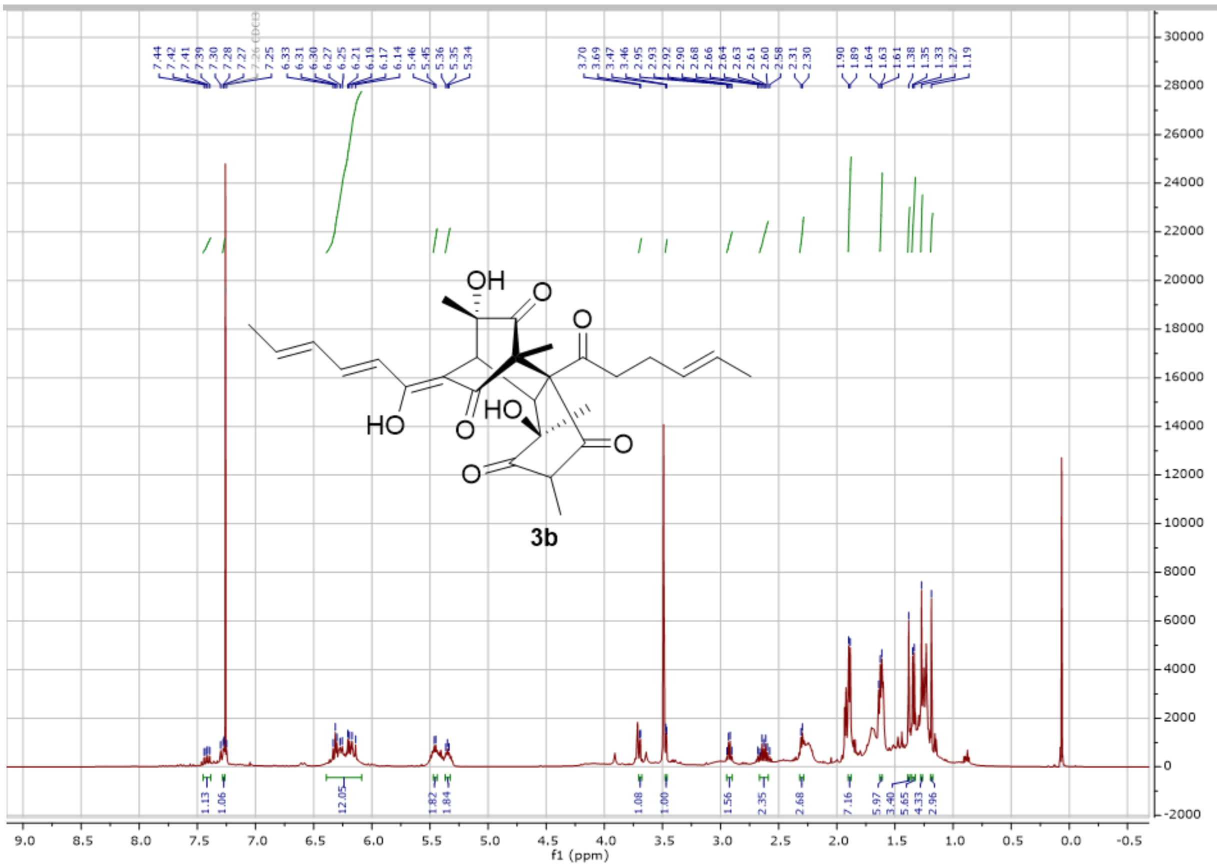

Figure S43: ^{1}H-NMR spectrum for **3b** recorded at 500 MHz in $CDCl_3$.

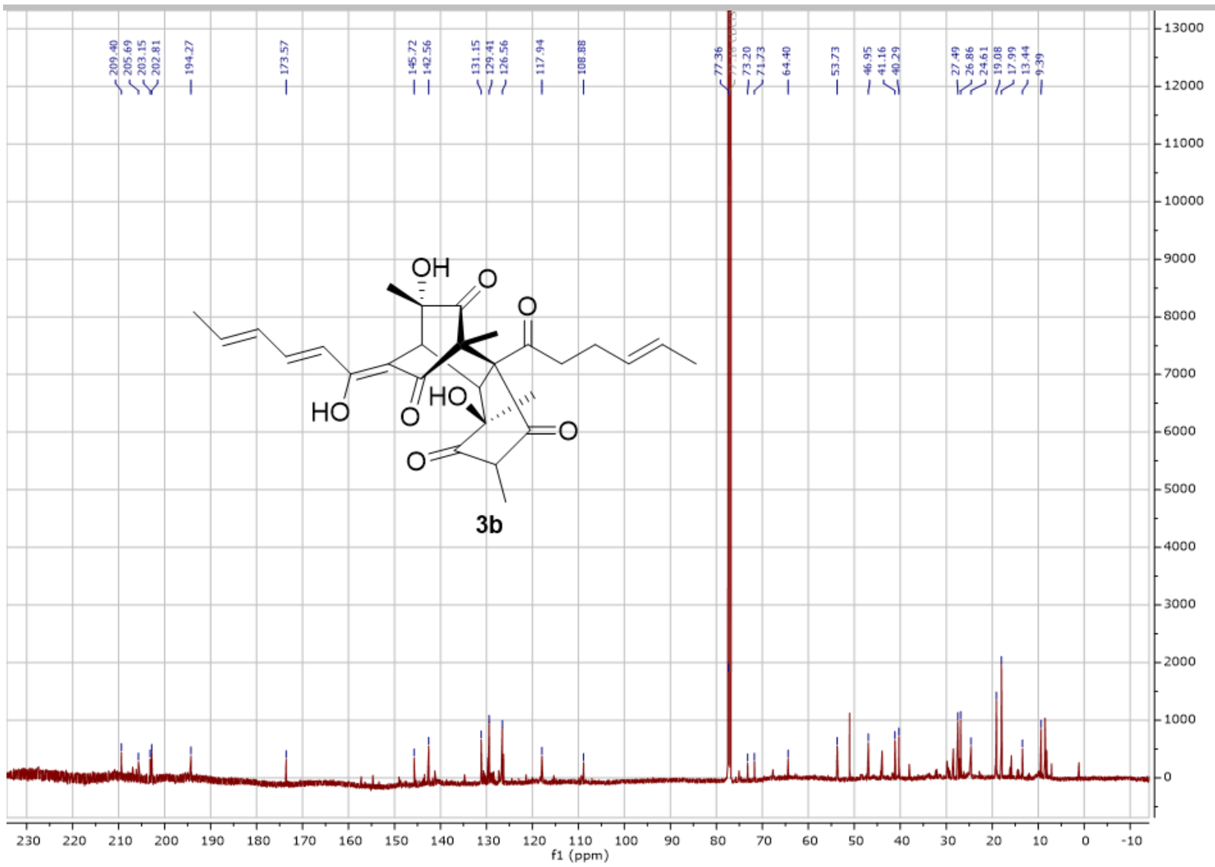

Figure S44: ^{13}C-NMR spectrum for **3b** recorded at 125 MHz in $CDCl_3$.

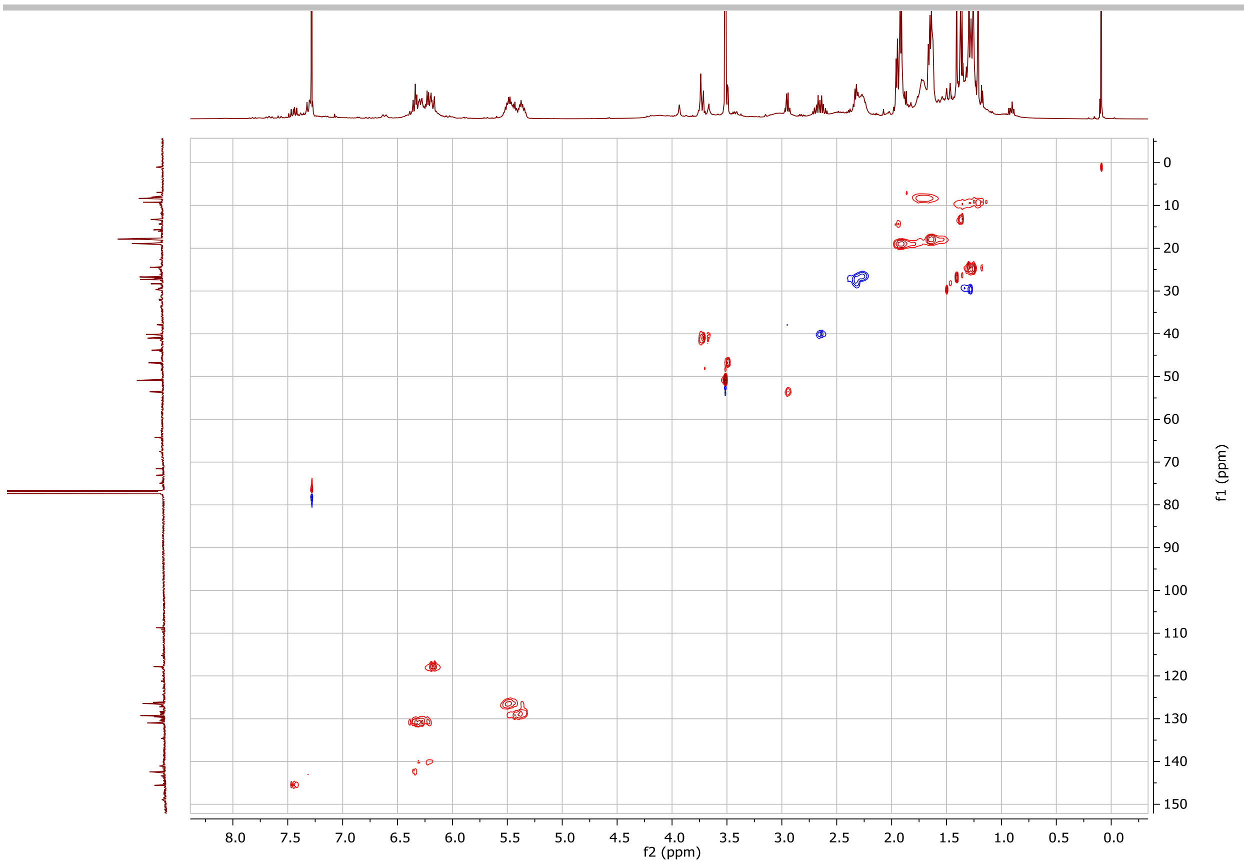

Figure S45: HSQC spectrum for **3b** recorded at 500 MHz in $CDCl_3$.

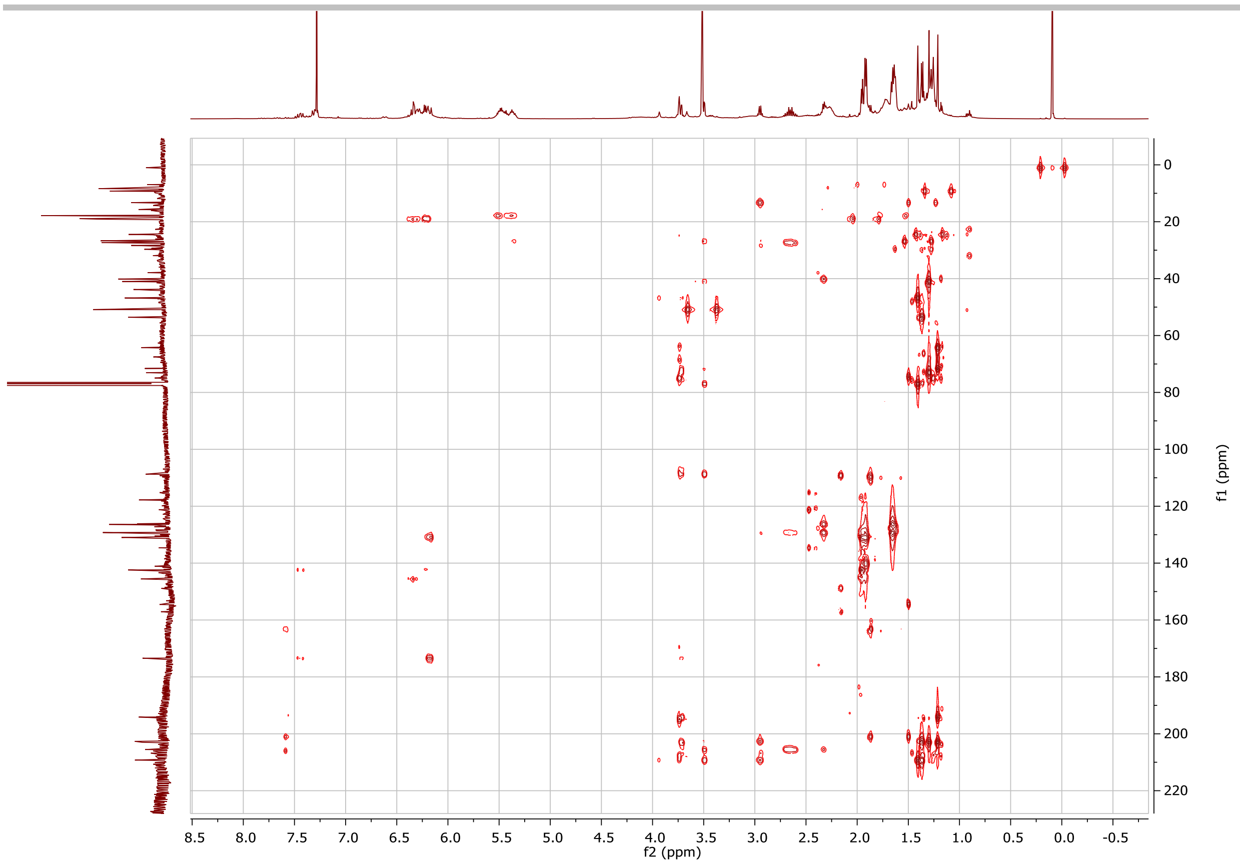

Figure S46: HMBC spectrum for **3b** recorded at 500 MHz in $CDCl_3$.

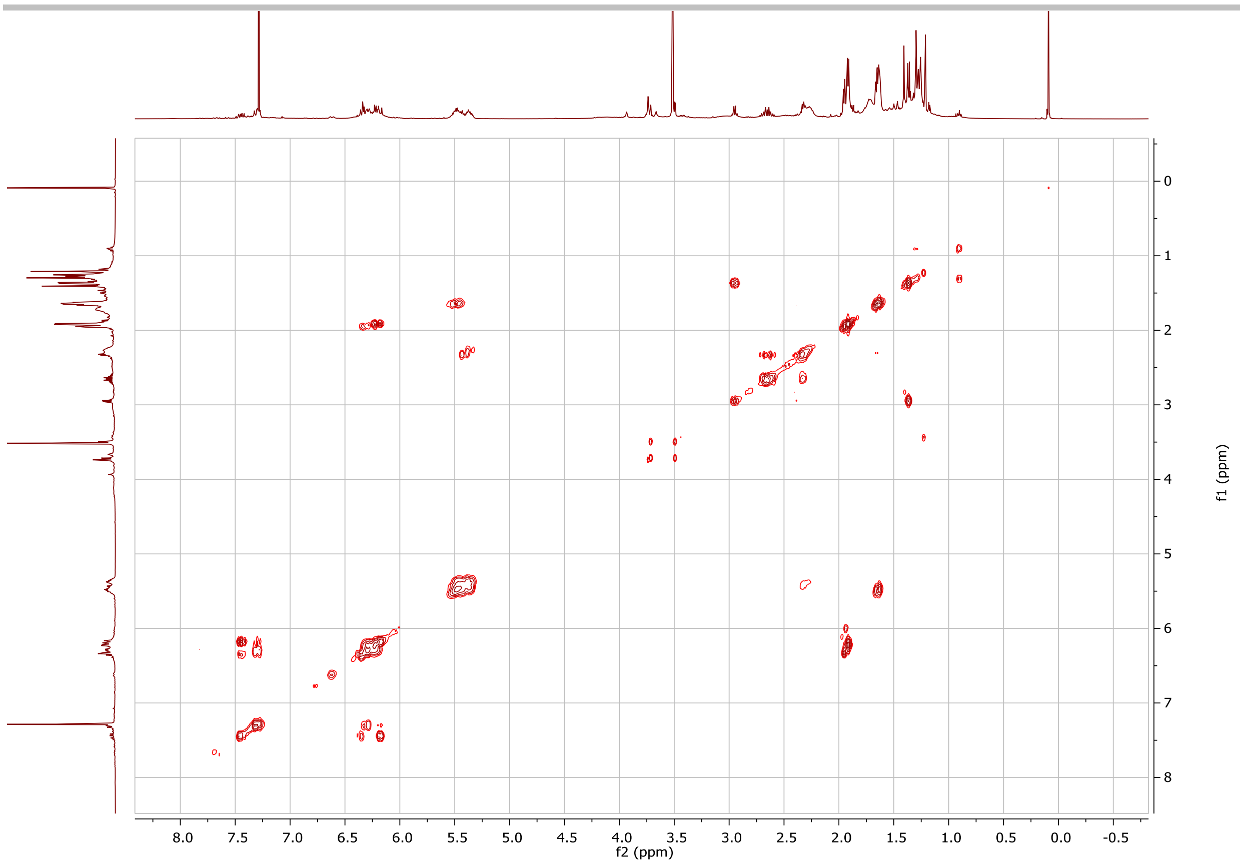

Figure S47: ^{1}H-^{1}H COSY spectrum for **3b** recorded at 500 MHz in $CDCl_3$.

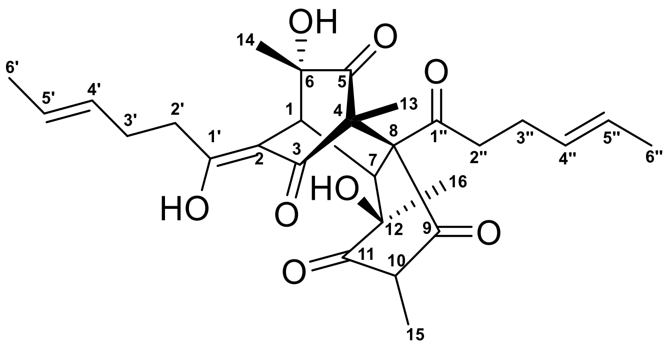

tetradihydrobisorbicillinol **3c**

Chemical Formula: $C_{28}H_{36}O_8$
Exact Mass: 500,2410

UV$_{\lambda max}$ (MeOH): 222 nm, 271 nm, 383 nm
HRMS (ESI) *m/z* (*M*-H)$^-$ calcd for $C_{28}H_{35}O_8$: 499.2332, found: 499.2337

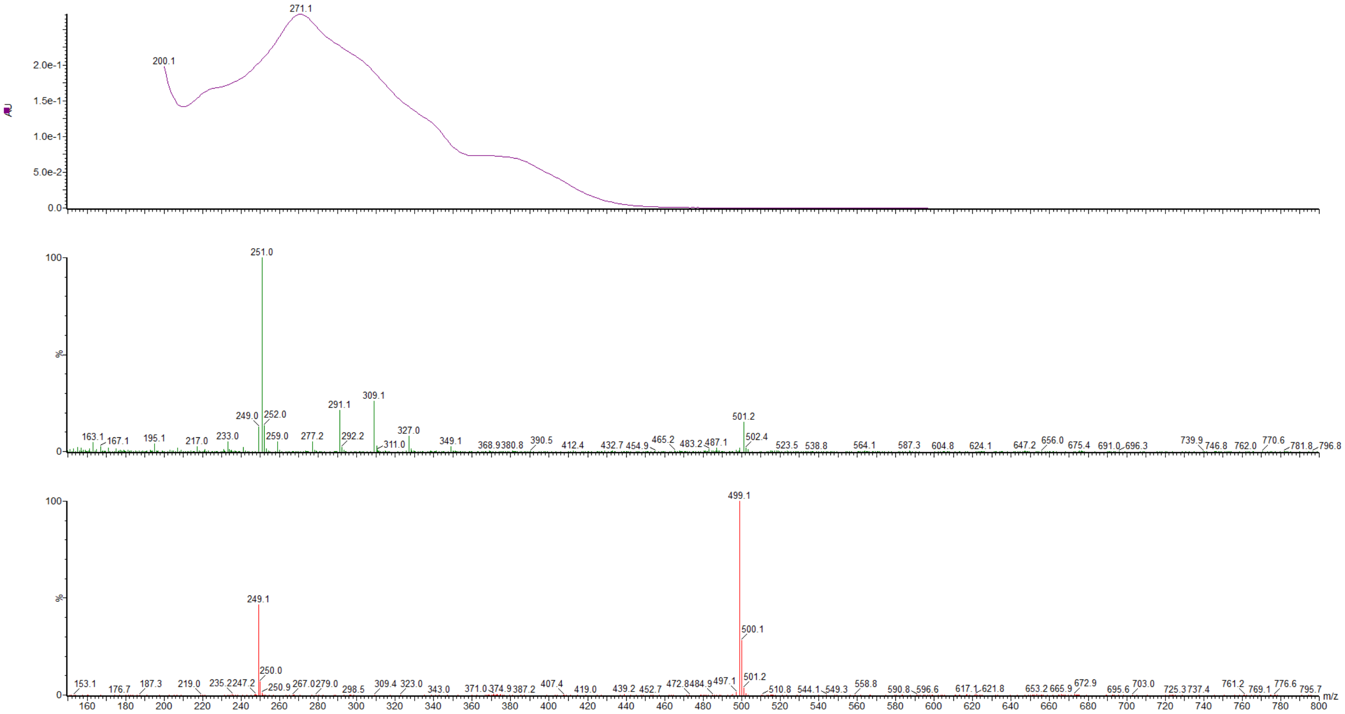

Figure S48: UV-absorption (top) and fragmentation pattern of **3c** in ES+ TIC (middle) and ES- TIC (bottom).

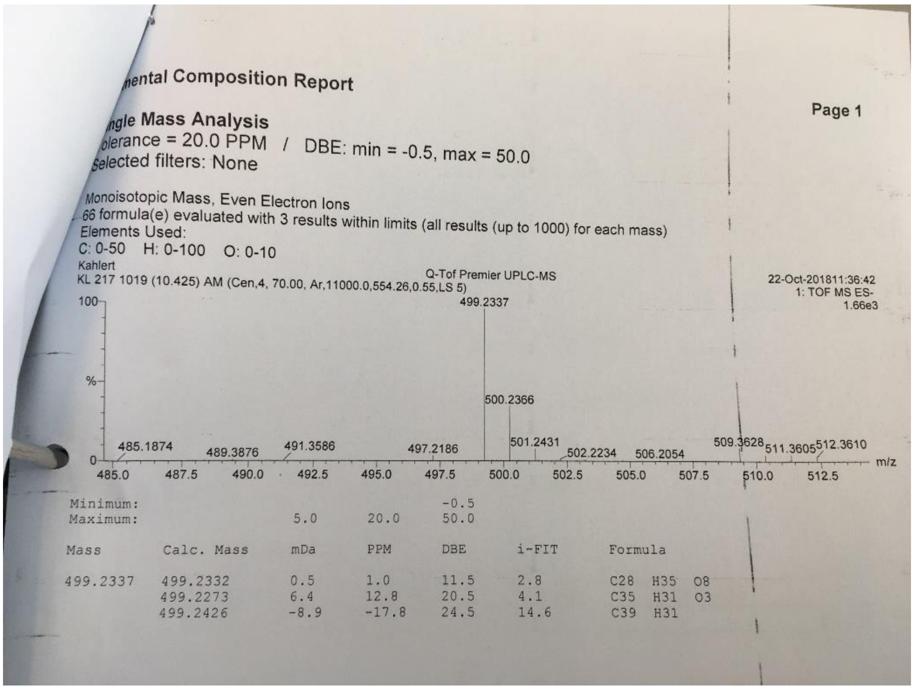

ental Composition Report

Page 1

ngle Mass Analysis
olerance = 20.0 PPM / DBE: min = -0.5, max = 50.0
Selected filters: None

Monoisotopic Mass, Even Electron Ions
66 formula(e) evaluated with 3 results within limits (all results (up to 1000) for each mass)
Elements Used:
C: 0-50 H: 0-100 O: 0-10
Kahlert
KL 217 1019 (10.425) AM (Cen,4, 70.00, Ar,11000.0,554.26,0.55,LS 5) Q-Tof Premier UPLC-MS 22-Oct-201811:36:42
1: TOF MS ES-
1.66e3

Minimum:				-0.5		
Maximum:		5.0	20.0	50.0		
Mass	Calc. Mass	mDa	PPM	DBE	i-FIT	Formula
499.2337	499.2332	0.5	1.0	11.5	2.8	C28 H35 O8
	499.2273	6.4	12.8	20.5	4.1	C35 H31 O3
	499.2426	-8.9	-17.8	24.5	14.6	C39 H31

Figure S49: HRMS data for **3c**.

Table S9: Summarized NMR data for **3c** recorded in $CDCl_3$.

Position	δ_C	δ_H	M	J_{H-H}/Hz	HSQC	HMBC H to C	H-H COSY
1	41.5	3.66	d	3.2	CH	2, 3, 5, 6, 8 (14)	7
2	109.8	-	-	-	-	-	-
3	190.3	-	-	-	-	-	-
4	63.2	-	-	-	-	-	-
5	202.7	-	-	-	-	-	-
6	72.4	-	-	-	-	-	-
7	47.3	3.44	d	3.1	CH	2, 5, 9, 11, 12, 16, 1‘‘	1
8	71.5	-	-	-	-	-	-
9	202.7	-	-	-	-	-	-
10	53.6	2.87	q (buried)	12.4	CH	9, 11, 15	15
11	209.9	-	-	-	-	-	-
12	77.0	-	-	-	-	-	-
13	9.2	1.18	s	-	CH_3	3, 4, 5, 8, 14,	-
14	25.1	1.29	s	-	CH_3	1, 5, 6	-
15	13.4	1.36	d	1.4	CH_3	8, 9, 11, 12	10
16	27.1	1.38	s	-	CH_3	4, 7, 11, 12	-
1‘	181.9	-	-	-	-		-
2‘	42.7	2.67	m	-	CH_2		3‘, 4‘
3‘	26.8	2.28	m	-	CH_2	4‘, 5‘	4‘
4‘ = 4‘‘	129.7	5.42	m	-	CH	6‘	3‘, 5‘, 6‘
5‘ = 5‘‘	126.3	5.51	m	-	CH	6‘	3‘, 4‘, 6‘
6‘ = 6‘‘	18.1	1.64	m	-	CH_3	4‘, 5‘	4‘, 5‘
1‘‘	205.7	-	-	-	-	-	-
2‘‘	38.1	2.92	m	-	CH_2	1‘‘, 3‘‘, 4‘‘	3‘‘
3‘‘	27.1	2.37	m	-	CH_2	1‘‘, 4‘‘, 5‘‘	2‘‘, 4‘‘, 5‘‘

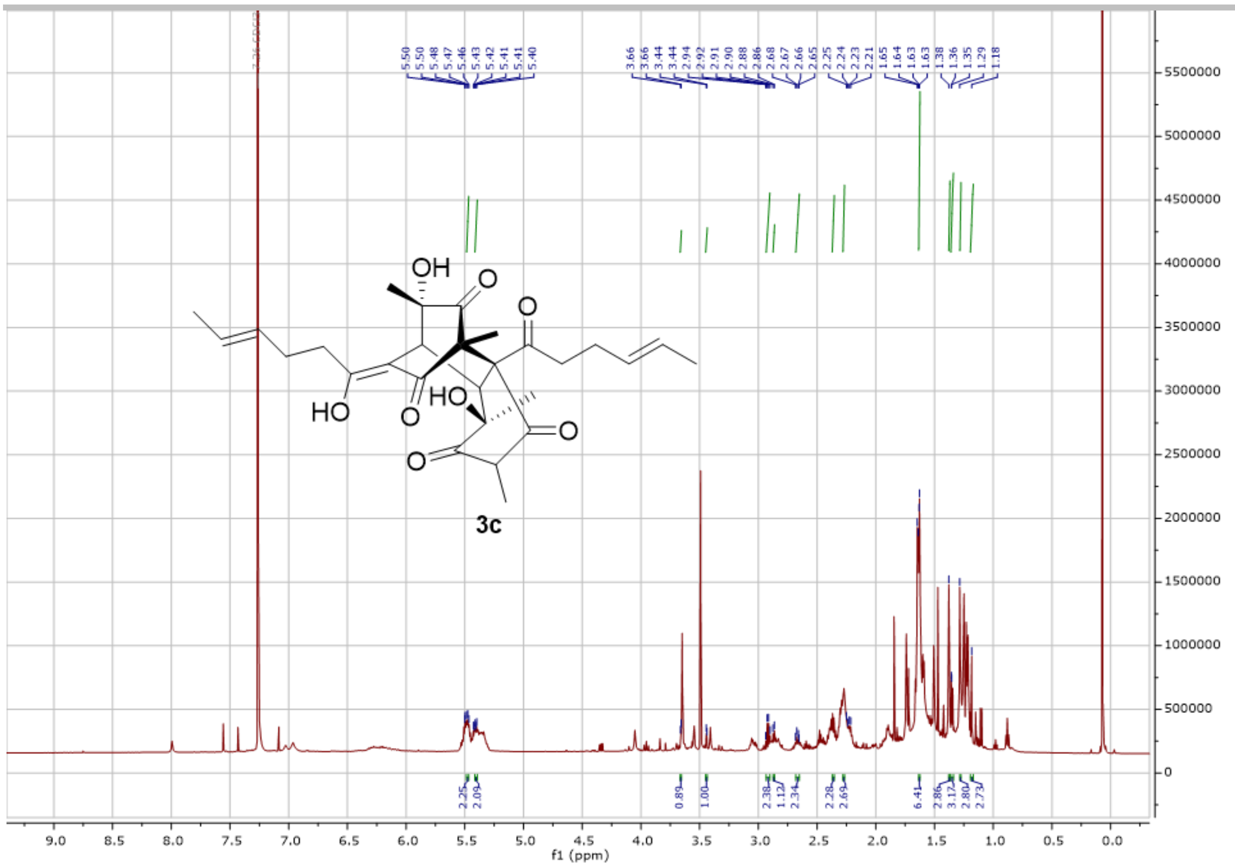

Figure S50: ^{1}H-NMR spectrum for **3c** recorded at 600 MHz in $CDCl_3$.

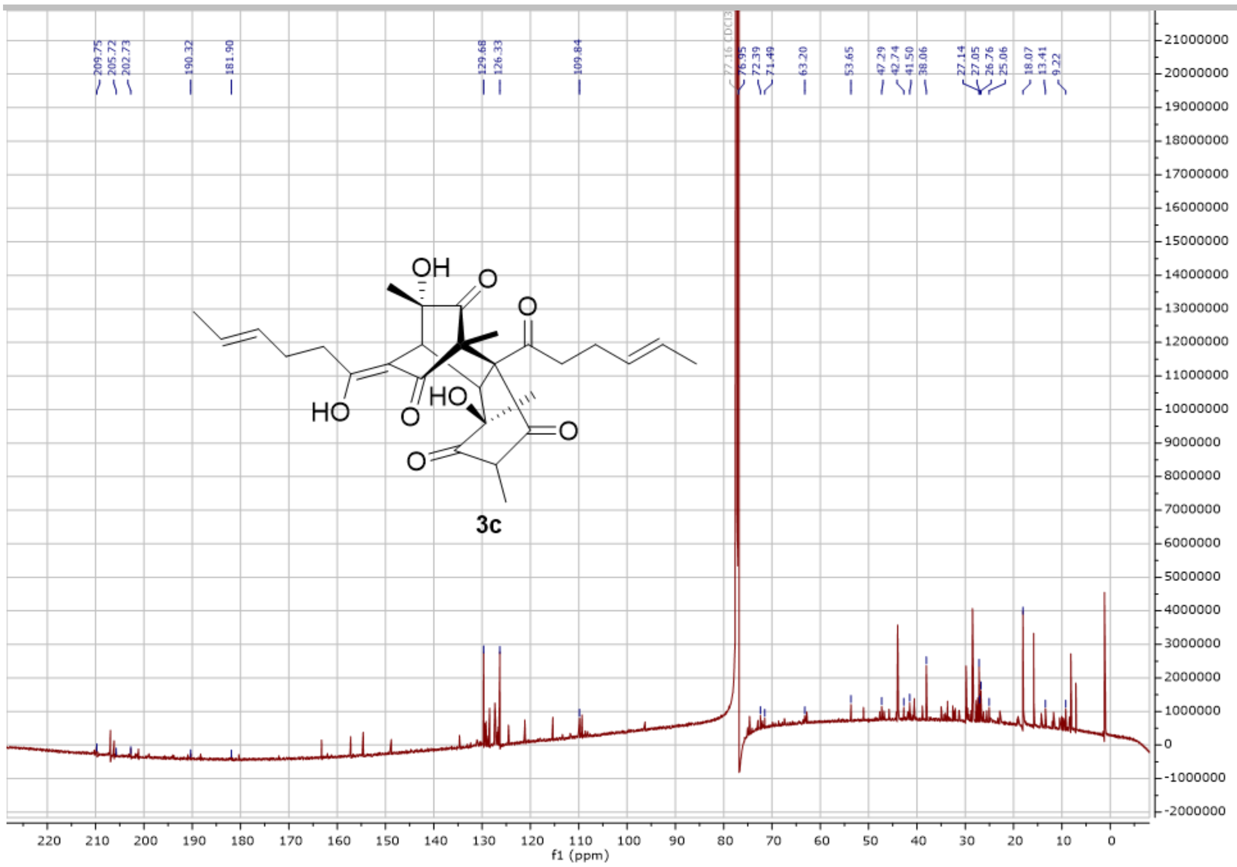

Figure S51: ^{13}C-NMR spectrum for **3c** recorded at 125 MHz in $CDCl_3$.

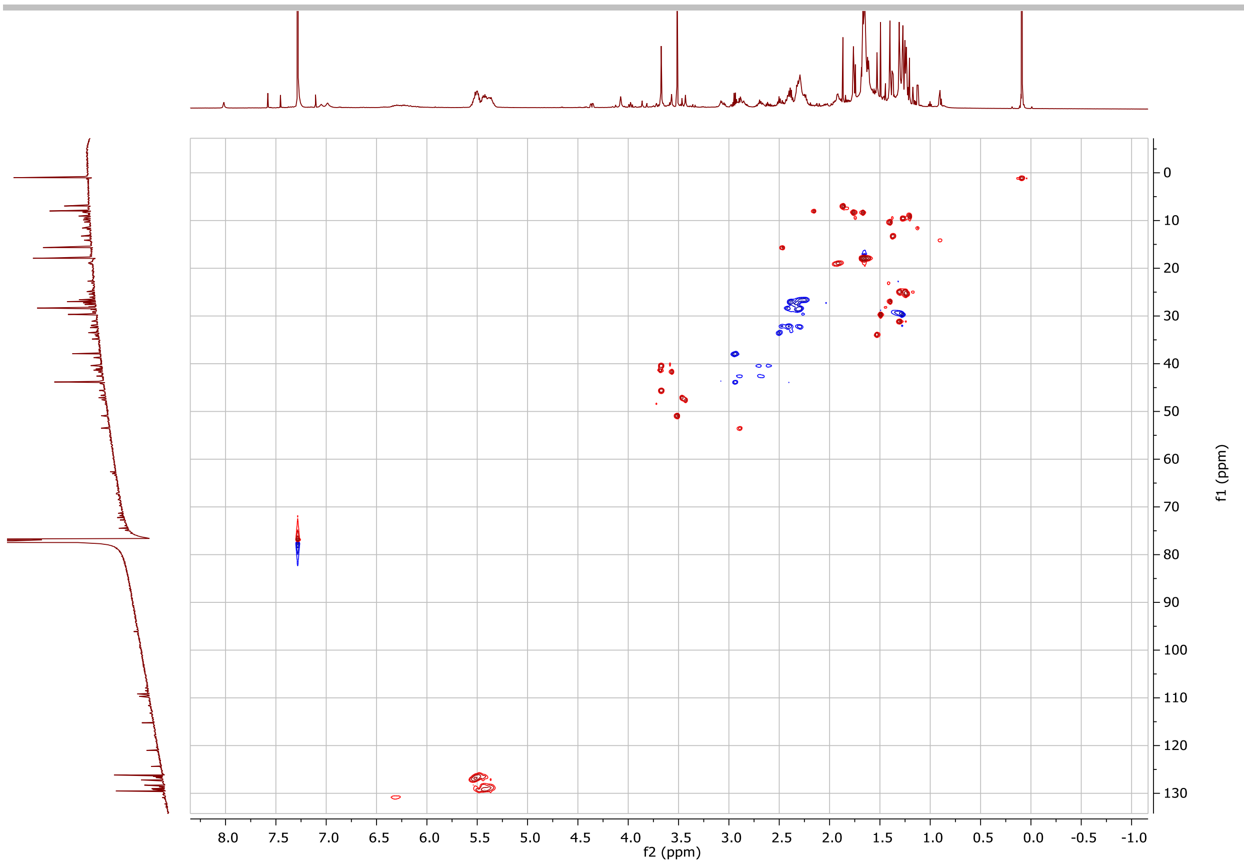

Figure S52: HSQC spectrum for **3c** recorded at 600 MHz in $CDCl_3$.

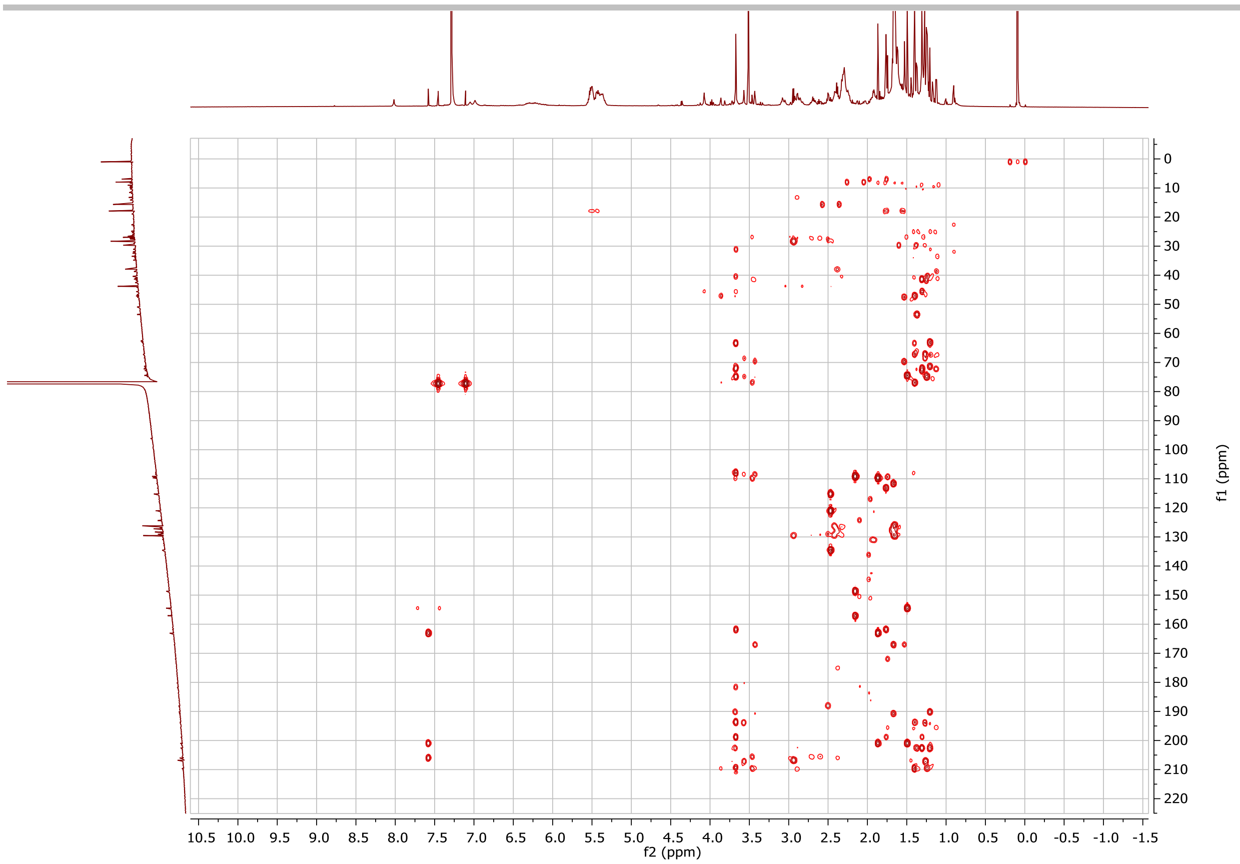

Figure S53: HMBC spectrum for **3c** recorded at 600 MHz in $CDCl_3$.

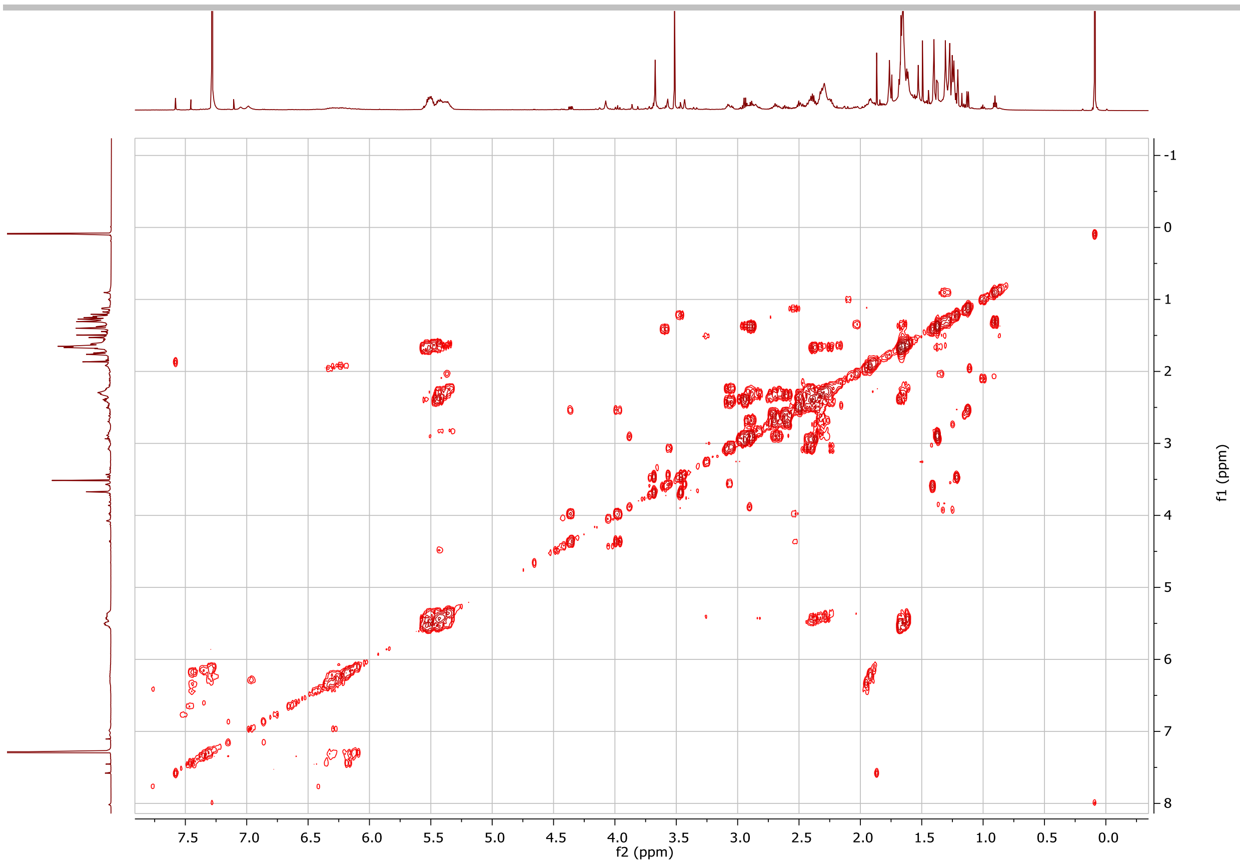

Figure S54: ^{1}H-^{1}H COSY spectrum for **3c** recorded at 600 MHz in $CDCl_3$.

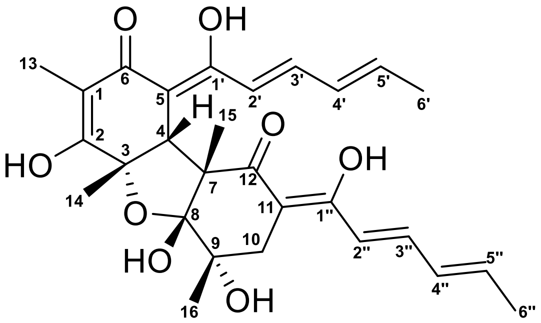

bisvertinol **4a**

Chemical Formula: $C_{28}H_{34}O_8$
Exact Mass: 498,2254

UV$_{\lambda max}$ (MeOH): 202 nm, 223 nm, 272 nm, 304 nm, 404 nm
HRMS (ESI) *m/z* (*M*-H)$^-$ calcd for $C_{28}H_{33}O_8$: 497.2175, found: 497.2174

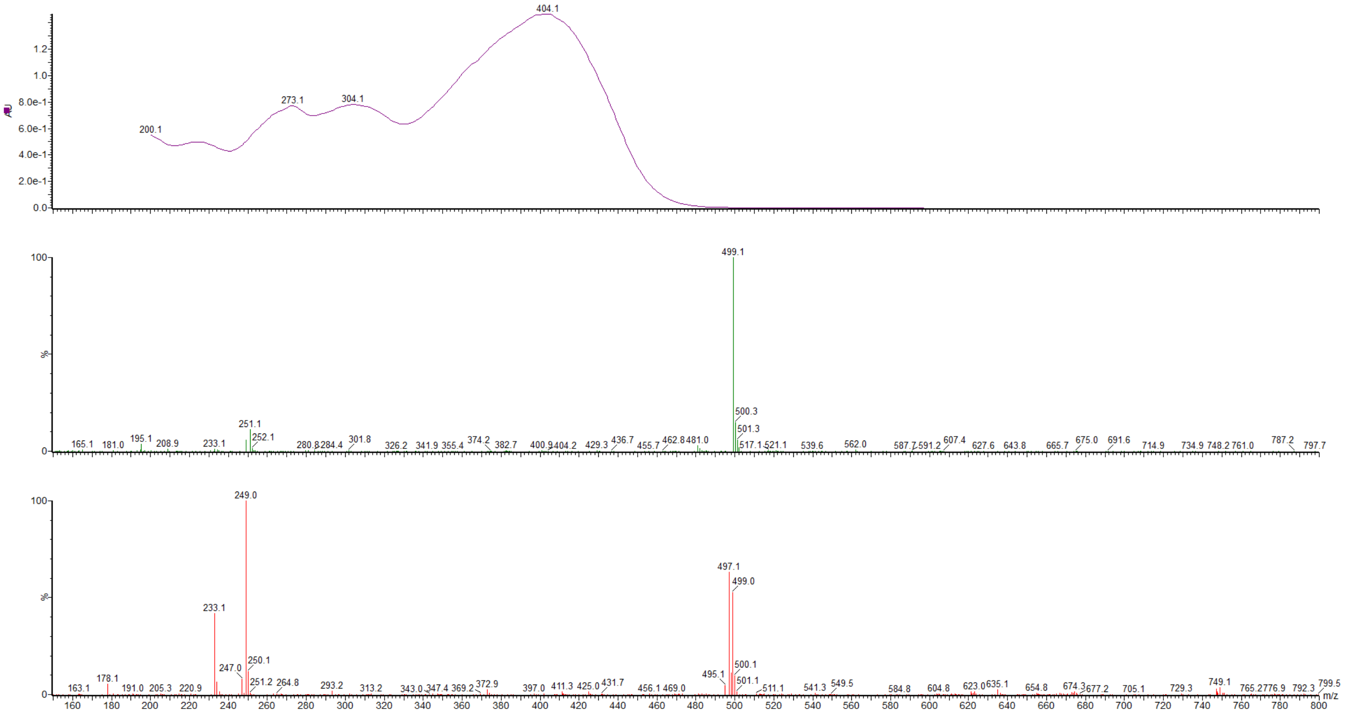

Figure S55: UV-absorption (top) and fragmentation pattern of **4a** in ES+ TIC (middle) and ES- TIC (bottom).

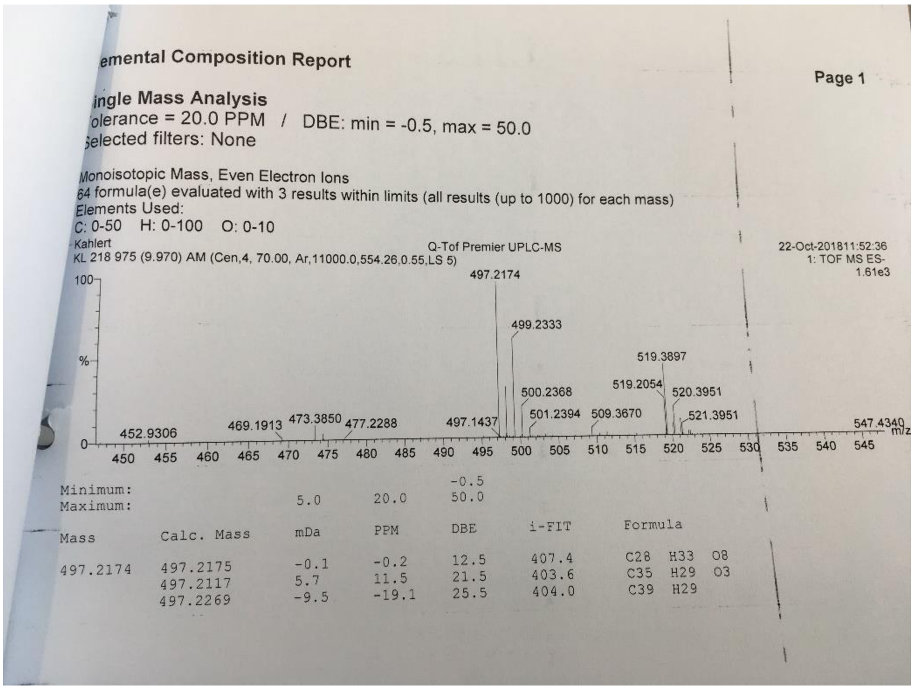

ementental Composition Report

Page 1

ingle Mass Analysis
'olerance = 20.0 PPM / DBE: min = -0.5, max = 50.0
;elected filters: None

Monoisotopic Mass, Even Electron Ions
64 formula(e) evaluated with 3 results within limits (all results (up to 1000) for each mass)
Elements Used:
C: 0-50 H: 0-100 O: 0-10
Kahlert
KL 218 975 (9.970) AM (Cen,4, 70.00, Ar,11000.0,554.26,0.55,LS 5) Q-Tof Premier UPLC-MS 22-Oct-201811:52:36
1: TOF MS ES-
1.61e3

Minimum:				-0.5		
Maximum:		5.0	20.0	50.0		
Mass	Calc. Mass	mDa	PPM	DBE	i-FIT	Formula
497.2174	497.2175	-0.1	-0.2	12.5	407.4	C28 H33 O8
	497.2117	5.7	11.5	21.5	403.6	C35 H29 O3
	497.2269	-9.5	-19.1	25.5	404.0	C39 H29

Figure S56: HRMS data for **4a**.

Table S10: Summarized NMR data for **4a** recorded in $CDCl_3$.

Position	δ_C	δ_H	M	J_{H-H}/Hz	HSQC	HMBC H to C	H-H COSY
1	110.5	-	-	-	-	-	-
2	162.7	-	-	-	-	-	-
3	79.7	-	-	-	-	-	-
4	53.4	3.62	s	-	CH	2, 3, 6, 7, 13, 14, 1‘	-
5	100.5	-	-	-	-	-	-
6	191.6	-	-	-	-	-	-
7	53.4	-	-	-	-	-	-
8	106.2	-	-	-	-	-	-
9	73.8	-	-	-	-	-	-
10	36.7	2.60	s	-	CH_2	8, 9, 11, 12, 16, 1“	-
11	103.7	-	-	-	-	-	-
12	191.6	-	-	-	-	-	-
13	6.9	1.52	s	-	CH_3	1, 2, 6	-
14	25.7	1.47	s	-	CH_3	2, 3, 4, 7,	-
15	19.2	1.33	s	-	CH_3	4, 7, 8, 12	-
16	22.3	1.27	s	-	CH_3	8, 9, 10	-
1‘	169.2	-	-	-	-	-	-
2‘	120.4	6.38	d	15.6	CH	1‘, 4‘	3‘
3‘	139.2	7.29	m	-	CH	1‘, 5“	2‘, 4‘
4‘	131.0	6.28	m	-	CH	2“, 6“	3‘, 5‘
5‘	137.1	6.12	dq	6.8, 13.9	CH	3‘, 6‘	6‘, 4‘
6‘ = 6“	19.1	1.88	m	-	CH_3	4‘, 5‘, 4“, 5“	5‘, 5“, 4‘
1“	180.3	-	-	-	-	-	-
2“	120.4	6.16	d	14.9	CH	1“, 4“	3“
3“	143.1	7.30	m	-	CH	1“, 4“ 5“	2“, 4“
4“	130.7	6.34	m	-	CH	2“, 3“, 6“	3“, 5“
5“	141.3	6.36	m	-	CH	3“, 6“	6“

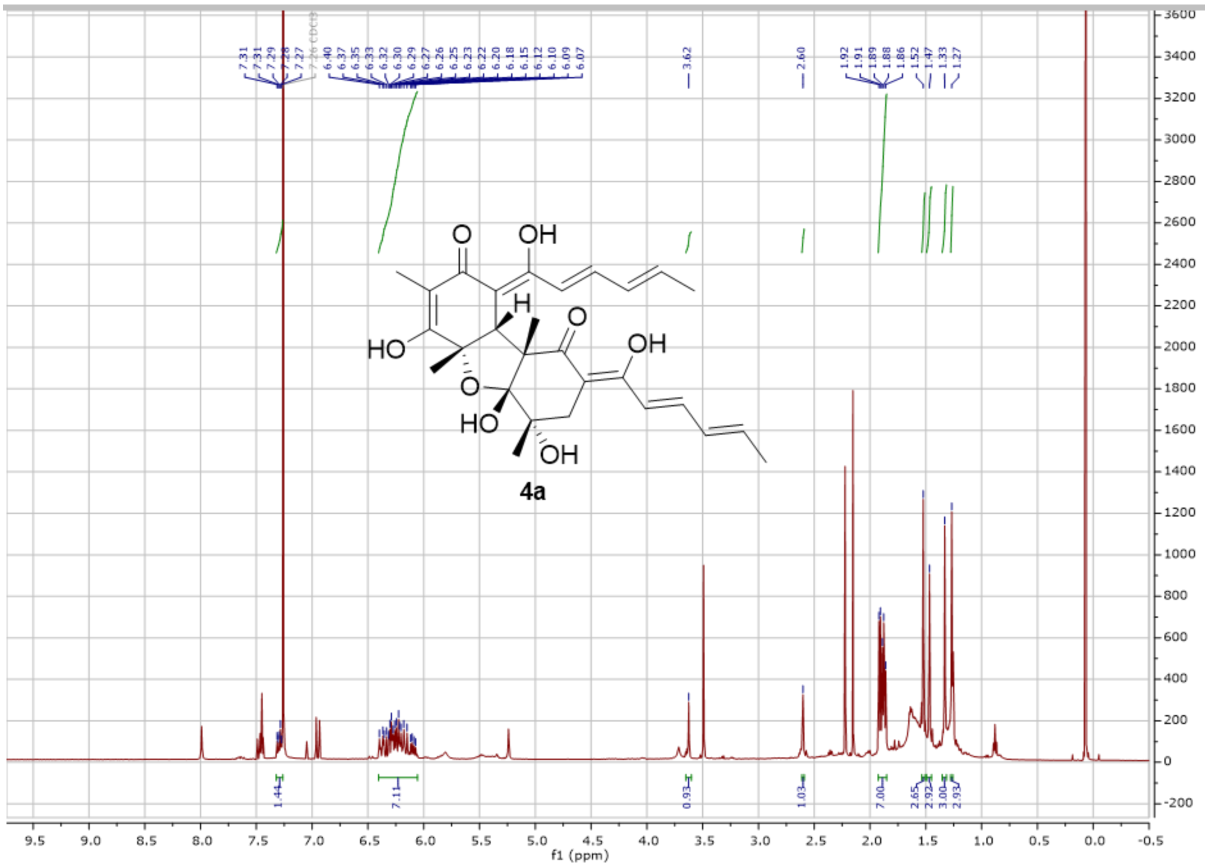

Figure S57: ^{1}H-NMR spectrum for **4a** recorded at 500 MHz in $CDCl_3$.

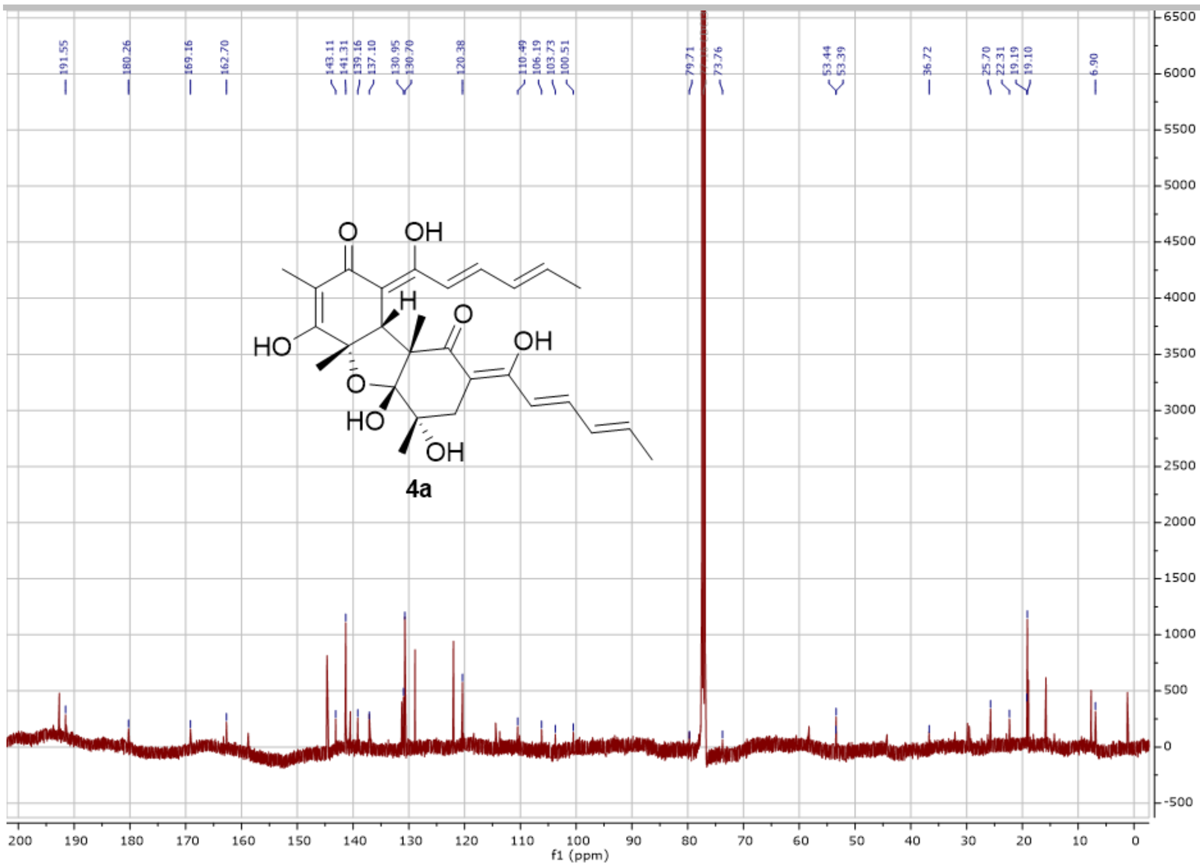

Figure S58: ^{13}C-NMR spectrum for **4a** recorded at 125 MHz in $CDCl_3$.

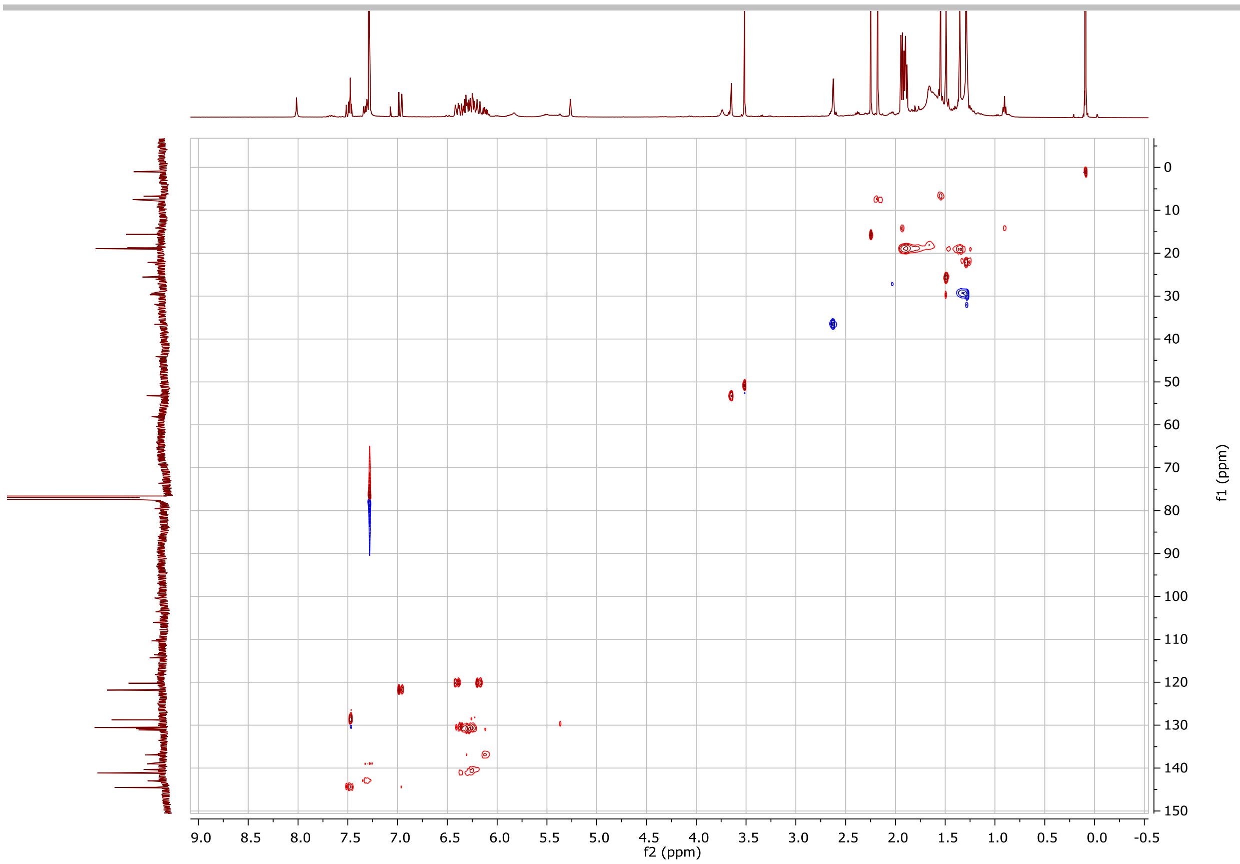

Figure S59: HSQC spectrum for **4a** recorded at 500 MHz in $CDCl_3$.

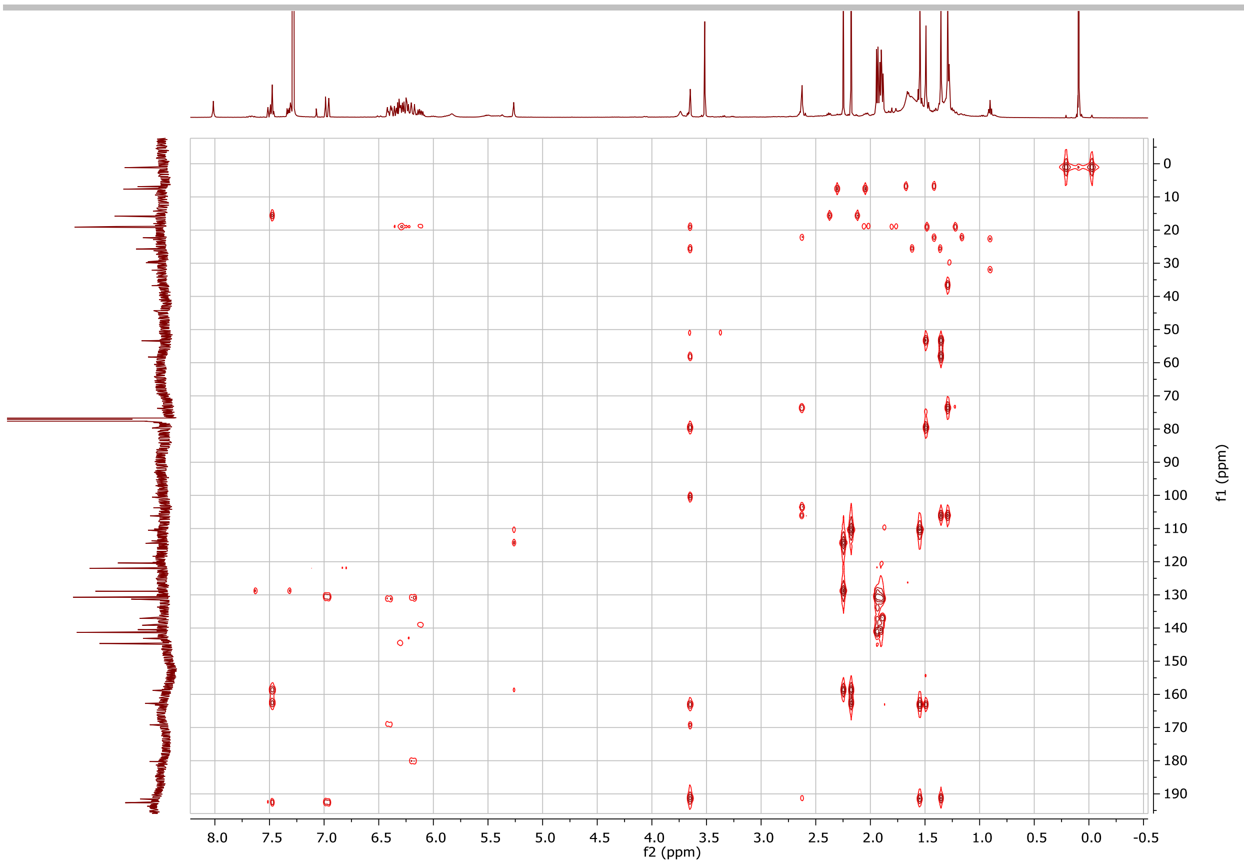

Figure S60: HMBC spectrum for **4a** recorded at 500 MHz in $CDCl_3$.

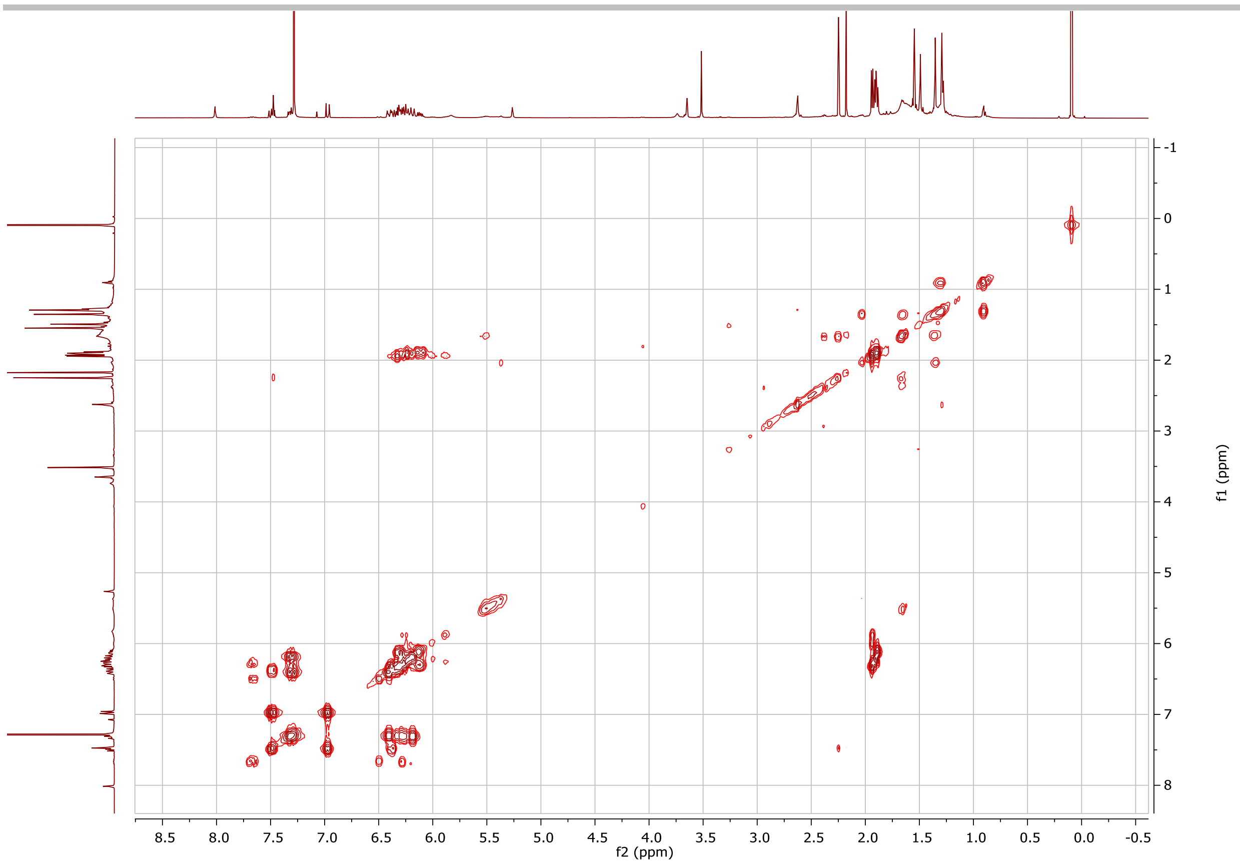

Figure S61: ^{1}H-^{1}H COSY spectrum for **4a** recorded at 500 MHz in $CDCl_3$.

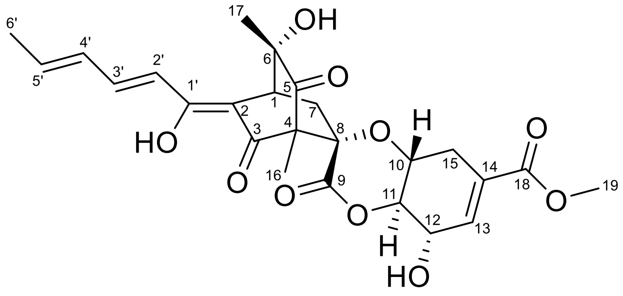

spirosorbicillinol B **6b**

Chemical Formula: $C_{25}H_{28}O_{10}$

Exact Mass: 488,1682

UV$_{\lambda max}$ (MeOH): 361 nm

HRMS (ESI) *m/z* (*M*+H)+ calcd for $C_{25}H_{29}O_{10}$: 489.1761, found: 489.1764

Isomer B was identified by comparing NMR data with the those reported by Hirota *et al.*[9]

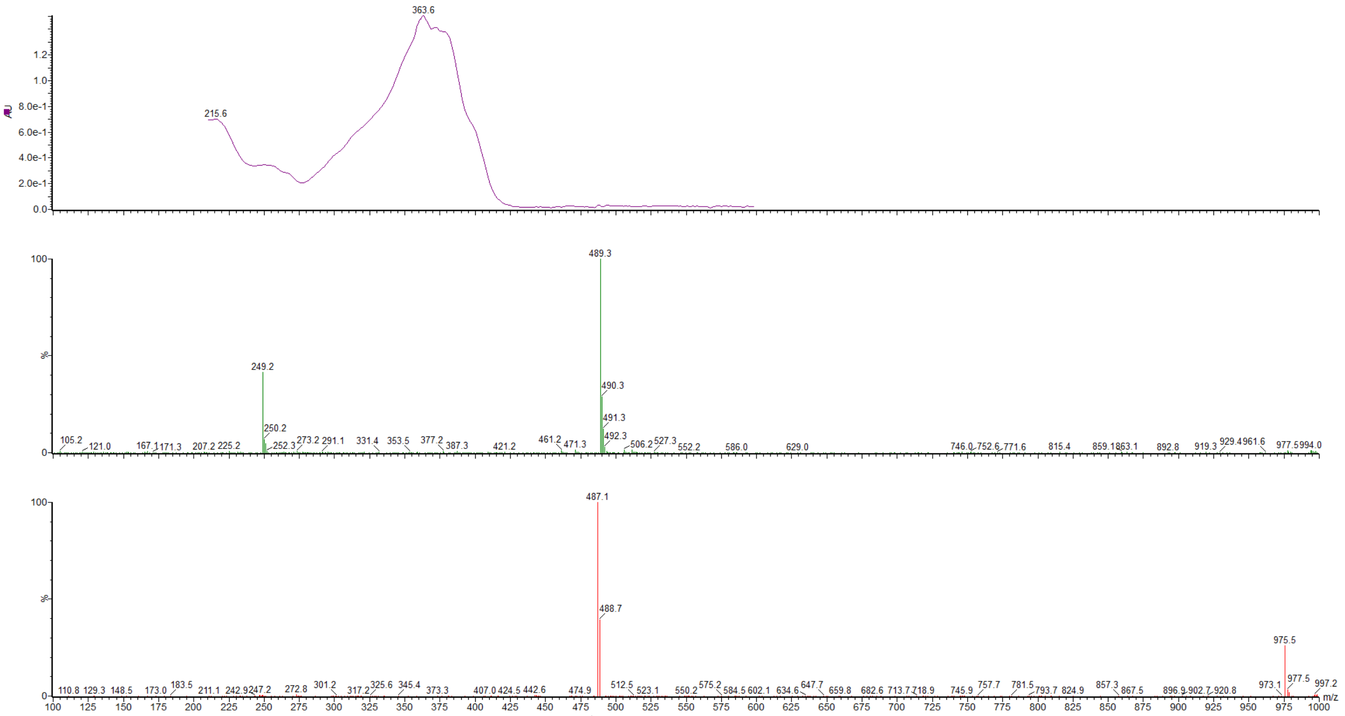

Figure S62: UV-absorption (top) and fragmentation pattern of **6b** in ES+ TIC (middle) and ES- TIC (bottom).

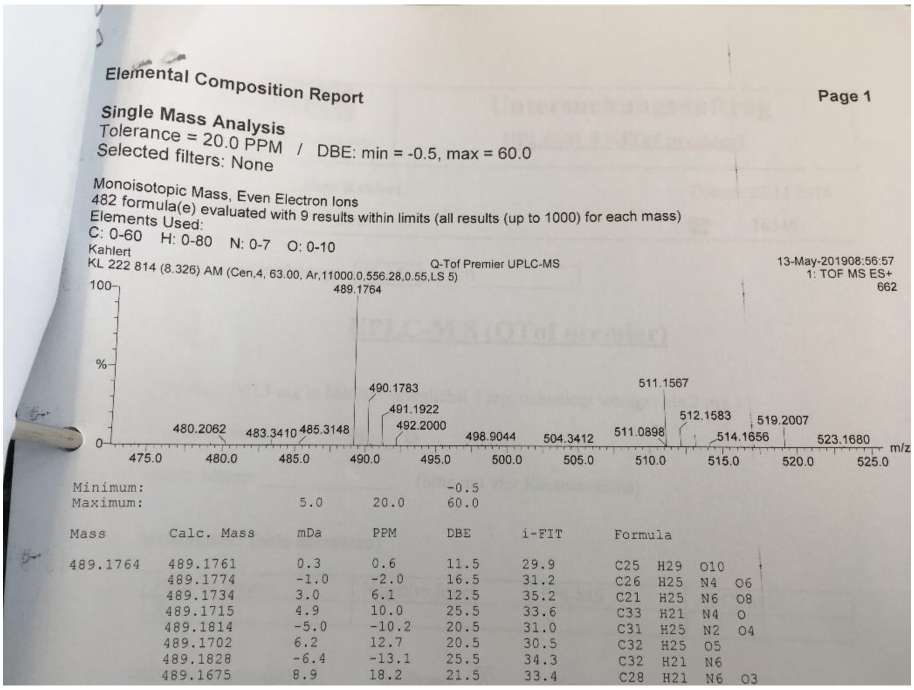

Elemental Composition Report Page 1

Single Mass Analysis
Tolerance = 20.0 PPM / DBE: min = -0.5, max = 60.0
Selected filters: None

Monoisotopic Mass, Even Electron Ions
482 formula(e) evaluated with 9 results within limits (all results (up to 1000) for each mass)
Elements Used:
C: 0-60 H: 0-80 N: 0-7 O: 0-10
Kahlert
KL 222 814 (8.326) AM (Cen,4, 63.00, Ar,11000.0,556.28,0.55,LS 5) Q-Tof Premier UPLC-MS 13-May-201908:56:57 1: TOF MS ES+ 662

Minimum:				-0.5		
Maximum:		5.0	20.0	60.0		
Mass	Calc. Mass	mDa	PPM	DBE	i-FIT	Formula
489.1764	489.1761	0.3	0.6	11.5	29.9	C25 H29 O10
	489.1774	-1.0	-2.0	16.5	31.2	C26 H25 N4 O6
	489.1734	3.0	6.1	12.5	35.2	C21 H25 N6 O8
	489.1715	4.9	10.0	25.5	33.6	C33 H21 N4 O
	489.1814	-5.0	-10.2	20.5	31.0	C31 H25 N2 O4
	489.1702	6.2	12.7	20.5	30.5	C32 H25 O5
	489.1828	-6.4	-13.1	25.5	34.3	C32 H21 N6
	489.1675	8.9	18.2	21.5	33.4	C28 H21 N6 O3

Figure S63: HRMS data for **6b**.

Table S11: Summarized NMR data for **6b** recorded in $CDCl_3$.

Position	δ_C	δ_H	M	J_{H-H}/Hz	HSQC	HMBC H to C	H-H COSY
1	39.9	3.27	t	2.8	CH	2, 3, 4, 5, 6, 7, 8 17, 1'	-
2	109.8	-	-	-	C	-	-
3	193.3	-	-	-	C	-	-
4	69.5	-	-	-	C	-	-
5	205.7	-	-	-	C	-	-
6	74.4	-	-	-	C	-	-
7a	40.0	2.42	dd	2.5, 14.1	CH_2	1, 2, 6, 8, 9	7b
7b	40.0	2.87	dd	3.3, 14.0	CH_2	1, 2, 6, 8, 9	7a
8	81.6	-	-	-	C	-	-
9	165.6 or 167.8	-	-	-	C	-	-
10	70.1	3.98	td	6.4, 9.9	CH	11, 12, 15	11, 15
11	83.2	4.35	dd	7.9, 10.2	CH	12, 15	10, 12
12	70.3	4.55	m	-	CH	11, 13, 14	11, 13
13	136.7	6.73	m	-	CH	11, 14, 15, 18	12, 15
14	128.9	-	-	-	C	-	-
15a	30.8	2.31	m	-	CH_2	10, 11, 18	10
15b	30.8	3.01	dd	6.4, 17.7	CH_2	10, 11, 18	10
16	8.6	1.35	s	-	CH_3	3, 4, 5, 8	-
17	24.4	1.29	s	-	CH_3	1, 5, 6	-
18	165.6	-	-	-	C	-	-
19	52.6	3.78	s	-	CH_3	18	-
1'	165.6 or 167.8	-	-	-	C	-	-
2'	117.8	6.12	d	14.9	CH	2, 1', 3', 4'	3'
3'	143.0	7.33	dd	10.6, 14.9	CH	1', 2', 4', 5'	2', 4'
4'	130.9	6.27	m	-	CH	6', 2'	5', 3'
5'	140.3	6.21	m	-	CH	6', 3'	4', 6'
6'	19.1	1.90	d	6.3	CH_3	4', 5'	5'

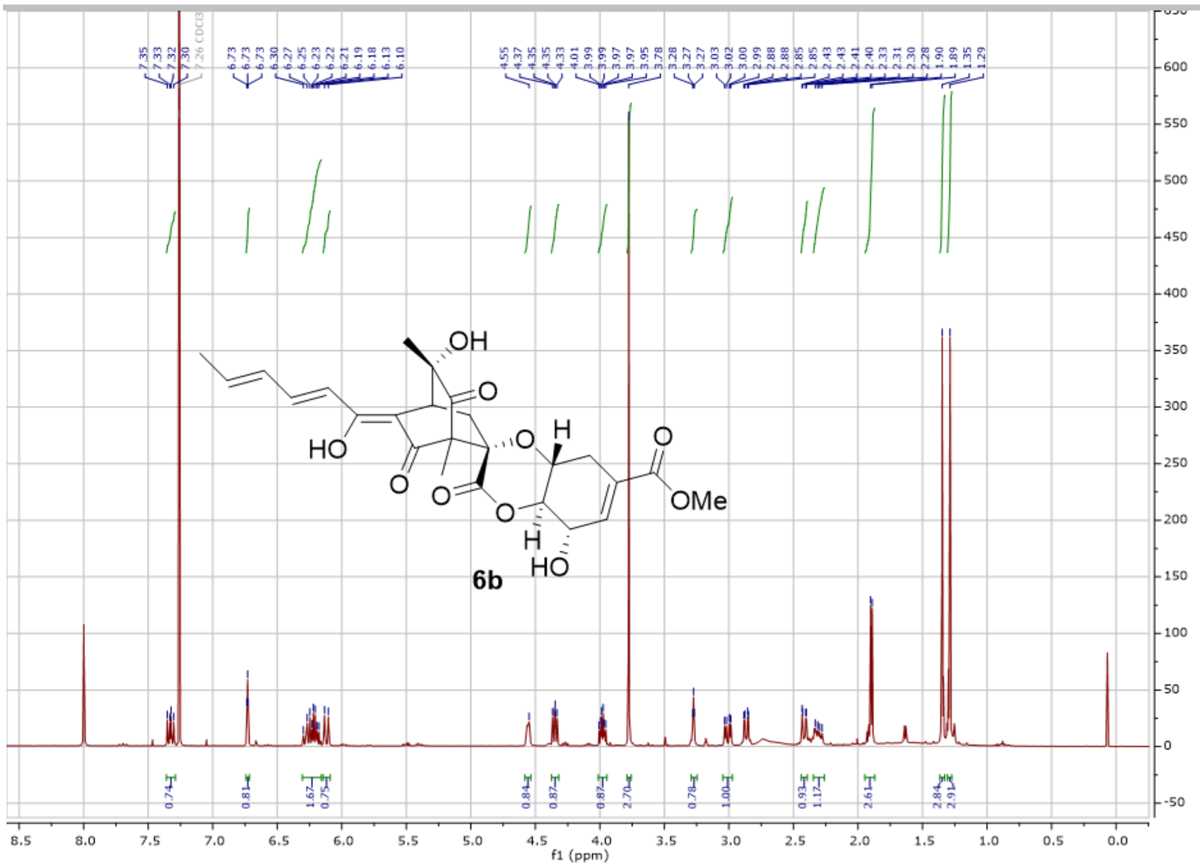

Figure S64: ^{1}H-NMR spectrum for **6b** recorded at 500 MHz in $CDCl_3$.

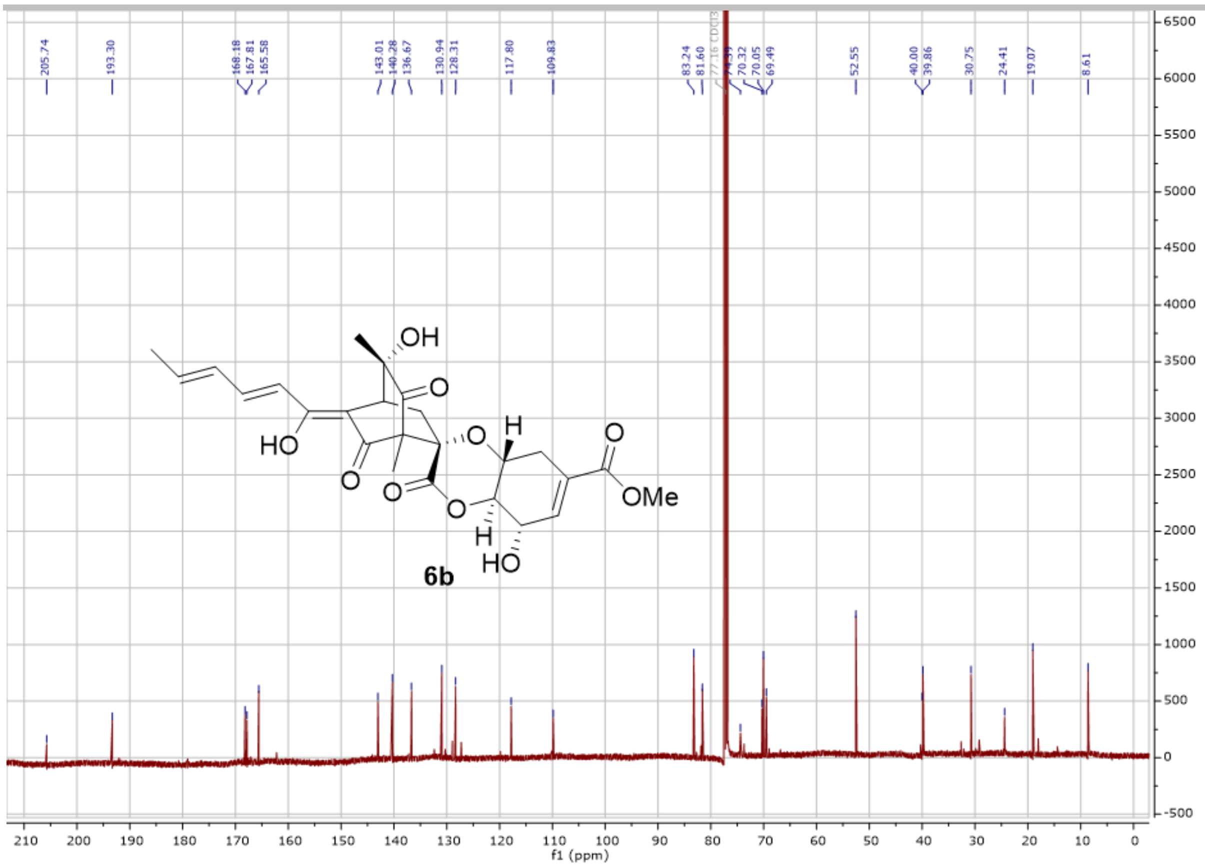

Figure S65: ^{13}C-NMR spectrum for **6b** recorded at 125 MHz in $CDCl_3$.

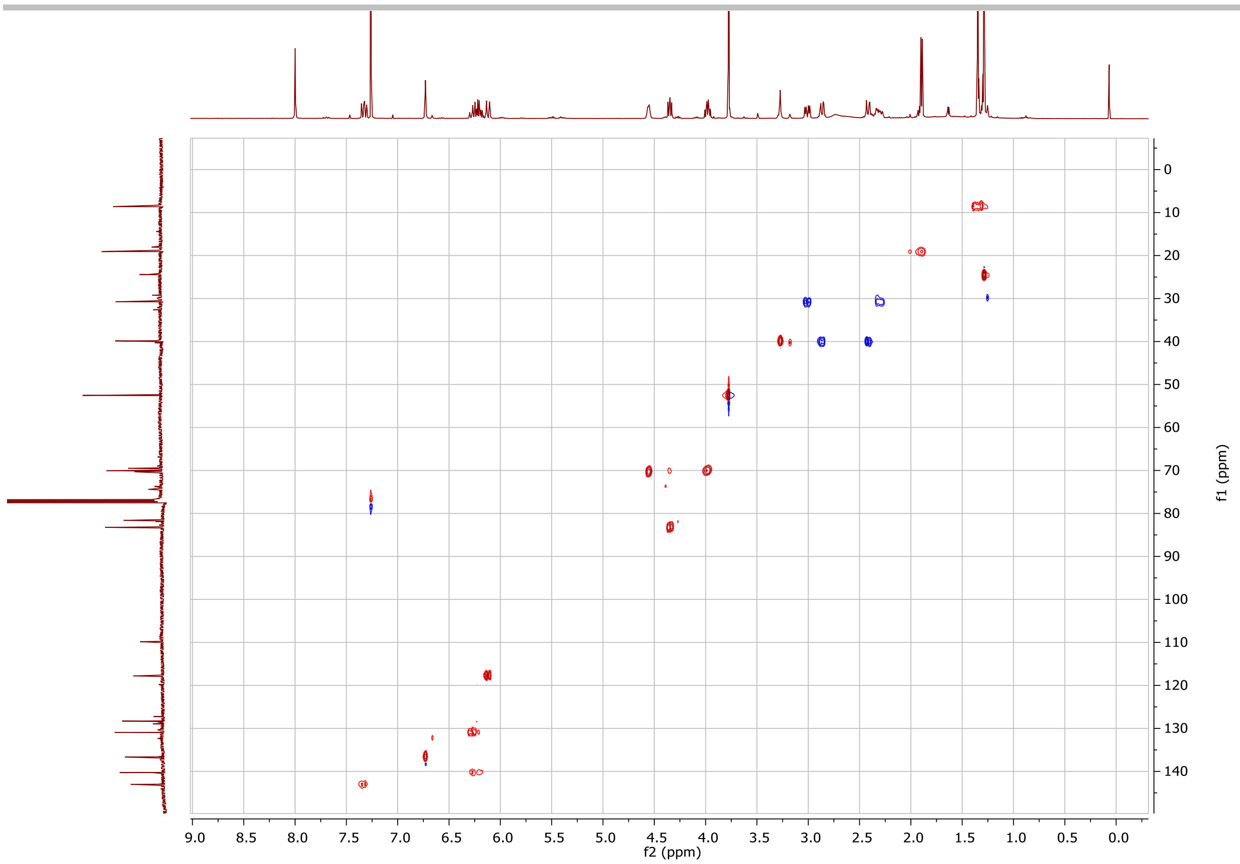

Figure S66: HSQC spectrum for **6b** recorded at 500 MHz in $CDCl_3$.

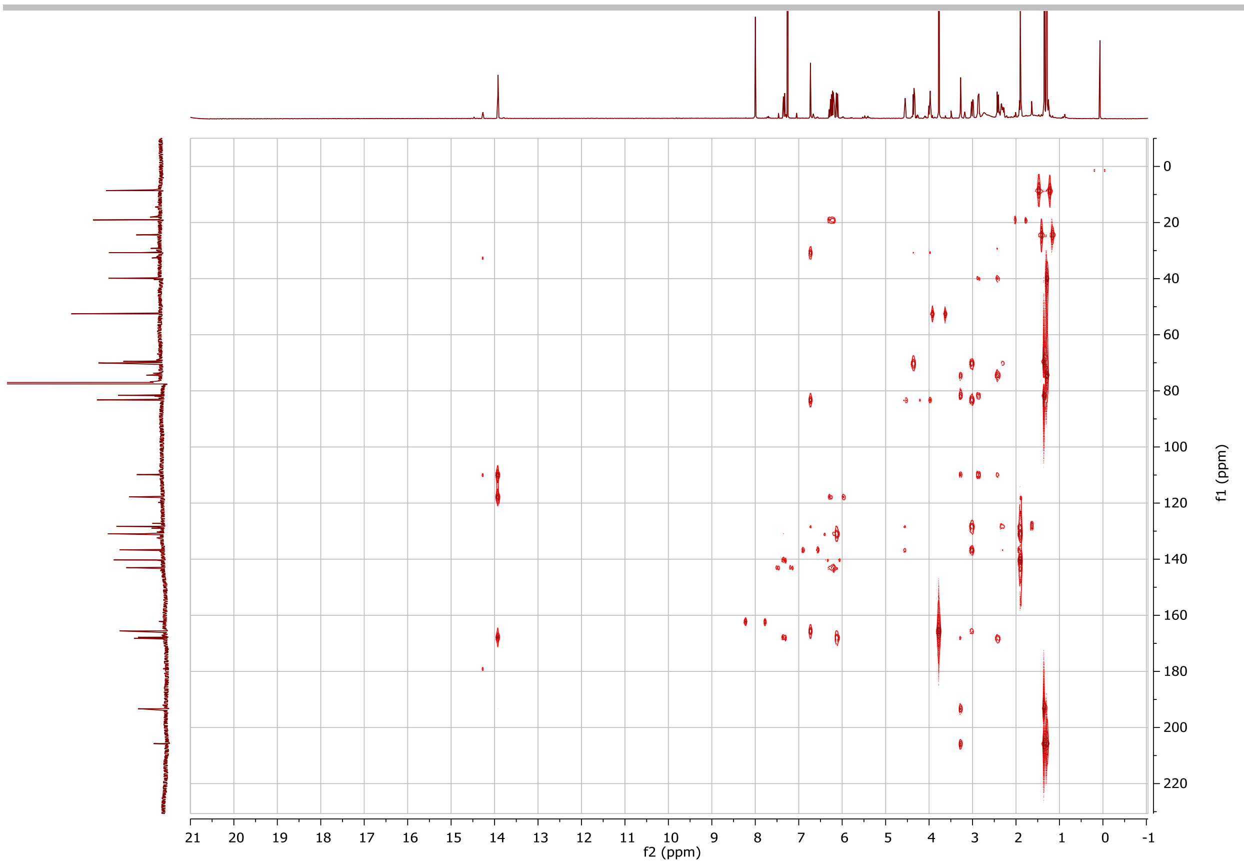

Figure S67: HMBC spectrum for **6b** recorded at 500 MHz in $CDCl_3$.

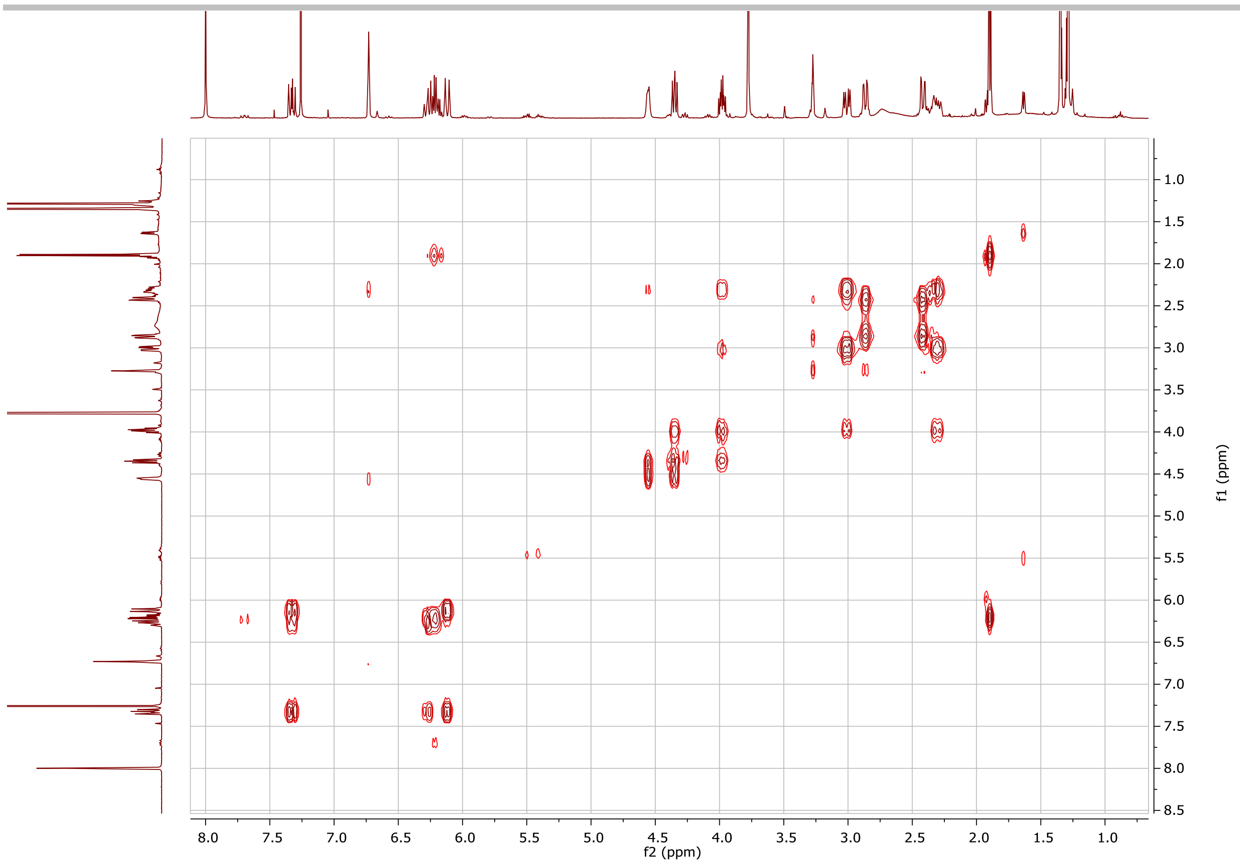

Figure S68: ^{1}H-^{1}H COSY spectrum for **6b** recorded at 500 MHz in $CDCl_3$.

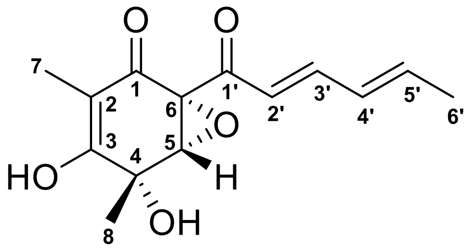

epoxysorbicillinol **10a**

Chemical Formula: $C_{14}H_{16}O_5$
Exact Mass: 264,0998

$UV_{\lambda max}$ (MeOH): 289 nm
HRMS (ESI) *m/z* (*M*+H)$^+$ calcd for $C_{14}H_{17}O_5$: 265.1076, found: 265.1099

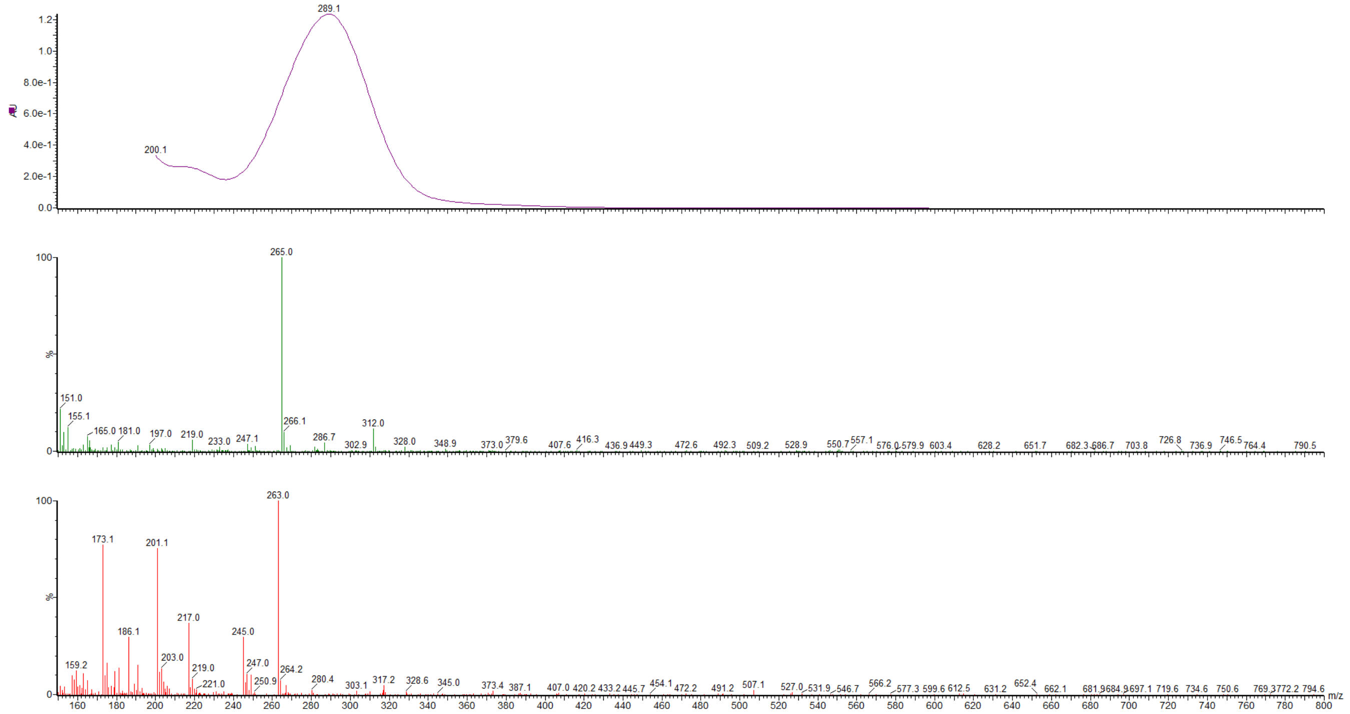

Figure S69: UV-absorption (top) and fragmentation pattern of **10a** in ES+ TIC (middle) and ES- TIC (bottom).

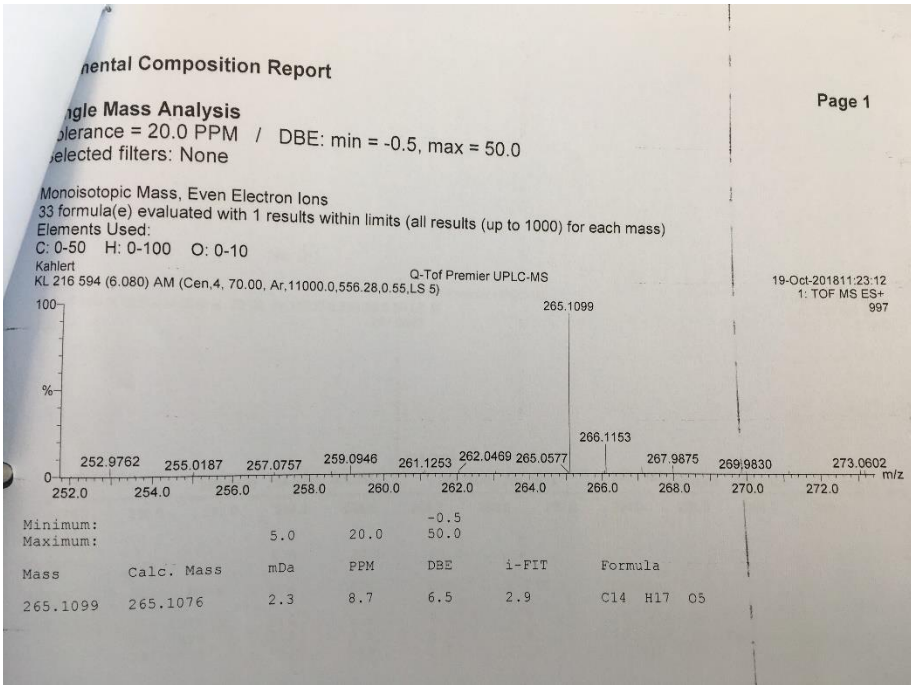

…nental Composition Report

Page 1

…ngle Mass Analysis
…olerance = 20.0 PPM / DBE: min = -0.5, max = 50.0
…elected filters: None

Monoisotopic Mass, Even Electron Ions
33 formula(e) evaluated with 1 results within limits (all results (up to 1000) for each mass)
Elements Used:
C: 0-50 H: 0-100 O: 0-10

Kahlert
KL 216 594 (6.080) AM (Cen,4, 70.00, Ar,11000.0,556.28,0.55,LS 5)
Q-Tof Premier UPLC-MS
19-Oct-201811:23:12
1: TOF MS ES+
997

Minimum:				-0.5		
Maximum:		5.0	20.0	50.0		
Mass	Calc. Mass	mDa	PPM	DBE	i-FIT	Formula
265.1099	265.1076	2.3	8.7	6.5	2.9	C14 H17 O5

Figure S70: HRMS data for **10a.**

Table S12: Summarized NMR data for **10a** recorded in DMSO-d6.

Position	δ_C	δ_H	M	$J_{H\text{-}H}$/Hz	HSQC	HMBC H to C	H-H COSY
1	187.5 (HMBC only)	-	-	-	-	-	-
2	104.1	-	-	-	-	-	-
3	172.7	-	-	-	-	-	-
4	62.7	-	-	-	-	-	-
5	60.9	3.62	s	-	CH	3, 4, 6, 8, 1‘	-
6	68.7	-	-	-	-	-	-
7	8.0	1.55	s	-	CH_3	1, 2, 3	-
8	26.1	1.39	s	-	CH_3	3, 4, 5	-
1‘	192.3	-	-	-	-	-	-
2‘	124.1	6.32	d	5.6	CH	1‘, 3‘, 4‘	3‘
3‘	144.1	7.15	dd	10.0, 15.3	CH	1‘,2‘, 4‘, 5‘	2‘, 4‘
4‘	130.2	6.33	m	-	CH	2‘, 3‘, 6‘	3‘, 5‘
5‘	142.4	6.33	m	-	CH	2’, 3’, 4’, 6’	4‘, 6‘
6‘	18.7	1.84	d	5.6	CH_3	2’, 3’, 4’, 5’	4‘, 5‘

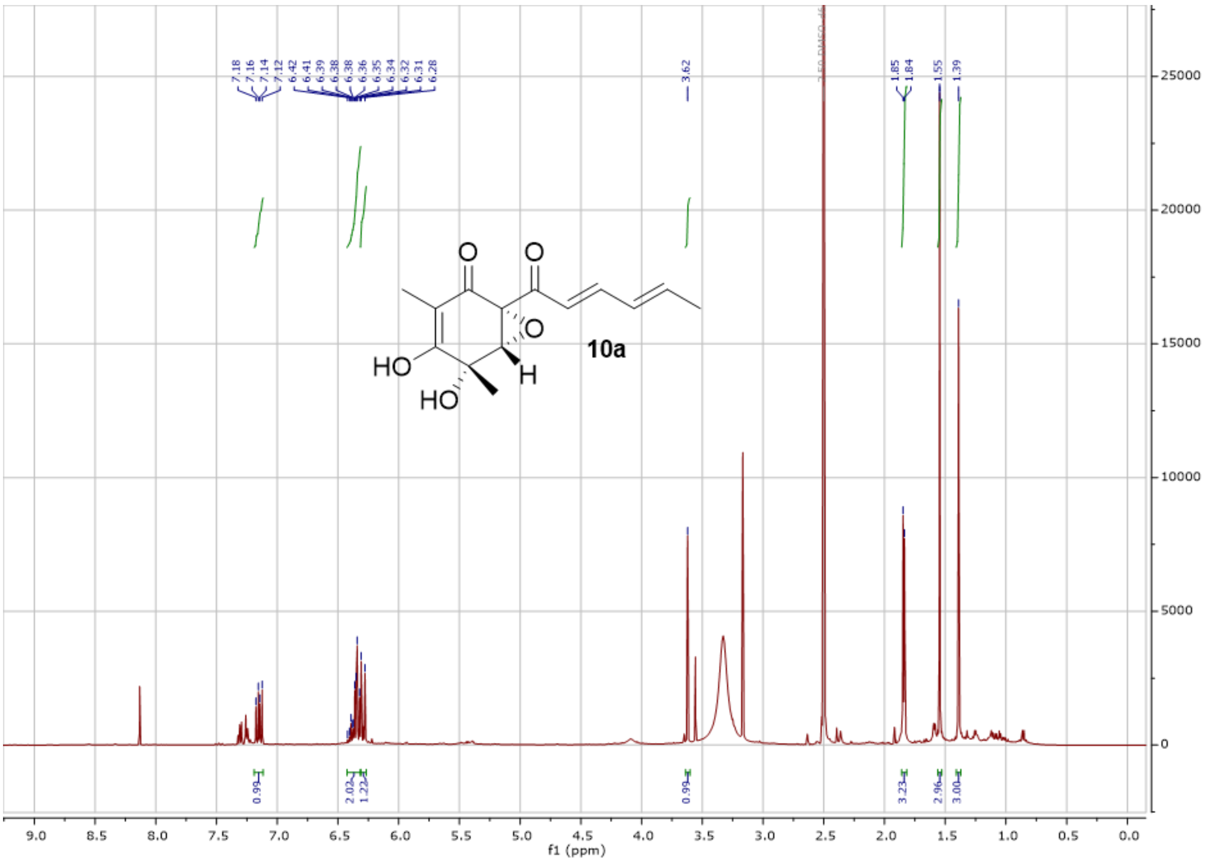

Figure S71: ^{1}H-NMR spectrum for **10a** recorded at 500 MHz in DMSO-d6.

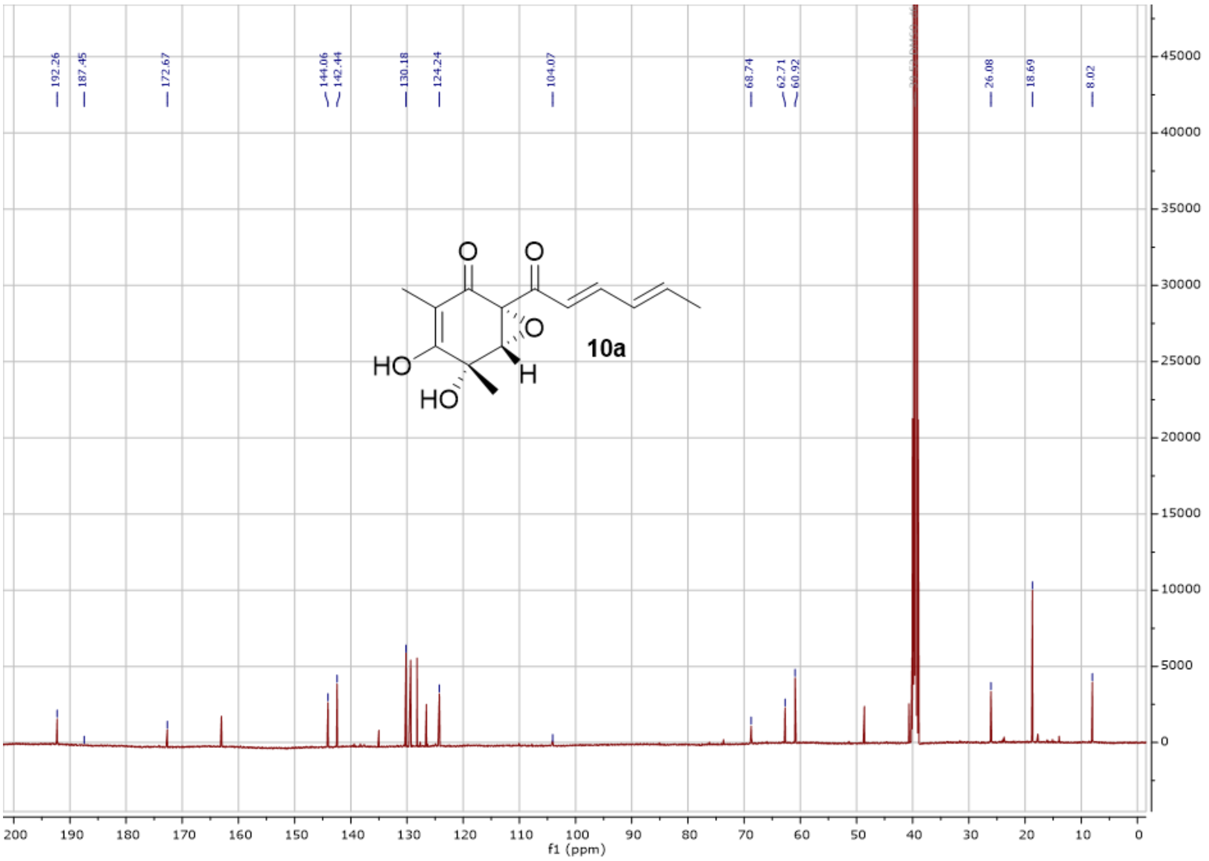

Figure S72: ^{13}C-NMR spectrum for **10a** recorded at 125 MHz in DMSO-d6.

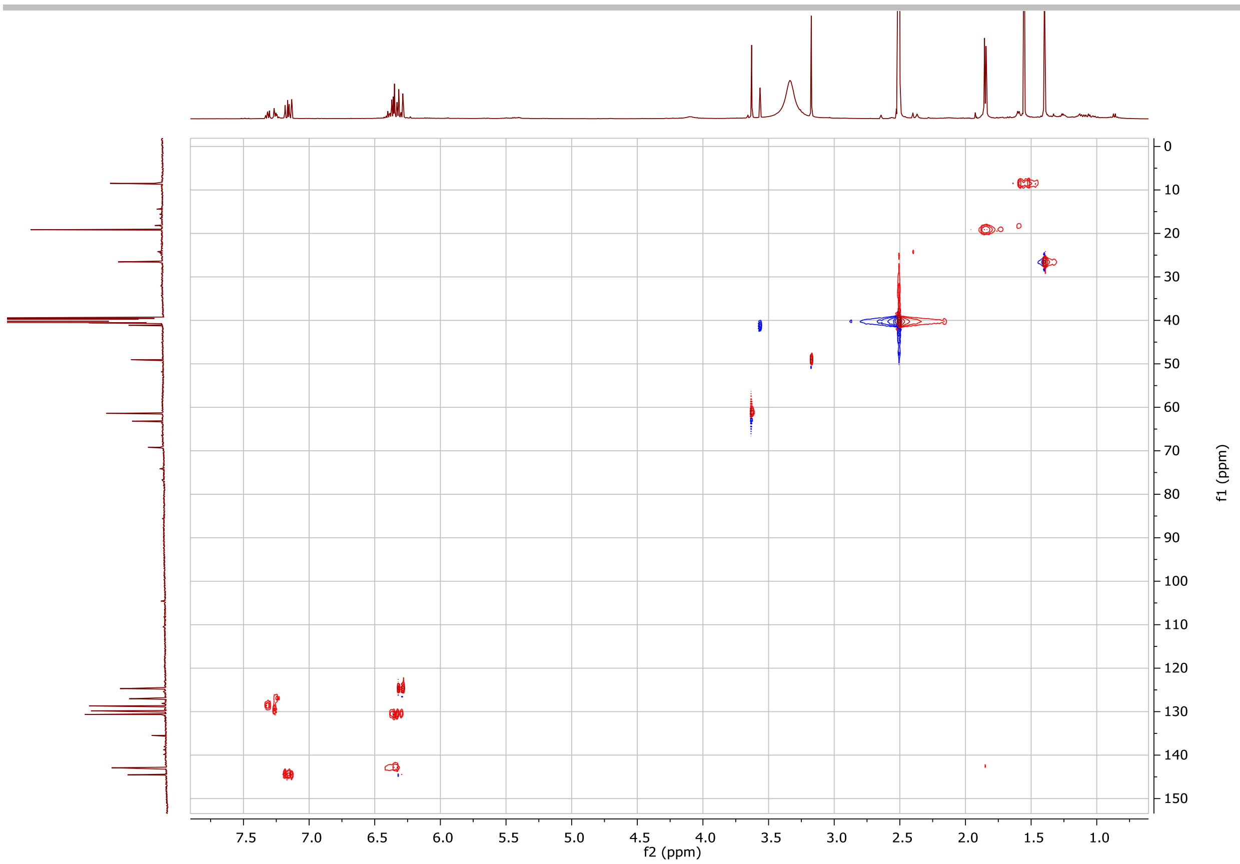

Figure S73: HSQC spectrum for **10a** recorded at 500 MHz in DMSO-d6.

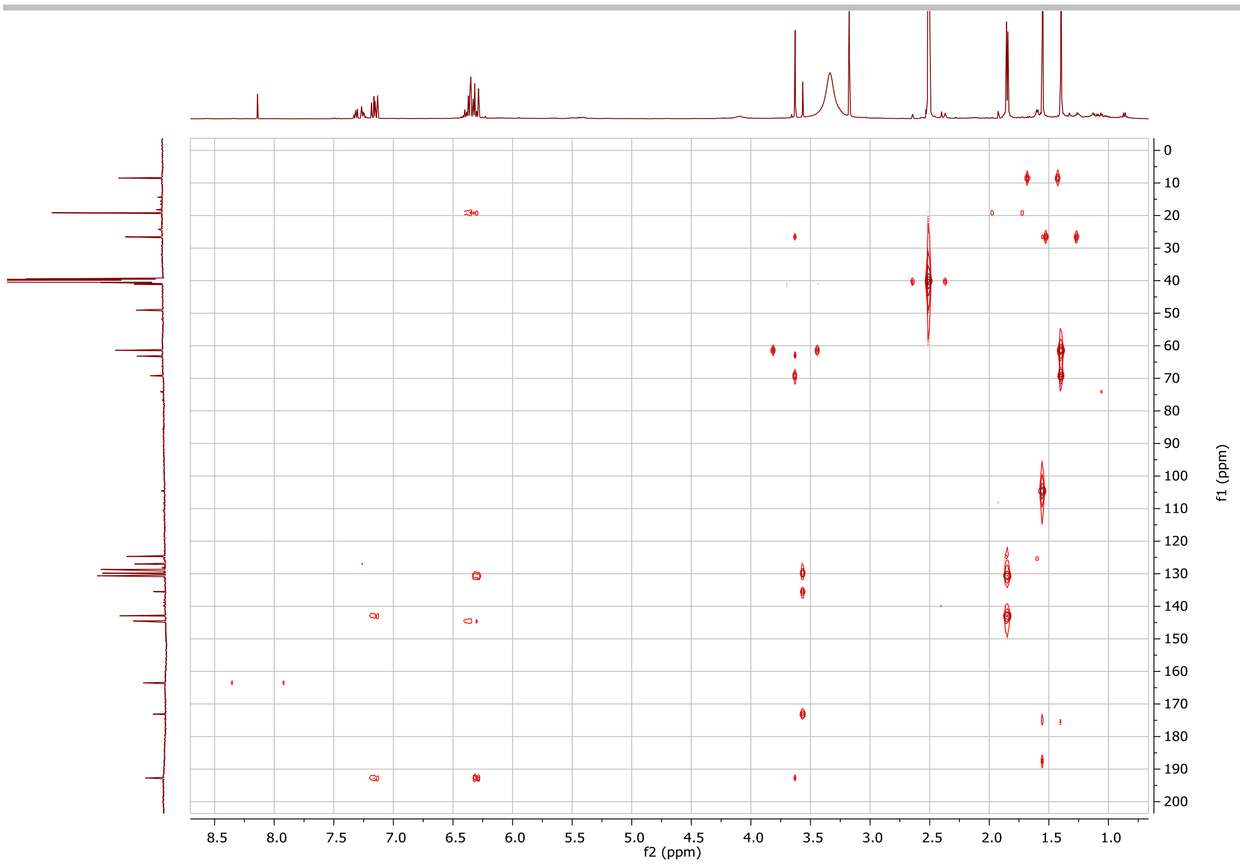

Figure S74: HMBC spectrum for **10a** recorded at 500 MHz in DMSO-d6.

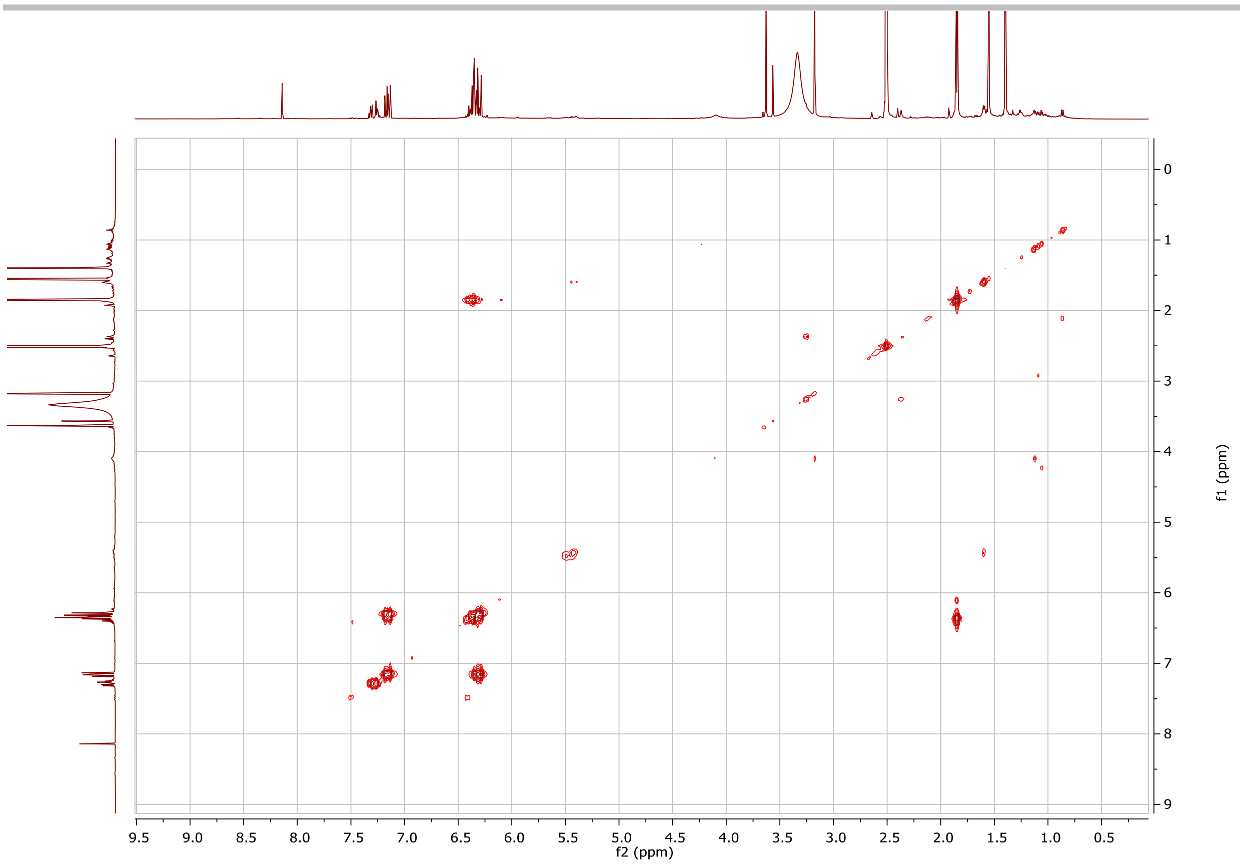

Figure S75: ^{1}H-^{1}H COSY spectrum for **10a** recorded at 500 MHz in DMSO-d6.

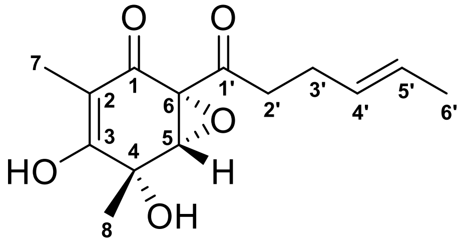

2', 3'-dihydroepoxysorbicillinol **10b**

Chemical Formula: $C_{14}H_{18}O_5$
Exact Mass: 266,1154

UV$_{\lambda max}$ (MeOH): 277 nm
HRMS (ESI) *m/z* (*M*+H)$^+$ calcd for $C_{14}H_{17}O_5$: 265.1076, found: 265.1099

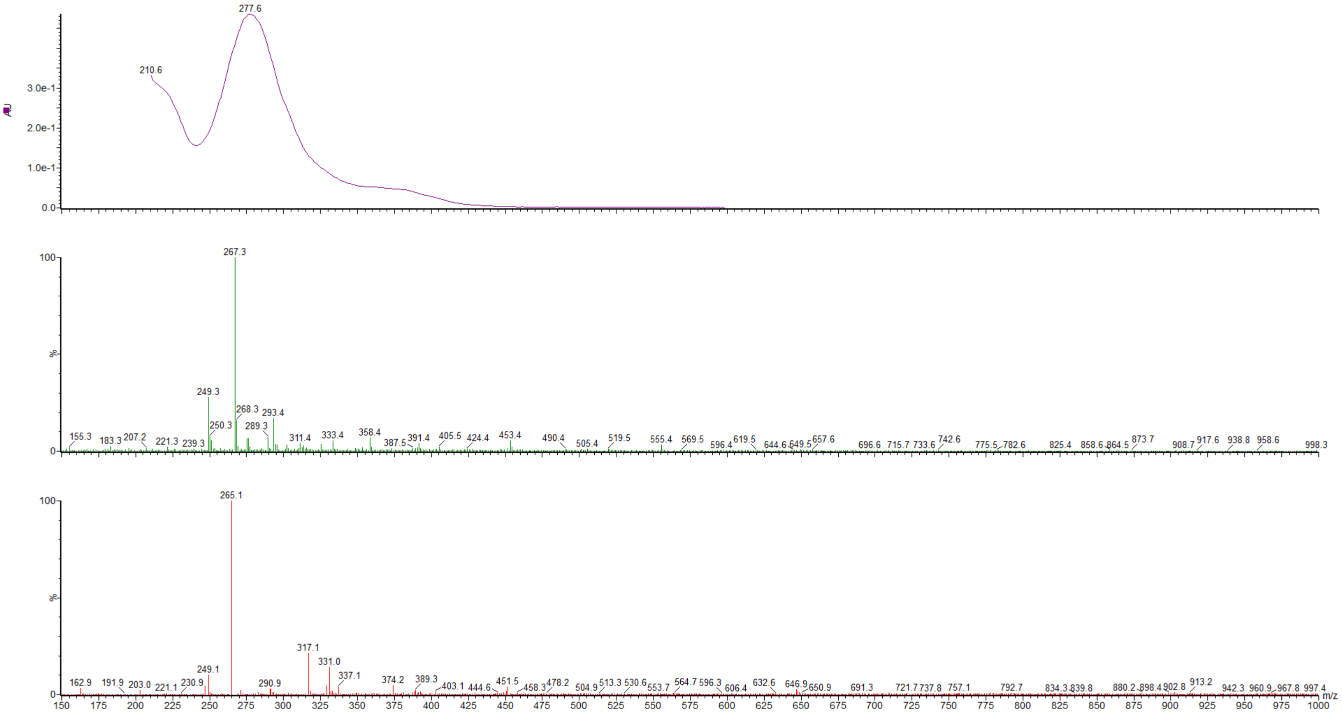

Figure S76: UV-absorption (top) and fragmentation pattern of **10b** in ES+ TIC (middle) and ES- TIC (bottom).

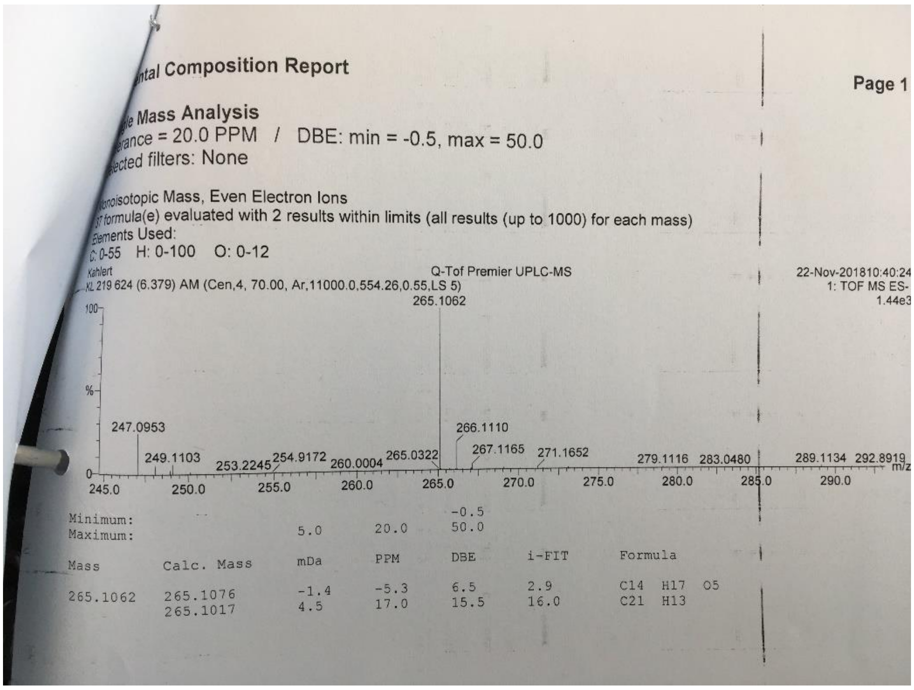
ntal Composition Report

Page 1

le Mass Analysis
rance = 20.0 PPM / DBE: min = -0.5, max = 50.0
ected filters: None

noisotopic Mass, Even Electron Ions
formula(e) evaluated with 2 results within limits (all results (up to 1000) for each mass)
Elements Used:
C: 0-55 H: 0-100 O: 0-12

Kahlert
KL 219 624 (6.379) AM (Cen,4, 70.00, Ar,11000.0,554.26,0.55,LS 5) Q-Tof Premier UPLC-MS 22-Nov-201810:40:24
1: TOF MS ES-
1.44e3

Minimum:				-0.5		
Maximum:		5.0	20.0	50.0		
Mass	Calc. Mass	mDa	PPM	DBE	i-FIT	Formula
265.1062	265.1076	-1.4	-5.3	6.5	2.9	C14 H17 O5
	265.1017	4.5	17.0	15.5	16.0	C21 H13

Figure S77: HRMS data for **10b**.

Table S13: Summarized NMR data for **10b** recorded in DMSO-d6.

Position	δ_C	δ_H	M	$J_{H\text{-}H}$/Hz	HSQC	HMBC H to C	H-H COSY
1	not visible	-	-	-	-	-	-
2	104.3	-	-	-	-	-	-
3	173.7	-	-	-	-	-	-
4	62.8	-	-	-	-	-	-
5	60.8	3.67	s	-	CH	4, 6, 8, 1‘	-
6	68.6	-	-	-	-	-	-
7	7.9	1.55	s	-	CH_3	2, 3	-
8	26.0	1.38	s	-	CH_3	3, 5, 6	-
1‘	202.9	-	-	-	-	-	-
2‘a	40.1	2.57	dd	8.0, 6.7	CH_2	1‘, 4‘, 5‘	2’b, 3‘
2‘b	40.1	2.62	m	-	CH_2	1‘, 4‘, 5‘	2’a, 3‘
3‘	25.5	2.17	m	-	CH_2	1‘,2, 4‘, 5‘	2‘, 4‘, 5‘
4‘	125.2	5.42	m	-	CH	3’, 6’	3‘, 5‘, 6‘
5‘	129.7	5.40	m	-	CH	3’, 6’	3‘, 4‘, 6‘
6‘	17.7	1.61	d	4.9	CH_3	4’, 5’	4‘, 5‘

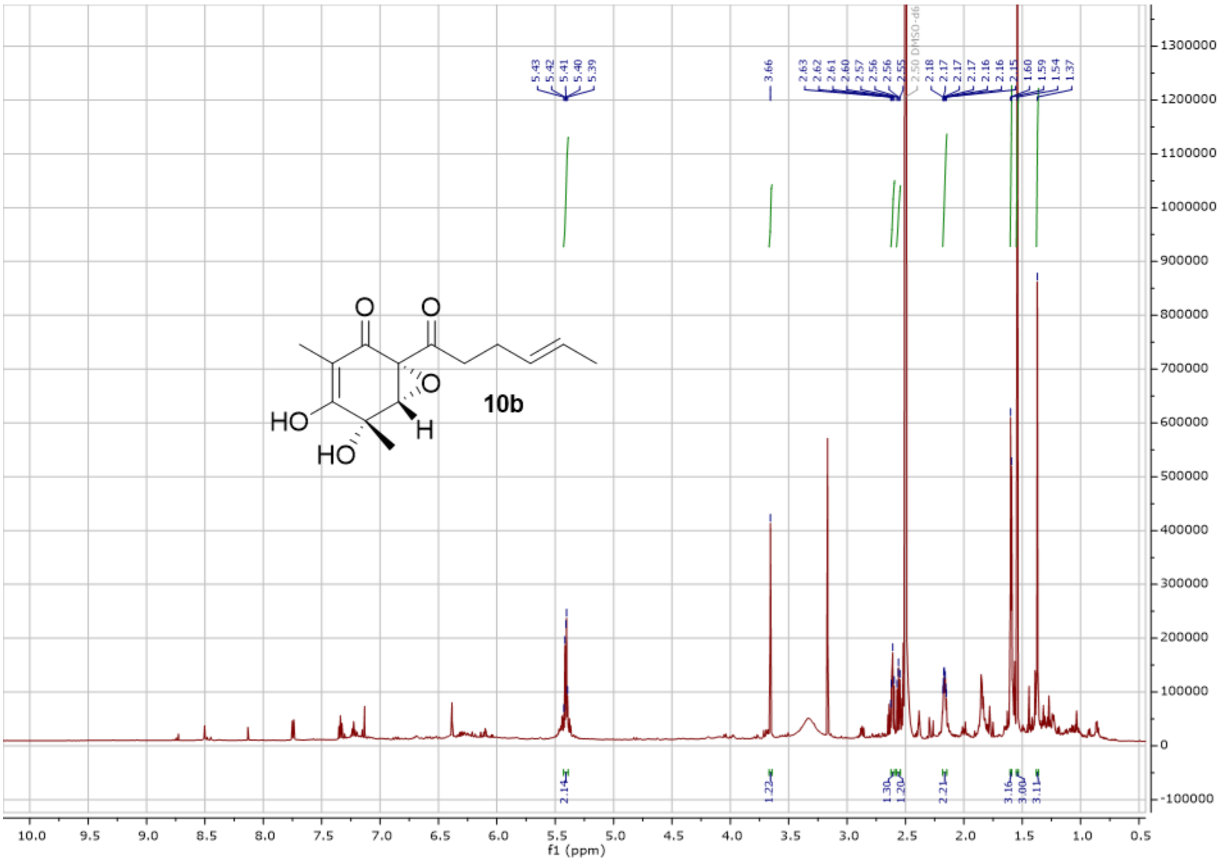

Figure S78: ^{1}H-NMR spectrum for **10b** recorded at 500 MHz in DMSO-d6.

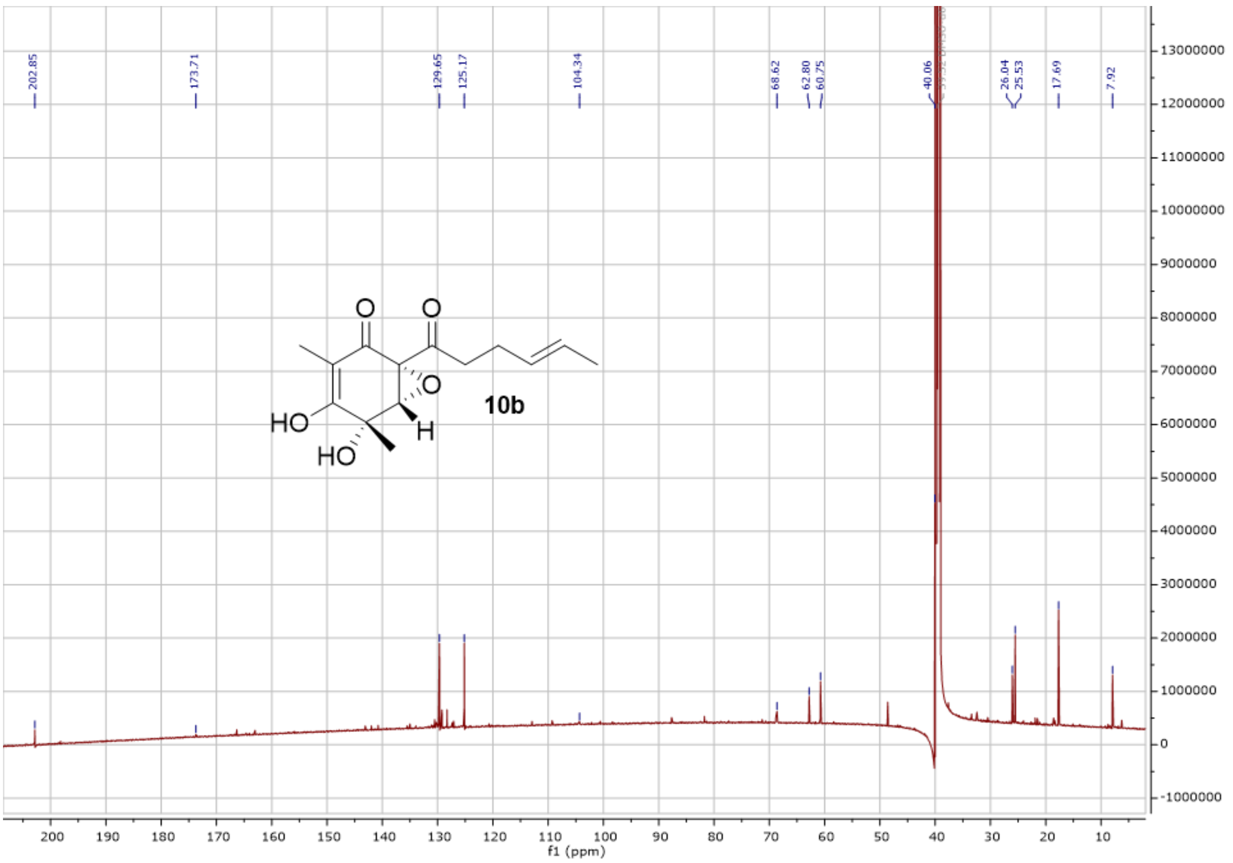

Figure S79: ^{13}C-NMR spectrum for **10b** recorded at 125 MHz in DMSO-d6.

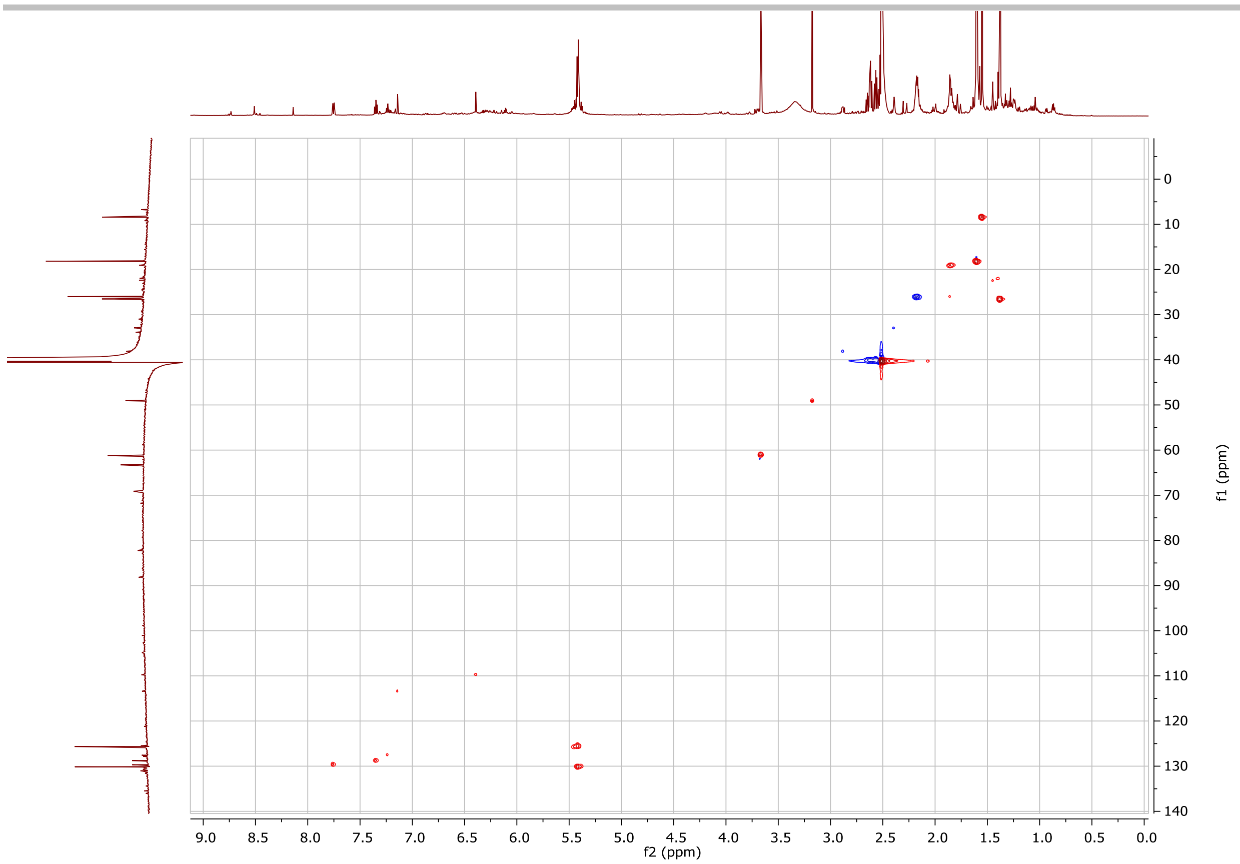

Figure S80: HSQC spectrum for **10b** recorded at 500 MHz in DMSO-d6.

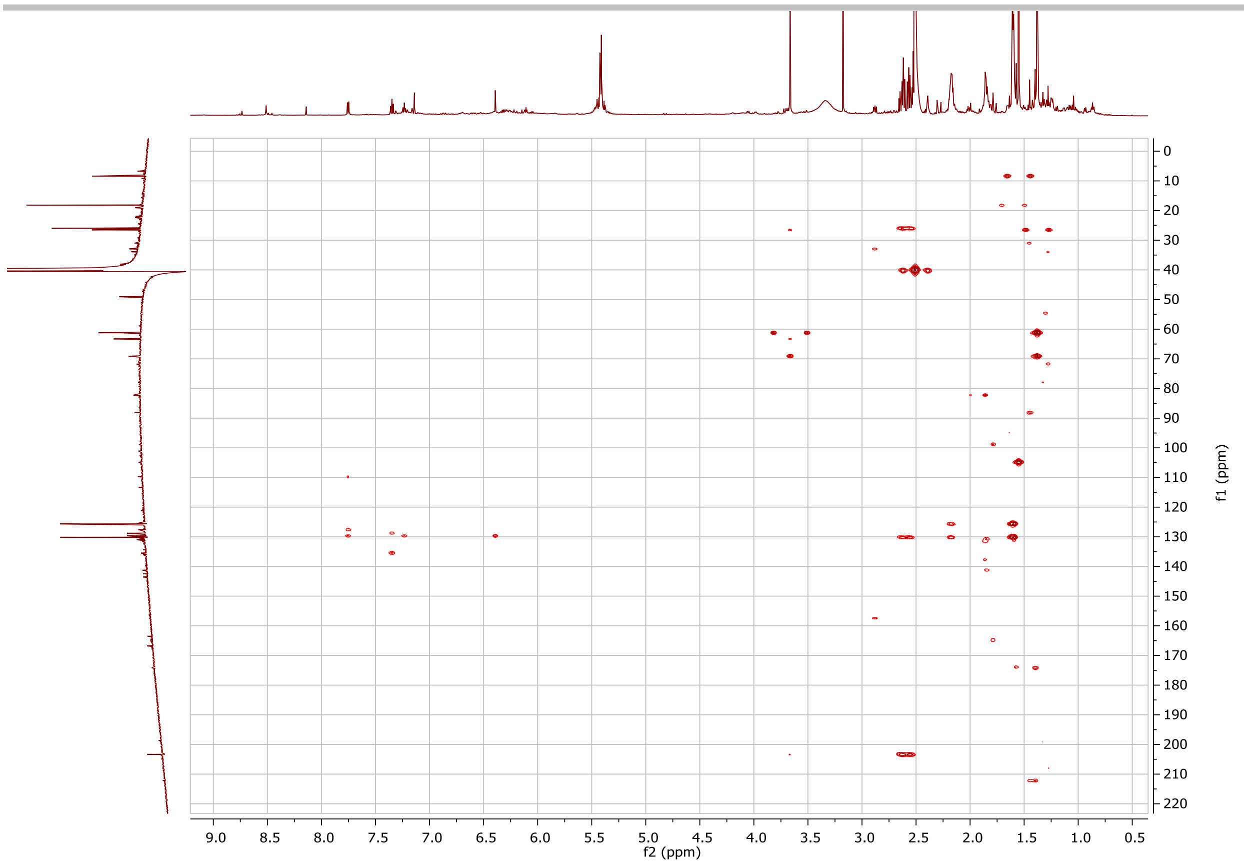

Figure S81: HMBC spectrum for **10b** recorded at 500 MHz in $CDCl_3$.

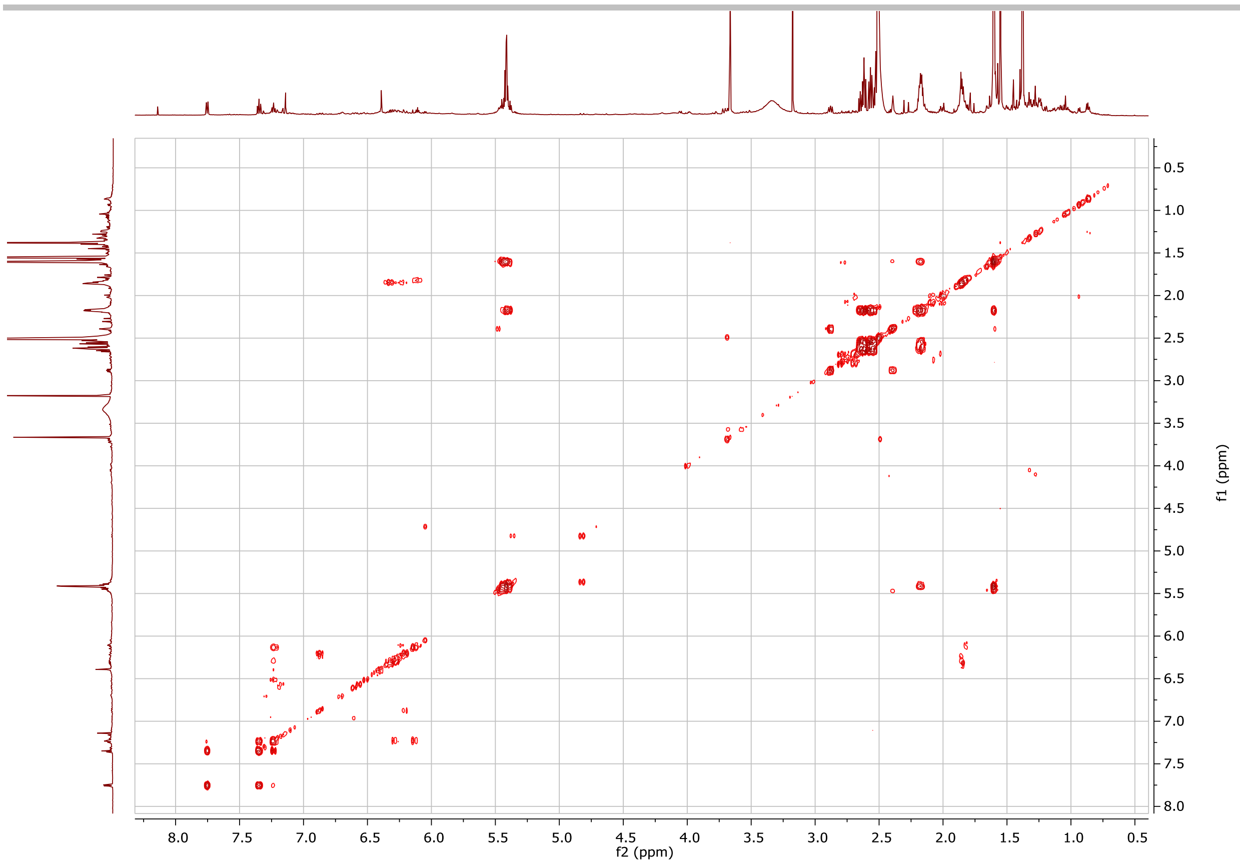

Figure S82: ^1H-^1H COSY spectrum for **10b** recorded at 500 MHz in DMSO-d6.

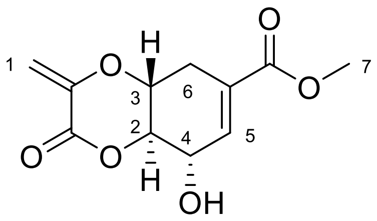

scytolide **12**

Chemical Formula: $C_{11}H_{12}O_6$
Exact Mass: 240,0634

UV$_{\lambda max}$ (MeOH): 214 nm, 245 nm

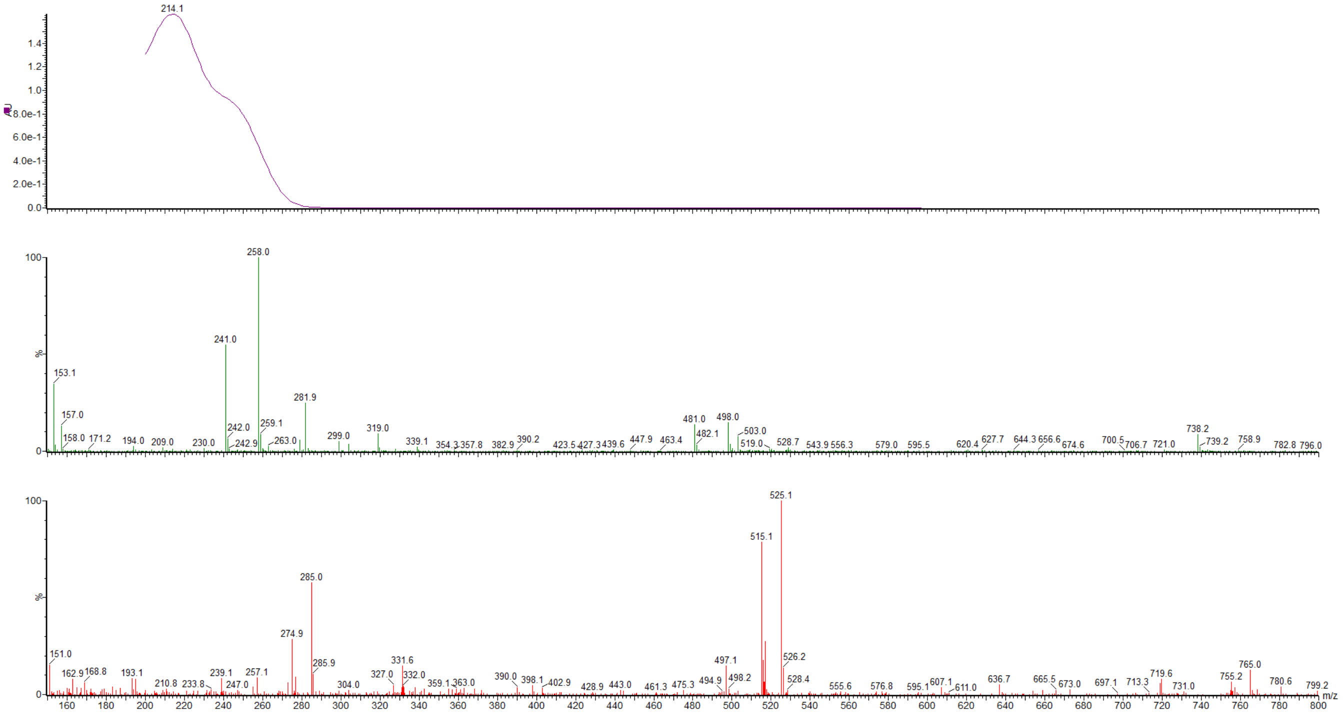

Figure S83: UV-absorption (top) and fragmentation pattern of **12** in ES+ TIC (middle) and ES- TIC (bottom).

Table S14: Summarized NMR data for **12** recorded in $CDCl_3$.

Position	δ_H	M	$J_{H\text{-}H}$/Hz	H-H COSY
1a	5.12	s		1b
1b	5.69	s		1a
2	4.41	dd	10.3, 7.9	3, 4
3	4.11	td	10.1, 6.5	2, 6a, 6b
4	4.60	m		3, 5
5	6.78	s (broad)		4, 6
6a	3.08	dd	17.6, 6.3	3, 6
6b	2.42-2.49	m		3, 6
7	3.79	s	-	-

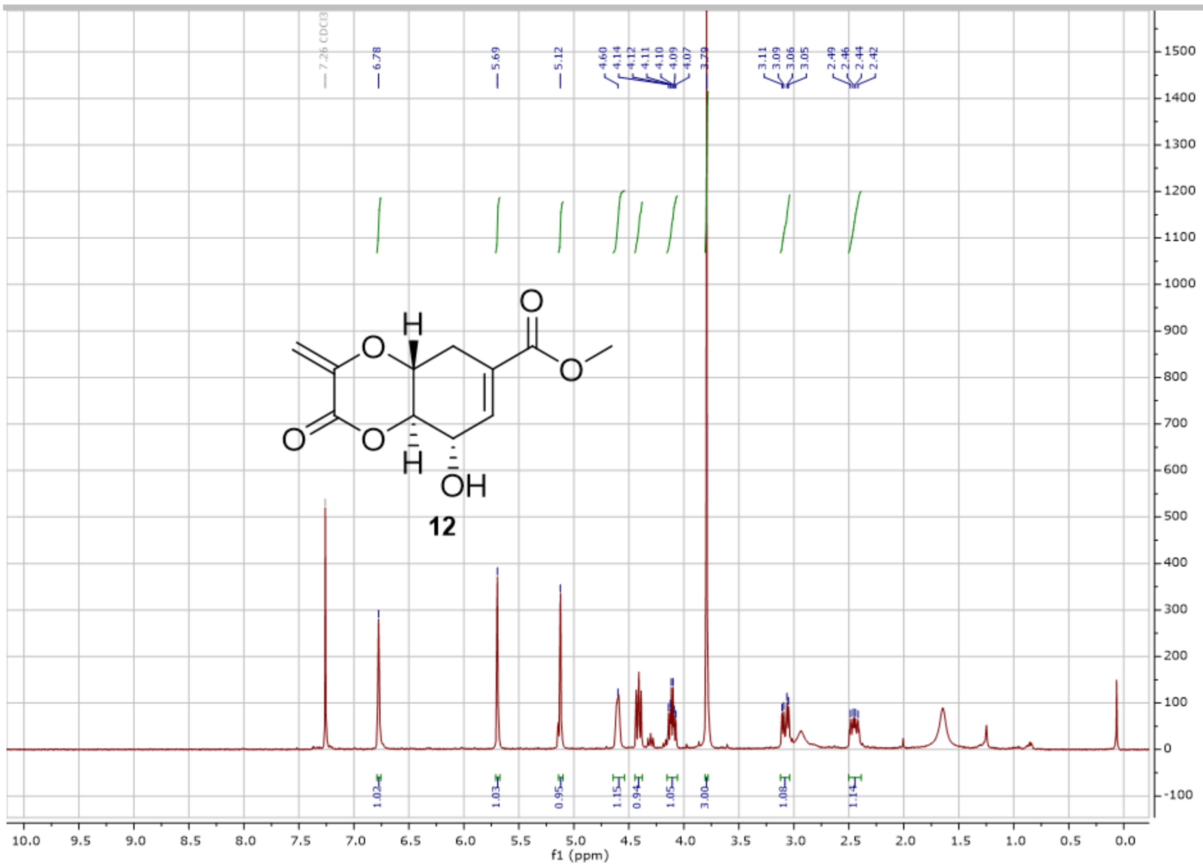

Figure S84: ^{1}H-NMR spectrum for **12** recorded at 600 MHz in CDCl3.

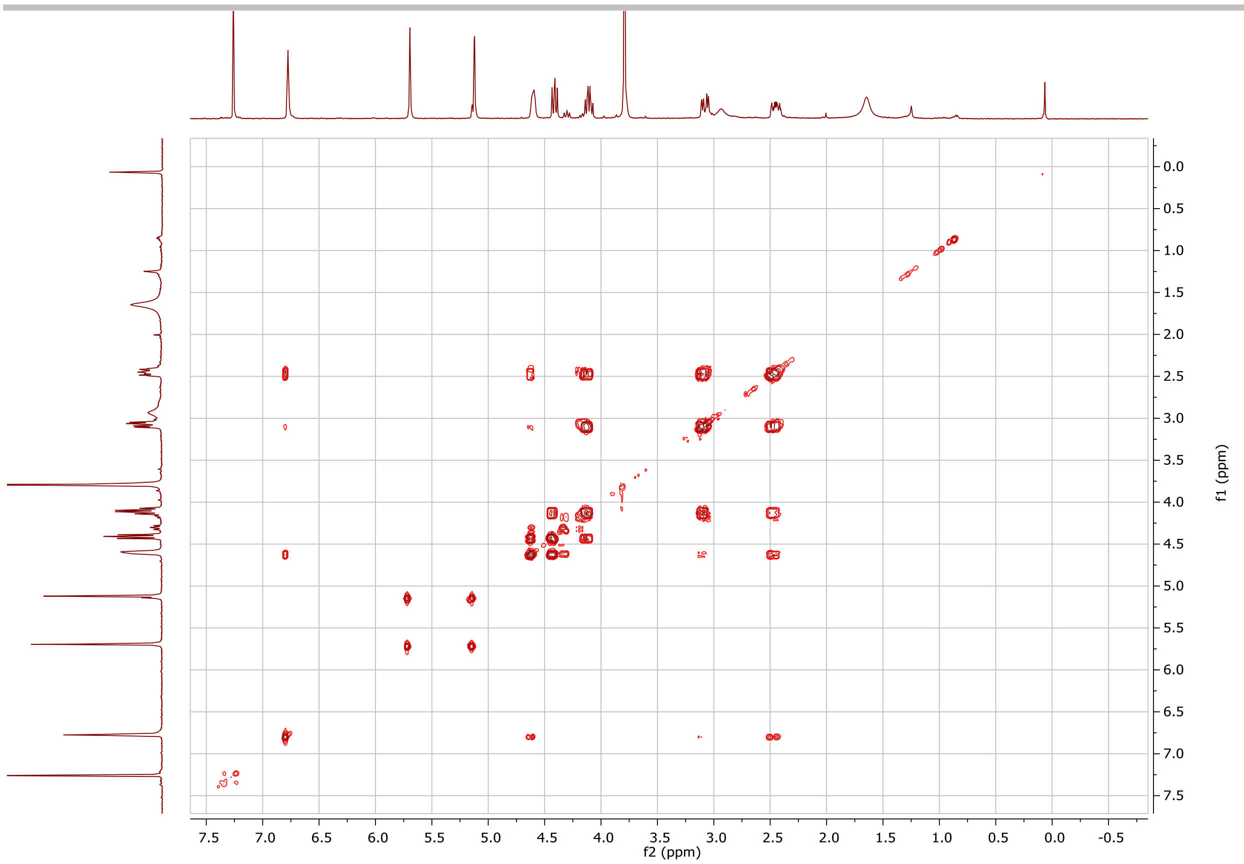

Figure S85: ^{1}H-^{1}H COSY spectrum for **12** recorded at 600 MHz in $CDCl_3$.

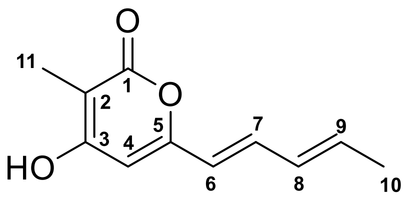

trichopyrone **13**

Chemical Formula: $C_{11}H_{12}O_3$
Exact Mass: 192,0786

$UV_{\lambda max}$ (MeOH): 250 nm, 350 nm
HRMS (ESI) *m/z* (*M*-H)$^-$ calcd for $C_{11}H_{11}O_3$: 191.0708, found: 191.0714

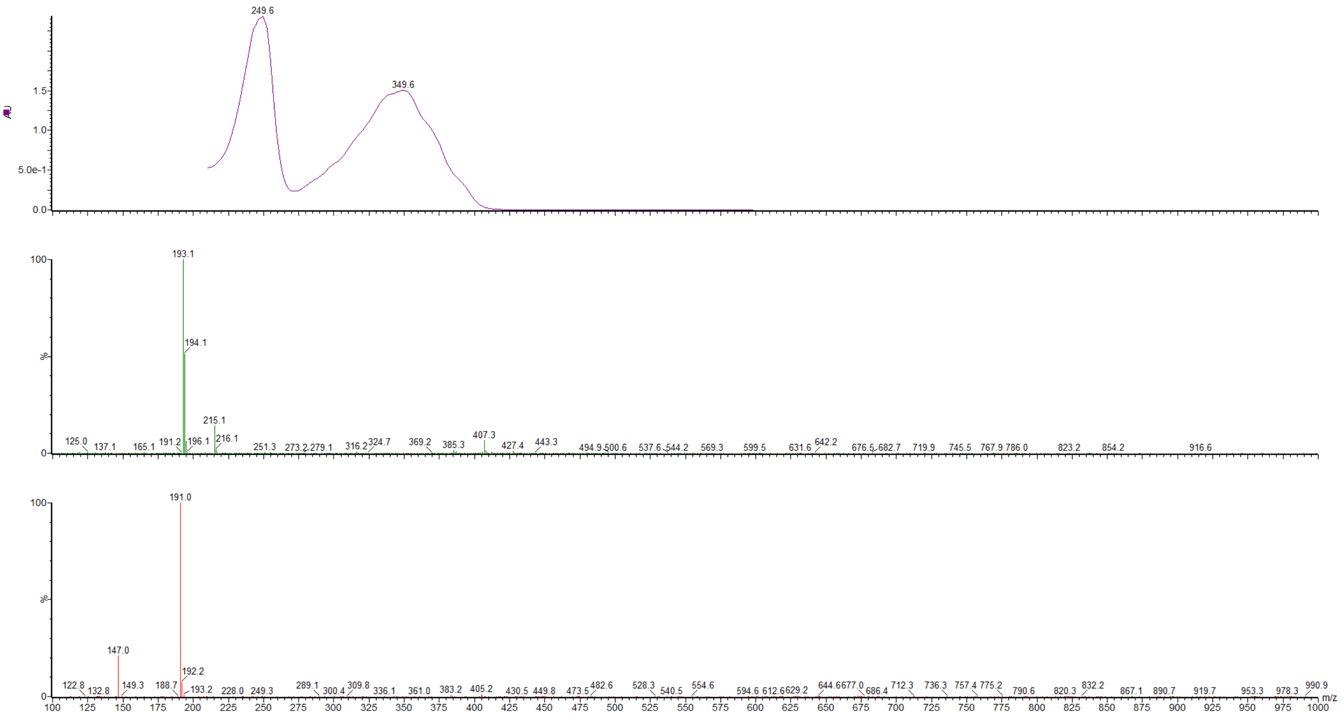

Figure S86: UV-absorption (top) and fragmentation pattern of **13** in ES+ TIC (middle) and ES- TIC (bottom).

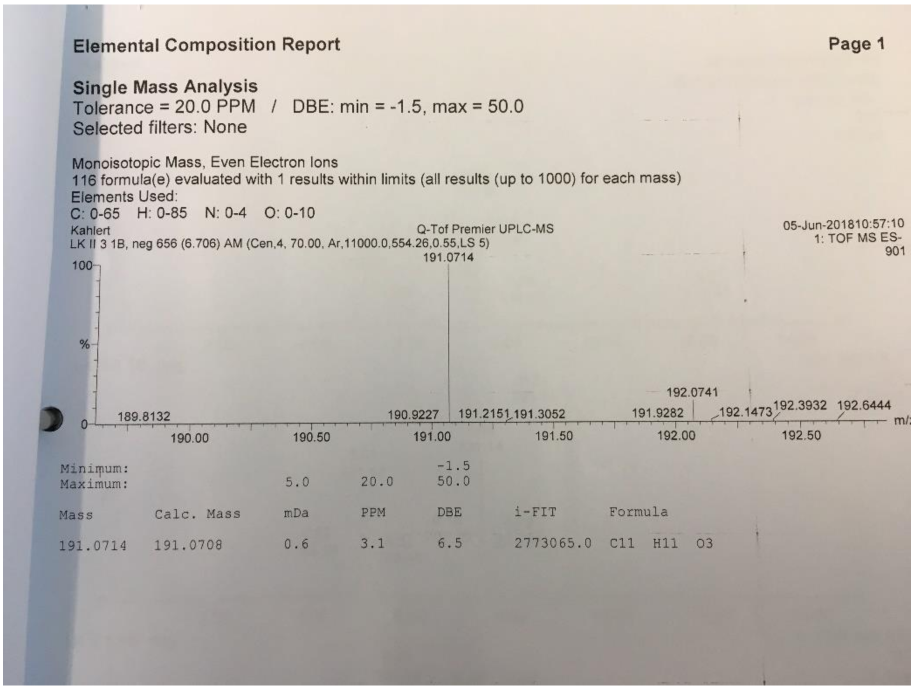

Elemental Composition Report Page 1

Single Mass Analysis
Tolerance = 20.0 PPM / DBE: min = -1.5, max = 50.0
Selected filters: None

Monoisotopic Mass, Even Electron Ions
116 formula(e) evaluated with 1 results within limits (all results (up to 1000) for each mass)
Elements Used:
C: 0-65 H: 0-85 N: 0-4 O: 0-10
Kahlert Q-Tof Premier UPLC-MS 05-Jun-201810:57:10
LK II 3 1B, neg 656 (6.706) AM (Cen,4, 70.00, Ar,11000.0,554.26,0.55,LS 5) 1: TOF MS ES- 901

Minimum:				-1.5		
Maximum:		5.0	20.0	50.0		
Mass	Calc. Mass	mDa	PPM	DBE	i-FIT	Formula
191.0714	191.0708	0.6	3.1	6.5	2773065.0	C11 H11 O3

Figure S87: HRMS data for **13**.

Table S15: Summarized NMR data for **13** recorded in DMSO-d6.

Position	δ_C	δ_H	M	$J_{H\text{-}H}$/Hz	HSQC	HMBC H to C	H-H COSY
1	164.9	-	-	-	-	-	-
2	98.3	-	-	-	-	-	-
3	164.1	-	-	-	-	-	-
4	100.8	6.11	s		CH	2, 5, 6	-
5	155.8	-	-	-	-	-	-
6	120.8	6.20	d	15.4	CH	5, 8	7
7	133.8	6.87	dd	10.7, 15.4	CH	5, 6, 8, 9	6, 8
8	130.8	6.23	m	-	CH	10	9
9	135.5	6.10	m	-	CH	7, 10	10
10	18.4	1.82	d	6.9	CH_3	8, 9	8, 9
11	8.7	1.79	s	-	CH_3	1, 2, 3	-

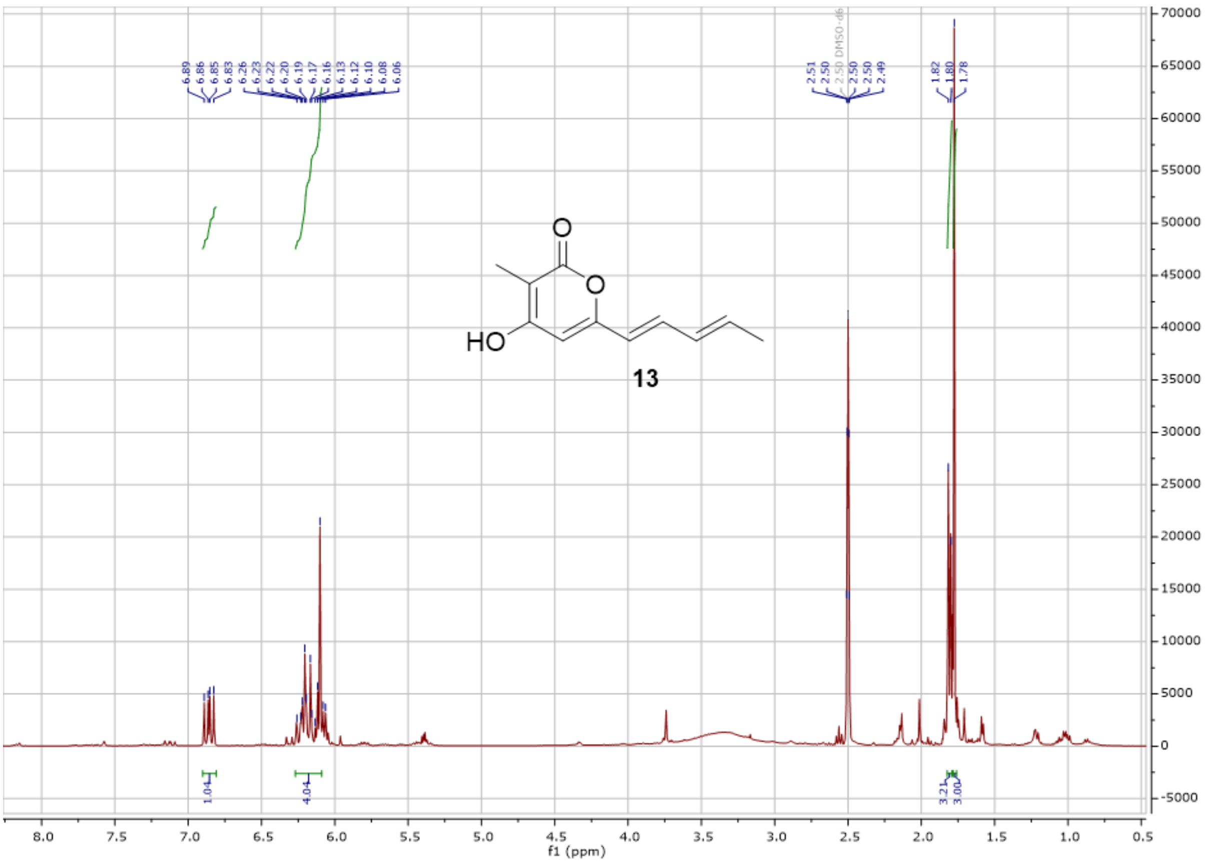

Figure S88: ^{1}H-NMR spectrum for **13** recorded at 400 MHz in DMSO-d6.

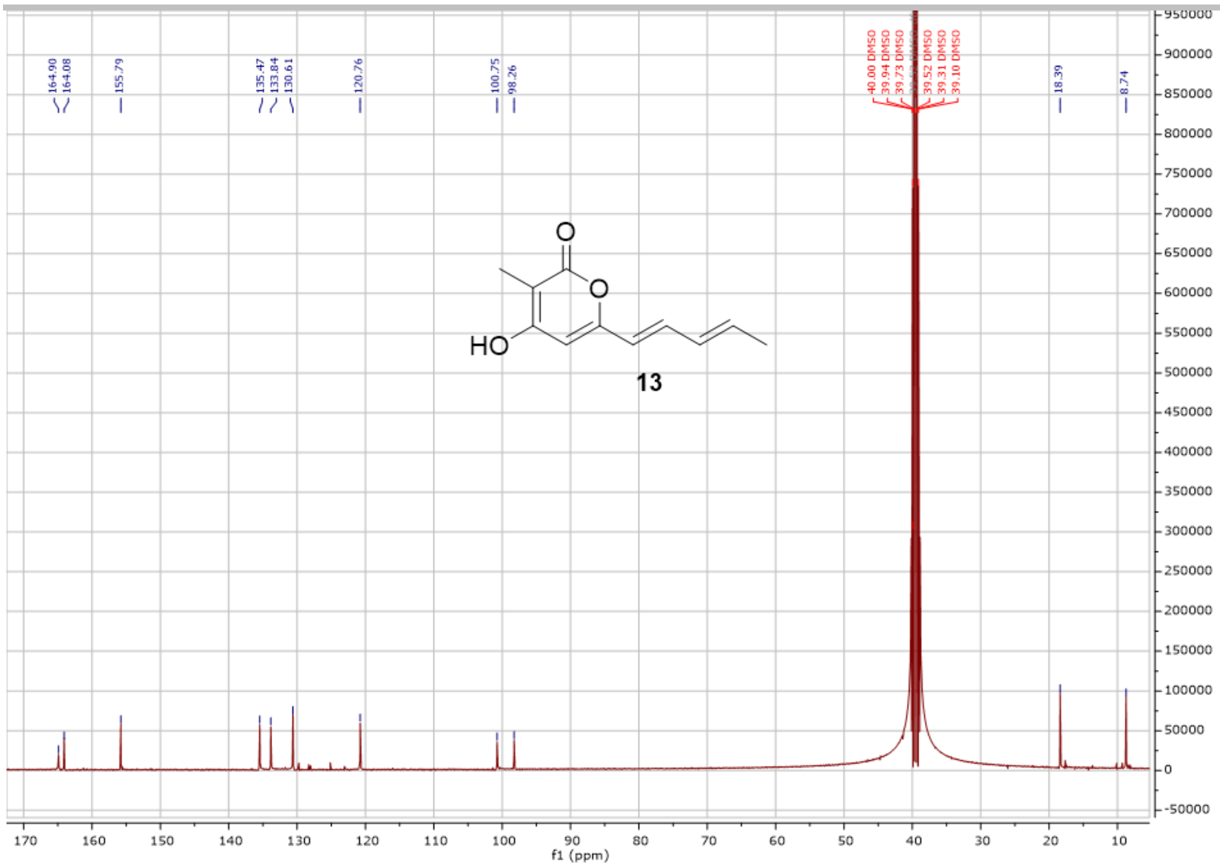

Figure S89: ^{13}C-NMR spectrum for **13** recorded at 125 MHz in DMSO-d6.

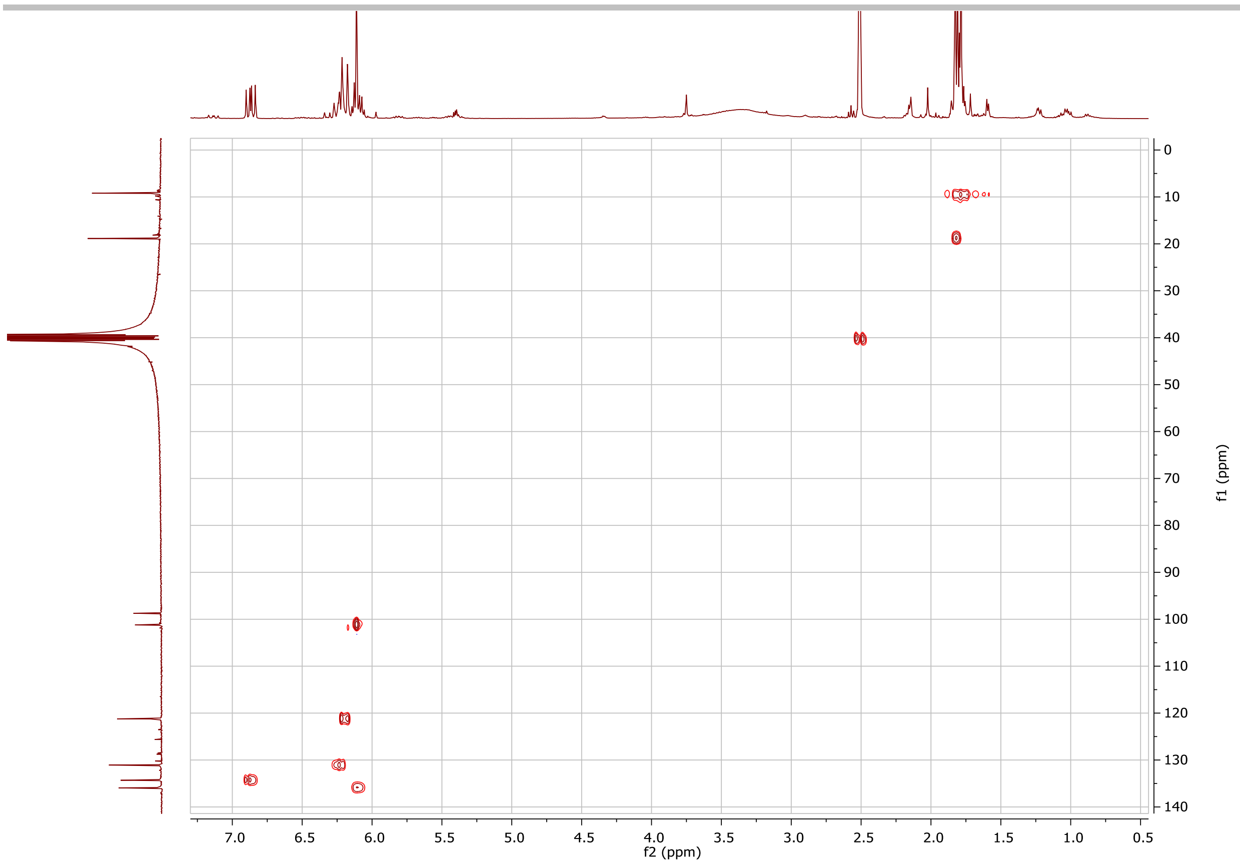

Figure S90: HSQC spectrum for **13** recorded at 400 MHz in DMSO-d6.

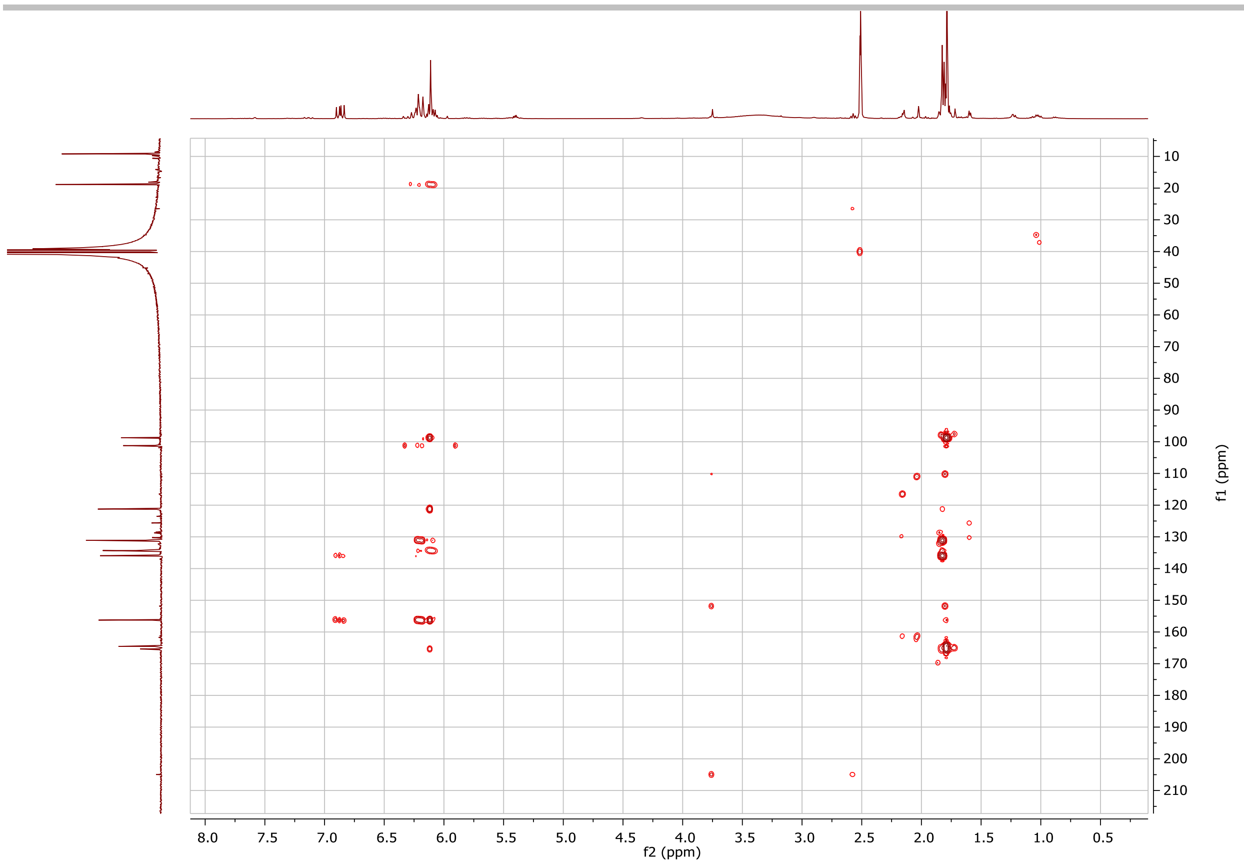

Figure S91: HMBC spectrum for **13** recorded at 400 MHz in DMSO-d6.

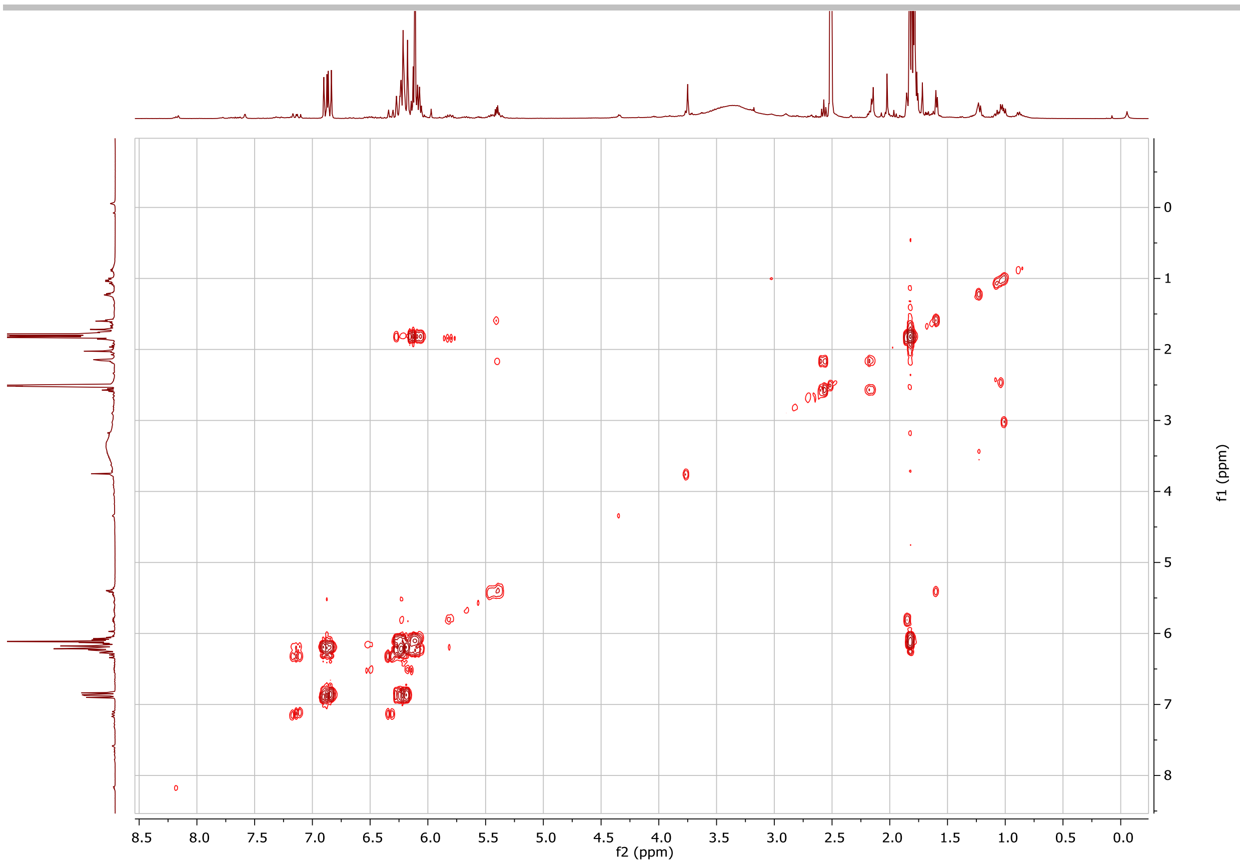

Figure S92: ^{1}H-^{1}H COSY spectrum for **13** recorded at 400 MHz in DMSO-d6.

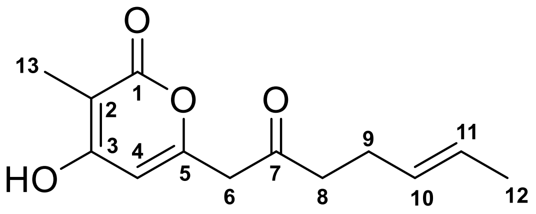

14

Chemical Formula: $C_{13}H_{16}O_4$
Exact Mass: 236,1049

UV$_{\lambda max}$ (MeOH): 243 nm, 343 nm
HRMS (ESI) *m/z* (*M*-H)$^-$ calcd for $C_{13}H_{15}O_4$: 235.0970, found: 235.0991

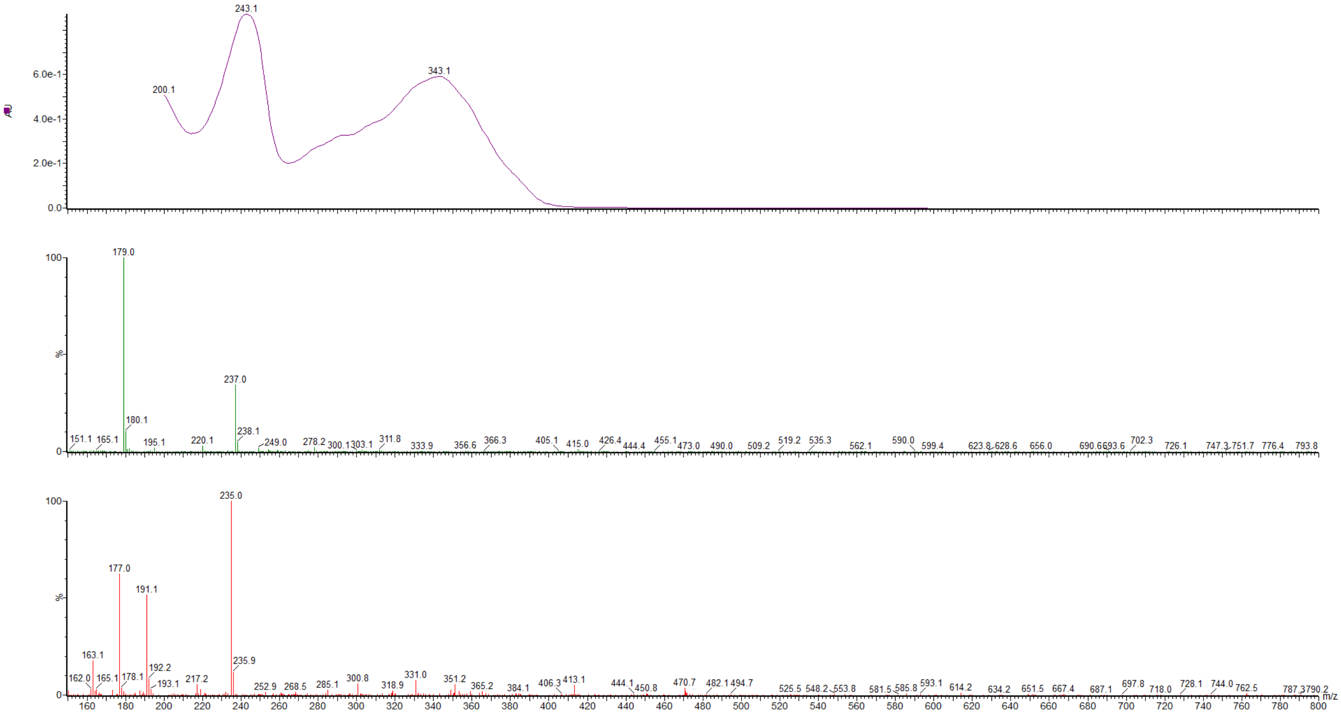

Figure S93: UV-absorption (top) and fragmentation pattern of **14** in ES+ TIC (middle) and ES- TIC (bottom).

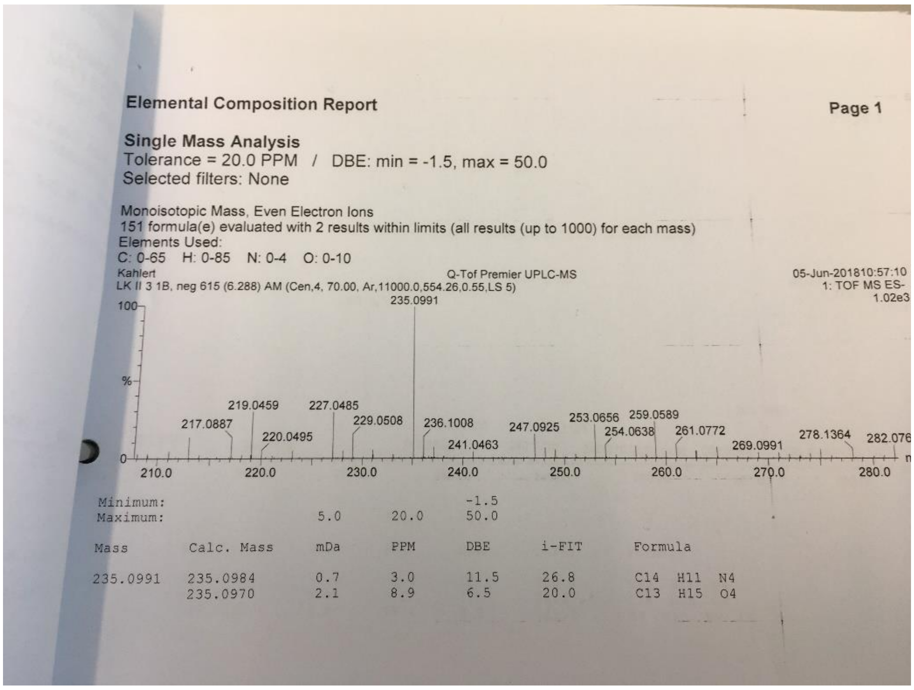

Elemental Composition Report

Page 1

Single Mass Analysis
Tolerance = 20.0 PPM / DBE: min = -1.5, max = 50.0
Selected filters: None

Monoisotopic Mass, Even Electron Ions
151 formula(e) evaluated with 2 results within limits (all results (up to 1000) for each mass)
Elements Used:
C: 0-65 H: 0-85 N: 0-4 O: 0-10
Kahlert
LK II 3 1B, neg 615 (6.288) AM (Cen,4, 70.00, Ar,11000.0,554.26,0.55,LS 5)
Q-Tof Premier UPLC-MS
05-Jun-201810:57:10
1: TOF MS ES-
1.02e3

Minimum:				-1.5		
Maximum:		5.0	20.0	50.0		
Mass	Calc. Mass	mDa	PPM	DBE	i-FIT	Formula
235.0991	235.0984	0.7	3.0	11.5	26.8	C14 H11 N4
	235.0970	2.1	8.9	6.5	20.0	C13 H15 O4

Figure S94: HRMS data for **14**.

Table S16: Summarized NMR data for **14** recorded in DMSO-d6.

Position	δ_C	δ_H	M	$J_{H\text{-}H}$/Hz	HSQC	HMBC H to C	H-H COSY
1	165.7	-	-	-	-	-	-
2	92.2	-	-	-	-	-	-
3	175.0	-	-	-	-	-	-
4	107.9	5.61	s	-	CH	2, 5	-
5	153.5	-	-	-	-	-	-
6	47.1	3.43	s	-	CH_2	4, 5, 7	-
7	205.2	-	-	-	-	-	-
8	41.2	2.52	d	7.3	CH_2	7, 9, 10	9
9	26.0	2.13	m	-	CH_2	7, 8, 10, 11	8, 10
10	129.9	5.39	m	-	CH	12	9, 11, 12
11	125.1	5.39	m	-	CH	12	9, 10, 12
12	17.7	1.59	d	5.1	CH_3	10, 11	10, 11
13	9.2	1.59	s	-	CH_3	1, 2, 3	-

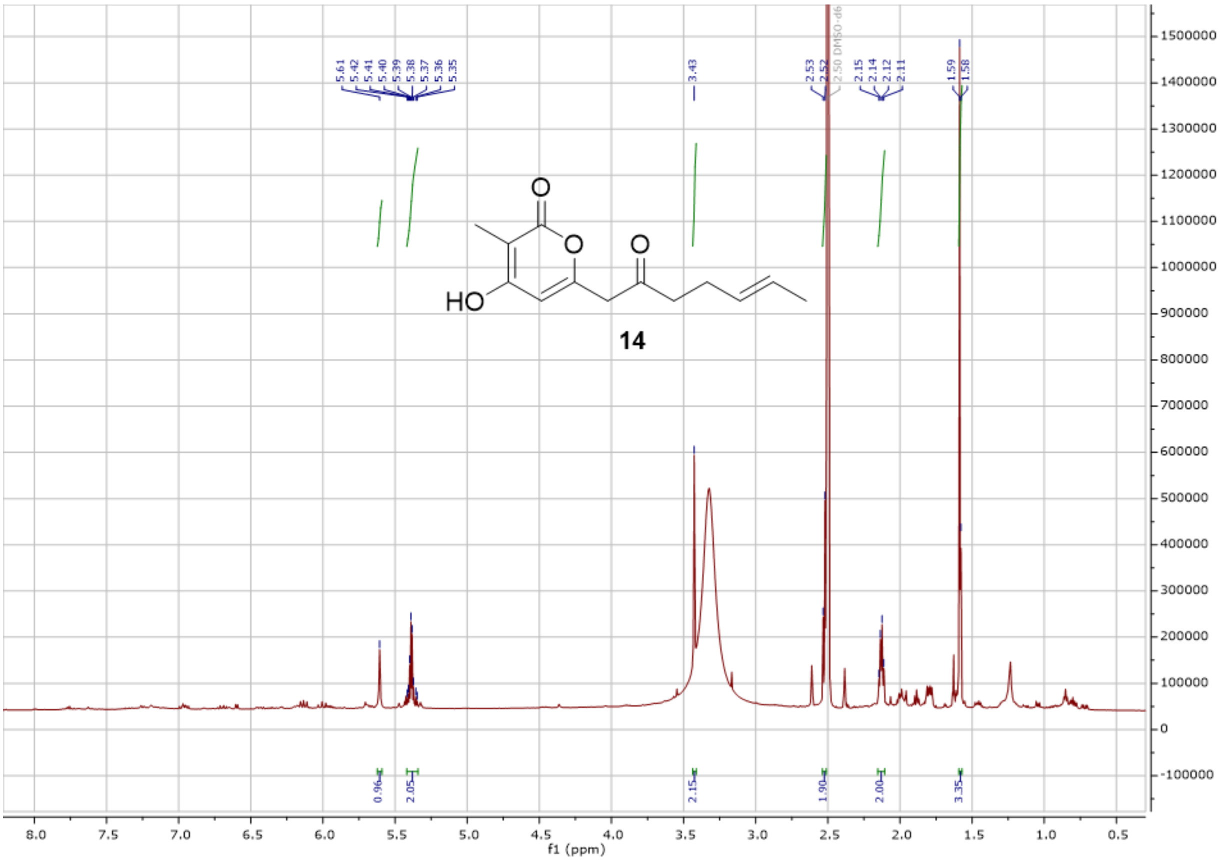

Figure S95: ^{1}H-NMR spectrum for **14** recorded at 600 MHz in DMSO-d6.

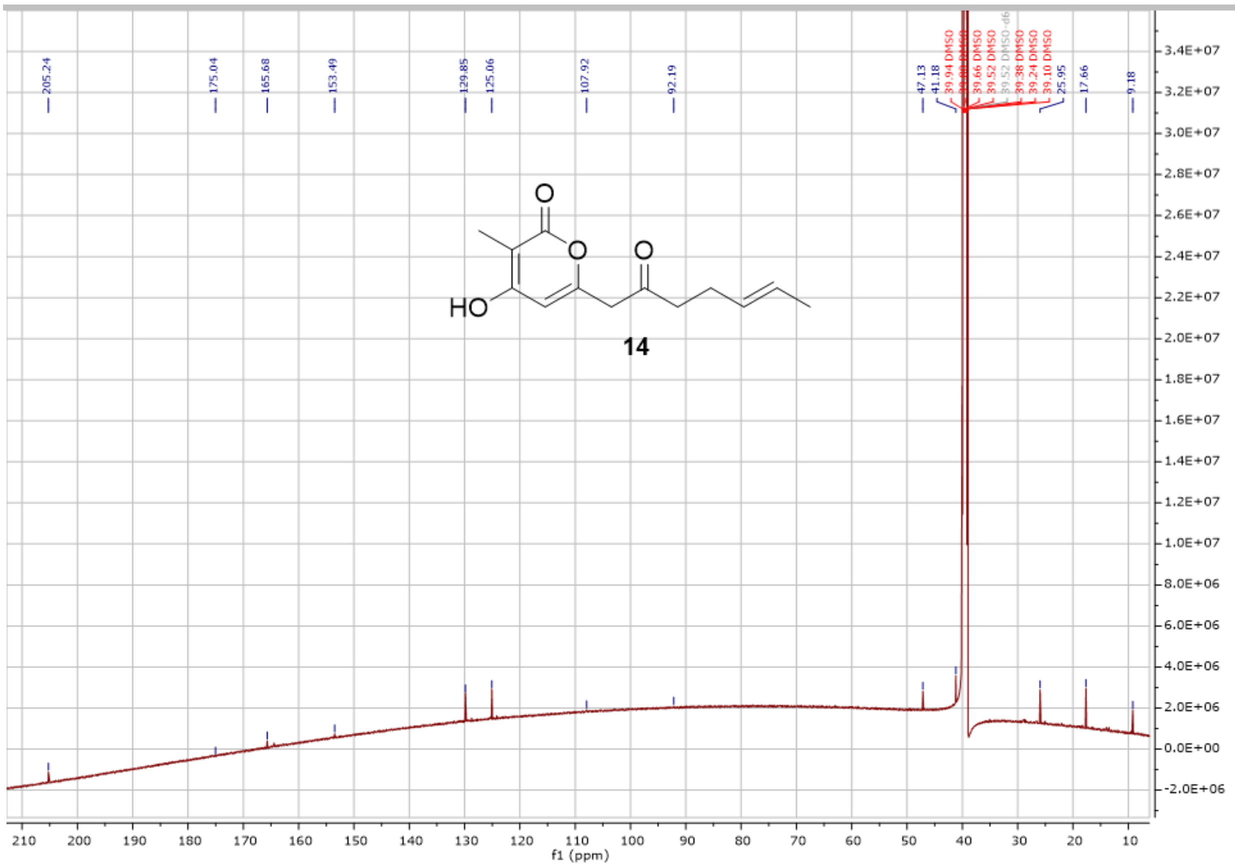

Figure S96: ^{13}C-NMR spectrum for **14** recorded at 125 MHz in DMSO-d6.

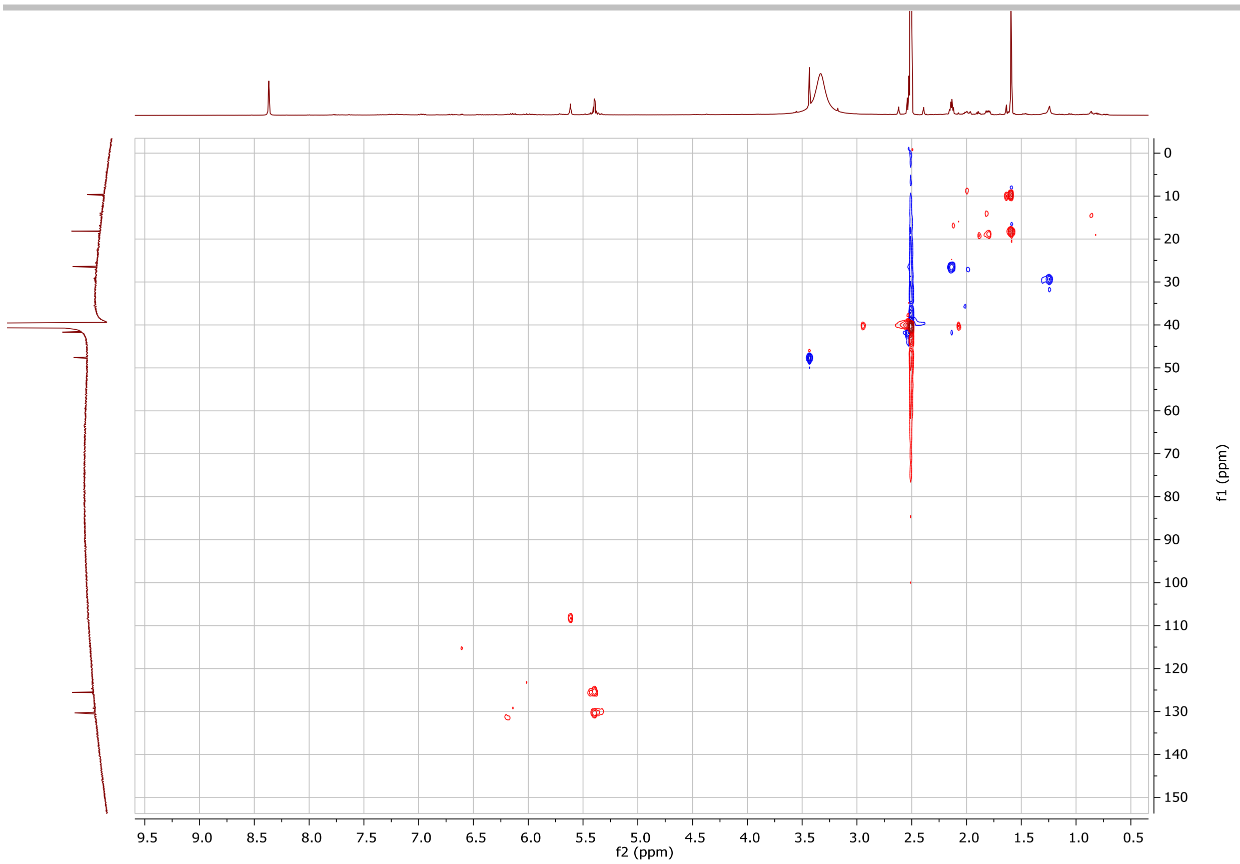

Figure S97: HSQC spectrum for **14** recorded at 600 MHz in DMSO-d6.

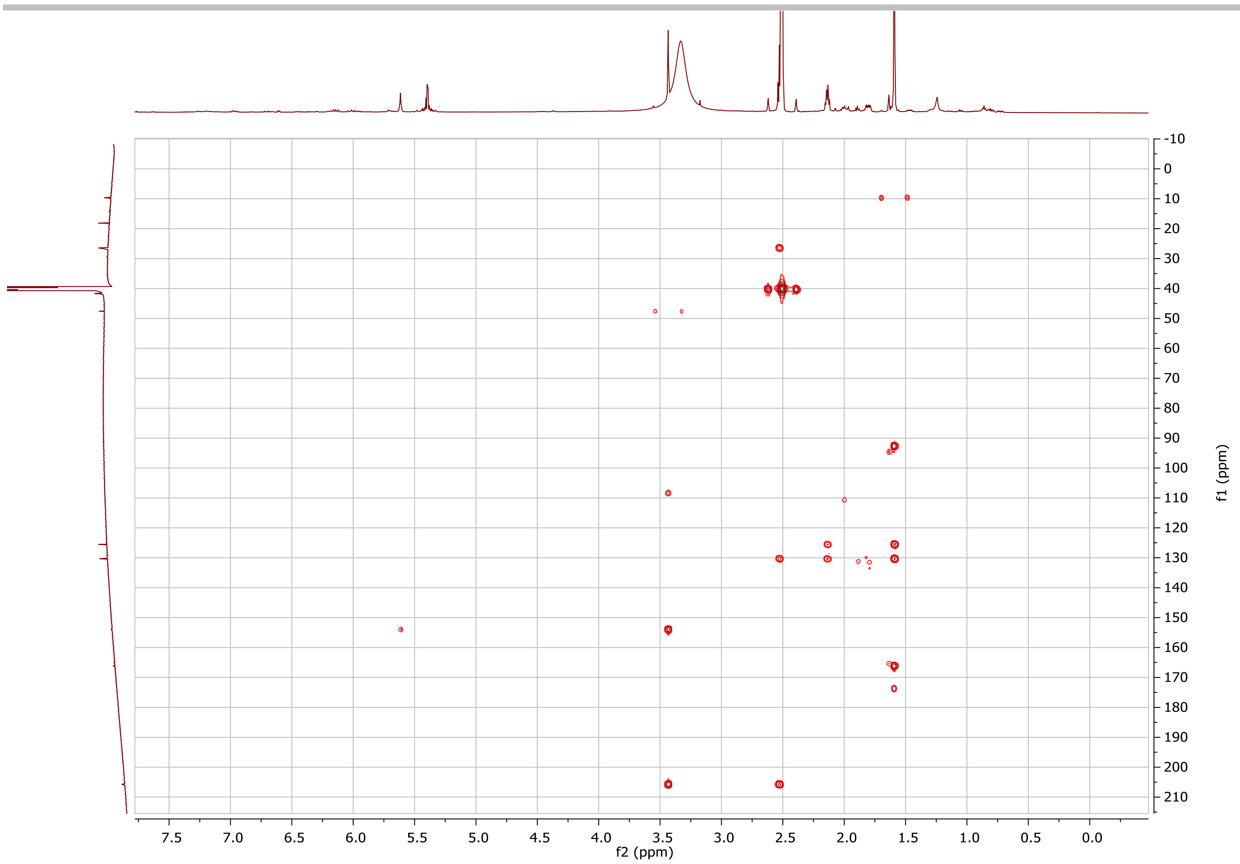

Figure S98: HMBC spectrum for **14** recorded at 600 MHz in DMSO-d6.

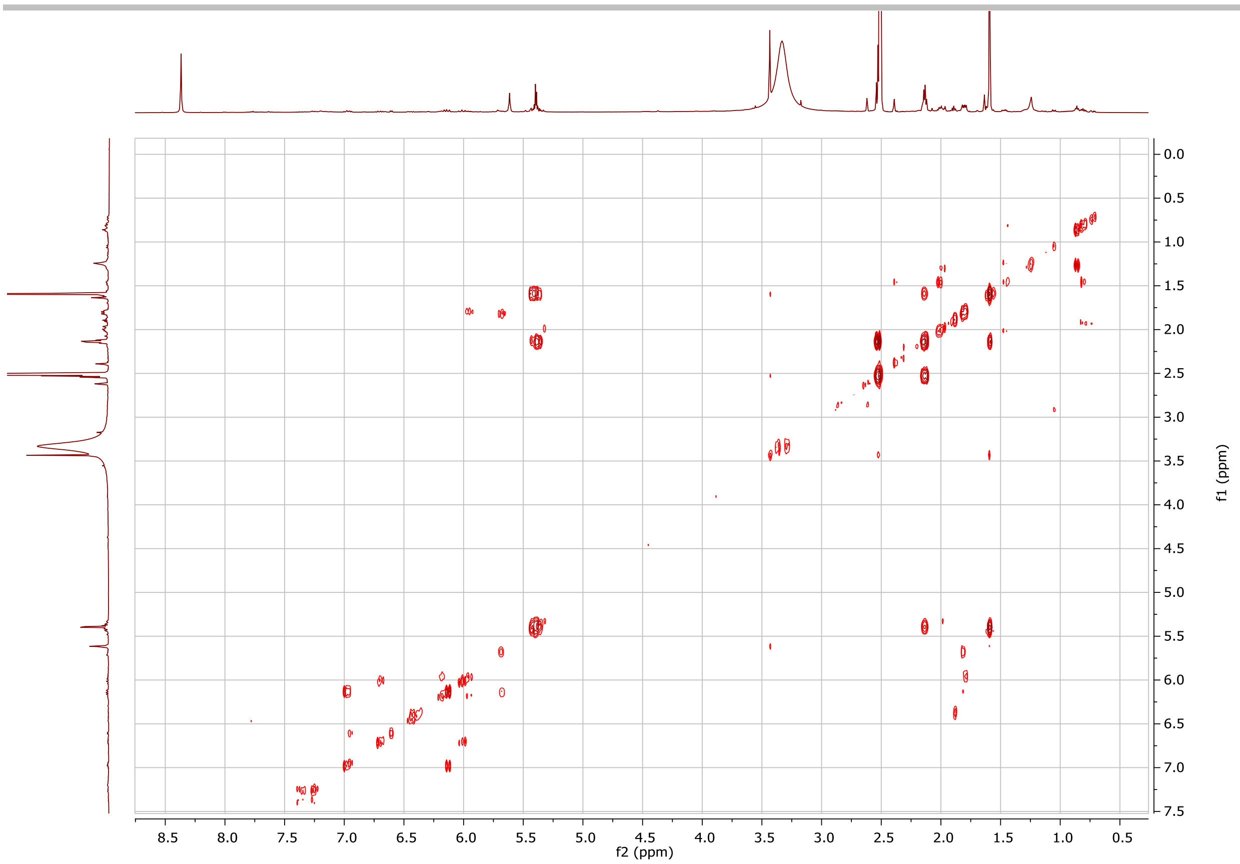

Figure S99: ^{1}H-^{1}H COSY spectrum for **14** recorded at 600 MHz in DMSO-d6.

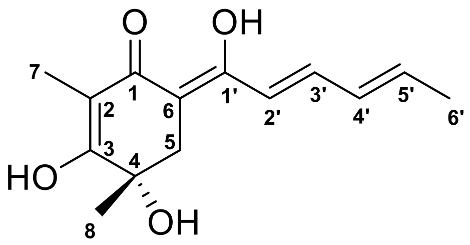

reduced sorbicillinol **15a**

Chemical Formula: $C_{14}H_{18}O_4$

Exact Mass: 250,1205

UV$_{\lambda max}$ (MeOH): 222 nm, 265 nm, 374 nm

HRMS (ESI) *m/z* (M-H)$^-$ calcd for $C_{14}H_{17}O_4$: 249.1127, found: 249.1135

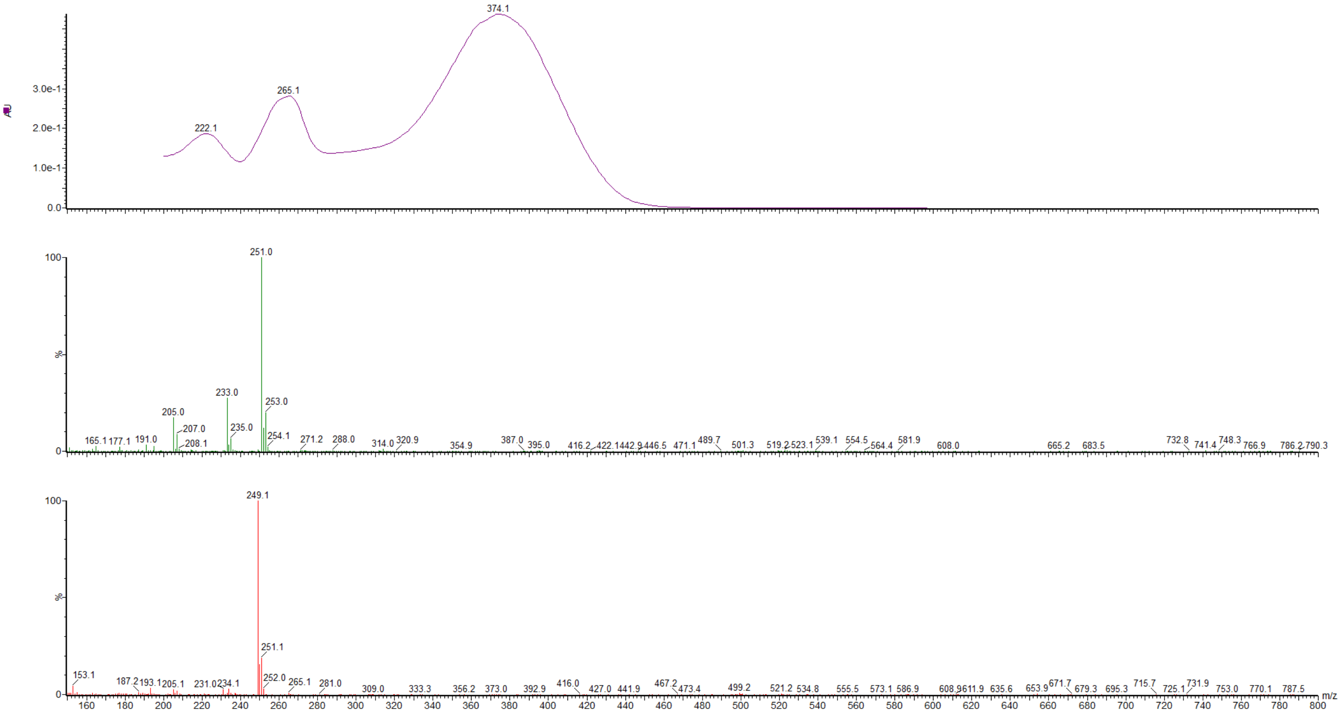

Figure S100: UV-absorption (top) and fragmentation pattern of **15a** in ES+ TIC (middle) and ES- TIC (bottom).

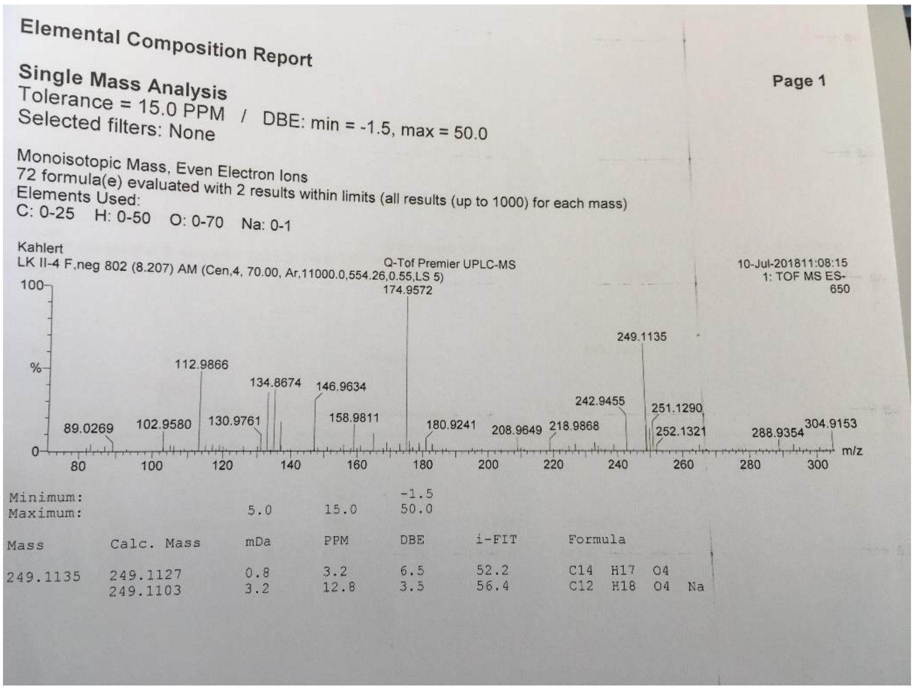

Elemental Composition Report

Page 1

Single Mass Analysis
Tolerance = 15.0 PPM / DBE: min = -1.5, max = 50.0
Selected filters: None

Monoisotopic Mass, Even Electron Ions
72 formula(e) evaluated with 2 results within limits (all results (up to 1000) for each mass)
Elements Used:
C: 0-25 H: 0-50 O: 0-70 Na: 0-1

Kahlert
LK II-4 F.neg 802 (8.207) AM (Cen,4, 70.00, Ar,11000.0,554.26,0.55,LS 5) Q-Tof Premier UPLC-MS 10-Jul-201811:08:15 1: TOF MS ES- 650

Minimum:				-1.5	
Maximum:		5.0	15.0	50.0	

Mass	Calc. Mass	mDa	PPM	DBE	i-FIT	Formula
249.1135	249.1127	0.8	3.2	6.5	52.2	C14 H17 O4
	249.1103	3.2	12.8	3.5	56.4	C12 H18 O4 Na

Figure S101: HRMS data for **15a**.

Table S17: Summarized NMR data for **15a** recorded in DMSO-d6.

Position	δ_C	δ_H	M	$J_{H\text{-}H}$/Hz	HSQC	HMBC H to C	H-H COSY
1	190.9	-	-	-	-	-	-
2	106.1	-	-	-	-	-	-
3	175.9	-	-	-	-	-	-
4	69.5	-	-	-	-	-	-
5a	37.2	2.56	d	14.3	CH_2	4, 6, 8, 1‘	5b
5b	37.2	2.72	d	14.3	CH_2	1, 3, 4, 6, 8, 1‘	5a
6	103.2	-	-	-	-	-	-
7	7.7	1.61	s	-	-	-	-
8	25.8	1.19	s	-	-	-	-
1‘	164.6	-	-	-	-	-	-
2‘	121.0	6.42	d	14.9	CH	6, 1‘, 4‘	3‘
3‘	136.7	6.97	dd	11.0, 14.9	CH	1‘, 2‘, 4‘, 4‘	2‘, 4‘
4‘	131.1	6.33	m	-	CH	2‘, 3‘, 6‘	3‘, 5‘, 6‘
5‘	135.4	6.06	dq	6.8, 14.0	CH	3‘, 6‘	4‘, 6‘
6‘	18.4	1.82	d	6.8	CH_3	4‘, 5‘	4‘, 5‘

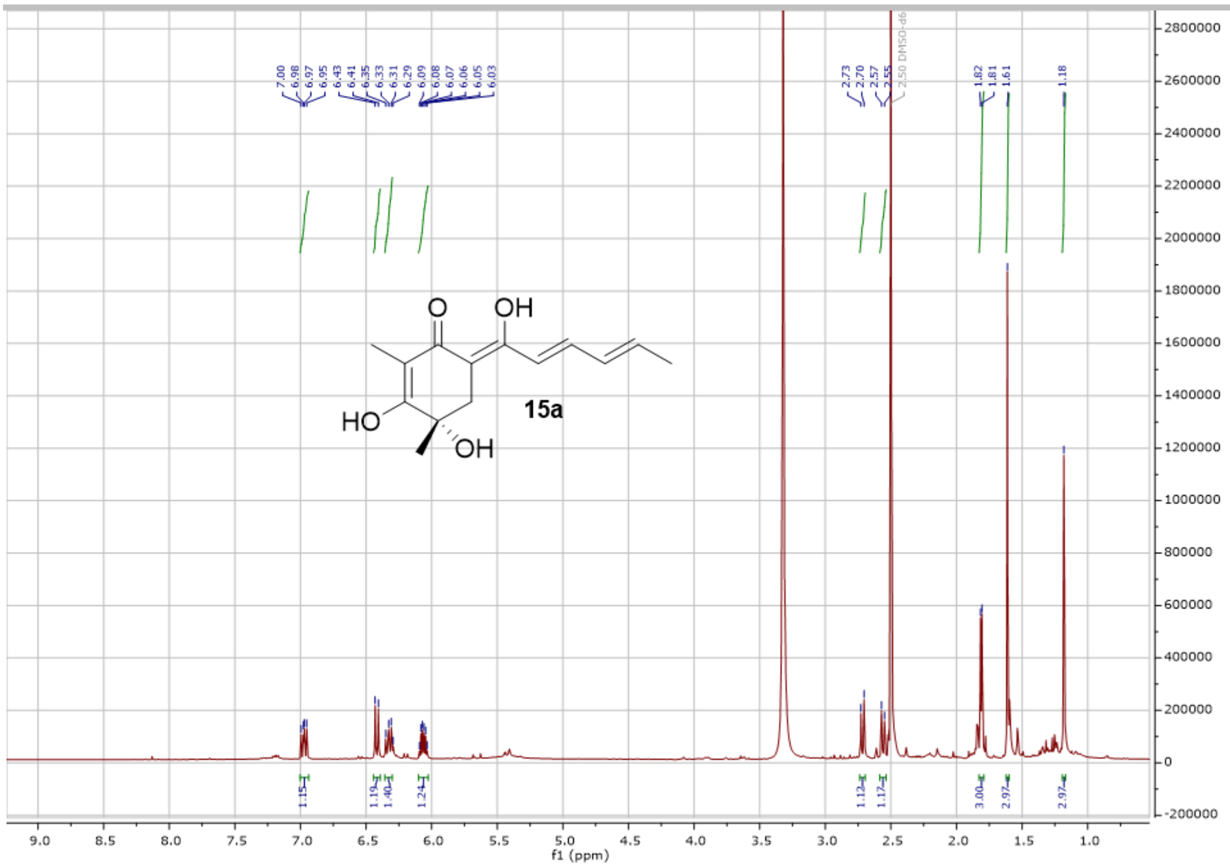

Figure S102: ^{1}H-NMR spectrum for **15a** recorded at 600 MHz in DMSO-d6.

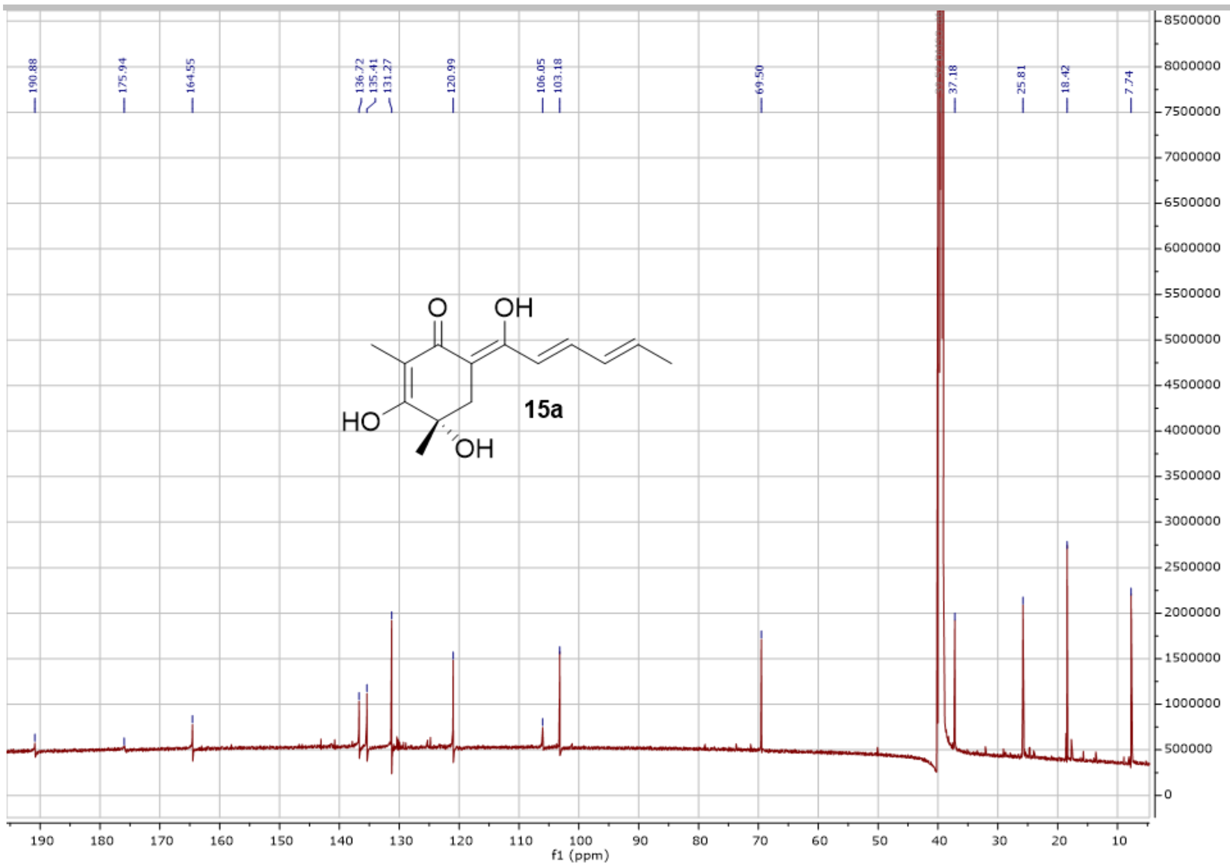

Figure S103: ^{13}C-NMR spectrum for **15a** recorded at 125 MHz in DMSO-d6.

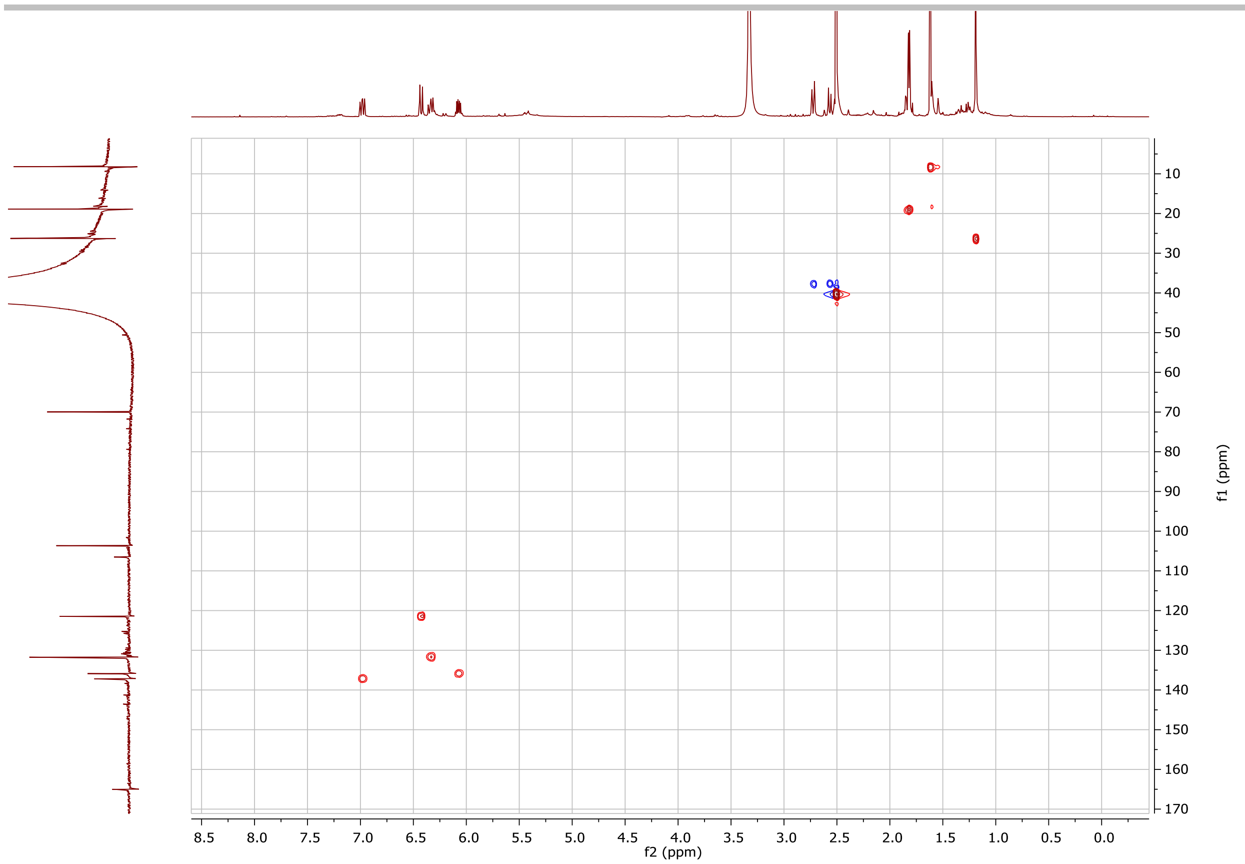

Figure S104: HSQC spectrum for **15a** recorded at 600 MHz in DMSO-d6.

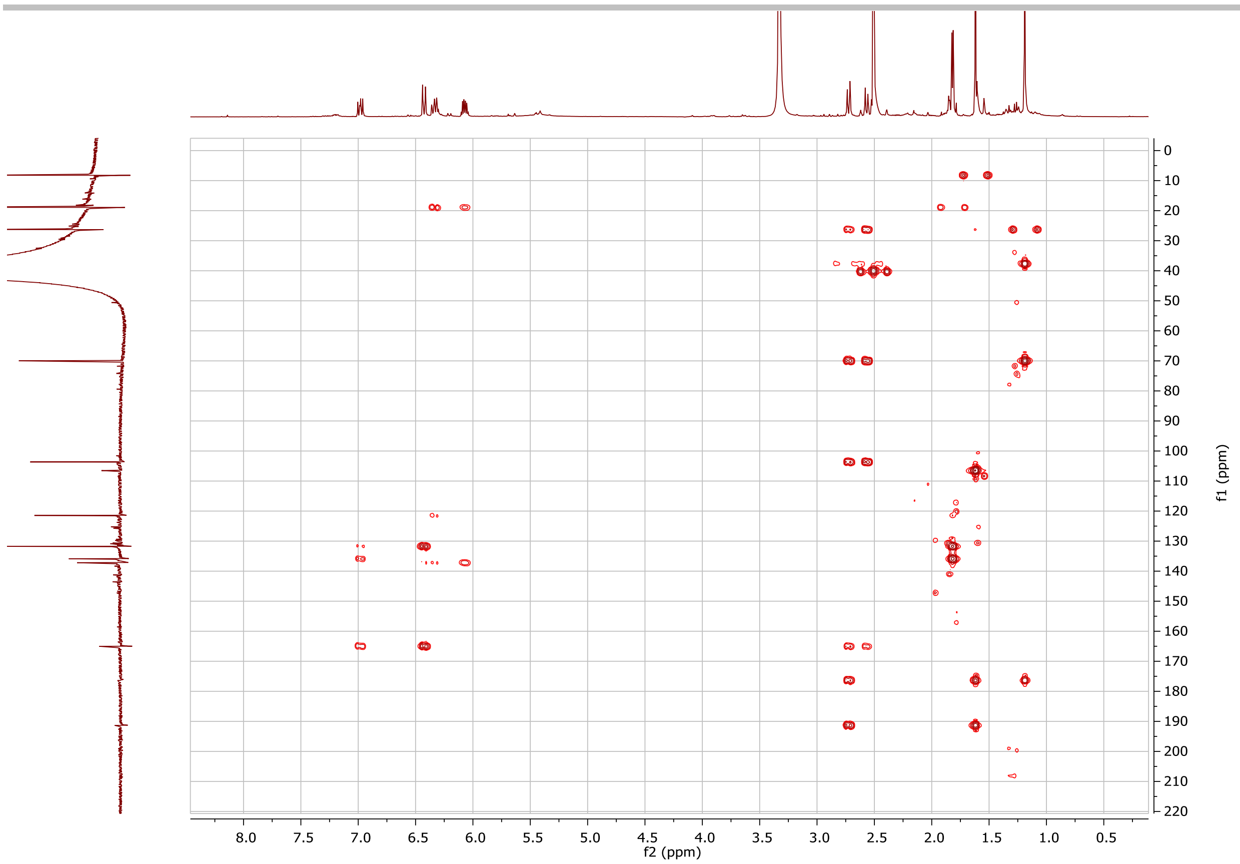

Figure S105: HMBC spectrum for **15a** recorded at 600 MHz in DMSO-d6.

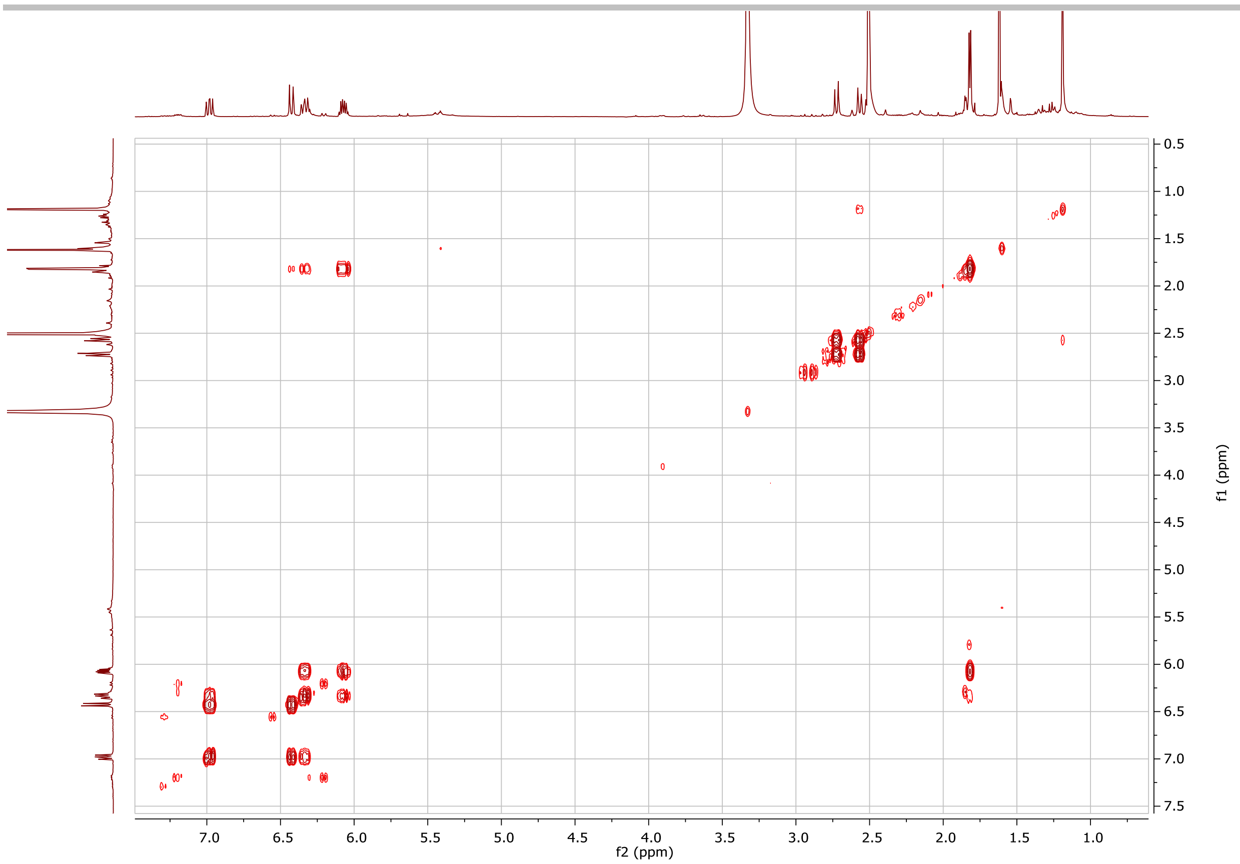

Figure S106: ^{1}H-^{1}H COSY spectrum for **15a** recorded at 600 MHz in DMSO-d6.

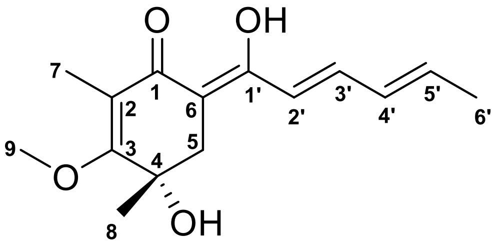

monomethylated reduced sorbicillinol **15a***

Chemical Formula: $C_{15}H_{20}O_4$
Exact Mass: 264,1362

Table S18: Summarized NMR data for **15a*** recorded in DMSO-d6.

Position	δ_C	δ_H	M	J_{H-H}/Hz	HSQC	HMBC H to C	H-H COSY
1	190.8	-	-	-	-	-	-
2	115.9	-	-	-	-	-	-
3	173.8	-	-	-	-	-	-
4	69.9	-	-	-	-	-	-
5	38.5	2.66	d	5.1	CH_2	1, 3, 4, 6, 8, 1‘	5
6	103.3	-	-	-	-	-	-
7	8.8	1.70	s	-	CH_3	1, 2, 3	-
8	25.9	1.29	s	-	CH_3	3, 4, 5	-
9	60.4	3.97	s	-	CH_3	3	-
1‘	166.8	-	-	-	-	-	-
2‘	120.6	6.44	d	14.8	CH	8, 1‘, 4‘	3‘
3‘	138.3	7.06	dd	11.0, 14.9	CH	1‘, 4‘, 5‘	2‘, 4‘
4‘	131.1	6.35	m	-	CH	2‘, 6‘	3‘, 5‘
5‘	136.8	6.13	dq	6.8, 14.0	CH	3‘, 6‘	4‘, 6‘
6‘	18.5	1.82	d	6.9	CH_3	2‘, 4‘, 5‘	5‘

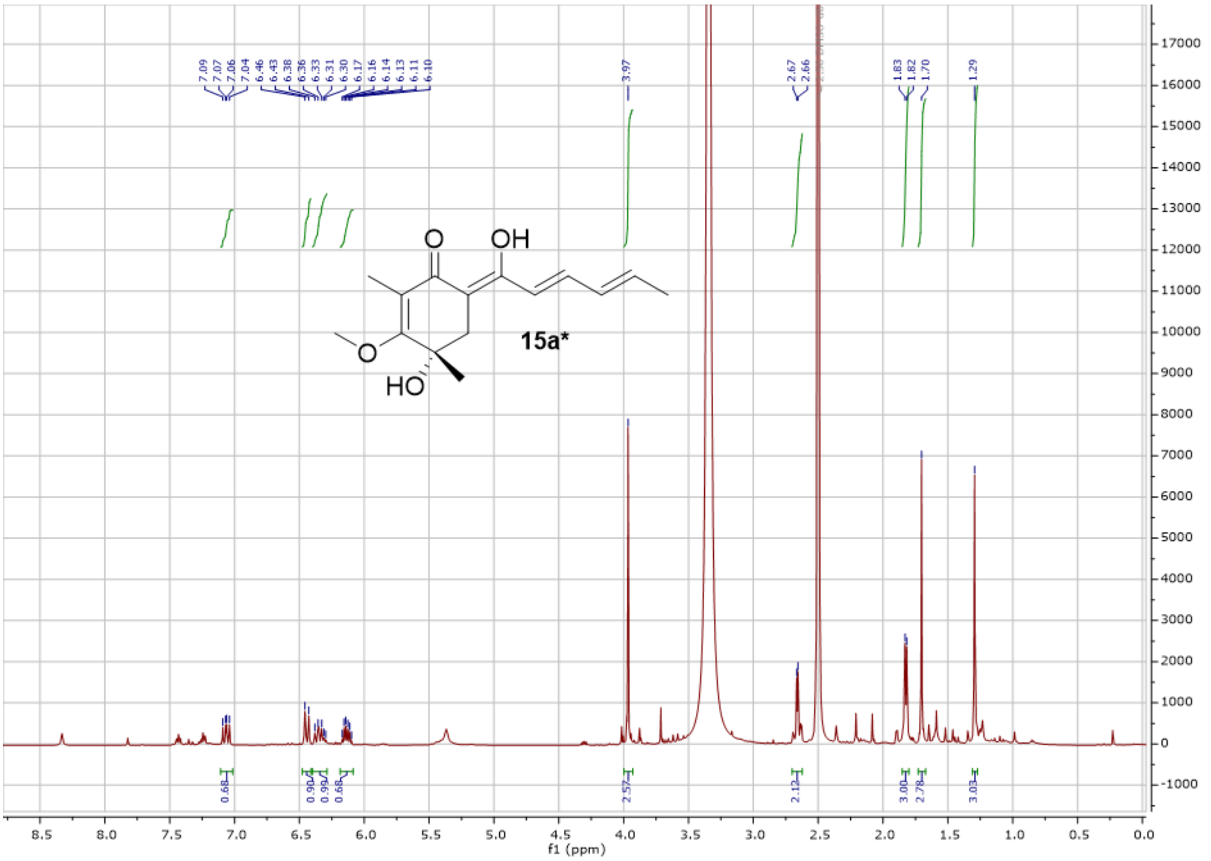

Figure S107: ^{1}H-NMR spectrum for **15a*** recorded at 500 MHz in DMSO-d6.

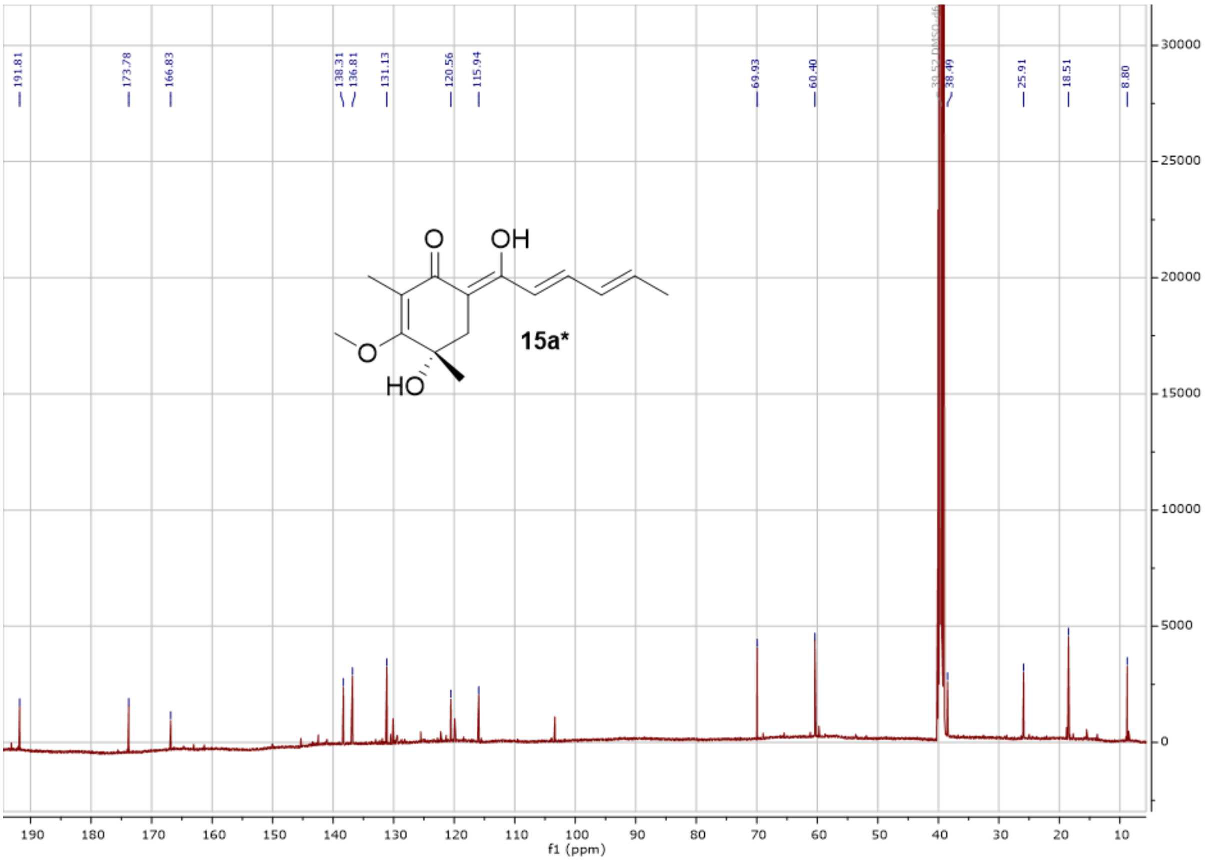

Figure S108: ^{13}C-NMR spectrum for **15a*** recorded at 125 MHz in DMSO-d6.

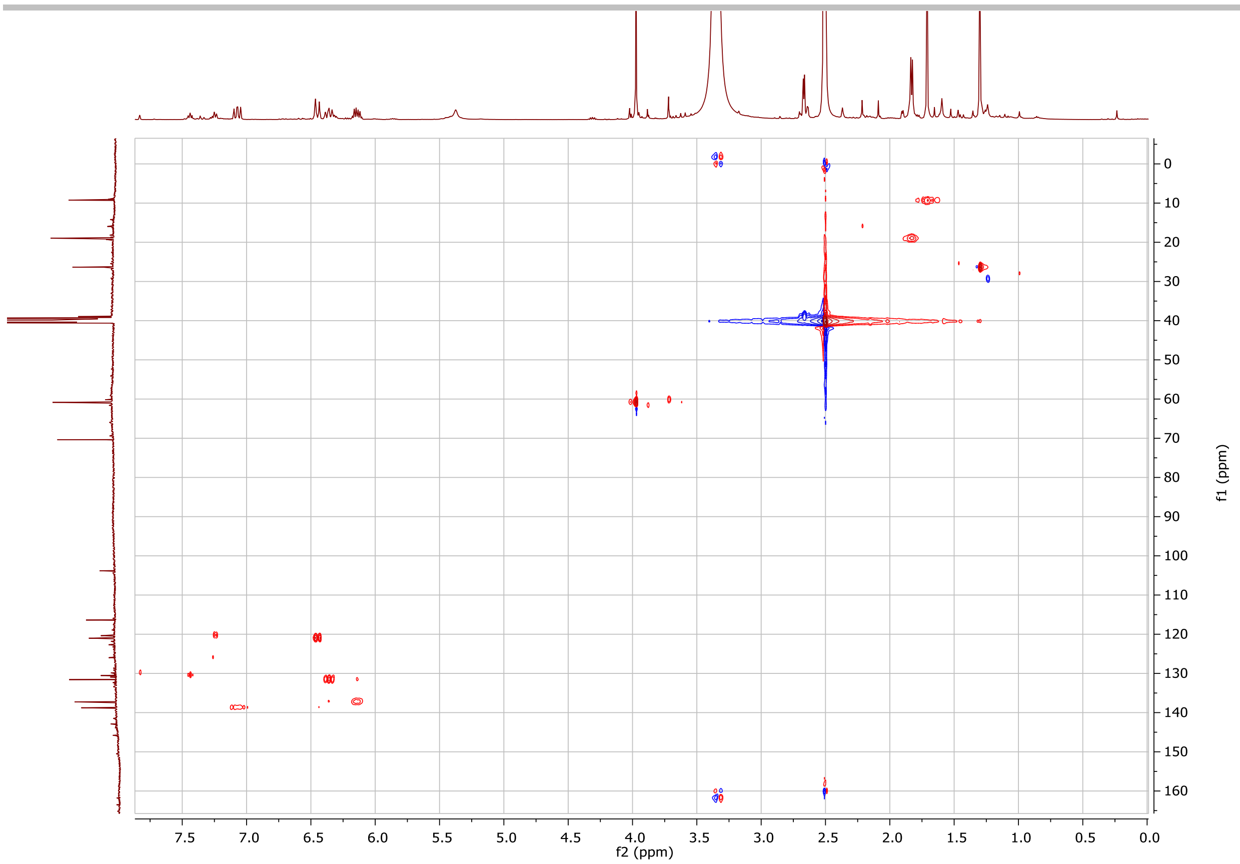

Figure S109: HSQC spectrum for **15a*** recorded at 500 MHz in DMSO-d6.

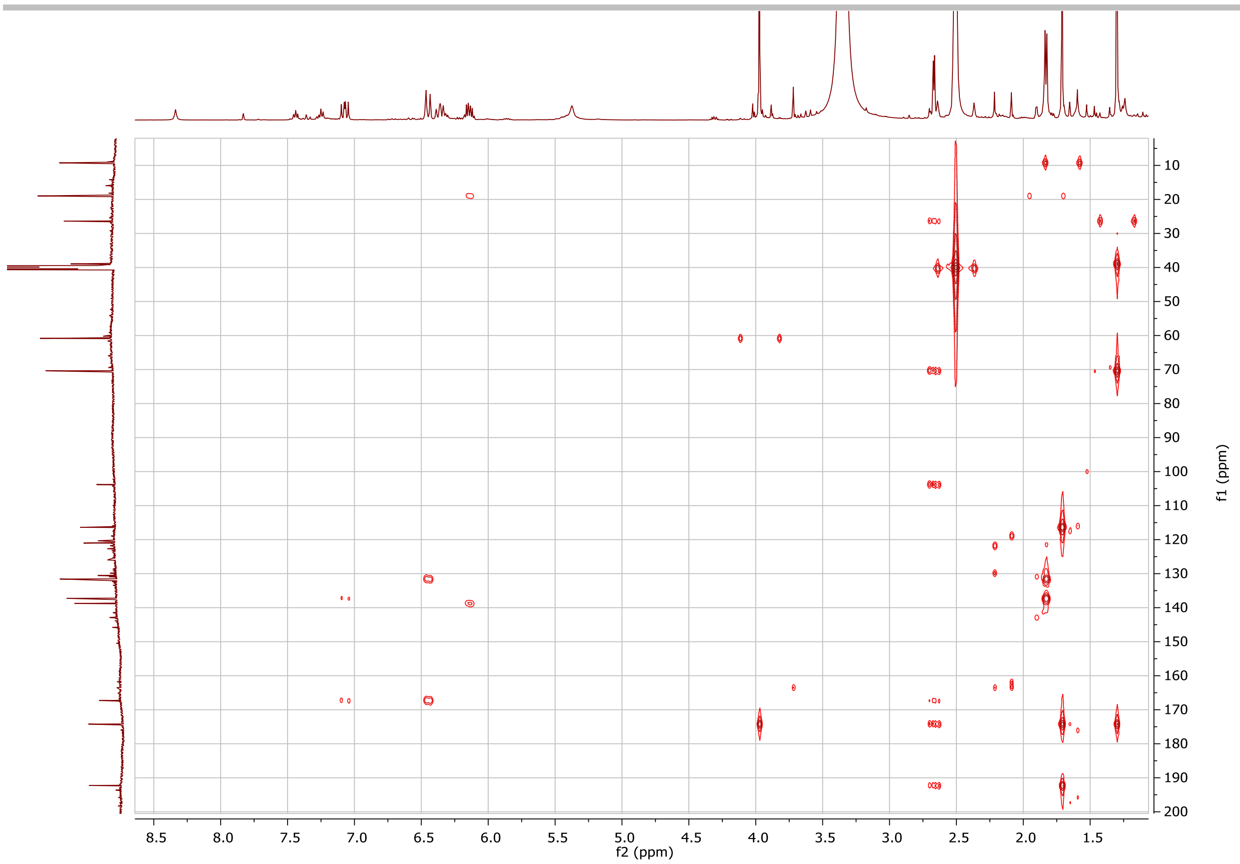

Figure S110: HMBC spectrum for **15a*** recorded at 500 MHz in DMSO-d6.

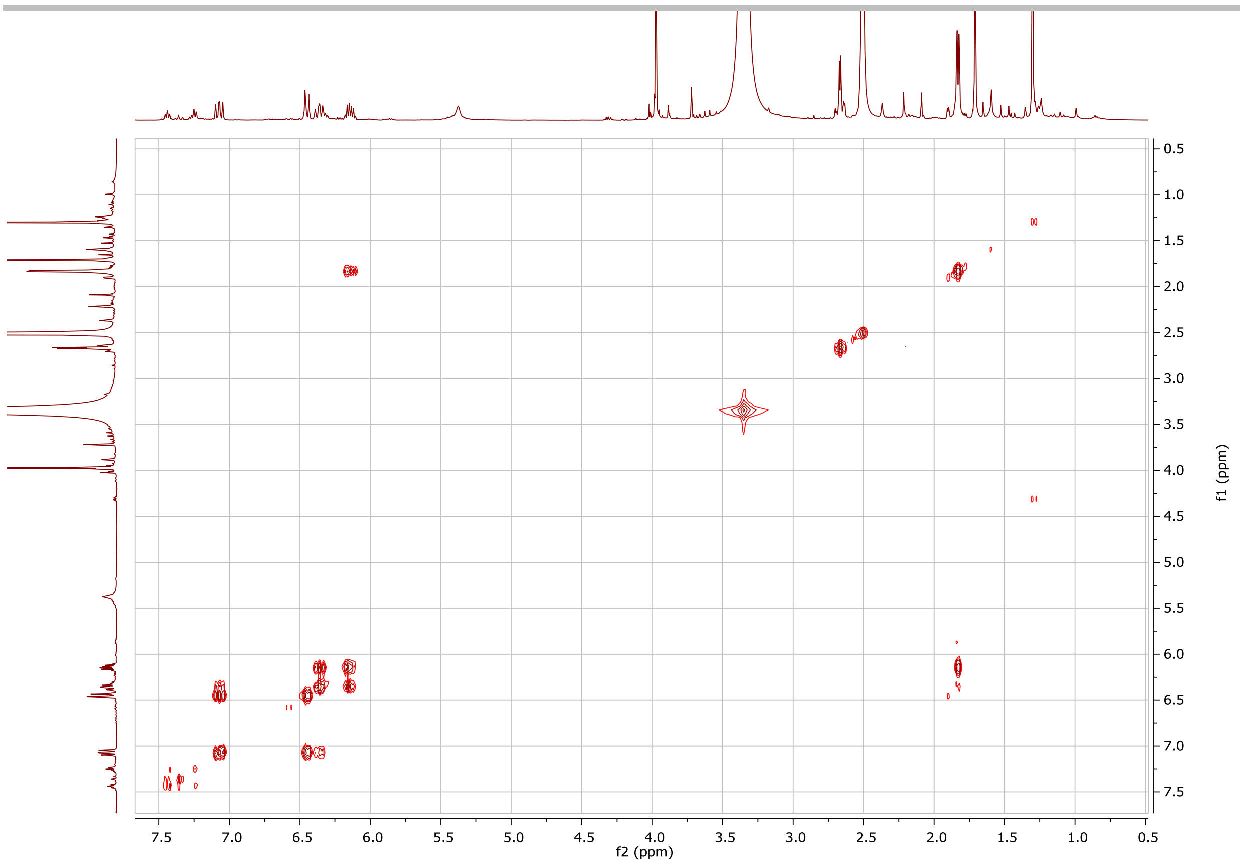

Figure S111: ^{1}H-^{1}H COSY spectrum for **15a*** recorded at 500 MHz in DMSO-d6.

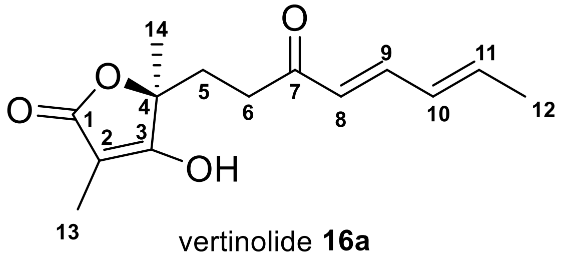

vertinolide **16a**

Chemical Formula: $C_{14}H_{18}O_4$
Exact Mass: 250,1205

UV$_{\lambda max}$ (MeOH): 232 nm, 274 nm, 358 nm
HRMS (ESI) *m/z* (*M*-H)$^-$ calcd for $C_{14}H_{18}O_4$Na: 273.1127, found: 273.1132

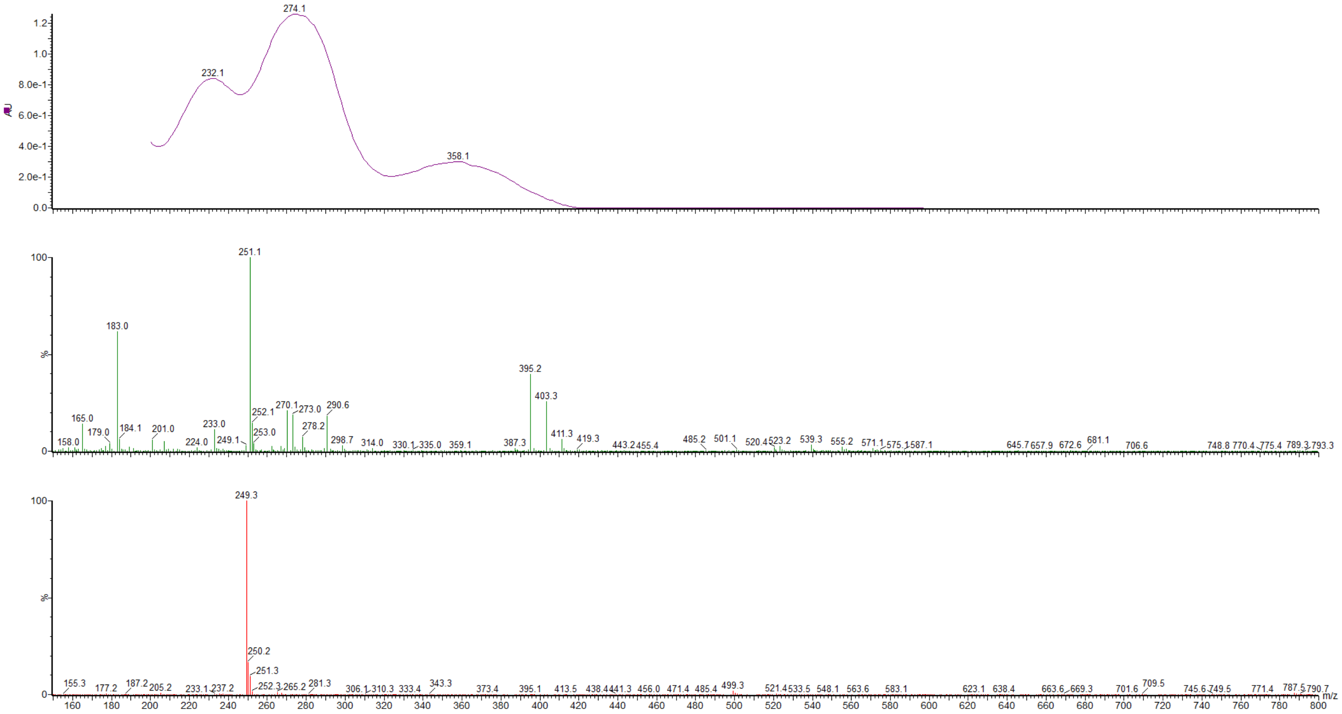

Figure S112: UV-absorption (top) and fragmentation pattern of **16a** in ES+ TIC (middle) and ES- TIC (bottom).

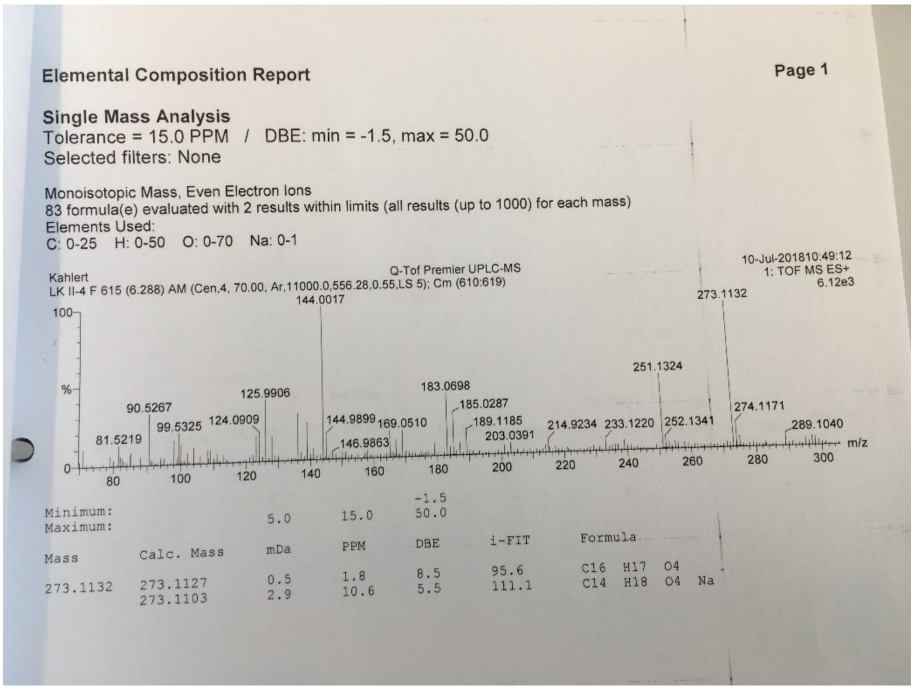

Elemental Composition Report

Page 1

Single Mass Analysis
Tolerance = 15.0 PPM / DBE: min = -1.5, max = 50.0
Selected filters: None

Monoisotopic Mass, Even Electron Ions
83 formula(e) evaluated with 2 results within limits (all results (up to 1000) for each mass)
Elements Used:
C: 0-25 H: 0-50 O: 0-70 Na: 0-1

Kahlert
LK II-4 F 615 (6.288) AM (Cen,4, 70.00, Ar,11000.0,556.28,0.55,LS 5); Cm (610:619)
Q-Tof Premier UPLC-MS
10-Jul-201810:49:12
1: TOF MS ES+
6.12e3

Mass	Calc. Mass	mDa	PPM	DBE	i-FIT	Formula
Minimum:				-1.5		
Maximum:		5.0	15.0	50.0		
273.1132	273.1127	0.5	1.8	8.5	95.6	C16 H17 O4
	273.1103	2.9	10.6	5.5	111.1	C14 H18 O4 Na

Figure S113: HRMS data for **16a**.

Table S19: Summarized NMR data for **16a** recorded at 400 MHz in DMSO-d6.

Position	δ_C	δ_H	M	$J_{H\text{-}H}$/Hz	HSQC	HMBC H to C	H-H COSY
1	174.9	-	-	-	-	-	-
2	97.5	-	-	-	-	-	-
3	176.4	-	-	-	-	-	-
4	82.8	-	-	-	-	-	-
5	30.9	2.14	m	-	CH_2	3, 4, 6, 7, 14	6
6	34.0	2.48, 2.66	m, m	-	CH_2	4, 5, 7	5
7	201.8	-	-	-	-	-	-
8	127.0	6.04	d	15.5	CH	6, 7, 9, 10	9, 10
9	145.4	7.17	dd	10.5, 15.5	CH	7, 10, 11	8, 10
10	142.7	6.26	m	-	CH	9, 11, 12	9, 11, 12
11	130.2	6.20	m	-	CH	9, 10, 12	9, 10, 12
12	19.1	1.88	d	6.7	CH_3	9, 10, 11	10, 11
13	6.1	1.71	s	-	CH_3	1, 2, 3	-
14	23.4	1.48	s	-	CH_3	3, 4, 5	-

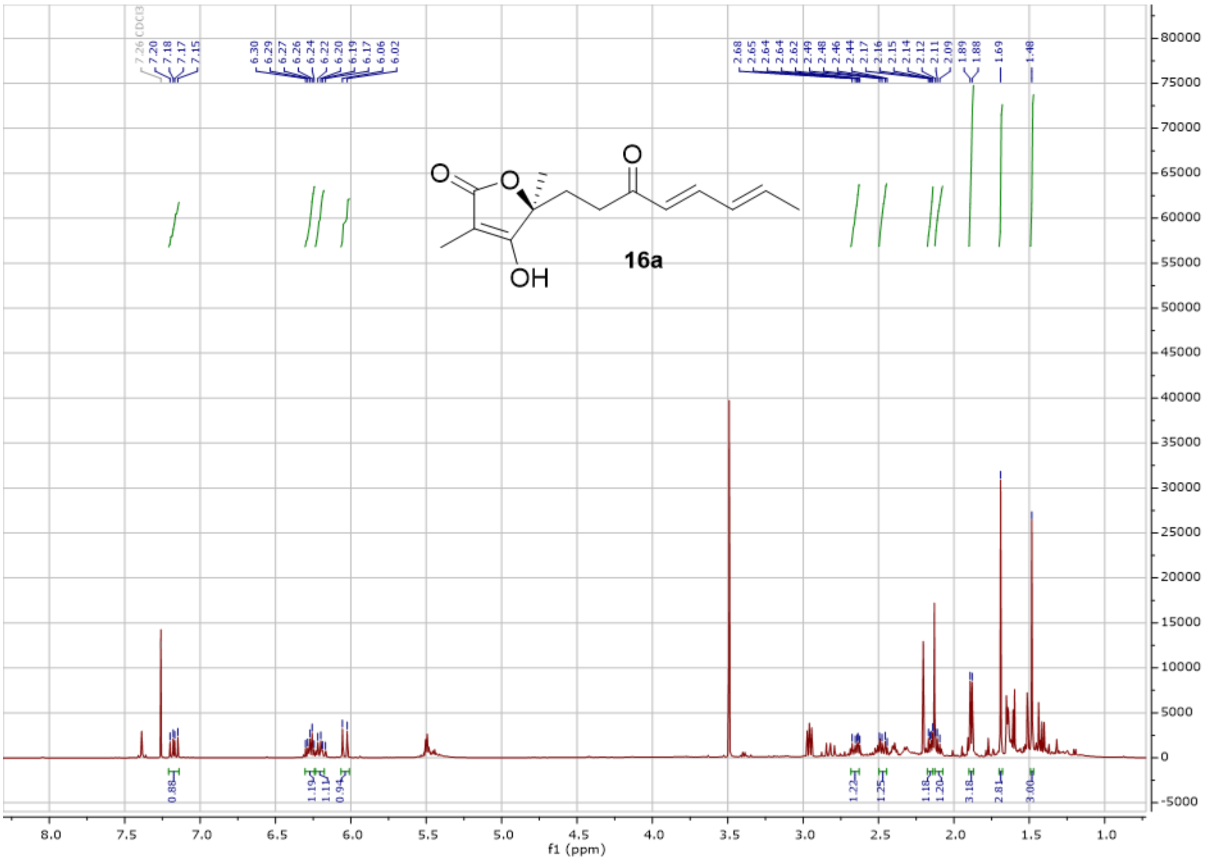

Figure S114: ^{1}H-NMR spectrum for **16a** recorded at 400 MHz in DMSO-d6.

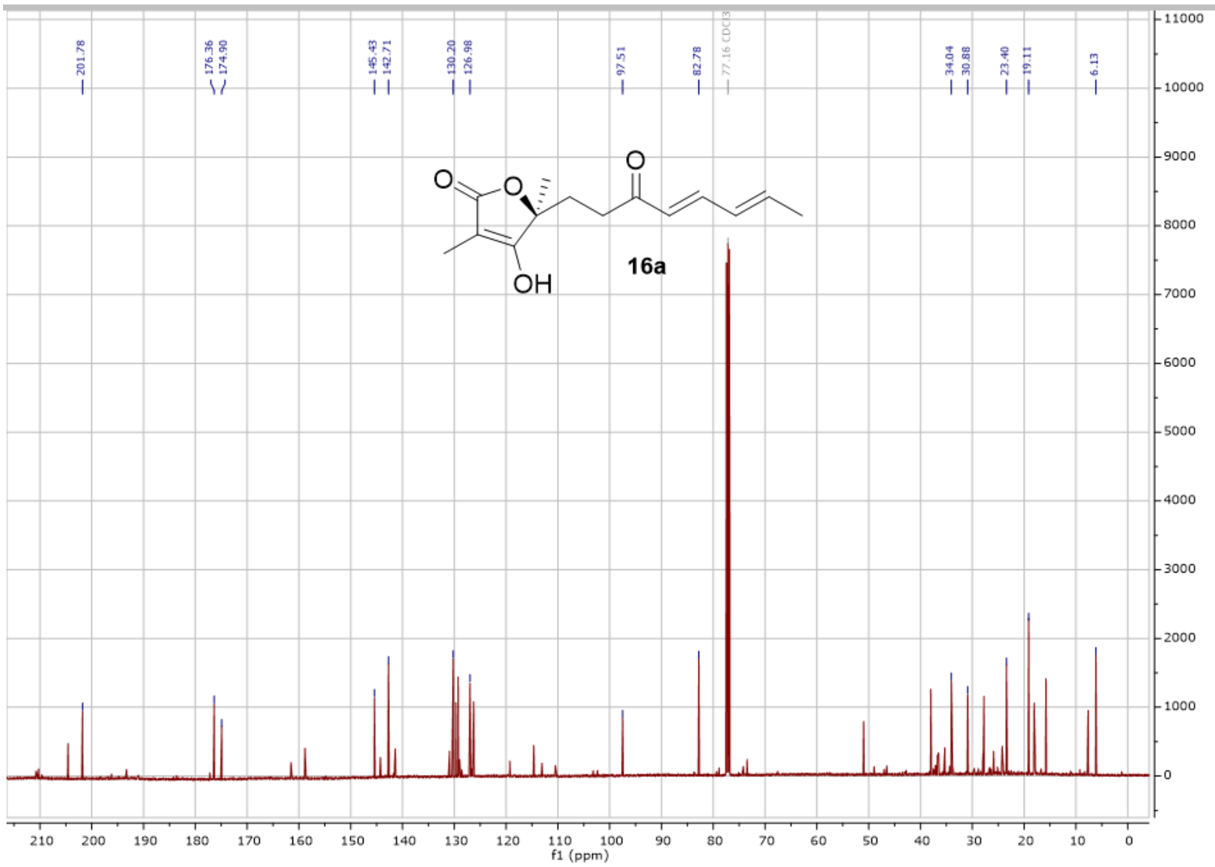

Figure S115: ^{13}C-NMR spectrum for **16a** recorded at 125 MHz in DMSO-d6.

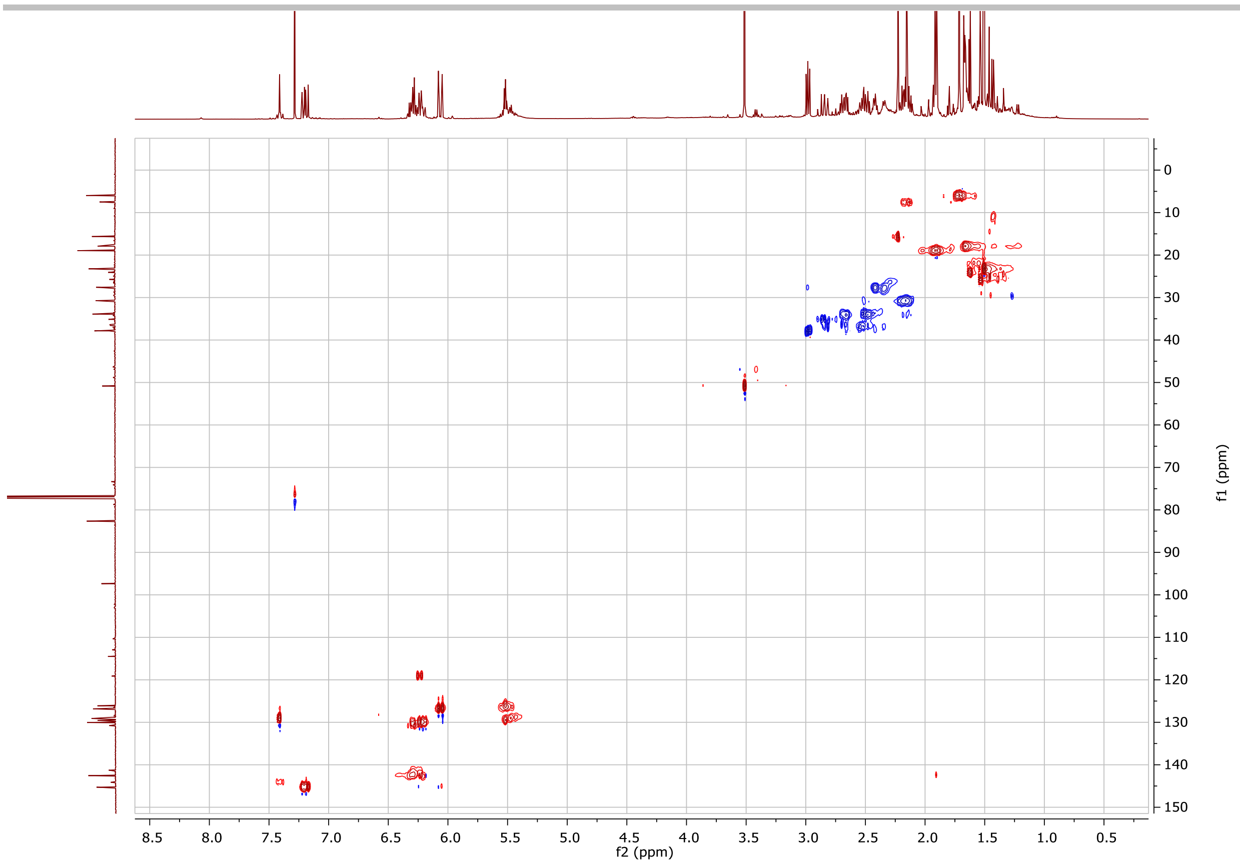

Figure S116: HSQC spectrum for **16a** recorded at 400 MHz in DMSO-d6.

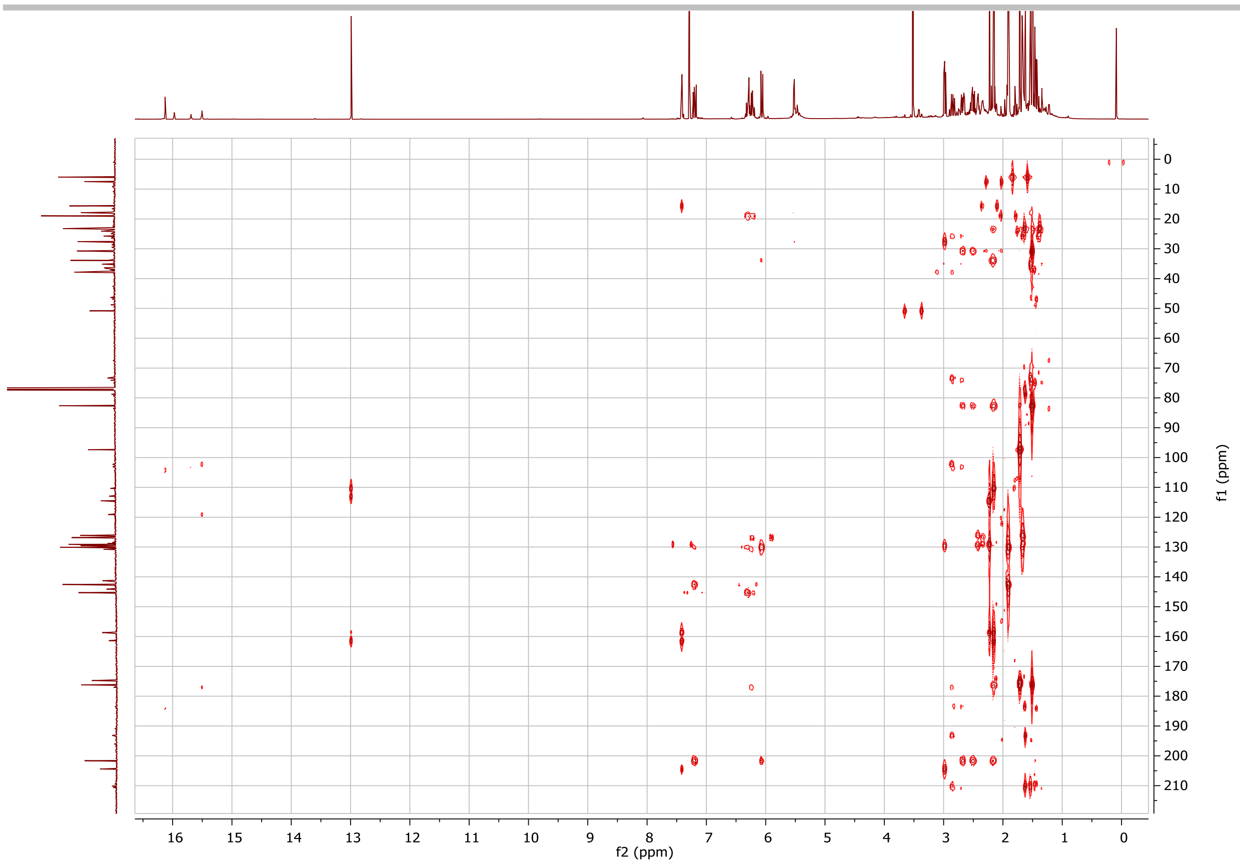

Figure S117: HMBC spectrum for **16a** recorded at 400 MHz in DMSO-d6.

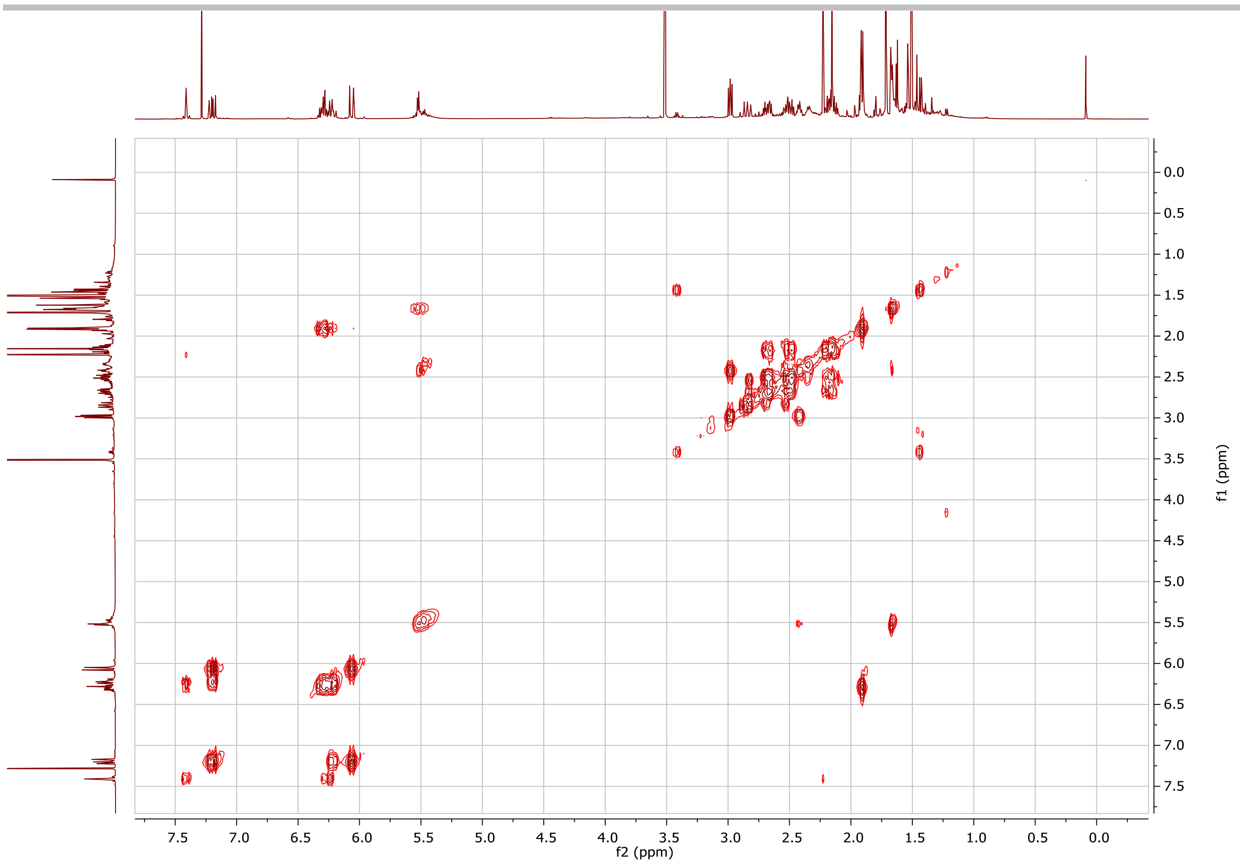

Figure S118: ^{1}H-^{1}H COSY spectrum for **16a** recorded at 400 MHz in DMSO-d6.

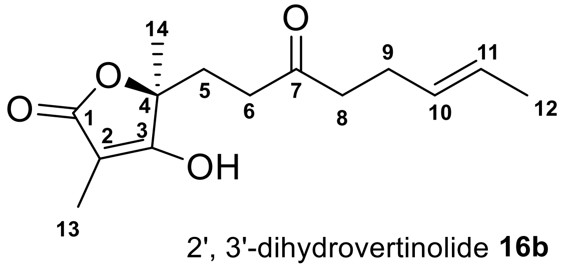

2', 3'-dihydrovertinolide **16b**

Chemical Formula: $C_{14}H_{20}O_4$

Exact Mass: 252,1362

UV$_{\lambda max}$ (MeOH): 226 nm, 274 nm, 367 nm

HRMS (ESI) *m/z* (*M*-H)$^-$ calcd for $C_{14}H_{21}O_4$: 253.1440, found: 253.1453

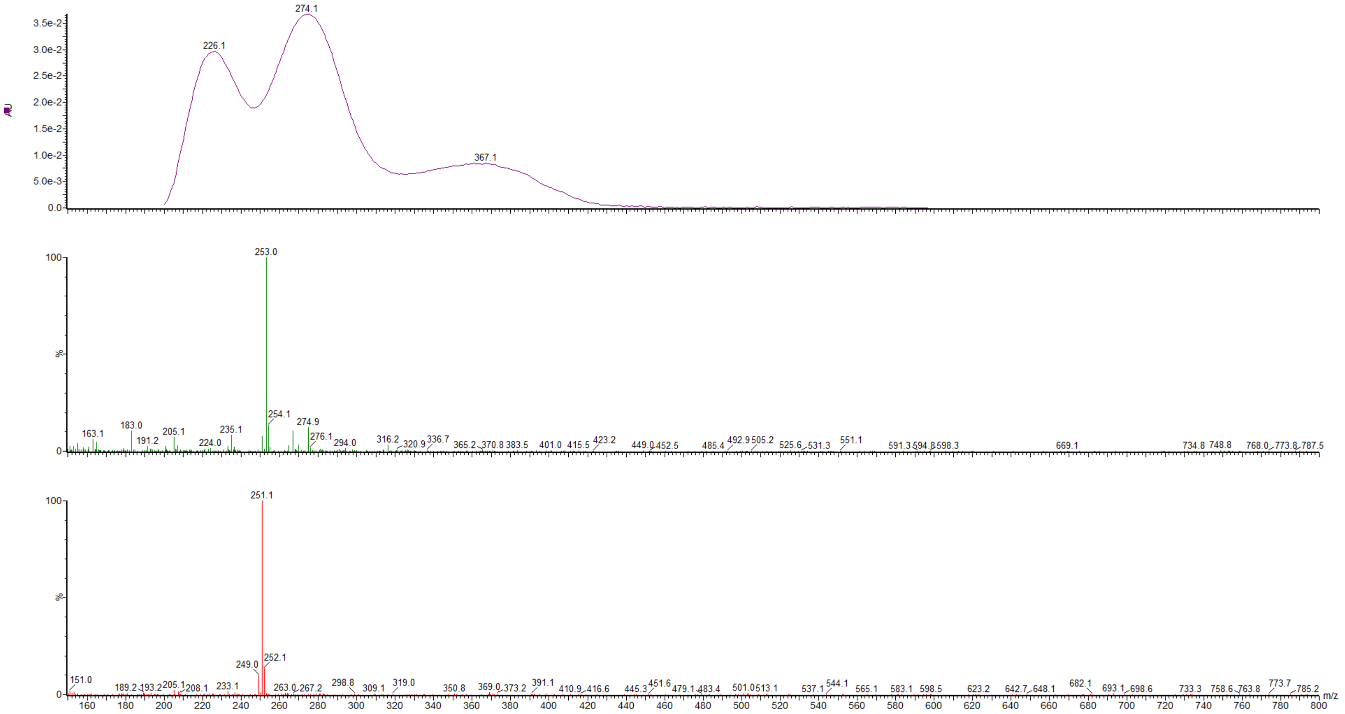

Figure S119: UV-absorption (top) and fragmentation pattern of **16b** in ES+ TIC (middle) and ES- TIC (bottom).

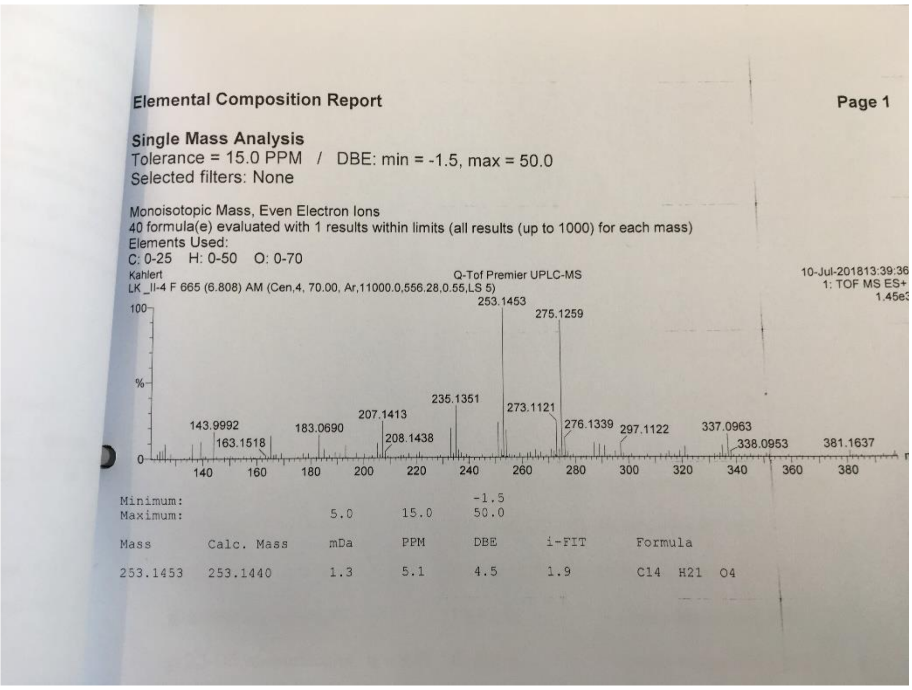

Elemental Composition Report

Page 1

Single Mass Analysis
Tolerance = 15.0 PPM / DBE: min = -1.5, max = 50.0
Selected filters: None

Monoisotopic Mass, Even Electron Ions
40 formula(e) evaluated with 1 results within limits (all results (up to 1000) for each mass)
Elements Used:
C: 0-25 H: 0-50 O: 0-70

Kahlert Q-Tof Premier UPLC-MS 10-Jul-201813:39:36
LK _II-4 F 665 (6.808) AM (Cen,4, 70.00, Ar,11000.0,556.28,0.55,LS 5) 1: TOF MS ES+ 1.45e3

Minimum:				-1.5		
Maximum:		5.0	15.0	50.0		
Mass	Calc. Mass	mDa	PPM	DBE	i-FIT	Formula
253.1453	253.1440	1.3	5.1	4.5	1.9	C14 H21 O4

Figure S120: HRMS data for **16b**.

Table S20: Summarized NMR data for **16b** recorded in DMSO-d6.

Position	δ_C	δ_H	M	$J_{H\text{-}H}$/Hz	HSQC	HMBC H to C	H-H COSY
1	174.5	-	-	-	-	-	-
2	97.8	-	-	-	-	-	-
3	176.0	-	-	-	-	-	-
4	82.6	-	-	-	-	-	-
5	30.0	2.08	m (broad)	-	CH_2	3, 4, 6, 7, 14	6
6	36.8	2.35, 2.48	m, m (broad)	-	CH_2	3, 4, 7	5
7	211.4	-	-	-	-	-	-
8	42.9	2.47	m (broad)	-	CH_2	7, 9, 10	9
9	26.9	2.22	m	-	CH_2	7, 8, 10, 11	8, 10
10	129.2	5.36	m	-	CH	9, 11, 12	9, 11
11	126.5	5.43	m	-	CH	8, 9, 10, 12	10, 12
12	18.1	1.64	d (broad)	9.3	CH_3	8, 9, 10, 11	10, 11, 9
13	6.1	1.71	s	-	CH_3	1, 2, 3	-
14	23.5	1.47	s	-	CH_3	3, 4, 5	

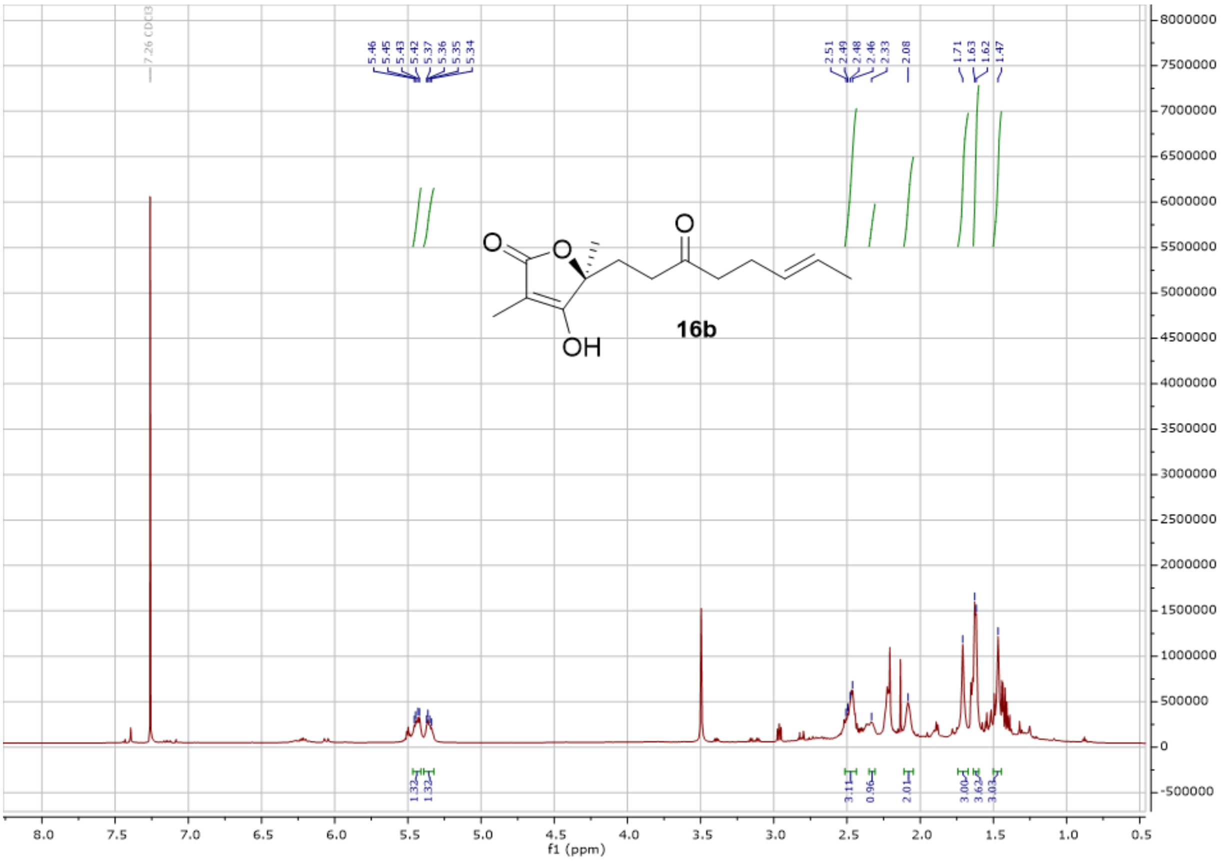

Figure S121: ^{1}H-NMR spectrum for **16b** recorded at 500 MHz in DMSO-d6.

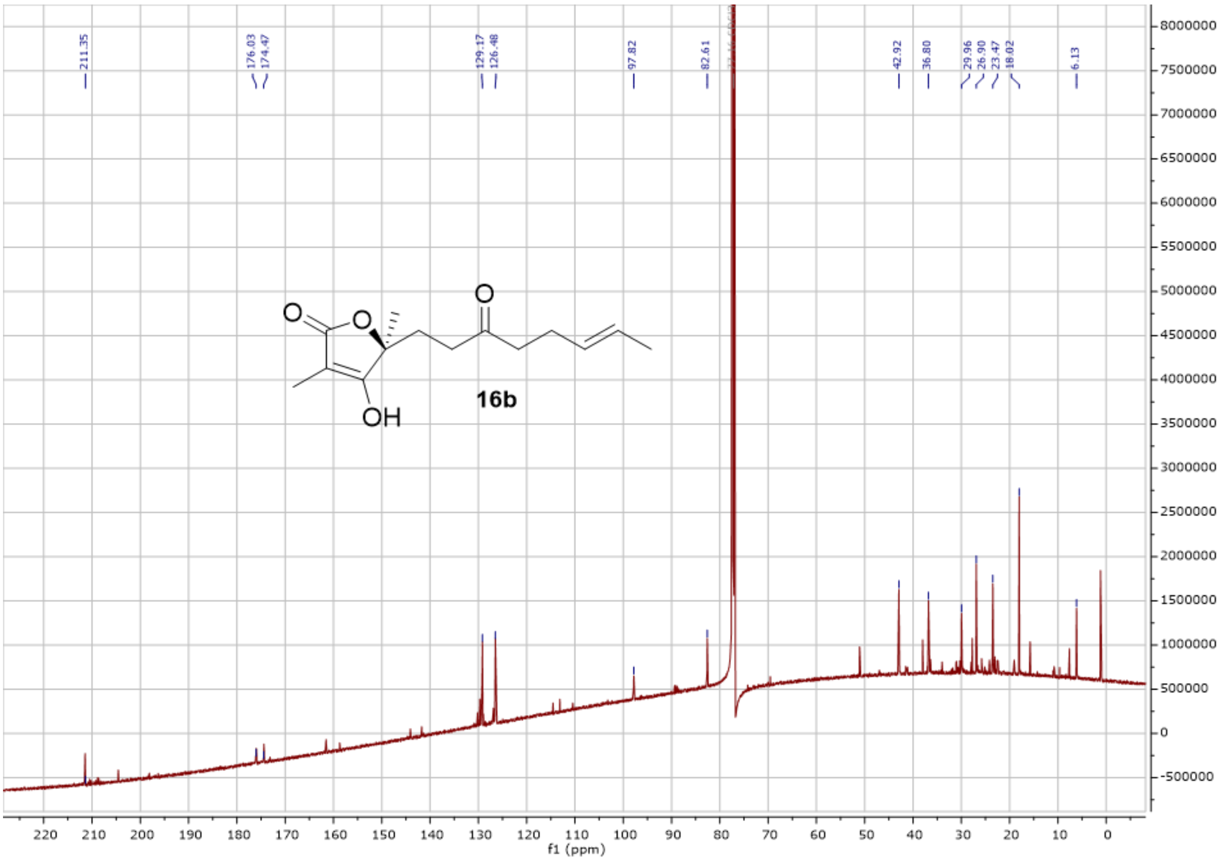

Figure S122: ^{13}C-NMR spectrum for **16b** recorded at 125 MHz in DMSO-d6.

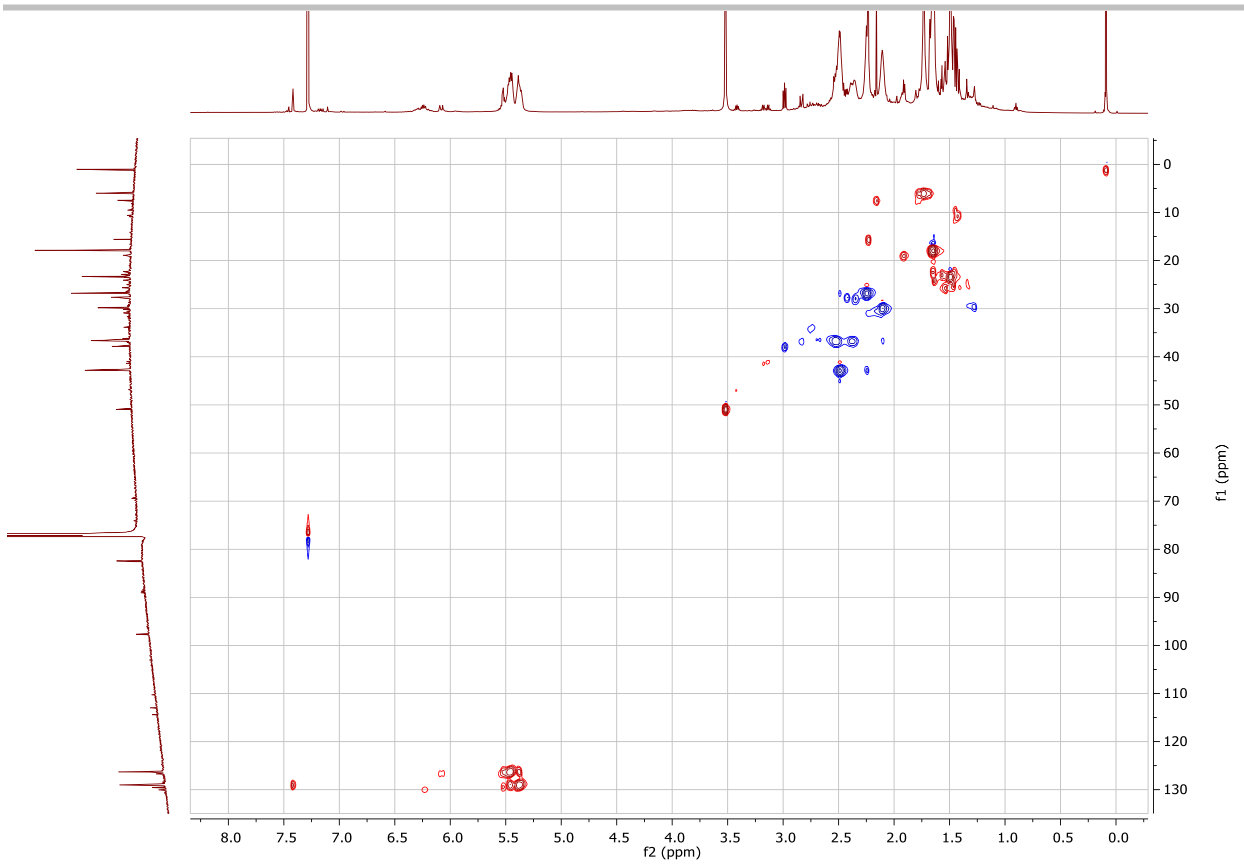

Figure S123: HSQC spectrum for **16b** recorded at 500 MHz in DMSO-d6.

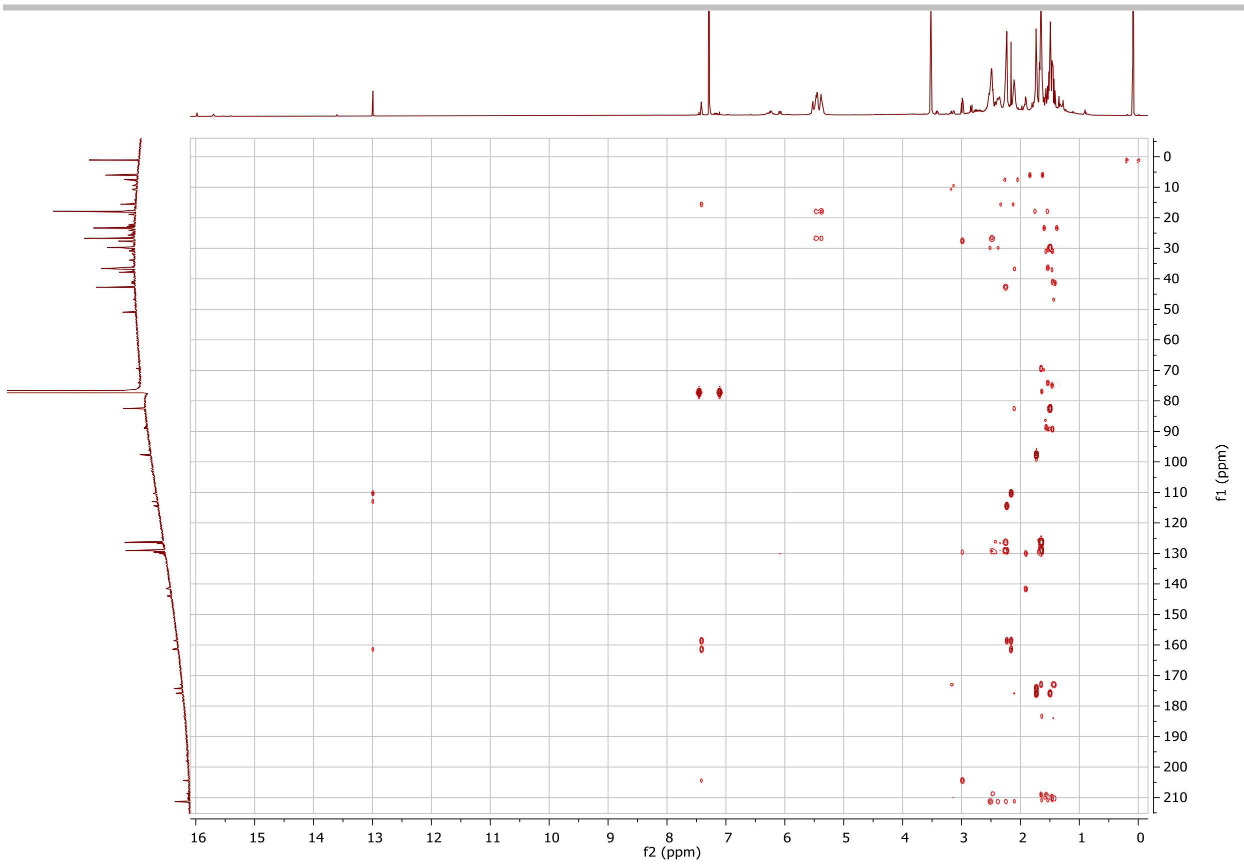

Figure S124: HMBC spectrum for **16b** recorded at 500 MHz in DMSO-d6.

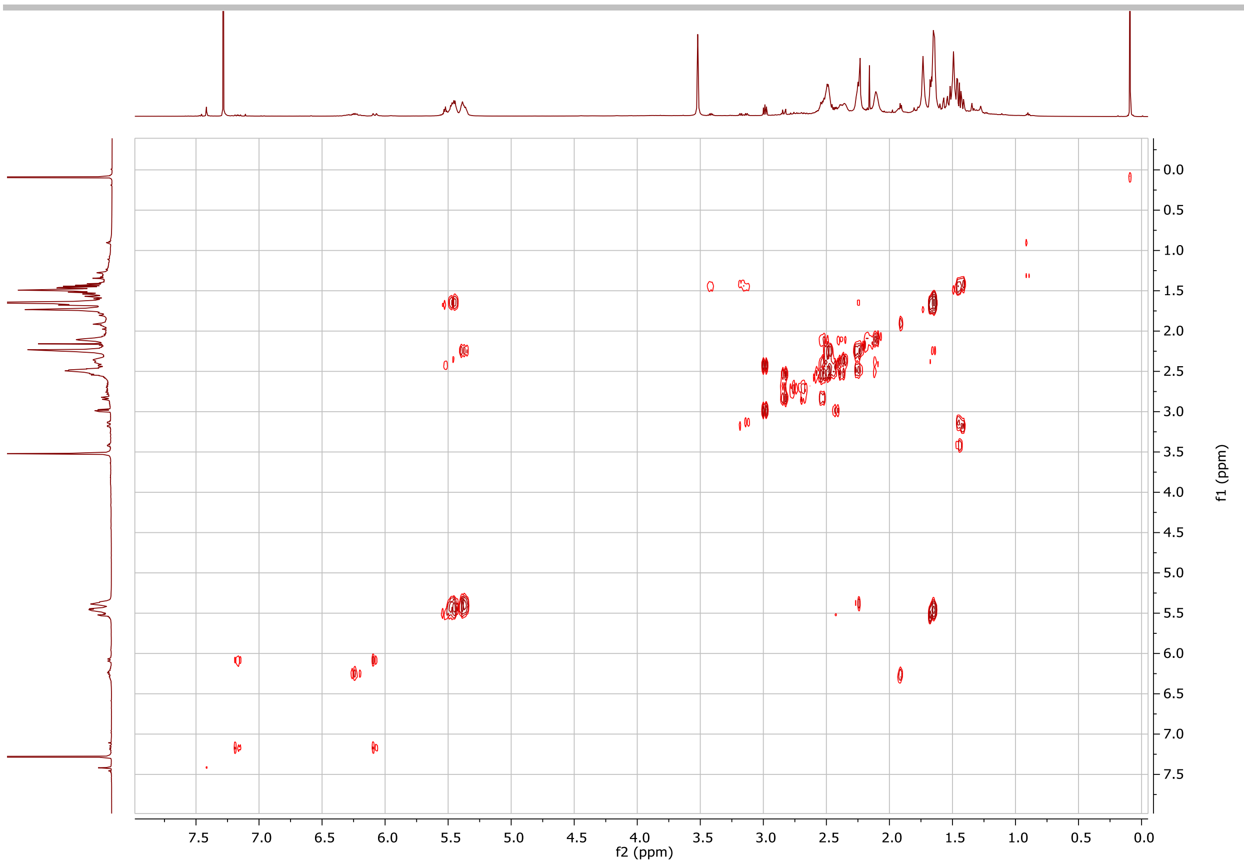

Figure S125: ^{1}H-^{1}H COSY spectrum for **16b** recorded at 500 MHz in DMSO-d6.

References

[1] K. A. K. Pahirulzaman, K. Williams, C. M. Lazarus, in *Methods Enzymol.*, Academic Press, **2012**, pp. 241–260.
[2] K. Blin, S. Shaw, K. Steinke, R. Villebro, N. Ziemert, S. Y. Lee, M. H. Medema, T. Weber, *Nucleic Acids Res.* **2019**, *47*, W81–W87.
[3] V. Solovyev, P. Kosarev, I. Seledsov, D. Vorobyev, *Genome Biol.* **2006**, *7 Suppl 1*, S10.
[4] T. Carver, S. R. Harris, M. Berriman, J. Parkhill, J. A. McQuillan, *Bioinformatics* **2012**, *28*, 464–469.
[5] M. G. Steiger, M. Vitikainen, P. Uskonen, K. Brunner, G. Adam, T. Pakula, M. Penttilä, M. Saloheimo, R. L. MacH, A. R. Mach-Aigner, *Appl. Environ. Microbiol.* **2011**, *77*, 114–121.
[6] R. Andrade, W. A. Ayer, P. P. Mebe, *Can. J. Chem.* **1992**, *70*, 2526–2535.
[7] L. S. Trifonov, H. Hilpert, P. Floersheim, A. S. Dreiding, D. M. Rast, R. Skrivanova, L. Hoesch, *Tetrahedron* **1986**, *42*, 3157–3179.
[8] A. Al Fahad, A. Abood, K. M. Fisch, A. Osipow, J. Davison, M. Avramović, C. P. Butts, J. Piel, T. J. Simpson, R. J. Cox, *Chem. Sci.* **2014**, *5*, 523–527.
[9] K. Washida, N. Abe, Y. Sugiyama, A. Hirota, *Biosci. Biotechnol. Biochem.* **2009**, *73*, 1355–1361.

Author Contributions

Conception of the work, data collection, data analysis and interpretation (LK, EB, EJS, RJC); Drafting the article (LK, EB, EJS, RJC); Critical revision of the article (LK, EB, EJS, RJC); Funding acquisition (EJS, RJC).
